# Supplementary material for: Operational priorities for engaging with India’s private healthcare sector for the control of tuberculosis: a modelling study
Source: BMJ Open. 2024 Mar 19;14(3):e069304. doi: 10.1136/bmjopen-2022-069304 (PMC10952976; doi:10.1136/bmjopen-2022-069304)
Supplement: Supplementary data [file bmjopen-2022-069304supp001.pdf]

Appendix 3

The value and cost efficiency of private sector engagement

1. Materials and methods

Below, we describe the model, including its structure, governing equations, and table of parameters. The model was implemented in MATLAB.

1.1. Model structure

We modelled TB transmission amongst adults (>15 years old) in a typical urban setting in India. We modelled the fragmented healthcare system, by differentiating between the public and private sector (1–7). We further divided the private sector by engagement status, CB-NAAT use and FDC use (see main text for more details on these categories). We calibrated the model to TB prevalence, annual risk of infection, and mortality (table 1). We also modelled the acquisition and transmission of DR-TB, but ignored HIV status as well as distinctions between pulmonary and extrapulmonary TB. The overall model structure is illustrated schematically in fig 1.

For a given set of model parameters, we initially simulated the model to equilibrium after introducing TB into a disease-free population. From 1997 to 2007 and its maintenance until the present day, we incorporated the scale-up of RNTCP (India's national TB programme) and its impact on the TB epidemic (8). From 1997, we also incorporated population growth. From 2017 to 2020, we simulated the scale-up of PPSA services to current 2019 levels. We then simulated the model forward from 2020 to 2035.

1.2. Governing equations for the mathematical transmission model

The equations correspond to the model described in fig 1. State variables (capital letters) are as listed in table S1, while model parameters (lower-case and Greek letters) are as listed in tables S2-S5.

Model stage	Description
U	Uninfected
L	Latent infection
I_q	Active TB
$\boldsymbol{Dx_q^{(pu)}}$	Sought care, awaiting TB diagnosis, public sector
$\boldsymbol{Dx_q^{(pr)}}$	Sought care, awaiting TB diagnosis, private sector. This is split further depending on engagement status and CB-NAAT use (table S6)
$\boldsymbol{Tx_q^{(FL:pu)}}$	Undergoing first line TB treatment, public sector
$\boldsymbol{Tx_q^{(FL:pr)}}$	Undergoing first line TB treatment, private sector. This is split further depending on FDC use and adherence support offered (table S6).
$\boldsymbol{Tx^{(SL)}}$	Undergoing second line TB treatment.
$\boldsymbol{E_q}$	Between seeking care, after misdiagnosis or loss-to-follow-up
$R^{(L)}$	Recovered after treatment completion, low risk of relapse
$R^{(H)}$	Recovered after self-cure or incomplete treatment, high risk of relapse
**Subscript**	**Description**
q	Drug resistance status (0 = DS-TB, 1 = DR-TB)

Table S1. Model stages and subscript description

Parameter	Symbol	Value		Source
**TB natural history**				
Mean rate of transmission per TB case	β	10.42 yr^{-1} [7.28-17.24]		Model estimate
Relative rate of transmission between DS- and DR-TB	d	0.61 yr^{-1} [0.49-0.79]		Model estimate
Rate of MDR acquisition	m_a	Public sector/active providers that offer either adherence support of government supplied FDCs	0.01 [0.008-0.012]	(9)
	m_b	All private providers that are unengaged, inactive, or active that do not offer government supplied FDCs or adherence support	0.05 [0.04-0.06]	
Proportion of TB infections undergoing rapid progression	θ	0.11 [0.09-0.15]		(10,11)
Per capita hazard rate of progression to active disease from latency	ρ	0.001 yr^{-1} [0.0003-0.0024]		(11,12)
Per-capita hazard rate of relapse	r_1	Following self-cure/treatment default	0.14 yr^{-1} [0.10-0.18]	(9,13)

	r_2	Following treatment cure, >2 years	0.002 yr^{-1} [0.001-0.003]	
Stabilisation of relapse risk following treatment	ς	0.5 yr^{-1} [0.4-0.6]		(13): most relapse occurs in the first two years after treatment
Per-capita hazard rate of spontaneous cure	φ	0.17 yr^{-1} [0.13-0.21]		(14)
Per-capita hazard rate of TB mortality	μ_1	0.18 [0.13-0.25]		Model estimate
Proportion reduction in susceptibility to reinfection due to previous infection	π	0.21 [0.15-0.25]		(15)
**Demographics**				
Per-capita hazard rate of background mortality	μ_2	0.015 yr^{-1}		World Bank estimates: corresponds to an average lifespan of 69 years
Per-capita rate of population growth	σ	0.02 [0.01-0.025]		(16)

Table S2. List of model parameters: TB natural history

Parameter	Symbol	Value		Source
**TB services: diagnosis**				
Proportion of TB cases seeking care in the public sector	η	0.50 [0.40-0.60]		(2,17–19)
Proportion of private providers that are engaged	e	Ahmedabad	0.50	Data provided by CHAI
		Delhi	0.73	
Heterogeneity index of the behaviour of engaged private providers	$\propto$	Ahmedabad	0.39 [0.32-0.46]	Data provided by CHAI
		Delhi	0.32 [0.25-0.41]	
Proportion of engaged private providers that are active	a	Ahmedabad	0.80	Data provided by CHAI
		Delhi	0.44	
Per-capita hazard rate of initial careseeking for TB symptoms	δ_1	1.38 yr^{-1} [0.88-2.38]		Model estimate
Per-capita hazard rate of repeat careseeking for TB symptoms (following missed diagnosis)	δ_2	12 yr^{-1} [6-26]		Assumption: corresponds to range of 2 weeks to 2 months

Proportion of TB cases diagnosed correctly, per careseeking attempt in routine public sector TB services	ε_{sn}	0.84 [0.80-0.85]		(20,21)
Specificity of diagnosis in routine public sector TB services	ε_{sp}	0.98 [0.97-0.99]		(20)
CB-NAAT sensitivity	x_{sn}	0.89 [0.85-0.92]		(22)
CB-NAAT specificity	x_{sp}	0.99 [0.98-1.00]		(22)
Proportion of active private providers that send samples for confirmatory CB-NAAT testing 100% of the time	p_{Xpert1}	Ahmedabad	0.09	Data provided by CHAI
		Delhi	0.04	
Proportion of active private providers that never send samples for confirmatory CB-NAAT testing	p_{Xpert0}	Ahmedabad	0.55	Data provided by CHAI
		Delhi	0.66	
Proportion of samples that are sent off for confirmatory CB-NAAT testing amongst active private providers that occasionally send samples off for confirmatory CB-NAAT testing	x_p	Ahmedabad	0.23	Data provided by CHAI
		Delhi	0.15	
Clinical diagnosis sensitivity (including chest X-ray)	c_{sn}	0.75 [0.65-0.80]		(21)
Clinical diagnosis specificity (including chest x-ray)	c_{sp}	0.85 [0.80-0.95]		(21)
Proportion of DR-TB cases receiving drug susceptibility testing at point of TB diagnosis	$\text{Þ}^{(pu)}$	Public sector	0.45 [0.40-0.50]	(23)
	$\text{Þ}^{(pr0)}$	Private sector (providers not using CB-NAAT)	0	
	$\text{Þ}^{(pr1)}$	Private sector (providers using CB-NAAT)	1.00	

Table S3. List of model parameters: TB diagnosis

Parameter	Symbol	Value		Source
**TB services: treatment**				
Proportion of diagnosed TB cases initiating TB treatment	ω	0.87 [0.85-0.90]		(20). Assumed that patients from active providers are referred to the public sector to initiate second line treatment.
Amongst DR-TB cases failing first-line treatment, the proportion successfully transferred to second-line treatment, public sector	p_{SL}	0.87 [0.85-0.92]		Assumption
TB treatment initiation delay	ϕ	52 yr^{-1}		Assumed: corresponds to a mean treatment delay of 1 week in routine TB care
Proportion of patients that agree to adherence support	p_{adh1}	0.92		Data provided by CHAI
Proportion of active providers that agree to adherence support	p_{adh2}	Ahmedabad	0.93	Data provided by CHAI
		Delhi	0.93	
Proportion of active providers that agree to government supplied FDCs	p_{fdc}	Ahmedabad	0.39	Data provided by CHAI
		Delhi	0.07	
Per-capita hazard rate of treatment completion	τ	First line	2 yr^{-1}	Corresponds to a duration of 6 months
	τ_{mdr}	Second line	0.5 yr^{-1}	Corresponds to a duration of 2 years
Proportion of TB patients that do not complete treatment	χ_{Pu}	First line, public sector	0.15 [0.13-0.17]	(23,24), data provided by CHAI
	χ_{Pr_Z}	First line, private sector: unengaged/inactive/active but no adherence support of government supplied FDCs offered	0.48 [0.45-0.55]	
	χ_{Pr_F}	First line, private sector: active (government	0.17 [0.16 -0.19]	

		supplied FDCs offered, no adherence support offered)		
	$\chi_{\text{Pr\_A}}$	First line, private sector: active (no government supplied FDCs offered, adherence support offered)	0.21 [0.20-0.25]	
	$\chi_{\text{Pr\_AF}}$	First line, private sector: active (government supplied FDCs and adherence support offered)	0.15 [0.13-0.17]	
	χ_{mdr}	Second line	0.50 [0.45-0.60]	

Table S4. List of model parameters: TB treatment

Parameter	Symbol	Value	Source
**Costs**			
Cost per active TB disease diagnosis (passive case finding)	C_D	USD 18.30	(25)
Cost of first line treatment for active TB disease per person	C_T	USD 23.70 per treatment-month	(25)

Table S5. List of model parameters: TB costs

Label	Description
**Private sector diagnosis classification**	
pr1	Unengaged private providers
pr2	Engaged, inactive private providers
pr3	Engaged, active private providers that send samples for confirmatory CB-NAAT testing 100% of the time
pr4	Engaged, active private providers that send samples for confirmatory CB-NAAT testing occasionally, CB-NAAT
pr5	Engaged, active private providers that send samples for confirmatory CB-NAAT testing occasionally, no CB-NAAT
pr6	Engaged, active private providers that never send samples for confirmatory CB-NAAT testing
**Private sector first-line treatment classification**	
prZ1	Treatment offered by inactive and unengaged private providers
prAF	Engaged, active private providers that offer adherence support and government supplied FDCs
prA	Engaged, active private providers that only offer adherence support
prF	Engaged, active private providers that only offer government supplied FDCs
prZ2	Engaged, active private providers that offer neither adherence support or government supplied FDCs

Table S6. Private sector classification

Label	Classes
S_1	pu, prZ1, prZ2, prAF, prA, prF
S_2	pr1, pr2, pr5, pr6
S_3	pr3, pr4

Table S7. Notation

Uninfected stage

$$\frac{dU}{dt} = T - (\lambda_q + \mu_2)U \quad (1)$$

where term T represents births. To maintain a constant population for simplicity, we assumed that T is equivalent to the number of deaths occurring in the model.

Latent TB infection

$$\frac{dL_q}{dt} = (1-\theta)\lambda_q U + \pi\lambda_q(1-\theta) - (\rho + \mu_2 + \lambda_q)L_q \quad (2)$$

Active disease, pre-careseeking

$$\frac{dI_q}{dt} = \theta\lambda_q U + \pi\lambda_q\theta + \rho L_q + r_1 R^{(H)} + r_2 R^{(L)} - (\varphi + \delta_1 + \mu_1 + \mu_2)I_q \quad (3)$$

where the subscript q denotes drug resistance status:

$$q = \begin{cases} 0 & DS\,TB \\ 1 & DR\,TB \end{cases}$$

We assumed that, when presenting for care, a proportion η of patients visit the public sector, the remainder $(1-\eta)$ visiting the private sector. We divided the private sector further depending on whether private providers are engaged by JEET or not, and whether engaged providers are active or inactive, in notifying TB. Finally, active providers were categorised by their use of CB-NAAT (table S6-S7). The proportion of patients that visit each different type of provider is summarised in table S8.

**Provider type**	**Notation**	**Formula**
Public	$v^{(pu)}$	$v^{(pu)} = \eta$
Unengaged, private	$v^{(pr1)}$	$v^{(pr1)} = (1-\eta)(1-h^{\propto})^{*}$
Engaged, inactive, private	$v^{(pr2)}$	$v^{(pr2)} = (1-\eta)h^{\propto}(1-a)$
Engaged, active, private (always use CB-NAAT)	$v^{(pr3)}$	$v^{(pr3)} = (1-\eta)h^{\propto}ap_{CBNAAT1}$
Engaged, active, private (occasionally use CB-NAAT, patient receives an CB-NAAT test)	$v^{(pr4)}$	$v^{(pr4)} = (1-\eta)h^{\propto}a(1-p_{CBNAAT1}-p_{CBNAAT0})x_p$
Engaged, active, private (occasionally use CB-NAAT, patient does not receive an CB-NAAT test)	$v^{(pr5)}$	$v^{(pr5)} = (1-\eta)h^{\propto}a(1-p_{CBNAAT1}-p_{CBNAAT0})(1-x_p)$
Engaged, active, private (non CB-NAAT user)	$v^{(pr6)}$	$v^{(pr6)} = (1-\eta)h^{\propto}ap_{CBNAT0}$

Table S8. Proportion of patients that visit different providers. Private providers under the classifications pr1, pr2, pr5 and pr6 rely on clinical diagnosis, whereas private providers under the classification pr3 and pr4 employ CB-NAAT testing. *h is the proportion of private providers that have been engaged by the PPSA, and α is a 'heterogeneity index' governing the distribution of patient load across all providers (see fig S1).

The following equations describe the different pathways for patients that seek care with the different providers described above:

Seeking care, awaiting TB diagnosis

$$\frac{dDx_q^{(pu)}}{dt} = v^{(pu)}\delta_1 I_q + v^{(pu)}\delta_2 E_q$$

$$-\left[\varepsilon_1\omega\phi\left(1-\mathrm{s}_q^{(pu)}\right) + \left(\varepsilon_1\omega\phi\mathrm{s}_q^{(pu)}\right)_q + (1-\omega\varepsilon_1)\phi\mathrm{s}_q^{(pu)} + \varphi + \mu_1 + \mu_2\right] Dx_q^{(pu)} \quad (4)$$

$$\frac{dDx_q^{(s)}}{dt} = v^{(s)}\delta_1 I_q + v^{(s)}\delta_2 E_q$$

$$-\left[\varepsilon_3\omega\phi\left(1-\mathrm{s}_q^{(pr0)}\right) + (\varepsilon_3\omega\phi\mathrm{s}_q^{(pr0)})_q + (1-\omega\varepsilon_3)\phi\mathrm{s}_q^{(pr0)} + \varphi + \mu_1 + \mu_2\right] Dx_q^{(s)} \quad (5)$$

$$\frac{dDx_q^{(h)}}{dt} = v^{(h)}\delta_1 I_q + v^{(h)}\delta_2 E_q$$

$$-\left[\varepsilon_2\omega\phi(1-\mathrm{s}_q^{(pr1)}) + (\varepsilon_2\omega\phi\mathrm{s}_q^{(pr1)})_q + (1-\omega\varepsilon_2)\phi\mathrm{s}_q^{(pr1)} + \varphi + \mu_1 + \mu_2\right] Dx_q^{(h)} \quad (6)$$

Where superscript (s) represents either pr1, pr2, pr5 or pr6 and superscript (h) represents either pr3 or pr4 as defined in table S8.

Patients that are misdiagnosed (as false-negative) enter the compartment E, to re-initiate careseeking after a delay:

Temporarily dropped out of careseeking

$$\frac{dE_q}{dt} = (1-\omega\varepsilon_1)\phi Dx_q^{(pu)} + \sum_{i\in S_3}[(1-\omega\varepsilon_2)\phi]Dx_q^{(i)} - (\varphi + \delta_2 + \mu_1 + \mu_2)E_q$$

when q=0 (7)

$$\frac{dE_q}{dt} = (1-\omega\varepsilon_1)\phi\mathrm{s}_{pu}Dx_q^{(pu)} + \sum_{i\in S_2}[(1-\omega\varepsilon_3)\phi\mathrm{s}_{pr0}]Dx_q^{(i)} + \sum_{i\in S_3}\left[(1-\omega\varepsilon_2)\phi\mathrm{s}_{pr1}\right]Dx_q^{(i)}$$

$$+\sum_{i\in S_1}\left[\tau(1-p_{SL}) + \frac{\tau\chi_i}{1-\chi_i}\right]Tx_q^{(i)} - (\varphi + \delta_2 + \mu_1 + \mu_2)E_q$$

when q=1 (8)

where the summand represents patients not being cured by first-line treatment, but not being transferred to second-line treatment. Subscript S_1, S_2, and S_3 are defined in table S7.

Treatment

We categorised active private providers by the treatment services offered: adherence support and government supplied FDCs (prAF); adherence support only (prA); government supplied FDCs only (prF); or neither (prZ2) (table S6). The proportion of patients at active providers that receive the different treatment services offered is defined in table S9.

Provider type	Notation	Formula
Adherence support and government supplied FDCs	$v^{(prAF)}$	$v^{(prAF)} = p_{adh1}p_{adh2}p_{fdc}$
Adherence support only	$v^{(prA)}$	$v^{(prA)} = p_{adh1}p_{adh2}(1 - p_{fdc})$
Government supplied FDCs only	$v^{(prF)}$	$v^{(prF)} = (1 - p_{adh1}p_{adh2})p_{fdc}$
Neither adherence support or government supplied FDCs	$v^{(prZ)}$	$v^{(prZ)} = (1 - p_{adh1}p_{adh2})(1 - p_{fdc})$

Table S9. Proportion of patients at active providers that receive treatment services.

Governing equations for the different Tx compartments are:

$$\frac{dTx_q^{(FL:y)}}{dt} = \varepsilon_i \phi \omega v^{(pr\_y)} Dx_q^{(y)} - (m_y + \tau + \frac{\tau\chi_y}{1-\chi_y} + \mu_2) Tx_q^{(FL:y)} \quad \text{when q = 0} \quad (9)$$

$$\frac{dTx_q^{(FL:y)}}{dt} = \varepsilon_i \phi \omega v^{(pr\_y)} (1 - s_y) Dx_q^{(y)} + m_y Tx_{q=0}^{(FL:y)} - (\tau p_{SL} + [\tau(1 - p_{SL}) + \frac{\tau\chi_y}{1-\chi_y}] + \mu_2) Tx_q^{(FL:y)} \quad \text{when q = 1} \quad (10)$$

$dTx_q^{(FL:y)}$ represents the different Tx compartments (pu, prZ1, prAF, prA, prF and prz2), defined in table S6. The parameter ε_i is the proportion of patients tested that are correctly diagnosed (in other words, the sensitivity of the test) and is dependent on where the patient is diagnosed: ε_1 when Dx^{pu}; ε_3 when Dx^{pr1}, Dx^{pr2}, Dx^{pr5}, Dx^{pr6}; and ε_2 when Dx^{pr3}, Dx^{pr4}. The parameter ϕ is the treatment initiation delay (assumed conservatively to be 1 week across all providers) and ω is the proportion of diagnosed TB cases initiating TB treatment (assumed to be 0.87 across all providers) (20). $v^{(pr\_y)}$, described above, applies only to TB patients that are diagnosed by active private providers. s_y is the proportion of MDR-TB cases receiving drug susceptibility testing at the point of TB diagnosis and is dependent on the amount of CB-NAAT testing. We assume the proportion is 0.45, 1.00 and 0 in the public sector ($s^{(pu)}$), respectively amongst private providers that always use CB-NAAT, and amongst private providers that do not use CB-NAAT, respectively (23). Thus, $1 - s_y$ represents TB patients with DR-TB that are not tested for drug resistance and are therefore mistakenly initiated onto first-line treatment. The parameter m_y is the rate of MDR acquisition while on first-line treatment and is assumed to be higher amongst all private providers (m_b) except active providers that offer government supplied FDCs and adherence support, than public sector providers (m_a) (9). The parameter τ is the per-capita rate of treatment completion for first line treatment and

its value corresponds to a duration of 6 months.

The parameter p_{SL} applies only to TB patients with DR-TB and is defined as the proportion of DR-TB cases that are successfully transferred to second-line treatment following failure of first-line treatment. The parameter χ_y is the proportion of TB cases that do not complete, or are non-adherent to, first line treatment; it is dependent on the quality of treatment services offered by providers and is informed by programmatic data (tables 2-5).

The following equation represents the number of DR-TB patients receiving second-line treatment:

$$\frac{dTx_q^{(SL)}}{dt} = \varepsilon_1\omega\phi s_{pu}Dx_q^{(pu)} + \varepsilon_3\omega\phi s_{pr0}\sum_{i\in S_2} Dx_q^{(i)} + \varepsilon_2\omega\phi s_{pr1}\sum_{i\in S_3} Dx_q^{(i)}$$

$$+\tau p_{SL}\sum_{i\in S_1} Tx_q^{(i)} - (\tau_{mdr} + \frac{\tau_{mdr}\chi_{\mathrm{mdr}}}{1-\chi_{\mathrm{mdr}}} + \mu_2)Tx_q^{(SL)}$$

(11)

Where S_1, S_2 and S_3 are sets of indices defined in table S7.

TB patients that successfully complete TB treatment, recover with a low risk of relapse. Governing equations for those with low risk of relapse are:

Recovery, low risk of relapse

$$\frac{dR^{(L)}}{dt} = \tau\sum_{i\in S_1} Tx_q^{(i)} + \varsigma R^{(H)} - \left(r_2 + \mu_2 + \lambda_q\right)R^{(L)}$$

(12)

Where S_1 is the set of indices defined in table S7.

On the other hand, those recovered from TB with a high risk of relapse (either from previous inadequate treatment or self-cure) have governing equations:

Recovery, high risk of relapse

$$\frac{dR^{(H)}}{dt} = \frac{\tau\chi_{pu}}{1-\chi_{pu}} Tx_q^{(FL:pu)} + \sum_{i\in S_1} (\frac{\tau\chi_{(i)}}{1-\chi_{(i)}}) Tx_q^{(i)} + \varphi \sum_{i\in[S_{2,}S_3]} [Dx_q^{(pu)} + Dx_q^{(i)} + E_q + I_q]$$

$$-(\varsigma + r_1 + \mu_2 + \lambda_q)R^{(H)} \qquad (13)$$

Where S_1, S_2 and S_3 are sets of indices defined in table S7.

The force of infection can be written as:

$$\lambda_q = \frac{\beta\left\{\sum_{i\in[S_2,S_3]}[I_q + Dx_q^{pu} + Dx_q^{(i)} + E_q]\right\} + d_q\beta\left\{\sum_{i\in[S_2,S_3]}[I_q + Dx_q^{pu} + Dx_q^{(i)} + E_q]\right\}}{N} \qquad (14)$$

Where S_2 and S_3 are sets of indices defined in table S7.

Non-TB symptomatic population

To count the number of false positive diagnoses that arise, we modelled a non-TB symptomatic population (fig 1). We defined non-TB symptomatics as patients who do not have TB, but who have symptoms that would make them eligible for testing for TB. We assumed this population to be independent of the TB population described above.

Non-TB symptomatics seeking care

$$\frac{dU^{(NTB)}}{dt} = y_i\phi\omega Dx^{(NTB)} + \tau Tx^{(NTB)} - \delta_1 v^{(x)} U^{(NTB)} \qquad (15)$$

where $v^{(x)}$ is drawn from equations 4 to 10, depending on the type of provider a patient seeks care from, and where y_i represents either the specificity of TB diagnosis in the public sector (y_1), specificity of CB-NAAT (y_2) or the specificity of clinical diagnosis (y_3).

Awaiting diagnosis

$$\frac{dDx^{(NTB)}}{dt} = \delta_1 v^{(x)} U^{(NTB)} - \phi\omega Dx^{(NTB)} \qquad (16)$$

Undergoing (unnecessary) first-line TB treatment

$$\frac{dTx^{(NTB)}}{dt} = (1 - y_i)\phi\omega Dx^{(NTB)} - \tau Tx^{(NTB)} \quad (17)$$

We assumed the total population size of the non-TB symptomatic population remains constant; we determined the size of this population so that for every true positive TB diagnosis, approximately nine uninfected individuals who present for diagnosis with symptoms of TB are also tested for TB (20,26–30).

1.3. Model calibration

We performed calibrations using Adaptive Bayesian MCMC (table 1 -5)(31), allowing propagation of uncertainty from model inputs to projections in a systematic way.

We define the posterior distribution as:

$$p(\theta|D) \propto L(D|\theta)\,.\,\pi(\theta) \quad (18)$$

where D is data; θ is a vector of model inputs subject to uncertainty; L is the likelihood of the data given θ, and π is the prior distribution. We assumed uniform distributions for the prior distributions. We defined the likelihood by fitting log-normal distributions to the calibration targets (see table 1).

Once the MCMC converged, we removed the burn-in period (10,000 iterations) and selected every 50th sample to reduce any autocorrelation. This provided 1200 samples from the posterior distribution.

2. Supporting figures

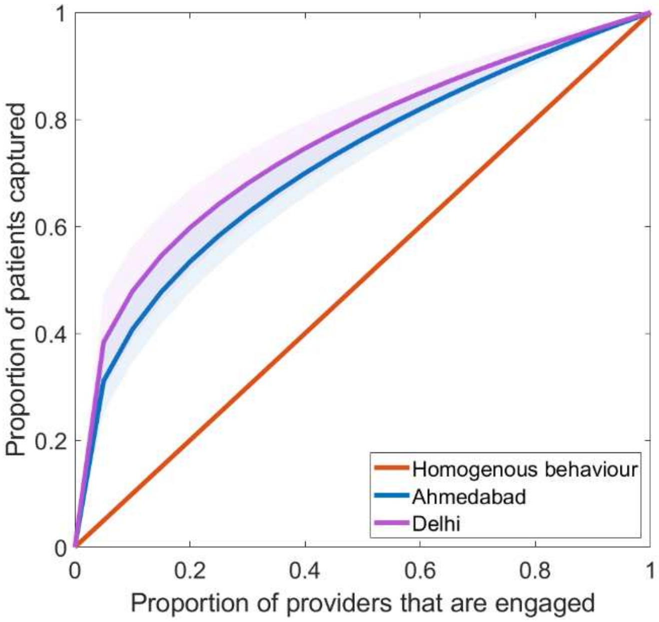

Figure S1. Heterogeneity in the patient share of private providers across Ahmedabad and Delhi. Homogenous patient share (red line) corresponds to a scenario where all private providers have the same numbers of patients. Under this assumption, increasing the proportion of engaged providers will linearly increase the proportion of patients benefiting from a PPSA. In reality, there are a few large private providers that manage most TB patients, and many smaller providers that manage few patients. The larger the area under the curve, the greater the heterogeneity. If the providers that capture the most patients are already engaged by JEET, increasing the proportion of engaged providers further will have little incremental benefit. Therefore, assuming homogenous patient share can overestimate the impact on TB epidemiology. As described in the main text, the 'heterogeneity index' controls the curvature of these plots. Table S11 shows the heterogeneity index calculated for each city.

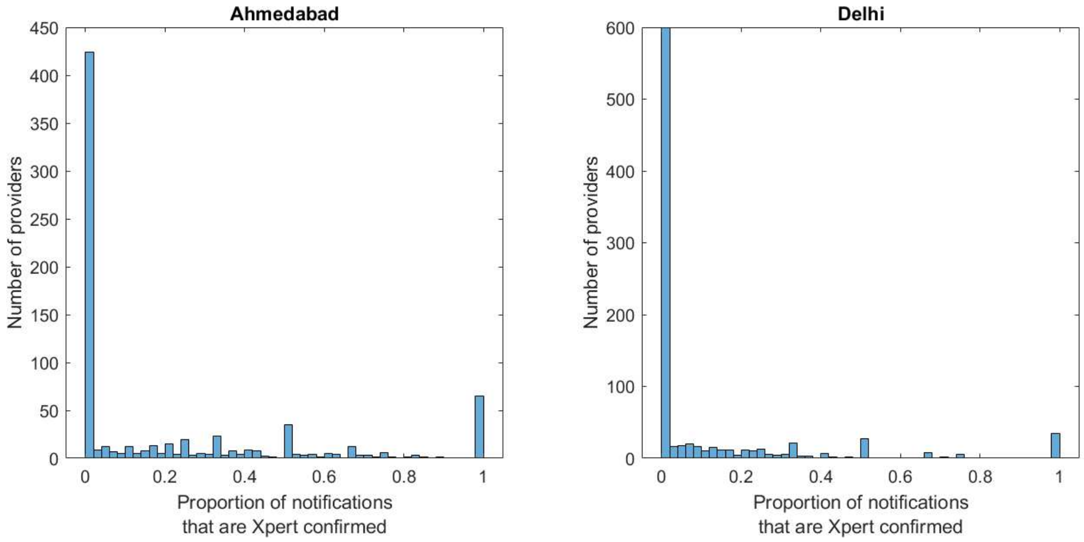

Figure S2. CB-NAAT use amongst active private providers in Ahmedabad and Delhi in 2019. From this data, we classified active providers into three categories: those that always use CB-NAAT, those that use CB-NAAT occasionally, and those that never use CB-NAAT. To be classified as occasionally using CB-NAAT, we assume that providers need to use CB-NAAT for at least 1% of notifications and less than 100% of notifications.

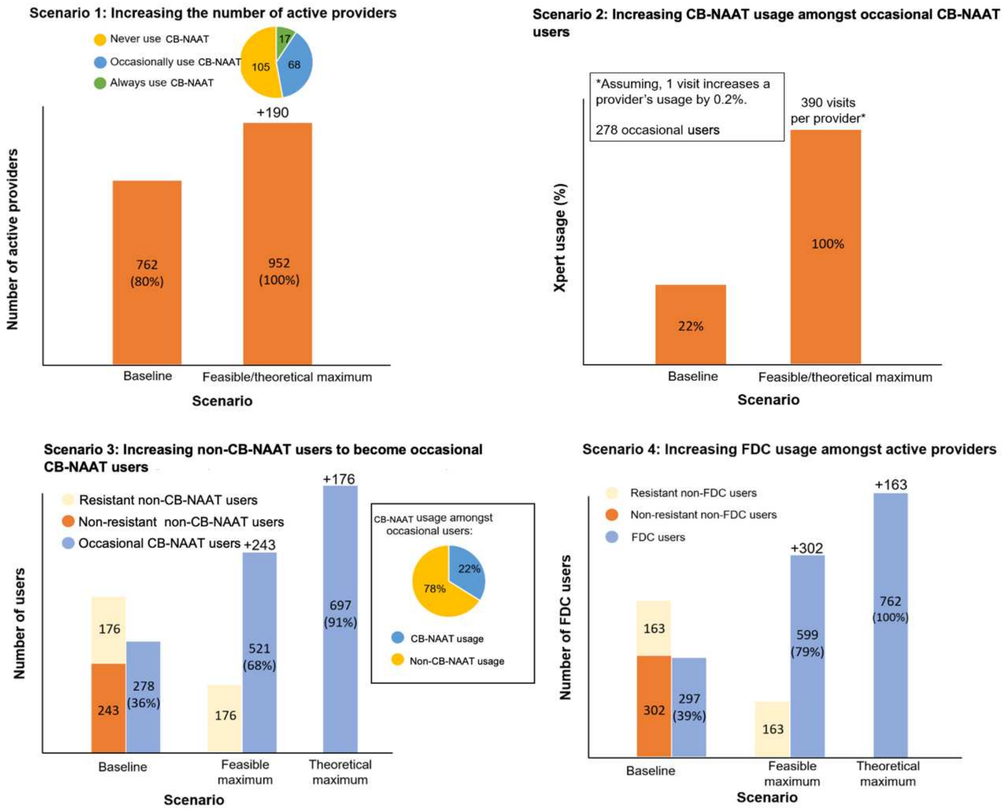

Figure S3. Feasible and theoretical maxima for each type of service provision, Ahmedabad. *Scenario 1:* We assume all inactive providers (e.g., 190) are converted into active providers. We assume no inactive providers are resistant, thus the feasible and theoretical maximum scenarios are the same. Newly active providers are assumed to have the same behaviour as pre-existing active providers; in the case of Ahmedabad, 9% use CB-NAAT 100% of the time, 36% use CB-NAAT occasionally (with an average CB-NAAT usage of 22%), 55% never use CB-NAAT, 98% offer adherence support and 39% consistently use governmental FDCs. For example, of the 190 newly active providers, 17 providers use CB-NAAT 100% of the time, 68 use CB-NAAT occasionally, and 105 never use CB-NAAT. *Scenario 2:* At baseline, occasional CB-NAAT users use CB-NAAT 22% of the time; this is increased to 100% under the feasible and theoretical maximum scenario. We assume no occasional users are resistant to increasing their CB-NAAT usage. It is estimated that one visit by a field officer increases a provider's usage of CB-NAAT by 0.2%. At baseline, Ahmedabad has 278 occasional CB-NAAT users. *Scenario 3:* At baseline, there are 419 non-CB-NAAT users and 278 occasional users. Of the 419 non-CB-NAAT users, it is estimated that 42% are resistant to using CB-NAAT; thus, only 243 non-CB-NAAT users can be converted to become an occasional user. At the theoretical maximum, the percentage of active providers that use CB-NAAT occasionally is 91%; the remaining 9% are providers that always use CB-NAAT. *Scenario 4:* At baseline, there are 465 non-FDC users and 297 consistent FDC users. Of the 465, 35% are resistant to FDCs.

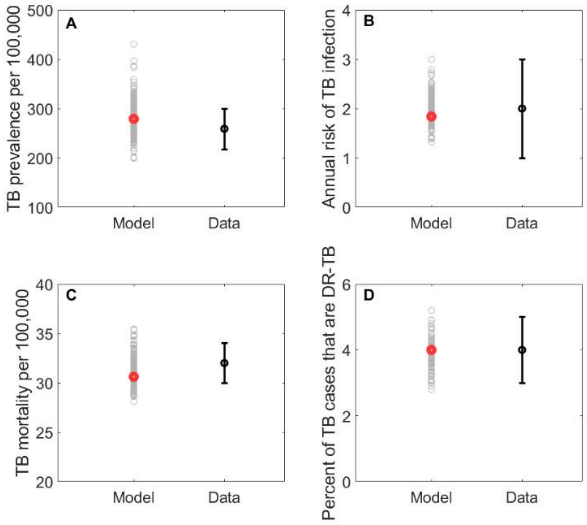

Figure S4. Model fits to data. Data points are described in table 1. Panel A – TB prevalence per 100,000 in a typical urban Indian setting; B – Annual risk of TB infection in a typical urban Indian setting; C – TB deaths per 100,000 in 2019, India; D – Percent of TB cases that are drug-resistant TB in 2019

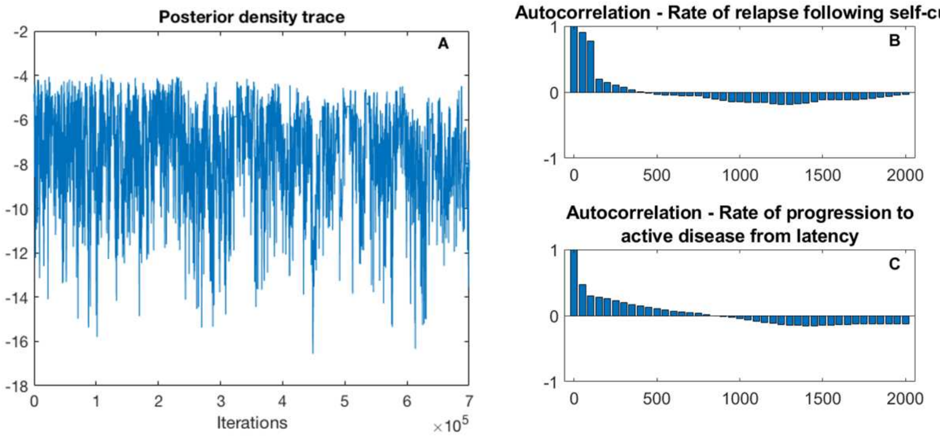

Figure S5. MCMC diagnostics. Panel A shows the posterior density trace, excluding the ‘burn-in’ period. Panel B and C show autocorrelation plots for two selected parameters (rate of relapse following self-cure and the rate of progression to active disease from latency).

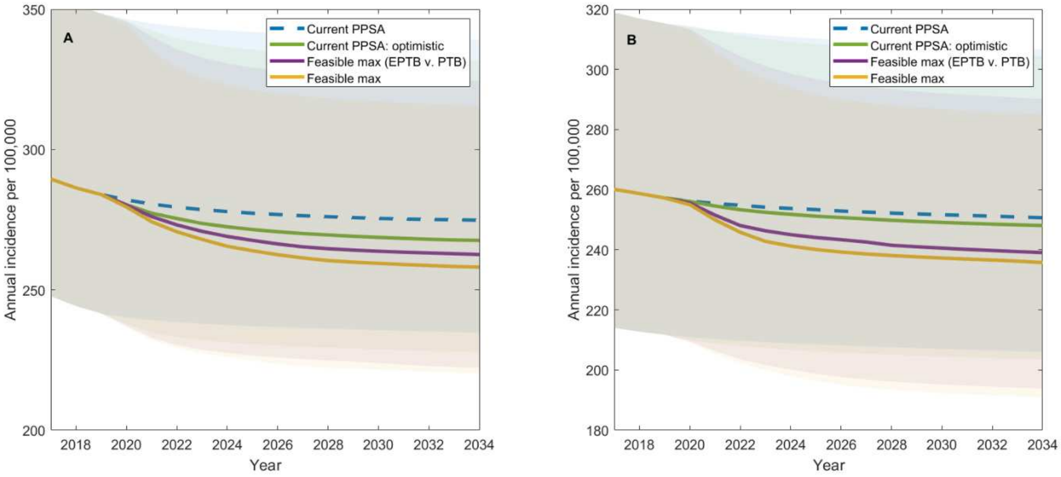

Figure S6. Sensitivity analysis on model projections for the impact of increasing all service provisions to their feasible maximum has on TB incidence in Ahmedabad and Delhi between 2020 and 2035. Panel A shows the projected trajectory of TB incidence for Ahmedabad and Panel B for Delhi, assuming current PPSA scale up between 2017 and 2020 (blue dashed line) and its maintenance indefinitely; assuming current PPSA scale up between 2017 and 2020 whilst optimistically assuming that active providers are continuously active (green line); service provisions are increased to their feasible maximum from 2020 to 2021 and maintained indefinitely, but assuming that 50% of TB cases that present to care are extra-pulmonary TB (purple line); that service provisions are increased to their feasible maximum from 2020 to 2021 and maintained indefinitely, as presented in the main text (yellow line).

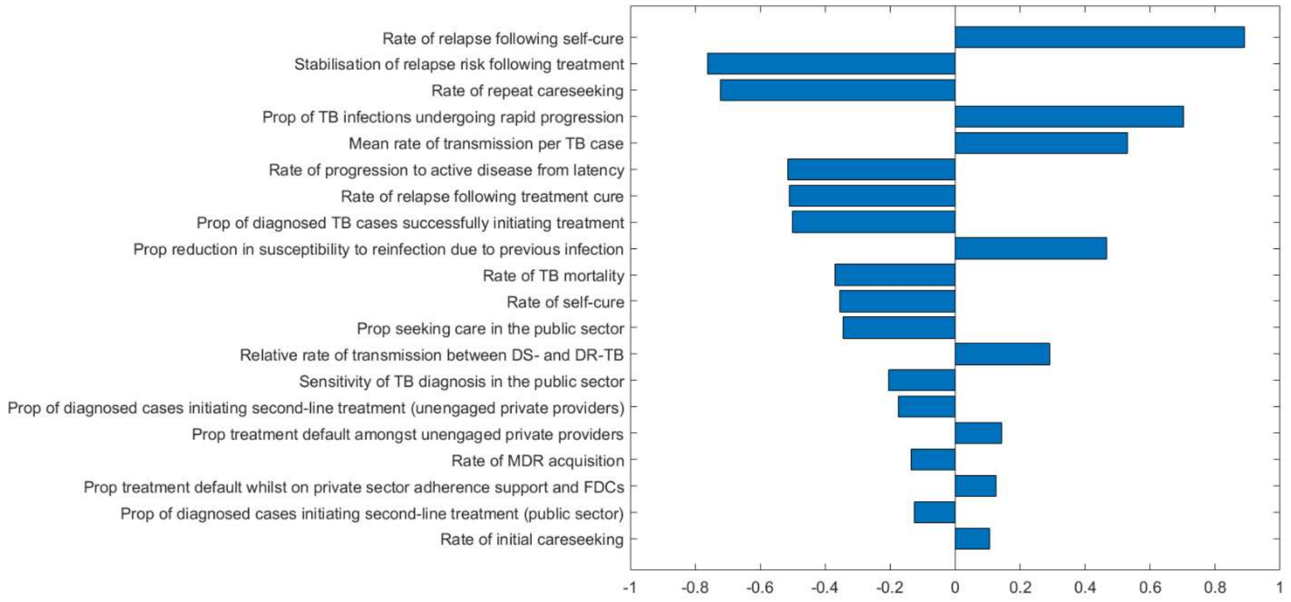

Figure S7. Multivariate sensitivity analysis of impact for Ahmedabad. We used partial rank correlation coefficient (PRCC) to examine which parameter listed in tables 2-5 the output cases averted is most sensitive towards. Larger bars represent more sensitive parameters. Shown are the 20 most influential model parameters, in decreasing order of sensitivity from top to bottom.

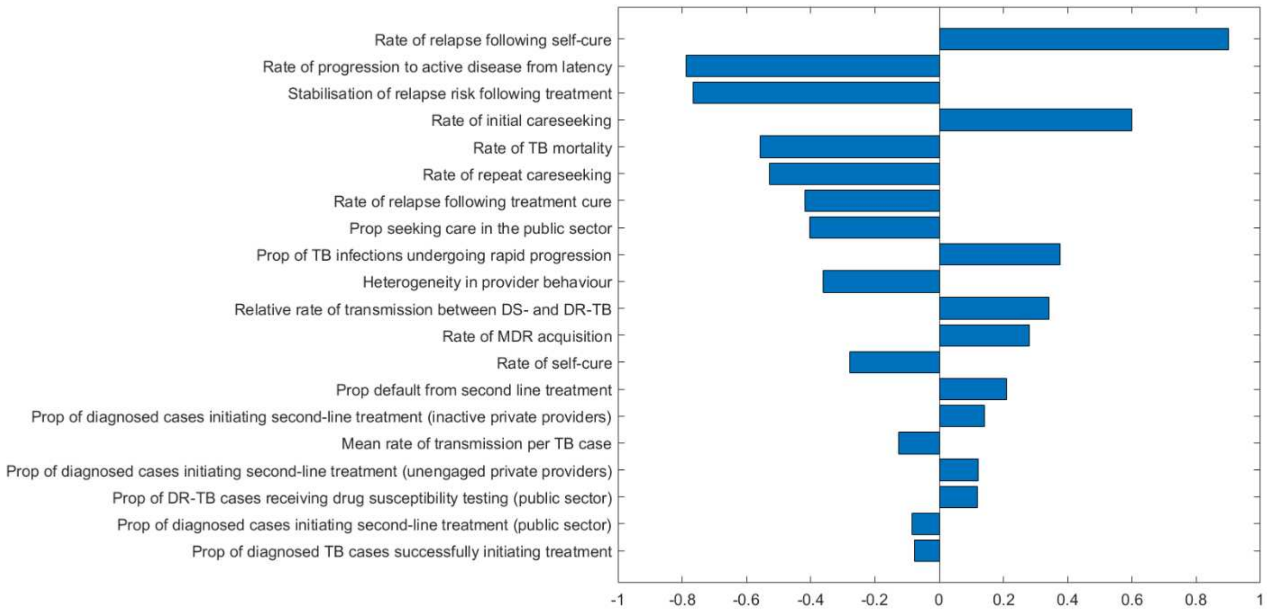

Figure S8. Multivariate sensitivity analysis of impact for Delhi. We used partial rank correlation coefficient (PRCC) to examine which parameter listed in tables 2-5 the output cases averted is most sensitive towards. Larger bars represent more sensitive parameters. Shown are the 20 most influential model parameters, in decreasing order of sensitivity from top to bottom.

3. Supporting tables

City	Number of identified private providers	Number of engaged private providers	Number of active private providers
Ahmedabad	1899	952	762
Delhi	2780	2016	894

Table S10. Number of identified, engaged, and active private providers in 2019 in Ahmedabad and Delhi. We assume that all private providers have been identified.

City	Heterogeneity index (α)
Ahmedabad	0.39 [0.32-0.46]
Delhi	0.32 [0.25-0.41]

Table S11. Heterogeneity index of private providers across Ahmedabad and Delhi. A heterogeneity index of 1 suggests that each provider sees the same number of patients (represented by the red line in fig S1). A smaller heterogeneity index indicates that a smaller number of providers are responsible for a larger number of patients. To calculate the heterogeneity, we divided the logarithm of the proportion of notifications by the logarithm of the proportion of providers responsible for those notifications.

Cost item	Total cost in 2019 (USD)	
	Ahmedabad	Delhi
Field officer salary	31,433	55,936
Hub agent salary	113,808	281,373
SCT agent salary	2055	5287
Treatment coordinator salary	8569	13,445
CMEs	460	6095
Promotional material	10	10
Sputum sample logistics	2020	5090
CB-NAAT tests	83,886	98,088
FDCs**	N/A	

Table S12. Total costs of PPSA activities in Ahmedabad and Delhi in 2019. We assume a conversion rate of USD 1 = 73 INR. *We assume a fix cost per FDC of 45 USD.

Category	Description
Provider activation	Costs associated with the engagement of inactive providers to encourage them to become active and start notifying their TB cases
Provider retention	Costs associated with the engagement of active providers to encourage them to remain active and keep notifying their TB cases
Diagnostic engagement	Costs associated with encouraging active providers to start using CB-NAAT, to increase their use of CB-NAAT and maintain their use of CB-NAAT
Diagnostic logistics	All other costs associated with CB-NAAT testing that are not to do with engagement. Includes cost of transporting sputum samples, cost of staff and cost of the CB-NAAT tests.
Treatment engagement	Costs associated with encouraging active providers to start using, increase or maintain their use of government supplied FDCs and adherence support.
Treatment logistics	Cost of government supplied FDCs and adherence support

Table S13. Costing categories. We categorise each cost item into one of the categories listed in this table to help identify the cost items that will increase as the coverage of the different interventions increase.

PPSA activity	Effort needed
Provider activation	5 (4-7) visits needed to onboard an inactive provider
Increasing CB-NAAT usage amongst occasional users	One visit increases a provider's CB-NAAT use by 0.2%. Assumed the same across all cities.
Converting non-CB-NAAT users to occasionally use CB-NAAT	7 (5-8) visits needed to convert a non-CB-NAAT user. Assumed the same across all cities
Converting non-FDC users to use FDC	6 (4-7) visits needed to convert a non-FDC user. Assumed the same across all cities.

Table S14. Engagement effort needed for each PPSA service provision in Ahmedabad and Delhi in 2019. As described in the text, values shown in these tables are essentially expert opinion, elicited through in-dept discussion with implementing teams from CHAI.

Ahmedabad						
**Cost item**	**Cost category**					
	Provider activation	Provider retention	Diagnostic engagement	Diagnostic logistics	Treatment engagement	Treatment logistics
Field officer	10%	42%	24%	-	24%	-
Hub agent	-	51%	-	24%	-	25%
SCT agent	-	-	-	100%	-	-
Treatment coordinator	-	-	-	-	-	100%
CMEs	-	40%	30%	-	30%	-
Promotional material	-	52%	29%	-	19%	-
Sputum sample logistics	-	-	-	100%	-	-
CB-NAAT	-	-	-	100%	-	-
FDCs	-	-	-	-	-	100%
**Delhi**						
**Cost item**	**Cost category**					
	Provider activation	Provider retention	Diagnostic engagement	Diagnostic logistics	Treatment engagement	Treatment logistics
Field officer	13%	28%	32%	-	27%	-
Hub agent	-	74%	-	18%	-	8%
SCT agent	-	-	-	100%	-	-
Treatment coordinator	-	-	-	-	-	100%
CMEs	-	40%	30%	-	30%	-
Promotional material	-	52%	29%	-	19%	-
Sputum sample logistics	-	-	-	100%	-	-
CB-NAAT	-	-	-	100%	-	-
FDCs	-	-	-	-	-	100%

Table S15. Costing split across PPSA activities for Ahmedabad and Delhi in 2019. The field officer split was determined by the percentage of calls that were made by a field officer for provider activation, provider retention, diagnostic engagement and treatment engagement; similarly, the hub agent split was determined by the proportion of time hub agents estimated they had spent on provider retention, diagnostic logistics and treatment logistics; the CMEs and promotional materials splits were determined by the amount of engagement that was aimed at provider retention, diagnostic engagement and treatment engagement, which was assumed constant across all cities. Each row adds up to 100%.

Cost item	Total cost	Category split	Unit description	No. of units	Cost per unit (USD)
Field officer	31,433	10%	No. of visits in 2019 made to onboard an inactive provider	1548	2.08
**PPSA activity: Provider retention**					
Field officer	31,433	42%	No. of active providers in 2019	762	17.77
Hub agent	113,808	51%	No. of active providers in 2019	762	78.12
CMEs	460	40%	No. of providers that attended CMEs in 2019	51	3.70
Promotional material	10	52%	No. of active providers in 2019	762	0.007
**PPSA activity: Diagnostic engagement**					
Field officer	31,433	24%	No. of visits in 2019 made to active providers to increase CB-NAAT usage	3572	2.17
			No. of active providers in 2019	762	10.15
CMEs	460	30%	No. of providers that attended CMEs in 2019	51	2.78
Promotional material	10	29%	No. of active providers in 2019	762	0.004
**PPSA activity: Diagnostic logistics**					
Hub agent	113,808	24%	No. of CB-NAAT tests performed in 2019	3615	7.75
SCT agent	2055	100%	No. of CB-NAAT tests performed in 2019	3615	0.58
Sputum sample logistics	2020	100%	No. of CB-NAAT tests performed in 2019	3615	0.57
CB-NAAT tests	83,886	100%	No. of CB-NAAT tests performed in 2019	3615	23.80
**PPSA activity: Treatment engagement**					
Field officer	31,433	24%	No. of visits in 2019 made to active providers to use FDCs	3619	2.14
			No. of active providers in 2019	762	10.15
CMEs	460	30%	No. of providers that attended CMEs in 2019	51	2.78
Promotional material	10	19%	No. of active providers in 2019	762	0.003
**PPSA activity: Treatment logistics**					
Treatment coordinator	8569	100%	No. of patient treatment months in 2019	57,868	0.15
Hub agent	113,808	25%	No. of FDC initiations in 2019	3445	8.47
FDCs	-	-	-	-	45

Table S16. Unit costs for Ahmedabad

PPSA activity: Provider activation					
**Cost item**	**Total cost**	**Category split**	**Unit description**	**No. of units**	**Cost per unit (USD)**
Field officer	55,936	13%	No. of visits in 2019 made to onboard an inactive provider	2984	2.50
**PPSA activity: Provider retention**					
Field officer	55,936	28%	No. of active providers in 2019	894	17.97
Hub agent	281,373	74%	No. of active providers in 2019	894	238.88
CMEs	6095	40%	No. of providers that attended CMEs in 2019	1469	1.70
Promotional material	10	52%	No. of active providers in 2019	894	0.006
**PPSA activity: Diagnostic engagement**					
Field officer	55,936	32%	No. of visits in 2019 made to active providers to increase CB-NAAT usage	7219	2.54
			No. of active providers in 2019	894	20.54
CMEs	6095	30%	No. of providers that attended CMEs in 2019	1469	1.28
Promotional material	10	29%	No. of active providers in 2019	894	0.003
**PPSA activity: Diagnostic logistics**					
Hub agent	281,373	18%	No. of CB-NAAT tests performed in 2019	4227	12.29
SCT agent	5287	100%	No. of CB-NAAT tests performed in 2019	4227	1.28
Sputum sample logistics	5090	100%	No. of CB-NAAT tests performed in 2019	4227	1.23
CB-NAAT tests	98,088	100%	No. of CB-NAAT tests performed in 2019	4227	23.80
**PPSA activity: Treatment engagement**					
Field officer	55,936	27%	No. of visits in 2019 made to active providers to use FDCs	5863	2.64
			No. of active providers in 2019	894	17.33
CMEs	6095	30%	No. of providers that attended CMEs in 2019	1469	1.28
Promotional material	10	19%	No. of active providers in 2019	894	0.002
**PPSA activity: Treatment logistics**					
Treatment coordinator	13,445	100%	No. of patient treatment months in 2019	108,915	0.13
Hub agent	281,373	8%	No. of FDC initiations in 2019	1480	13.65
FDCs	-	-	-	-	45

Table S17. Unit costs for Delhi.

Intervention	Incremental cost (2020-2035, USD)	Percentage of cumulative TB cases averted (2020-2035)	Cost-efficiency
**Individual interventions**			
Increasing the number of active providers (assuming newly active providers adopt average behaviour)	2,269,300 (1,477,000 – 3,723,000)	3.00% (2.59-3.37)	0.0032 (0.0019- 0.0050)
Increasing the number of active providers (assuming newly active providers' behaviour is 25% worse than average)	2,224,400 (1,435,200 – 3,672,300)	2.98% (2.58-3.35)	0.0032 (0.0019- 0.0051)
Increasing the number of active providers (assuming newly active providers' behaviour is 50% worse than average)	2,120,000 (1,339,000 – 3,555,300)	2.89% (2.48-3.24)	0.0033 (0.0019- 0.0053)
Increasing CB-NAAT use amongst occasional users	9,005,000 (6,801,000 – 13,190,000)	2.83% (2.42-3.33)	0.0008 (0.0006- 0.0010)
Increasing the number of occasional CB-NAAT users	2,211,700 (1,683,500 – 3,275,100)	2.32% (1.97-2.67)	0.0025 (0.0018- 0.0033)
Increasing the number of FDC users	1,787,700 (1,438,900 – 2,503,800)	2.53% (2.13-2.91)	0.0031 (0.0024- 0.0041)
**Combination of the two most cost-efficient interventions: Increasing the number of active providers* + …**			
+Increasing the number of occasional CB-NAAT users	5,020,300 (3,590,000 – 7,512,900)	3.21% (2.84-3.61)	0.0015 (0.0010- 0.0022)
+ Increasing CB-NAAT use amongst occasional users	13,427,000 (10,019,000 – 19,839,000)	3.81% (3.40-4.45)	0.0007 (0.0005- 0.0009)
+ Increasing the number of FDC users	5,217,100 (3,791,600 – 8,182,300)	3.46% (2.96-3.91)	0.0016 (0.0010- 0.0022)
**Combination of the three most cost-efficient interventions: Increasing the number of active providers* + the number of FDC-users + …**			
+Increasing the number of occasional CB-NAAT users	7,163,000 (5,671,000 – 10,478,000)	3.67% (3.18-4.16)	0.0012 (0.0008- 0.0017)
+ Increasing CB-NAAT use amongst occasional users	15,467,000 (11,921,000 – 22,411,000)	4.27% (3.81-4.87)	0.0007 (0.0005- 0.0009)
**Combination of all interventions***			
+ Increasing CB-NAAT use amongst occasional users	27,976,000 (21,331,000 – 40,913,000)	5.17% (4.49-6.17)	N/A

Table S18. Cost efficiency of service provisions in Ahmedabad. We also conducted a sensitivity analysis on the behaviour of newly active providers, and whether increasing the number of active providers remains the most cost-efficient individual intervention if the behaviour of newly active providers is assumed to be 25% and 50% worse than pre-existing active providers. Indeed, even if the behaviour of newly active providers is 50% worse, this intervention is still the most cost-efficient individual intervention when scaled up to its feasible maximum. *We assume that these newly active providers adopt average behaviour.

Intervention	Incremental cost (2020-2035, USD)	Percentage of cumulative TB cases averted (2020-2035)	Cost-efficiency
**Individual interventions**			
Increasing the number of active providers (assuming newly active providers adopt average behaviour)	20,760,000 (12,478,000 – 36,094,000)	3.06% (2.43-3.98)	0.0003 (0.0002- 0.0006)
Increasing CB-NAAT use amongst occasional users	22,249,000 (13,546,000 – 30,724,000)	1.11% (0.97-1.27)	0.0001 (0.0001- 0.0002)
Increasing the number of occasional CB-NAAT users	4,920,900 (2,985,500 – 6,819,600)	0.86% (0.73-1.03)	0.0004 (0.0003- 0.0006)
Increasing the number of FDC users	4,496,400 (3,283,400 – 5,866,200)	1.13% (0.97-1.34)	0.0006 (0.0004- 0.0007)
**Combination of the two most cost-efficient interventions: Increasing the number of FDC-users + …**			
+Increasing the number of active providers	30,981,000 (21,744,000-46,622,000)	3.80% (2.92-5.06)	0.00026 (0.00017- 0.0046)
+Increasing the number of occasional CB-NAAT users	9,250,000 (6,796,000 – 12,272,000)	1.21% (1.05-1.41)	0.00028 (0.00021-0.00038)
+ Increasing CB-NAAT use amongst occasional users	26,794,000 (17,716,000 – 36,310,000)	1.47% (1.29-1.72)	0.00012 (0.00008-0.00017)
**Combination of the three most cost-efficient interventions: Increasing the number of FDC users + increasing the number of occasional CB-NAAT users + …**			
+ Increasing the number of active providers	41,648,000 (30,441,000 - 60,488,000)	3.95% (3.05-5.22)	0.00020 (0.00013 – 0.00033)
+ Increasing CB-NAAT use amongst occasional users	60,238,000 (37,969,000 – 82,081,000)	1.96% (1.64-2.35)	0.00007 (0.00005 – 0.00010)
**Combination of all interventions**			
+ Increasing CB-NAAT use amongst occasional users	157,450,000 (100,600,000 – 213,200,000)	5.35% (4.30-7.00)	N/A

Table S19. Cost efficiency of service provisions in Delhi. *We assume that these newly active providers adopt average behaviour.

Cost category	Baseline	Increasing no. of active providers	Increasing CB-NAAT usage amongst occasional CB-NAAT users	Increasing the no. of occasional CB-NAAT users	Increasing the no. of providers offering FDCs
**Provider retention**	1.2 million	1.5 million	1.2 million	1.2 million	1.2 million
**Onboarding engagement**	N/A	2,000	N/A	N/A	N/A
**CB-NAAT engagement**	12,000	15,000	0.3 million	14,000	12,000
**FDC engagement**	11,000	14,000	11,000	11,000	17,000
**CB-NAAT logistics**	6.0 million (4.6 – 8.9 million)	7.6 million (5.7 – 11.0 million)	15.0 million (12.2 – 23.8 million)	8.5 million (6.4 – 12.7 million)	6.0 million (4.7 – 8.9 million)
**Adherence support**	0.1 million (0.1 – 0.2 million)	0.2 million (0.1 – 0.3 million)	0.1 million (0.1 – 0.2 million)	0.1 million (0.1 – 0.2 million)	0.1 million (0.1 – 0.2 million)
**FDCs**	5.5 million (3.2 – 9.0 million)	6.7 million (4.1 – 11.0 million)	4.3 million (2.7 – 7.0 million)	5.2 million (3.1 – 8.4 million)	7.4 million (4.9 – 11.6 million)
**Public sector diagnoses**	35.0 million (26.6 – 50.4 million)	35.0 million (25.2 – 50.4 million)	35.0 million (25.2 – 50.4 million)	35.0 million (26.6 – 50.4 million)	35.0 million (26.6 – 50.4 million)
**Public sector treatment**	68.6 million (53.2– 93.8 million)	68.6 million (53.2– 92.4 million)	68.6 million (53.2– 93.8 million)	68.6 million (53.2– 93.8 million)	68.6 million (53.2– 93.8 million)

Table S20. Breakdown of total costs across each intervention between 2020 and 2035 for Ahmedabad.

Cost category	Baseline	Increasing no. of active providers	Increasing CB-NAAT usage amongst occasional CB-NAAT users	Increasing the no. of occasional CB-NAAT users	Increasing the no. of providers offering FDCs
**Provider retention**	3.5 million	8.0 million	3.5 million	3.5 million	3.5 million
**Onboarding engagement**	N/A	14,000	N/A	N/A	N/A
**CB-NAAT engagement**	22,000	50,000	0.3 million	32,000	22,000
**FDC engagement**	18,000	41,000	18,000	18,000	32,000
**CB-NAAT logistics**	8.1 million (4.9 – 11.0 million)	18.0 million (11.2 – 25.2 million)	32.2 million (19.6 – 44.8 million)	13.5 million (8.3 – 18.2 million)	8.1 million (4.9 – 11.0 million)
**Adherence support**	0.3 million (0.2 – 0.4 million)	0.6 million (0.3 – 0.9 million)	0.2 million (0.1 – 0.4 million)	0.3 million (0.2 – 0.4 million)	0.3 million (0.2 – 0.4 million)
**FDCs**	10.2 million (5.3 – 19.6 million)	23.8 million (11.9 – 44.8 million)	7.7 million (4.2 – 15.4 million)	9.7 million (5.0 – 19.6 million)	15.4 million (9.8 – 25.2 million)
**Public sector diagnoses**	117.6 million (74.2 – 154 million)	116.2 million (72.8 – 154 million)	116.2 million (72.8 – 154 million)	117.6 million (74.2 – 154 million)	117.6 million (74.2 – 154 million)
**Public sector treatment**	224 million (168 million – 294 million)	224 million (168 million – 294 million)	224 million (168 million – 294 million)	224 million (168 million – 294 million)	224 million (168 million – 294 million)

Table S21. Breakdown of total costs across each intervention between 2020 and 2035 for Delhi.

Intervention	Maintenance costs sensitivity analysis	Incremental cost (2020-2035, USD)	Percentage of cumulative TB cases averted (2020-2035)	Cost-efficiency
Increasing the number of active providers	*-20%:*	1,963,400 (1,170,200-3,416,300)	3.00% (2.59-3.37)	0.0037 (0.0021-0.0063)
	+20%	2,575,300 (1,782,200-4,028,200)		0.0028 (0.0017-0.0041)
Increasing the number of occasional CB-NAAT users	*-20%:*	1,965,900 (1,440,400-3,030,200)	2.32% (1.97-2.67)	0.0028 (0.0020-0.0038)
	+20%	2,455,700 (1,930,200-3,520,000)		0.0023 (0.0017-0.0029)
Increasing CB-NAAT use amongst occasional users	*-20%:*	8,743,000 (6,671,000-13,031,000)	2.83% (2.42-3.33)	0.0008 (0.0006-0.0010)
	+20%	9,232,000 (7,160,000-13,521,000)		0.0007 (0.0006-0.0009)
Increasing the number of FDC users	*-20%:*	1,541,800 (1,194,000-2,260,900)	2.53% (2.13-2.91)	0.0039 (0.0026-0.0050)
	+20%	2,031,600 (1,683,800-2,750,700)		0.0027 (0.0022-0.0036)

Table S22. Sensitivity analysis on the cost of maintenance in Ahmedabad. We explored whether increasing or decreasing maintenance cost by 20% effected the cost-efficiency order of individual service provisions. Maintenance costs include the cost of provider retention, the cost of CB-NAAT engagement to maintain CB-NAAT use, and the cost of FDC engagement to maintain FDC use.

Intervention	Maintenance costs sensitivity analysis	Incremental cost (2020-2035, USD)	Percentage of cumulative TB cases averted (2020-2035)	Cost-efficiency
Increasing the number of active providers	*-20%:*	19,145,000 (10,865,000 – 34,481,000)	3.06% (2.43-3.98)	0.0003 (0.0002-0.0007)
	+20%	22,375,000 (14,095,000 – 37,712,000)		0.0003 (0.0002 – 0.0006)
Increasing the number of occasional CB-NAAT users	*-20%:*	4,205,900 (2,272,200 – 6,104,300)	0.86% (0.73-1.03)	0.0004 (0.0003-0.0008)
	+20%	5,638,400 (3,704,700 – 7,536,800)		0.0003 (0.0002-0.0005)
Increasing CB-NAAT use amongst occasional users	*-20%:*	21,455,000 (12,823,000 – 29,889,000)	1.11% (0.97-1.27)	0.0001 (0.0001-0.0002)
	+20%	22,887,000 (14,255,000–31,321,000)		0.0001 (0.0001-0.0002)
Increasing the number of FDC users	*-20%:*	3,782,600 (2,569,100 – 5,154,300)	1.13% (0.97-1.34)	0.0007 (0.0005-0.0009)
	+20%	5,215,100 (4,001,600 – 6,586,800)		0.0005 (0.0004-0.0006)

Table S23. Sensitivity analysis on the cost of maintenance in Delhi. We explored whether increasing or decreasing maintenance cost by 20% effected the cost-efficiency order of individual service provisions. Maintenance costs include the cost of provider retention, the cost of CB-NAAT engagement to maintain CB-NAAT use, and the cost of FDC engagement to maintain FDC use.

4. Bibliography

1. Mistry N, Lobo E, Shah S, Rangan S, Dholakia Y. Pulmonary Tuberculosis in Patna, India: Durations, Delays, and Health Care Seeking Behaviour Among Patients Identified Through Household Surveys. *Journal of Epidemiology and Global Health*. 2017;7(4): 241–248.

2. Veesa KS, John KR, Moonan PK, Kaliappan SP, Manjunath K, Sagili K, et al. Diagnostic Pathways and Direct Medical Costs Incurred by New Adult Pulmonary Tuberculosis Patients Prior to Anti-Tuberculosis Treatment - Tamil Nadu, India. *PLOS ONE*. 2018;13(2): e0191591.

3. Pandey A, Ploubidis GB, Clarke L, Dandona L. Hospitalisation Trends in India From Serial Cross-Sectional Nationwide Surveys: 1995 to 2014. *BMJ Open*. 2017;7(12): e014188.

4. Sachdeva KS, Satyanarayana S, Dewan P, Nair SA, Reddy R, Kundu D, et al. Source of Previous Treatment for Re-Treatment TB Cases Registered under the National TB Control Programme, India, 2010. *PLOS ONE*. 2011;6(7): e22061.

5. Satyanarayana S, Nair SA, Chadha SS, Shivashankar R, Sharma G, Yadav S, et al. From Where Are Tuberculosis Patients Accessing Treatment in India? Results From a Cross-Sectional Community Based Survey of 30 Districts. *PLOS ONE*. 2011;6(9): e24160.

6. Sharma G, Sinha A, Bastian S, Santha T. Knowledge and behaviour of chest symptomatics in urban slum populations of two states in India towards care-seeking. *The Indian Journal of Tuberculosis*. 2013;60(2): 95–106.

7. Satyanarayana S, Subbaraman R, Shete P, Gore G, Das J, Cattamanchi A, et al. Quality of Tuberculosis Care in India: A Systematic Review. *International Journal of Tuberculosis and Lung Disease*. 2015;19(7): 751–763.

8. World Health Organisation. *A brief history of tuberculosis control in India.* 2010. https://apps.who.int/iris/bitstream/handle/10665/44408/9789241500159_eng.pdf?sequence=1

9. Menzies D, Benedetti A, Paydar A, Martin I, Royce S, Pai M, et al. Effect of Duration and Intermittency of Rifampin on Tuberculosis Treatment Outcomes: A Systematic Review and Meta-Analysis. *PLOS Med*. 2009;6(9): e1000146. https://doi.org/10.1371/journal.pmed.1000146.

10. Vynnycky E, Fine PE. The natural history of tuberculosis: the implications of age-dependent risks of disease and the role of reinfection. *Epidemiology and Infection*. 1997;119(2): 183–201.

11. Dye C, Garnett GP, Sleeman K, Williams BG. Prospects for worldwide tuberculosis control under the WHO DOTS strategy. Directly observed short-course therapy. *Lancet (London, England)*. 1998;352(9144): 1886–1891.

12. Horsburgh CR, O'Donnell M, Chamblee S, Moreland JL, Johnson J, Marsh BJ, et al. Revisiting rates of reactivation tuberculosis: a population-based approach. *American Journal of Respiratory and Critical Care Medicine*. 2010;182(3): 420–425. https://doi.org/10.1164/rccm.200909-1355OC.

13. Thomas A, Gopi PG, Santha T, Chandrasekaran V, Subramani R, Selvakumar N, et al. Predictors of relapse among pulmonary tuberculosis patients treated in a DOTS programme in South India. *International Journal of Tuberculosis and Lung Disease*. 2005;9(5): 556–561.

14. Tiemersma EW, van der Werf MJ, Borgdorff MW, Williams BG, Nagelkerke NJD. Natural history of tuberculosis: duration and fatality of untreated pulmonary tuberculosis in HIV negative patients: a systematic review. *PloS One*. 2011;6(4): e17601. https://doi.org/10.1371/journal.pone.0017601.

15. Andrews JR, Noubary F, Walensky RP, Cerda R, Losina E, Horsburgh CR. Risk of progression to active tuberculosis following reinfection with Mycobacterium tuberculosis. *Clinical Infectious Diseases*. 2012;54(6): 784–791.

16. World Bank. *Data: India*. 2015 [Accessed 12th May 2016]. http://data.worldbank.org/country/india [Accessed 12th May 2016].

17. Pandey S, Chadha VK, Laxminarayan R, Arinaminpathy N. Estimating tuberculosis incidence from primary survey data: a mathematical modeling approach. *The International Journal of Tuberculosis and Lung Disease*. 2017;21(4): 366–374. https://doi.org/10.5588/ijtld.16.0182.

18. Sachdeva KS, Satyanarayana S, Dewan P, Nair SA, Reddy R, Kundu D, et al. Source of Previous Treatment for Re-Treatment TB Cases Registered under the National TB Control Programme, India, 2010. *PLOS ONE*. 2011;6(7): e22061.

19. Satyanarayana S, Nair SA, Chadha SS, Shivashankar R, Sharma G, Yadav S, et al. From Where Are Tuberculosis Patients Accessing Treatment in India? Results from a Cross-Sectional Community Based Survey of 30 Districts. *PLOS ONE*. 2011;6(9): e24160. https://doi.org/10.1371/journal.pone.0024160.

20. Subbaraman R, Nathavitharana RR, Satyanarayana S, Pai M, Thomas BE, Chadha VK, et al. The Tuberculosis Cascade of Care in India's Public Sector: A Systematic Review and Meta-analysis. *PLOS Medicine*. 2016;13(10): e1002149. https://doi.org/10.1371/journal.pmed.1002149.

21. Van't Hoog A, Langendam MW, Mitchell E, Cobelens FG, Sinclair D, Leeflang MMG, et al. *A sytematic review of the sensitivity and specificity of symptom- and chest-radiography screening for active pulmonary tuberculosis in HIV-negative persons and persons with unknown HIV status*. 2013. https://www.who.int/tb/Review2Accuracyofscreeningtests.pdf

22. Steingart KR, Horne DJ, Pai M, Boehme CC, Dendukuri N. Xpert MTB/RIF assay for pulmonary tuberculosis and rifampicin resistance in adults (Review). *Cochrane Database of Systematic Reviews*. 2014;(1).

23. World Health Organisation. *Global Tuberculosis Report 2020*. 2020. https://www.who.int/publications/i/item/9789240013131

24. Subbaraman R, Nathavitharana RR, Mayer KH, Satyanarayana S, Chadha VK, Arinaminpathy N, et al. Constructing care cascades for active tuberculosis: A strategy for program monitoring and identifying gaps in quality of care. *PLOS Medicine*. 2019;16(2): e1002754.

25. Menzies NA, Gomez GB, Bozzani F, Chatterjee S, Foster N, Baena IG, et al. Cost-effectiveness and resource implications of aggressive action on tuberculosis in China, India, and South Africa: a combined analysis of nine models. *The Lancet Global Health*. 2016;4(11): e816–e826. https://doi.org/10.1016/S2214-109X(16)30265-0.

26. Houben RMGJ, Lalli M, Kranzer K, Menzies NA, Schumacher SG, Dowdy DW. What if they don't have tuberculosis? The consequences and trade-offs involved in false-positive diagnoses of tuberculosis. *Clinical Infectious Diseases*. 2019;68(1): 150–156. https://doi.org/10.1093/cid/ciy544.

27. Fochsen G, Deshpande K, Diwan V, Mishra A, Diwan VK, Thorson A. Health care seeking among individuals with cough and tuberculosis: a population-based study from rural India. *International Journal of Tuberculosis and Lung Disease*. 2006;10(9): 995–1000.

28. Gopi PG, Subramani R, Narayanan PR. Evaluation of different types of chest symptoms for diagnosing pulmonary tuberculosis cases in community surveys. *Indian Journal of Tuberculosis*. 2008;55(3): 116–121.

29. Rao VG, Bhat J, Yadav R, Gopalan GP, Nagamiah S, Bhondeley MK, et al. Prevalence of pulmonary tuberculosis - a baseline survey in central India. *PLOS One*. 2012;7(8): e43225. https://doi.org/10.1371/journal.pone.0043225.

30. Sachdeva KS, Raizada N, Sreenivas A, Van't Hoog A, van den Hof S, Dewan PK, et al. Use of Xpert MTB/RIF in decentralized public health settings and its effect on pulmonary TB and DR-TB case findings in India. *PLOS ONE*. 2015;10(5): e0126065. https://doi.org/10.1371/journal.pone.0126065.

31. Haario H, Saksman E, Tamminen J. An adaptive metropolis algorithm. *Bernoulli*. 2001;7(2): 223–242.
