# Supplementary material for: Operational priorities for engaging with India’s private healthcare sector for the control of tuberculosis: a modelling study
Source: BMJ Open. 2024 Mar 19;14(3):e069304. doi: 10.1136/bmjopen-2022-069304 (PMC10952976; doi:10.1136/bmjopen-2022-069304)
Supplement: Author's manuscript [file bmjopen-2022-069304.draft_revisions.pdf]

BMJ Open

BMJ Open is committed to open peer review. As part of this commitment we make the peer review history of every article we publish publicly available.

When an article is published we post the peer reviewers' comments and the authors' responses online. We also post the versions of the paper that were used during peer review. These are the versions that the peer review comments apply to.

The versions of the paper that follow are the versions that were submitted during the peer review process. They are not the versions of record or the final published versions. They should not be cited or distributed as the published version of this manuscript.

BMJ Open is an open access journal and the full, final, typeset and author-corrected version of record of the manuscript is available on our site with no access controls, subscription charges or pay-per-view fees (http://bmjopen.bmj.com).

If you have any questions on BMJ Open's open peer review process please email info.bmjopen@bmj.com

BMJ Open

Operational priorities for engaging with India's private healthcare sector for the control of tuberculosis: a modelling study

Journal:	*BMJ Open*
Manuscript ID	bmjopen-2022-069304
Article Type:	Original research
Date Submitted by the Author:	29-Oct-2022

Complete List of Authors:	Ricks, Saskia ; Imperial College London,<br>Singh, Ananya; Clinton Health Access Initiative<br>Sodhi, Ridhima; Clinton Health Access Initiative<br>Pal, Arnab; Clinton Health Access Initiative<br>Arinaminpathy, Nimalan; Imperial College London School of Public Health

Keywords:	Tuberculosis < INFECTIOUS DISEASES, Epidemiology < INFECTIOUS DISEASES, HEALTH ECONOMICS

SCHOLARONE™
Manuscripts

I, the Submitting Author has the right to grant and does grant on behalf of all authors of the Work (as defined in the below author licence), an exclusive licence and/or a non-exclusive licence for contributions from authors who are: i) UK Crown employees; ii) where BMJ has agreed a CC-BY licence shall apply, and/or iii) in accordance with the terms applicable for US Federal Government officers or employees acting as part of their official duties; on a worldwide, perpetual, irrevocable, royalty-free basis to BMJ Publishing Group Ltd ("BMJ") its licensees and where the relevant Journal is co-owned by BMJ to the co-owners of the Journal, to publish the Work in this journal and any other BMJ products and to exploit all rights, as set out in our licence.

The Submitting Author accepts and understands that any supply made under these terms is made by BMJ to the Submitting Author unless you are acting as an employee on behalf of your employer or a postgraduate student of an affiliated institution which is paying any applicable article publishing charge ("APC") for Open Access articles. Where the Submitting Author wishes to make the Work available on an Open Access basis (and intends to pay the relevant APC), the terms of reuse of such Open Access shall be governed by a Creative Commons licence – details of these licences and which Creative Commons licence will apply to this Work are set out in our licence referred to above.

Other than as permitted in any relevant BMJ Author's Self Archiving Policies, I confirm this Work has not been accepted for publication elsewhere, is not being considered for publication elsewhere and does not duplicate material already published. I confirm all authors consent to publication of this Work and authorise the granting of this licence.

For peer review only

Operational priorities for engaging with India's private healthcare sector for the control of tuberculosis: a modelling study

Saskia Ricks*[1], Ananya Singh[2], Ridhima Sodhi[2], Arnab Pal[2] and Nimalan Arinaminpathy[1]

*Corresponding author; saskia.ricks12@imperial.ac.uk

[1]Imperial College London, Praed Street, London, W2 1NY, UK

[2]Clinton Health Access Initiative, New Delhi, India

For peer review only

Key words: tuberculosis, epidemiology, health economics

Word count: 6123

Abstract

Objectives: The Joint Effort for Elimination of Tuberculosis ("JEET") is the largest private sector engagement initiative for tuberculosis (TB) in India. Considering the potential impact of such an intervention, it is important to anticipate how the different services offered by JEET should be expanded in the future.

Design: We developed a mathematical model of TB transmission dynamics, coupled with a cost model.

Setting: Ahmedabad and New Delhi, two cities with contrasting levels of JEET coverage.

Participants: Estimated patients with TB in Ahmedabad and New Delhi.

Interventions: We investigated the epidemiological impact of expanding three different PPSA services: provider recruitment, uptake of Xpert and uptake of adherence support mechanisms (specifically government supplied fixed-dose combination drugs), all compared to a continuation of current TB services.

Results: Our results suggest that in Delhi, increasing the use of adherence support mechanisms amongst private providers should be prioritised, having the lowest incremental cost-per-case-averted between 2020 and 2035 of $170,000 (110,000 – 310,000). Likewise in Ahmedabad, increasing provider recruitment should be prioritised, having the lowest incremental cost-per-case averted of $18,000 (12,000-29,000) per TB case averted.

Conclusion: Results illustrate how intervention priorities may vary in different settings across India, depending on local conditions, and the existing degree of uptake of PPSA services. Modelling can be a useful tool for identifying these priorities for any given setting.

Strength and limitations

- We have used detailed programmatic data to inform costs, effectiveness, and real-world coverage of the different interventions discussed in this analysis to help identify operational priorities.
- However, due to a lack of city-specific data for Delhi and Ahmedabad, the mathematical model was instead calibrated to prevalence and infection surveys from Chennai, another large Indian city.
- Assumptions were also made to account for healthcare provider behaviour.

Introduction

For peer review only

For peer review only - http://bmjopen.bmj.com/site/about/guidelines.xhtml

India's vast, fragmented private healthcare sector manages the majority of TB patients in the country (1–3). Recent years have seen increasing recognition of the importance of engaging effectively with this sector (4–8): not only in notifying TB to public health authorities, but also in offering high-quality diagnostics and treatment adherence support for patients being managed by private providers. From 2013 onwards, pilots of 'public-private support agencies' (PPSAs) showed how private providers could be successfully engaged, through subsidising and providing incentives to private providers if they follow the standard of care for the diagnosis and treatment of TB, as well as providing training and adherence support (5,7).

To take the PPSA model to scale across the country, the Joint Effort for Elimination of Tuberculosis (JEET) was launched by 3 partner organisations (the Clinton Health Access Initiative; Centre for Health, Research and Innovation; and Foundation for Innovative New Diagnostics) in 2018. Supported by the Global Fund and coordinated by the Central TB Division of India, it is the largest private sector engagement initiative for TB in India to date. The main activities include increasing case notifications by engaging with private providers, improving the quality of TB diagnosis by providing free access to molecular diagnostics, and improving treatment outcomes by providing free fixed dose combination (FDCs) and adherence support. By, 2020 JEET was operating in 485 cities across India (9). Future operations in these cities face the question of which services to prioritise, for promoting uptake still further. Addressing this question can benefit from an understanding of the potential epidemiological impact of different strategies, along with projected costs and cost-effectiveness. Here, we aimed to meet this need using mathematical models of TB transmission. We considered two urban settings, Ahmedabad and Delhi, with contrasting levels of uptake of PPSA services.

Method

Model overview

We developed a deterministic, compartmental model of TB transmission amongst adults (>15 years old) consistent with an urban setting in India (fig 1). The model captures routine TB services in India, including the scale-up of the Revised National TB Control Programme (RNTCP) between 1997 and 2007 (10), and the scale-up of private sector engagement from 2017 onwards (11). We incorporated the acquisition and transmission of drug-resistant TB (DR-TB), including both rifampicin-resistant and multi-drug resistant TB. For simplicity, we ignored HIV status, and the distinction between pulmonary and extrapulmonary TB, although we conducted a sensitivity analysis on the latter. We separately simulated a non-TB symptomatic population (fig 1) to count the number of TB diagnoses and false positive treatments amongst those with symptoms suggestive of TB, but without TB infection. We calibrated the size of the non-TB symptomatic population to give a 10% prevalence of TB amongst those presenting for care, in either the public or private sector (12–14). The supplementary information provides further details on the model, including governing equations

For peer review only

(tables S1-S3), and a breakdown of the proportion of patients visiting different providers for diagnosis and treatment (tables S4-S5, repesctively).

Data sources

To reflect a typical urban setting in India (such as Ahmedabad and Delhi), we used survey data for the annual risk of TB infection (ARTI) and the prevalence of TB (15,16). Table 1 summarises the data from these sources. Tables 2-5 list parameter values used in the model (TB natural history, diagnosis, treatment and costs, respectively).

Indicator	Value	Source
Annual risk of TB infection	2% [1-3]	(15)
TB prevalence	259 per 100,000 [217-299]	(16)
TB mortality	32 per 100,000 [30-34]	(24)
Percent of TB cases that are MDR	4% [3-5]	(24)

Table 1. Calibration targets used to estimate model parameters. We assumed the same prevalence, annual risk of TB infection, TB mortality and percent of TB cases that are MDR across Ahmedabad and Delhi.

Parameter	Symbol	Value		Source
**TB natural history**				
Mean rate of transmission per TB case	β	10.42 yr^{-1} [7.28-17.24]		Model estimate
Relative rate of transmission between DS- and DR-TB	d	0.61 yr^{-1} [0.49-0.79]		Model estimate
Rate of MDR acquisition	m_a	Public sector/active providers that offer either adherence support of government supplied FDCs	0.01 [0.008-0.012]	(25)
	m_b	All private providers that are unengaged, inactive, or active that do not offer government supplied FDCs or adherence support	0.05 [0.04-0.06]	
Proportion of TB infections undergoing rapid progression	θ	0.11 [0.09-0.15]		(26,27)

For peer review only

Per capita hazard rate of progression to active disease from latency	ρ	0.001 yr^{-1} [0.0003-0.0024]		(27,28)
Per-capita hazard rate of relapse	r_1	Following self-cure/treatment default	0.14 yr^{-1} [0.10-0.18]	(25,29)
	r_2	Following treatment cure, >2 years	0.002 yr^{-1} [0.001-0.003]	
Stabilisation of relapse risk following treatment	ς	0.5 yr^{-1} [0.4-0.6]		(29): most relapse occurs in the first two years after treatment
Per-capita hazard rate of spontaneous cure	φ	0.17 yr^{-1} [0.13-0.21]		(30)
Per-capita hazard rate of TB mortality	μ_1	0.18 [0.13-0.25]		Model estimate
Proportion reduction in susceptibility to reinfection due to previous infection	π	0.21 [0.15-0.25]		(31)
**Demographics**				
Per-capita hazard rate of background mortality	μ_2	0.015 yr^{-1}		World Bank estimates: corresponds to an average lifespan of 69 years
Per-capita rate of population growth	σ	0.02 [0.01-0.025]		(36)

Table 2. List of model parameters: TB natural history

Parameter	Symbol	Value		Source
**TB services: diagnosis**				
Proportion of TB cases seeking care in the public sector	η	0.50 [0.40-0.60]		(1,32–34)
Proportion of private providers that are engaged	e	Ahmedabad	0.50	Data provided by CHAI
		Delhi	0.73	
Heterogeneity index of the behaviour of engaged private providers	$\propto$	Ahmedabad	0.39 [0.32-0.46]	Data provided by CHAI
		Delhi	0.32 [0.25-0.41]	
Proportion of engaged private providers that are active	a	Ahmedabad	0.80	Data provided by CHAI
		Delhi	0.44	
Per-capita hazard rate of initial careseeking for TB symptoms	δ_1	1.38 yr^{-1} [0.88-2.38]		Model estimate

For peer review only - http://bmjopen.bmj.com/site/about/guidelines.xhtml

Per-capita hazard rate of repeat careseeking for TB symptoms (following missed diagnosis)	δ_2	12 yr^{-1} [6-26]		Assumption: corresponds to range of 2 weeks to 2 months
Proportion of TB cases diagnosed correctly, per careseeking attempt in routine public sector TB services	ε_{sn}	0.84 [0.80-0.85]		(12,22)
Specificity of diagnosis in routine public sector TB services	ε_{sp}	0.98 [0.97-0.99]		(12)
Xpert test sensitivity	x_{sn}	0.89 [0.85-0.92]		(23)
Xpert test specificity	x_{sp}	0.99 [0.98-1.00]		(23)
Proportion of active private providers that send samples for confirmatory Xpert testing 100% of the time	p_{Xpert1}	Ahmedabad	0.09	Data provided by CHAI
		Delhi	0.04	
Proportion of active private providers that never send samples for confirmatory Xpert testing	p_{Xpert0}	Ahmedabad	0.55	Data provided by CHAI
		Delhi	0.66	
Proportion of samples that are sent off for confirmatory Xpert testing amongst active private providers that occasionally send samples off for confirmatory Xpert testing	x_p	Ahmedabad	0.23	Data provided by CHAI
		Delhi	0.15	
Clinical diagnosis sensitivity (including chest X-ray)	c_{sn}	0.75 [0.65-0.80]		(22)
Clinical diagnosis specificity (including chest x-ray)	c_{sp}	0.85 [0.80-0.95]		(22)
Proportion of DR-TB cases receiving drug susceptibility testing at point of TB diagnosis	$þ^{(pu)}$	Public sector	0.45 [0.40-0.50]	(24)
	$þ^{(pr0)}$	Private sector (providers not using Xpert)	0	

For peer review only - http://bmjopen.bmj.com/site/about/guidelines.xhtml

	$\text{Þ}^{(pr1)}$	Private sector (providers using Xpert)	1.00	

Table 3. List of model parameters: TB diagnosis

Parameter	Symbol	Value		Source
**TB services: treatment**				
Proportion of diagnosed TB cases initiating TB treatment	ω	0.87 [0.85-0.90]		(12). Assumed that patients from active providers are referred to the public sector to initiate second line treatment.
Amongst DR-TB cases failing first-line treatment, the proportion successfully transferred to second-line treatment, public sector	p_{SL}	0.87 [0.85-0.92]		Assumption
TB treatment initiation delay	ϕ	52 yr^{-1}		Assumed: corresponds to a mean treatment delay of 1 week in routine TB care
Proportion of patients that agree to adherence support	p_{adh1}	0.92		Data provided by CHAI
Proportion of active providers that agree to adherence support	p_{adh2}	Ahmedabad	0.93	Data provided by CHAI
		Delhi	0.93	
Proportion of active providers that agree to government supplied FDCs	p_{fdc}	Ahmedabad	0.39	Data provided by CHAI
		Delhi	0.07	
Per-capita hazard rate of treatment completion	τ	First line	2 yr^{-1}	Corresponds to a duration of 6 months
	τ_{mdr}	Second line	0.5 yr^{-1}	Corresponds to a duration of 2 years
Proportion of TB cases that default from treatment	χ_{Pu}	First line, public sector	0.15 [0.13-0.17]	(24,35), data provided by CHAI
	χ_{Pr_Z}	First line, private sector: unengaged/inactive/active but no adherence support	0.48 [0.45-0.55]	

For peer review only - http://bmjopen.bmj.com/site/about/guidelines.xhtml

		of government supplied FDCs offered		
	χ_{Pr_F}	First line, private sector: active (government supplied FDCs offered, no adherence support offered)	0.17 [0.16 -0.19]	
	χ_{Pr_A}	First line, private sector: active (no government supplied FDCs offered, adherence support offered)	0.21 [0.20-0.25]	
	χ_{Pr_AF}	First line, private sector: active (government supplied FDCs and adherence support offered)	0.15 [0.13-0.17]	
	χ_{mdr}	Second line	0.50 [0.45-0.60]	

Table 4. List of model parameters: TB treatment

Parameter	Symbol	Value	Source
**Costs**			
Cost per active TB disease diagnosis (passive case finding)	C_D	USD 18.30	(37)
Cost of first line treatment for active TB disease per person	C_T	USD 23.70 per treatment-month	(37)

Table 5. List of model parameters: TB costs

We incorporated uncertainty in parameter values and model inputs through Adaptive Bayesian Markov chain Monte Carlo (MCMC) (17). Thus, the uncertainty in model projections reflects the uncertainty in the model inputs; we refer to the uncertainty in model projections as the Bayesian credible intervals, using the 2.5th and 97.5th percentiles to reflect the lower and upper bound of an interval.

We also drew a range of operational data from CHAI, to inform elements such as existing uptake of JEET services in Ahmedabad and Delhi; provider behaviour over time in response to CHAI engagement efforts; and treatment outcomes under different types of adherence support in the private sector. Further details are provided below ('Modelling existing PPSA services').

Care cascade

For peer review only

For peer review only - http://bmjopen.bmj.com/site/about/guidelines.xhtml

We assumed that upon development of active disease, there is a delay (determined by calibration to the available data) before individuals seek care (8,18). We modelled public and private providers separately, assuming: (i) the quality of diagnosis to be lower in the latter than the former, and (ii) once individuals are diagnosed with TB and initiated on treatment, a lower rate of treatment completion in the private sector, due to the general lack of adherence support (19,20). Owing to a lack of systematic data for the private sector when not engaged with a PPSA, we allowed for a wide range of parameter values relating to both diagnosis and treatment in this sector (tables 3-4).

Modelling existing PPSA services

Provider recruitment

When commencing operations in any new setting, JEET conducts provider mapping to identify the highest-volume providers that should be prioritised. 'Engagement' with providers is achieved by field officers to encourage providers to notify their TB cases. However, even after these efforts, not all engaged providers notify TB, and require further engagement by field officers to do so, coupled with a strong enforcement of regulations. Accordingly, we distinguished different types of private providers: those who are not engaged; those who are engaged but not notifying TB ('inactive' providers); and those who are notifying TB ('active' providers) (table S6). We define 'active' providers as those notifying TB at least once a month. Active providers may, over time, turn inactive, for three main reasons: first, some providers may not have a sufficient number of TB patients every month and thus may notify infrequently depending on when they see a new TB patient; second, some providers, especially those that handle a smaller volume of TB patients, are seen less regularly by the field team and are therefore less likely to notify; and third, some providers may be dissatisfied with the services offered by JEET, and therefore intentionally stop notifying patients. If 'active' providers are defined as those notifying TB at least once a month, we accounted for this rate of attrition by weighting the amount of Xpert and FDC issued by the number of months providers are active for.

The transmission model follows the patient perspective of the careseeking journey; an important parameter relating to PPSA activities is the probability that a patient, when seeking care for their symptoms, visits a private provider amongst the three categories listed above. To estimate these probabilities from CHAI data for provider coverage, we modelled a 'heterogeneity index' α, such that:

$$\text{Proportion of patients visiting an engaged provider} = (\text{Proportion of providers engaged})^{\alpha}$$

See (fig S1, table S7). Thus, values of $\alpha < 1$ correspond with the situation where high-volume providers are preferentially engaged. We estimated the value of α using CHAI data from both Ahmedabad and Delhi.

TB diagnosis

For peer review only

Field officers engage with active providers to encourage them to offer Xpert testing to their presumptive TB patients. Specimen collection and transportation (SCT) agents collect the sputum samples from the provider clinic and transport them to laboratories where Xpert testing is performed for free. While efforts are made to encourage active providers to use these services, the services are not mandated under Project JEET, in the interest of a collaborative relationship.

There is a multimodal distribution for the uptake of this Xpert service amongst active private providers, including those who never use it; those who use it occasionally; and those for whom all notifications are Xpert confirmed (fig S2). In 2019, 55% and 66% of active providers in Ahmedabad and Delhi did not use Xpert confirmation when notifying TB. Providers who do not confirm a TB notification with Xpert instead evaluate patients using chest x-ray and clinical judgement (11,12,21). We assumed that inactive and unengaged private providers diagnose TB in the same way. We drew from the literature for the sensitivity and specificity of X-ray and clinical judgement in diagnosing TB ((22,23) (table 3).

Motivated by fig S2, we divided active providers into three categories depending on their use of Xpert: providers who always confirm TB notifications with Xpert, providers who occasionally use Xpert, and providers who never use Xpert. Table 6 shows the number of active providers in each of these categories for Ahmedabad and Delhi in 2019. Once active providers become an Xpert user, field officers continue visits to encourage these providers to continue confirming TB diagnoses with Xpert.

	**City**	
	Ahmedabad	*Delhi*
Total no. of active providers	762	894
No. of active providers that always use Xpert	65	35
No. of active providers that occasionally use Xpert	278	265
No. of active providers that never use Xpert	419	594
Percent of notifications that are Xpert confirmed by occasional users	22%	15%

Table 6. Xpert use amongst active providers in Ahmedabad and Delhi in 2019. To be classified as occasionally using Xpert, we assumed that providers use Xpert for at least 1% of notifications and less than 100% of notifications.

TB treatment

JEET offers two different adherence services for TB treatment via treatment coordinators: government supplied fixed-dose combinations (FDCs) and adherence support (table 7). Government supplied FDCs are supplied for free to the patient by the government, whereas patients on adherence support receive regular text messages and calls to ensure treatment adherence. Field

For peer review only - http://bmjopen.bmj.com/site/about/guidelines.xhtml

officers visit active providers regularly to ensure they are continuing to recommend government supplied FDCs and adherence support.

	City	
	Ahmedabad	*Delhi*
Total no. of active providers	762	894
No. of active providers that offer adherence support	712	835
Percent of patients that agree to adherence support	92%	92%
No. of active providers that agree to government supplied FDCs	529	242
Percent of active providers that consistently use FDCs, unweighted	45%	10%
Percent of active providers that consistently use FDCs, weighted	39%	7%

Table 7. Number of active providers that offer government supplied FDCs and adherence support in Ahmedabad and Delhi in 2019.

We considered 4 categories of active providers in the model: active providers that use government supplied FDCs and offer adherence support, those that use government supplied FDCs but do not offer adherence support, those that offer adherence support but do not use government supplied FDCs, and those that offer neither service. We drew from data provided by CHAI to inform treatment completion rates associated with each of these categories, active providers offering both services having the highest rate of treatment completion and those offering neither having the lowest rate (table 4). Finally, we assumed that inactive and unengaged providers do not offer adherence support and thus patients seeking treatment from these providers have the lowest treatment completion rates.

All patients diagnosed with DR-TB by a private provider are transferred to the public sector and initiated onto the second line treatment.

Interventions

We focused on the impact of the following four interventions:

- *Activate providers:* increasing the number of active providers amongst those already engaged but not notifying TB
- *Promote Xpert (occasional users)* – increasing Xpert use amongst occasional Xpert users

For peer review only

- *Promote Xpert (non-users)* – encouraging active providers that never use Xpert into occasionally using Xpert,
- *Promote adherence mechanisms* - increasing the use of government supplied FDCs amongst active providers.

We did not examine the impact of increasing adherence support, as current uptake is already high (above 90%) in Ahmedabad and Delhi.

We modelled a 'theoretical maximum coverage' scenario where all providers are amenable to intervention. In practice, however, some providers may remain uninterested in taking up the services offered despite sustained engagement efforts by field officers. To reflect these limits, we also modelled a 'feasible maximum' scenario where some proportion of providers remain unresponsive to the interventions listed above. Informed by expert consultation with CHAI, we assumed that: all inactive providers and occasional Xpert users are interested in notifying and increasing their Xpert use, whereas 40% of non-Xpert users are uninterested in using Xpert, and 35% of non-government supplied FDC users are uninterested in using government supplied FDCs (see fig S3 for more detail).

For both cities, we examined the epidemiological impact of increasing the four activities to their feasible and theoretical maxima, compared to an indefinite continuation of current levels of routine (publicly offered) TB and PPSA services. We modelled the scale-up of existing PPSA activities from 2017 to current levels in 2020. We assumed the interventions are scaled up linearly between 2020 and 2021, and simulated their impact until 2035.

Economic evaluation

We took the programmatic perspective, i.e., the costs that the national TB programme would have to pay. To examine which PPSA service should be prioritised in the future, we calculated the cost of expanding the different services to their feasible and theoretical maximum using operational cost data from 2019 for Ahmedabad and Delhi. Table S8 summarises the total cost in 2019 for the different activities involved in the service provisions offered by JEET. These activities include the salaries of the field officer, treatment coordinators, hub agents, and SCT agents; the cost of continuing medical education (CMEs; seminars organised for providers to increase engagement); the cost of promotional materials which includes the cost of designing, printing and dissemination; the cost of transporting sputum samples to the laboratories, (including the cost of procuring falcon tubes, carrier boxes, masks and hand sanitizer); the cost of Xpert tests, including test cartridges and manpower; and the cost of government supplied FDCs. We ignored sunk costs (office expenditure and state programme management costs) that have already been incurred in Ahmedabad and Delhi, and thus would not influence decisions for future interventions.

We categorised each cost item listed into table S8 into six different categories: provider activation, provider retention, diagnostic engagement, diagnostic logistics, treatment engagement and treatment logistics (described in further detail in table S9). Table S10 lists assumptions following

For peer review only - http://bmjopen.bmj.com/site/about/guidelines.xhtml

expert consultation with CHAI, for the level of effort needed to engage providers and achieve increased uptake (subject to uncertainty analysis). If a cost was associated with more than one category, the cost was subdivided across the relevant categories based on estimations from CHAI on the percentage of time spent on each category (table S11). Next, we calculated the per-unit cost of each cost item (table S12-13). For example, to calculate the cost of field officers to retain one provider per year, we multiplied the total cost of field officers in 2019, by the percentage of time spent by field officers on provider retention in 2019, divided by the number of active providers in 2019.

In practice, increasing the coverage of intervention activities is achieved through field officer visits, to engage directly with the providers. Finally, we also considered costs of routine TB services provided by the public sector, including the cost of diagnosis and the cost of treating DS- and DR-TB (table 5).

We calculated the incremental cost-effectiveness ratio (ICER) by dividing the incremental cost of increasing the coverage of PPSA services by the number of TB cases averted between 2020 and 2035, relative to a baseline of indefinite continuation of current levels of routine TB and JEET PPSA services.

Sensitivity analyses

We conducted various sensitivity analyses to explore the impact of certain model assumptions on model results. Firstly, we conducted multivariate sensitivity analysis using partial rank correlation coefficient to assess which model parameters are most influential for model outputs.

We also conducted sensitivity analyses on provider behaviour. In the main analysis, we accounted for the fact that active providers may revert to an inactive state, and we assumed that the behaviour of newly active providers is the same as pre-existing active behaviours. First, we considered an optimistic PPSA scale-up baseline that assumes active providers remain active once activated. Second, we assumed that newly active providers have 25% and 50% less uptake than pre-existing active providers (i.e., using Ahmedabad as an example, 9% of pre-existing active providers use Xpert 100% of the time; we modelled scenarios where this proportion is 4.5% and 2.3%). We did not differentiate extrapulmonary and pulmonary TB in the model; however, Xpert is unable to diagnose TB amongst patients with exclusively extrapulmonary disease. Thus, high levels of extrapulmonary TB can reduce the incremental benefit of Xpert testing. In Ahmedabad and Delhi, it is estimated that 32% and 38% of patients diagnosed by JEET PPSA services between 2019 and 2022 had extrapulmonary TB, respectively. To account for this in a simple way, we reduced the proportion of TB patients presenting to care that are correctly diagnosed by Xpert by 50%. For the purpose of sensitivity analysis, this reflects an extreme scenario where 50% of patients that present to care have extrapulmonary TB.

Finally, we examined the impact of varying maintenance costs by +/- 20% on the ICER of the different interventions. We defined maintenance costs as costs that are associated with maintaining

For peer review only - http://bmjopen.bmj.com/site/about/guidelines.xhtml

good engagement and uptake amongst active providers (i.e., maintaining notifications, FDC use and Xpert use).

Patient and public involvement

Patients or the public were not involved in the design, or conduct, or reporting, or dissemination plans of our research.

Results

Figs S4-S5 show results of model calibration, displaying the model fit against each of the calibration targets listed in table 1.

Figs 2 shows model projections for the impact on TB incidence between 2017 and 2035 in Ahmedabad and Delhi across varying levels of PPSA coverage, with cumulative impact summarised in table 8. Relative to a comparator where current TB services are maintained, increasing the four PPSA activities simultaneously to their feasible maximum coverage would avert 5.1% (95% CrI 4.4-6.2%) of cumulative TB cases between 2020 and 2035, whereas increasing them to their theoretical maximum would avert 7.4% (95% CrI 6.2-9.2%) of cumulative TB cases between 2020 and 2035 in Ahmedabad. In Delhi, 5.4% (95% CrI 4.4-6.9%) and 6.7% (95% CrI 5.3-8.9%) of cumulative TB cases would be averted under feasible and theoretical maximum coverages, respectively. There would be a larger impact on DR-TB cases and deaths averted as three of the four PPSA activities involved increasing the use of Xpert, thus increasing the early detection of rifampicin resistance. For example, assuming PPSA activities are increased to their theoretical maximum coverage 14% (95% CrI 11-16%) and 35% (95% CrI 30-39%) of cumulative DR-TB cases and deaths would be averted between 2020 and 2035, respectively. This impact increases to 18% (95% CrI 15-22%) and 41% (95% CrI 35-47%) DR-TB cases and deaths averted in Delhi, respectively, due to the lower current levels of Xpert uptake in Delhi compared to Ahmedabad.

Percent reduction in cumulative TB burden, 2020-2035	**Ahmedabad**	**Delhi**
**Feasible maximum**		
TB incidence	5.1% (4.4-6.2)	5.4% (4.4-6.9)
TB deaths	4.1% (2.8-5.8)	6.4% (4.9-8.2)
DR-TB incidence	8.0% (6.2-9.7)	14% (11-17)
DR-TB deaths averted	20% (18-24)	32% (27-37)
**Theoretical maximum**		
TB incidence	7.4% (6.2-9.2)	6.7% (5.3-8.9)
TB deaths	7.9% (5.7-11)	8.7% (6.8-11.3)
DR-TB incidence	14% (11-16)	18% (15-22)
DR-TB deaths averted	35% (30-39)	41% (35-47)

For peer review only - http://bmjopen.bmj.com/site/about/guidelines.xhtml

Table 8. Projected epidemiological impact. Impact is measured as the cumulative impact on TB cases and deaths in Ahmedabad and Delhi between 2020 and 2035 relative to a baseline of current PPSA scale up

In Ahmedabad, the interventions with the lowest ICERS were provider activation and promoting FDC use, costing an estimated cost $18,000 (12,000-29,000) and $36,000 (26,000-56,000) per TB case averted between 2020 and 2035, assuming the interventions were scaled up to their feasible maximum. Despite their low ICERs, these interventions were also the least epidemiologically impactful (table S14; fig 3). The interventions with the highest ICERS were those promoting Xpert, costing $120,000 (96,000-180,000) and $46,000 (37,000-68,000) per TB case averted depending on whether Xpert use was promoted amongst occasional users and non-users, respectively. For Delhi, promoting FDC use also had the lowest ICER costing an estimated $170,000 (110,000-310,000) per TB case averted between 2020 and 2035 (table S15, fig 3). However, promoting the use of Xpert amongst non-users and provider activation had similar ICERs at $210,000 (150,000-320,000) and $280,000 (200,000-400,000) per TB case averted, respectively. Similar to Ahmedabad, promoting the use of Xpert amongst occasional users had the highest ICER at $780,000 (500,000-900,000) per TB case averted. Fig 3 summarises the order in which the different interventions should be prioritised in Ahmedabad and Delhi, and tables S16-S17 provide a breakdown of the total costs.

Figures S6 – S8 show results of sensitivity analyses. Alternate assumptions for the proportion of active providers that remain consistently active and the proportion of TB patients with exclusively extrapulmonary TB reduced the epidemiological impact of increasing PPSA service provisions (fig S6) The qualitative findings shown in fig 3 were unchanged by alternative assumptions on the uptake of Xpert and treatment adherence amongst newly-recruited providers (table S14). However, assumptions relating to maintenance costs (the cost associated with maintaining good behaviour amongst active providers) showed some influence on the prioritisation of interventions. For Ahmedabad, decreasing maintenance cost by 20% resulted in the promotion of government supplied FDCs being the priority intervention (table S18). On the other hand, increasing the proportion of active providers remained the individual intervention with the lowest ICER if maintenance cost increased by 20%. For Delhi, increasing the proportion of FDC users remained the intervention with the lowest ICER regardless of a 20% increase or decrease in maintenance cost (table S19).

Finally, we conducted multivariate sensitivity analysis to identify the parameters that are most influential for model outputs. Model projections for TB cases averted in Ahmedabad were influenced most strongly by the following three parameters: the rate of relapse following self-cure, the rate of stabilisation of the risk of relapse following treatment, and the rate of repeat care-seeking (fig S7). For Delhi, the three most influential parameters were also the rate of relapse following self-cure and the rate of stabilisation of the risk of relapse following treatment, as well as the rate of progression to active disease from latency (fig S8).

For peer review only - http://bmjopen.bmj.com/site/about/guidelines.xhtml

Discussion

Our analysis focuses on settings that have already seen an introduction of JEET services, and addresses how these services should be expanded in future. Focusing on Ahmedabad and Delhi, two cities with different coverage levels of PPSA services, our analysis suggests that JEET operations should prioritise working with already-engaged providers to encourage them to participate more actively in Ahmedabad, and increasing the use of government supplied FDCs amongst already-active providers in Delhi (fig 3). Intervention priorities may vary in different settings across India, depending on local conditions, and the existing degree of uptake of PPSA services. For example, a key reason for the importance of increasing the use of FDCs in Delhi is the relatively low uptake of FDCs there so far. Overall, this work illustrates how programmatic data could be analysed using transmission modelling, to identify priorities for any given setting.

Previous modelling work estimated the potential epidemiological impact of scaling up PPSA operations in Mumbai and Patna, the cities where the PPSA pilots were first implemented (8). Subsequent analysis used a relatively simple costing model to illustrate how PPSA scaleup would be cost-effective in both settings (5). However, both analyses were performed prospectively, and thus we had to make assumptions about the effectiveness of different interventions, in cities where PPSA operations did not already exist at scale. Now that JEET has been operating at scale in India, there is detailed programmatic data available for the costs, effectiveness, and real-world coverage of different interventions: our analysis thus adds to previous work by analysing this data in a systematic way, to identify operational priorities for future expansion of PPSA activities, in given cities.

Overall, the interventions with the highest ICERs are those that involve Xpert. Although these interventions are epidemiologically impactful, they are costly, due to the high cost of an Xpert test; the cost of testing symptomatic non-TB infected care-seekers; and the cost of engaging with the active providers to increase their use of Xpert. Reducing the effort needed to convince private providers to increase Xpert use will be key in decreasing the overall cost. A recent study found that larger providers may be more willing to change their behaviour and adopt the use of Xpert compared to smaller providers; thus, targeting these larger providers may provide a more cost-efficient solution and help bring down the cost of increasing the use of Xpert amongst private providers (6). On the other hand, promoting uptake of FDCs – although having modest impact – also consistently showed the lowest estimated ICERs, because of its low cost.

The epidemiological impact is likely to be reduced if there is a high rate of attrition from an active to an inactive status amongst private providers. There are several reasons behind this attrition: first, some providers may not have a sufficient load of TB patients every month and thus may notify infrequently depending on when they see a new TB patient; second, some providers, especially those handling a smaller volume of TB patients, are seen less regularly by the field team and are therefore less likely to notify; third, some providers remain dissatisfied with the services offered by JEET, and therefore intentionally stop notifying patients; fourth, providers may be wary of the

For peer review only - http://bmjopen.bmj.com/site/about/guidelines.xhtml

scrutiny they receive once they become active and involved with the programme. Understanding the reasons behind this behaviour will be important to ensure engagement efforts do not go to waste.

Limitations of this work can be divided into two categories: transmission model limitations and economic limitations. Owing to a lack of city-specific data, we drew from prevalence and infection surveys from Chennai, another large Indian city. This analysis focusses on urban settings and does not address rural settings, that may have different care-seeking pathways, different rates of TB infection and different provider behaviour (1,12). While JEET activities have so far focused on urban India, addressing needs for private sector engagement in rural settings is an important area for future work. On the cost side, we had to make certain assumptions to fill data gaps. For example, there is no systematic data for the number of visits needed to change the behaviour of a provider; this parameter was estimated by the CHAI field team as a form of expert opinion. Another assumption we have made is that the field team visits providers resistant to engagement efforts the same amount of time as non-resistant providers; in reality, after a couple of visits, field officers know which providers are resistant, and thus, may choose instead to focus their efforts on non-resistant providers. Similarly, we do not consider the fact that once a provider has been visited by field staff a certain number of times, any subsequent visits are likely to be ineffective; for example, data from the field team suggests that the increase in a provider's uptake of Xpert becomes negligible after approximately the 25th visit by a field officer.

In summary, as India accelerates efforts for drastically reducing TB burden, engaging effectively with the private sector will play a critical role. Modelling analysis can play a useful role in supporting these efforts, not only in informing strategic planning, but also in addressing operational priorities in different settings. Identifying the most cost-efficient ways of achieving PPSA impact will be critical in benefiting TB patients, in the future of India's TB response.

Contributorship statement

NA conceptualized and supervised the study. All authors contributed to the methodology. SR conducted the formal analysis. NA and SR wrote the original draft. AP, RS and AS contributed to data curation and reviewed the draft.

Competing interests

None declared.

Funding

SR was supported by the Wellcome Trust, grant number 203839/A/16/Z.

Data sharing statement

All data relevant to the study are included in the article or uploaded as supplementary information.

For peer review only

Ethics approval

This study does not involve human participants or animal subjects.

For peer review only

References

1. Satyanarayana S, Nair SA, Chadha SS, Shivashankar R, Sharma G, Yadav S, et al. From Where Are Tuberculosis Patients Accessing Treatment in India? Results from a Cross-Sectional Community Based Survey of 30 Districts. *PLOS ONE*. 2011;6(9): e24160. https://doi.org/10.1371/journal.pone.0024160.

2. Sreeramareddy CT, Qin ZZ, Satyanarayana S, Subbaraman R, Pai M. Delays in diagnosis and treatment of pulmonary tuberculosis in India: a systematic review. *The International Journal of Tuberculosis and Lung Disease: The Official Journal of the International Union Against Tuberculosis and Lung Disease*. 2014;18(3): 255–266. https://doi.org/10.5588/ijtld.13.0585.

3. Arinaminpathy N, Batra D, Khaparde S, Vualnam T, Maheshwari N, Sharma L, et al. The number of privately treated tuberculosis cases in India: an estimation from drug sales data. *The Lancet Infectious Diseases*. 2016;16(11): 1255–1260. https://doi.org/10.1016/S1473-3099(16)30259-6.

4. Kundu D, Chopra K, Khanna A, Babbar N, Padmini TJ. Accelerating TB notification from the private health sector in Delhi, India. *The Indian Journal of Tuberculosis*. 2016;63(1): 8–12. https://doi.org/10.1016/j.ijtb.2016.02.002.

5. Deo S, Jindal P, Gupta D, Khaparde S, Rade K, Sachdeva KS, et al. What would it cost to scale-up private sector engagement efforts for tuberculosis care? Evidence from three pilot programs in India. *PLOS ONE*. 2019;14(6): e0214928.

6. Deo S, Jindal P, Papineni S. Integrating Xpert MTB/RIF for TB diagnosis in the private sector: evidence from large-scale pilots in Patna and Mumbai, India. *BMC Infectious Diseases*. 2021;21(123). https://doi.org/10.1186/s12879-021-05817-1.

7. Sachdeva KS, Kumar A, Dewan P, Kumar A, Satyanarayana S. New vision for Revised National Tuberculosis Control Programme (RNTCP): Universal access - 'reaching the un-reached'. *The Indian Journal of Medical Research*. 2012;135(5): 690–694.

8. Arinaminpathy N, Deo S, Singh S, Khaparde S, Rao R, Vadera B, et al. Modelling the Impact of Effective Private Provider Engagement on Tuberculosis Control in Urban India. *Scientific Reports*. 2019;9(3810).

9. Joint Effort for Elimination of Tuberculosis. *Project JEET*. 2019. https://www.projectjeet.in

10. World Health Organisation. *A brief history of tuberculosis control in India*. 2010. https://apps.who.int/iris/bitstream/handle/10665/44408/9789241500159_eng.pdf?sequence=1

11. Central TB Division, Directorate General of Health Services, Ministry of Health with Family Welfare. *National Strategic Plan for Tuberculosis Elimination 2017-2025*. 2017.

12. Subbaraman R, Nathavitharana RR, Satyanarayana S, Pai M, Thomas BE, Chadha VK, et al. The Tuberculosis Cascade of Care in India's Public Sector: A Systematic Review and Meta-analysis. *PLOS Medicine*. 2016;13(10): e1002149. https://doi.org/10.1371/journal.pmed.1002149.

13. Houben RMGJ, Lalli M, Kranzer K, Menzies NA, Schumacher SG, Dowdy DW. What if they don't have tuberculosis? The consequences and trade-offs involved in false-positive diagnoses of tuberculosis. *Clinical Infectious Diseases*. 2019;68(1): 150–156. https://doi.org/10.1093/cid/ciy544.

14. Sachdeva KS, Raizada N, Sreenivas A, Van't Hoog A, van den Hof S, Dewan PK, et al. Use of Xpert MTB/RIF in decentralized public health settings and its effect on pulmonary TB and DR-TB case findings in India. *PLOS ONE*. 2015;10(5): e0126065. https://doi.org/10.1371/journal.pone.0126065.

15. Gopi PG, Prasad VV, Vasantha M, Subramani R, Tholkappian AS, Sargunan D, et al. Annual risk of tuberculosis infection in Chennai city. *Indian Journal of Tuberculosis*. 2008;55(3): 157–161.

16. Dhanaraj B, Papanna MK, Adinarayanan S, Vedachalam C, Sundaram V, Shanmugam S, et al. Prevalence and risk factors for adult pulmonary tuberculosis in a metropolitan city of South India. *PloS One*. 2015;10(4): e0124260. https://doi.org/10.1371/journal.pone.0124260.

17. Haario H, Saksman E, Tamminen J. An adaptive metropolis algorithm. *Bernoulli*. 2001;7(2): 223–242.

18. Ku C-C, MacPherson P, Khundi M, Soko RHN, Feasey HRA, Nliwasa M, et al. Durations of asymptomatic, symptomatic, and care-seeking phases of tuberculosis disease with a Bayesian analysis of prevalence survey and notification data. *BMC Medicine*. 2021;19(1): 298. https://doi.org/10.1186/s12916-021-02128-9.

19. Stallworthy G, Dias HM, Pai M. Quality of tuberculosis care in the private health sector. *Journal of Clinical Tuberculosis and Other Mycobacterial Diseases*. 2020;20: 100171. https://doi.org/10.1016/j.jctube.2020.100171.

20. Pai M, Daftary A, Satyanarayana S. TB control: challenges and opportunities for India. *Transactions of the Royal Society of Tropical Medicine and Hygiene*. 2016;110(3): 158–160.

21. Das J, Kwan A, Daniels B, Satyanarayana S, Subbaraman R, Bergkvist S, et al. Use of standardised patients to assess quality of tuberculosis care: a pilot, cross-sectional study. *Lancet Infectious Diseases*. 2015;15(11): 1305–1313. https://doi.org/10.1016/S1473-3099(15)00077-8.

22. Van't Hoog A, Langendam MW, Mitchell E, Cobelens FG, Sinclair D, Leeflang MMG, et al. *A sytematic review of the sensitivity and specificity of symptom- and chest-radiography screening for active pulmonary tuberculosis in HIV-negative persons and persons with unknown HIV status*. 2013. https://www.who.int/tb/Review2Accuracyofscreeningtests.pdf

23. Steingart KR, Horne DJ, Pai M, Boehme CC, Dendukuri N. Xpert MTB/RIF assay for pulmonary tuberculosis and rifampicin resistance in adults (Review). *Cochrane Database of Systematic Reviews*. 2014;(1).

24. World Health Organisation. *Global Tuberculosis Report 2020*. 2020. https://www.who.int/publications/i/item/9789240013131

25. Menzies D, Benedetti A, Paydar A, Martin I, Royce S, Pai M, et al. Effect of Duration and Intermittency of Rifampin on Tuberculosis Treatment Outcomes: A Systematic Review and Meta-Analysis. *PLOS Med*. 2009;6(9): e1000146. https://doi.org/10.1371/journal.pmed.1000146.

26. Vynnycky E, Fine PE. The natural history of tuberculosis: the implications of age-dependent risks of disease and the role of reinfection. *Epidemiology and Infection*. 1997;119(2): 183–201.

For peer review only

27. Dye C, Garnett GP, Sleeman K, Williams BG. Prospects for worldwide tuberculosis control under the WHO DOTS strategy. Directly observed short-course therapy. *Lancet (London, England)*. 1998;352(9144): 1886–1891.

28. Horsburgh CR, O'Donnell M, Chamblee S, Moreland JL, Johnson J, Marsh BJ, et al. Revisiting rates of reactivation tuberculosis: a population-based approach. *American Journal of Respiratory and Critical Care Medicine*. 2010;182(3): 420–425. https://doi.org/10.1164/rccm.200909-1355OC.

29. Thomas A, Gopi PG, Santha T, Chandrasekaran V, Subramani R, Selvakumar N, et al. Predictors of relapse among pulmonary tuberculosis patients treated in a DOTS programme in South India. *International Journal of Tuberculosis and Lung Disease*. 2005;9(5): 556–561.

30. Tiemersma EW, van der Werf MJ, Borgdorff MW, Williams BG, Nagelkerke NJD. Natural history of tuberculosis: duration and fatality of untreated pulmonary tuberculosis in HIV negative patients: a systematic review. *PloS One*. 2011;6(4): e17601. https://doi.org/10.1371/journal.pone.0017601.

31. Andrews JR, Noubary F, Walensky RP, Cerda R, Losina E, Horsburgh CR. Risk of progression to active tuberculosis following reinfection with Mycobacterium tuberculosis. *Clinical Infectious Diseases*. 2012;54(6): 784–791.

32. Veesa KS, John KR, Moonan PK, Kaliappan SP, Manjunath K, Sagili K, et al. Diagnostic Pathways and Direct Medical Costs Incurred by New Adult Pulmonary Tuberculosis Patients Prior to Anti-Tuberculosis Treatment - Tamil Nadu, India. *PLOS ONE*. 2018;13(2): e0191591.

33. Pandey S, Chadha VK, Laxminarayan R, Arinaminpathy N. Estimating tuberculosis incidence from primary survey data: a mathematical modeling approach. *The International Journal of Tuberculosis and Lung Disease*. 2017;21(4): 366–374. https://doi.org/10.5588/ijtld.16.0182.

34. Sachdeva KS, Satyanarayana S, Dewan P, Nair SA, Reddy R, Kundu D, et al. Source of Previous Treatment for Re-Treatment TB Cases Registered under the National TB Control Programme, India, 2010. *PLOS ONE*. 2011;6(7): e22061.

35. Subbaraman R, Nathavitharana RR, Mayer KH, Satyanarayana S, Chadha VK, Arinaminpathy N, et al. Constructing care cascades for active tuberculosis: A strategy for program monitoring and identifying gaps in quality of care. *PLOS Medicine*. 2019;16(2): e1002754.

36. World Bank. *Data: India*. 2015 [Accessed 12th May 2016]. http://data.worldbank.org/country/india [Accessed 12th May 2016].

37. Menzies NA, Gomez GB, Bozzani F, Chatterjee S, Foster N, Baena IG, et al. Cost-effectiveness and resource implications of aggressive action on tuberculosis in China, India, and South Africa: a combined analysis of nine models. *The Lancet Global Health*. 2016;4(11): e816–e826. https://doi.org/10.1016/S2214-109X(16)30265-0.

For peer review only

Figures

Figure 1. Schematic illustration of the model structure. Panel A illustrates the TB transmission model structure**.** Model compartments are as follows: uninfected with TB (U), latent infection (L), active TB disease (I), presented for care and awaiting diagnosis (Dx), on TB treatment (Tx), temporarily dropped out of the TB care cascade due to missed diagnosis or pre-treatment loss-to-follow-up (E), and recovered with a low risk of relapse ($R^{(L)}$) or with a high risk of relapse ($R^{(H)}$). Dx and Tx are stratified by healthcare sector: private (pr) and public (pu) sector. The private sector is further subdivided into several types of providers as described in the main text. Not shown for simplicity is the acquisition and transmission of DR-TB. See supplementary information for further technical details, including model equations and calibration. Panel B illustrates the non-infected TB-symptomatic model structure. Model compartments are as follows: uninfected with TB but TB symptomatic ($U^{(NTB)}$), presented for care and awaiting diagnosis in the public sector ($Dx^{(NTB\text{-}Pu)}$) or private sector ($Dx^{(NTB\text{-}Pr)}$), and TB treatment after a false-positive diagnosis in the public sector ($Tx^{(NTB\text{-}Pu)}$) or private sector ($Tx^{(NTB\text{-}Pr)}$). The private sector is further subdivided into several types of providers as described in the main text. In order to maintain a constant population size amongst the non-infected TB-symptomatic population, we assume that upon treatment completion, individuals return to the uninfected TB-symptomatic state. The size of this population was determined so that amongst all patients presenting for care, there is a 10% prevalence of TB, in either the public or private sector (12–14).

Figure 2. Model projections for the impact of increasing all PPSA service provisions to their feasible or theoretical maximum has on TB incidence in Ahmedabad and Delhi between 2020 and 2035. Panel A and B show the projected trajectory of TB incidence in Ahmedabad and Delhi, respectively, assuming current PPSA scale up between 2017 and 2020, and its maintenance indefinitely (blue-dashed line), assuming that PPSA service provisions are increased to their feasible or theoretical maximum from 2020 to 2021 and maintained indefinitely (green and purple line, respectively). Shaded areas show 95% credible intervals.

Figure 3. Expansion pathway of PPSA service provisions in Ahmedabad (panel A) and Delhi (panel B). The first point on the graph represents the intervention that had the lowest ICER on its own when scaled up to its feasible maximum coverage; the second point represents the additional PPSA, in combination with the first point, had the lowest ICER; and so on, until all interventions are included. Shaded area shows the 95% credible interval. See tables S14-15 for further details.

For peer review only - http://bmjopen.bmj.com/site/about/guidelines.xhtml

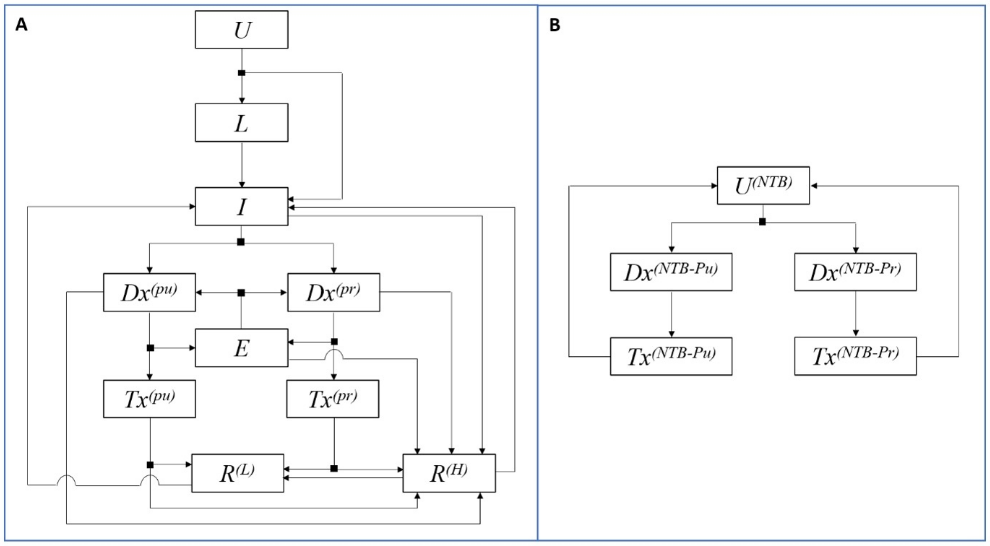

Figure 1. Schematic illustration of the model structure. Panel A illustrates the TB transmission model structure. Model compartments are as follows: uninfected with TB (U), latent infection (L), active TB disease (I), presented for care and awaiting diagnosis (Dx), on TB treatment (Tx), temporarily dropped out of the TB care cascade due to missed diagnosis or pre-treatment loss-to-follow-up (E), and recovered with a low risk of relapse (R(L)) or with a high risk of relapse (R(H)). Dx and Tx are stratified by healthcare sector: private (pr) and public (pu) sector. The private sector is further subdivided into several types of providers as described in the main text. Not shown for simplicity is the acquisition and transmission of DR-TB. See supplementary information for further technical details, including model equations and calibration. Panel B illustrates the non-infected TB-symptomatic model structure. Model compartments are as follows: uninfected with TB but TB symptomatic (U(NTB)), presented for care and awaiting diagnosis in the public sector (Dx(NTB-Pu)) or private sector (Dx(NTB-Pr)), and TB treatment after a false-positive diagnosis in the public sector (Tx(NTB-Pu)) or private sector (Tx(NTB-Pr)). The private sector is further subdivided into several types of providers as described in the main text. In order to maintain a constant population size amongst the non-infected TB-symptomatic population, we assume that upon treatment completion, individuals return to the uninfected TB-symptomatic state. The size of this population was determined so that amongst all patients presenting for care, there is a 10% prevalence of TB, in either the public or private sector (12–14).

159x90mm (220 x 220 DPI)

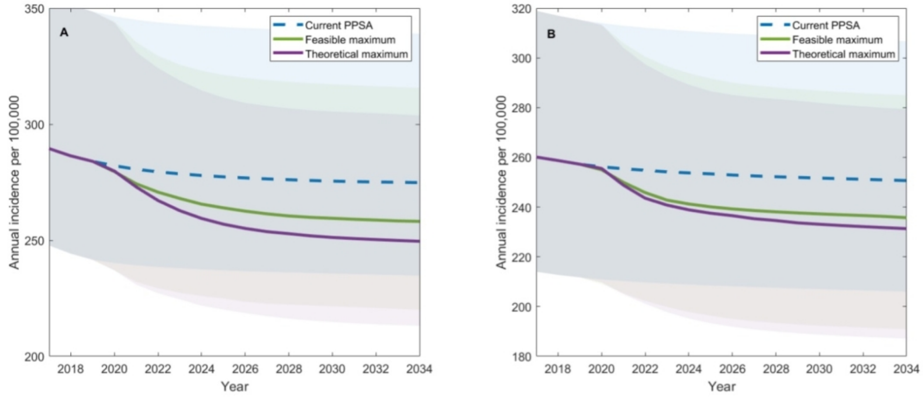

Figure 2. Model projections for the impact of increasing all PPSA service provisions to their feasible or theoretical maximum has on TB incidence in Ahmedabad and Delhi between 2020 and 2035. Panel A and B show the projected trajectory of TB incidence in Ahmedabad and Delhi, respectively, assuming current PPSA scale up between 2017 and 2020, and its maintenance indefinitely (blue-dashed line), assuming that PPSA service provisions are increased to their feasible or theoretical maximum from 2020 to 2021 and maintained indefinitely (green and purple line, respectively). Shaded areas show 95% credible intervals.

189x81mm (220 x 220 DPI)

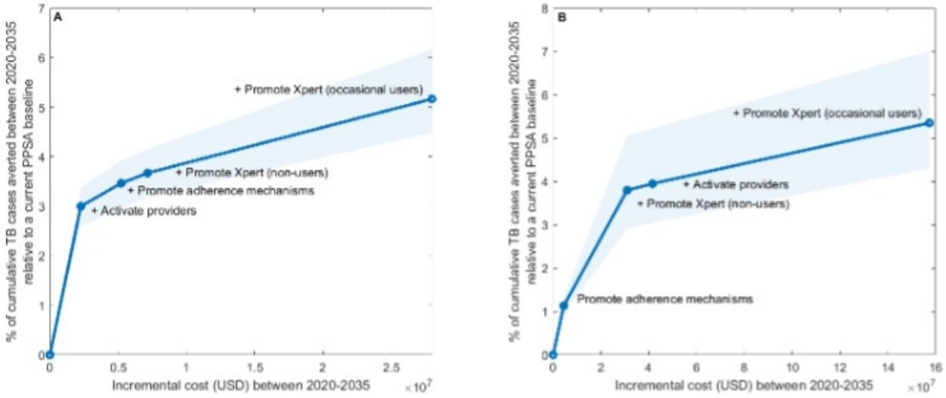

Figure 3. Expansion pathway of PPSA service provisions in Ahmedabad (panel A) and Delhi (panel B). The first point on the graph represents the intervention that had the lowest ICER on its own when scaled up to its feasible maximum coverage; the second point represents the additional PPSA, in combination with the first point, had the lowest ICER; and so on, until all interventions are included. Shaded area shows the 95% credible interval. See tables S14-15 for further details.

207x88mm (96 x 96 DPI)

Appendix 3

The value and cost efficiency of private sector engagement

1. Materials and methods

Below, we describe the model, including its structure, governing equations, and table of parameters. The model was implemented in MATLAB.

1.1. Model structure

We modelled TB transmission amongst adults (>15 years old) in a typical urban setting in India. We modelled the fragmented healthcare system, by differentiating between the public and private sector (1–7). We further divided the private sector by engagement status, Xpert use and FDC use (see main text for more details on these categories). We calibrated the model to TB prevalence, annual risk of infection, and mortality (table 1). We also modelled the acquisition and transmission of DR-TB, but ignored HIV status as well as distinctions between pulmonary and extrapulmonary TB. The overall model structure is illustrated schematically in fig 1.

For a given set of model parameters, we initially simulated the model to equilibrium after introducing TB into a disease-free population. From 1997 to 2007 and its maintenance until the present day, we incorporated the scale-up of RNTCP (India's national TB programme) and its impact on the TB epidemic (8). From 1997, we also incorporated population growth. From 2017 to 2020, we simulated the scale-up of PPSA services to current 2019 levels. We then simulated the model forward from 2020 to 2035.

1.2. Governing equations for the mathematical transmission model

The equations correspond to the model described in fig 1. State variables (capital letters) are as listed in table S1, while model parameters (lower-case and Greek letters) are as listed in tables 2-5.

For peer review only

Model stage	Description
U	Uninfected
L	Latent infection
I_q	Active TB
$Dx_q^{(pu)}$	Sought care, awaiting TB diagnosis, public sector
$Dx_q^{(pr)}$	Sought care, awaiting TB diagnosis, private sector. This is split further depending on engagement status and Xpert use (table S2)
$Tx_q^{(FL:pu)}$	Undergoing first line TB treatment, public sector
$Tx_q^{(FL:pr)}$	Undergoing first line TB treatment, private sector. This is split further depending on FDC use and adherence support offered (table S2).
$Tx^{(SL)}$	Undergoing second line TB treatment.
E_q	Between seeking care, after misdiagnosis or loss-to-follow-up
$R^{(L)}$	Recovered after treatment completion, low risk of relapse
$R^{(H)}$	Recovered after self-cure or incomplete treatment, high risk of relapse
**Subscript**	**Description**
q	Drug resistance status (0 = DS-TB, 1 = DR-TB)

Table S1. Model stages and subscript description

Label	Description
	**Private sector diagnosis classification**
pr1	Unengaged private providers
pr2	Engaged, inactive private providers
pr3	Engaged, active private providers that send samples for confirmatory Xpert testing 100% of the time
pr4	Engaged, active private providers that send samples for confirmatory Xpert testing occasionally, Xpert
pr5	Engaged, active private providers that send samples for confirmatory Xpert testing occasionally, no Xpert
pr6	Engaged, active private providers that never send samples for confirmatory Xpert testing
	**Private sector first-line treatment classification**
prZ1	Treatment offered by inactive and unengaged private providers
prAF	Engaged, active private providers that offer adherence support and government supplied FDCs
prA	Engaged, active private providers that only offer adherence support
prF	Engaged, active private providers that only offer government supplied FDCs
prZ2	Engaged, active private providers that offer neither adherence support or government supplied FDCs

Table S2. Private sector classification

Label	Classes
S_1	pu, prZ1, prZ2, prAF, prA, prF
S_2	pr1, pr2, pr5, pr6
S_3	pr3, pr4

Table S3. Notation

For peer review only

Uninfected stage

$$\frac{dU}{dt} = T - (\lambda_q + \mu_2)U \quad (1)$$

where term T represents births. To maintain a constant population for simplicity, we assumed that T is equivalent to the number of deaths occurring in the model.

Latent TB infection

$$\frac{dL_q}{dt} = (1-\theta)\lambda_q U + \pi\lambda_q(1-\theta) - (\rho + \mu_2 + \lambda_q)L_q \quad (2)$$

Active disease, pre-careseeking

$$\frac{dI_q}{dt} = \theta\lambda_q U + \pi\lambda_q\theta + \rho L_q + r_1 R^{(H)} + r_2 R^{(L)} - (\varphi + \delta_1 + \mu_1 + \mu_2)I_q \quad (3)$$

where the subscript q denotes drug resistance status:

$$q = \begin{cases} 0 & DS\ TB \\ 1 & DR\ TB \end{cases}$$

We assumed that, when presenting for care, a proportion η of patients visit the public sector, the remainder $(1-\eta)$ visiting the private sector. We divided the private sector further depending on whether private providers are engaged by JEET or not, and whether engaged providers are active or inactive, in notifying TB. Finally, active providers were categorised by their use of Xpert (table S2). The proportion of patients that visit each different type of provider is summarised in table S4.

For peer review only

Provider type	Notation	Formula
Public	$v^{(pu)}$	$v^{(pu)} = \eta$
Unengaged, private	$v^{(pr1)}$	$v^{(pr1)} = (1-\eta)(1-h^{\propto})^{*}$
Engaged, inactive, private	$v^{(pr2)}$	$v^{(pr2)} = (1-\eta)h^{\propto}(1-a)$
Engaged, active, private (always use Xpert)	$v^{(pr3)}$	$v^{(pr3)} = (1-\eta)h^{\propto}ap_{Xpert1}$
Engaged, active, private (occasionally use Xpert, patient receives an Xpert test)	$v^{(pr4)}$	$v^{(pr4)} = (1-\eta)h^{\propto}a(1-p_{Xpert1}-p_{Xpert0})x_p$
Engaged, active, private (occasionally use Xpert, patient does not receive an Xpert test)	$v^{(pr5)}$	$v^{(pr5)} = (1-\eta)h^{\propto}a(1-p_{Xpert1}-p_{Xpert0})(1-x_p)$
Engaged, active, private (non Xpert user)	$v^{(pr6)}$	$v^{(pr6)} = (1-\eta)h^{\propto}ap_{Xpert0}$

Table S4. Proportion of patients that visit different providers. Private providers under the classifications pr1, pr2, pr5 and pr6 rely on clinical diagnosis, whereas private providers under the classification pr3 and pr4 employ Xpert testing. *h is the proportion of private providers that have been engaged by the PPSA, and α is a 'heterogeneity index' governing the distribution of patient load across all providers (see fig S1).

The following equations describe the different pathways for patients that seek care with the different providers described above:

Seeking care, awaiting TB diagnosis

$$\frac{dDx_q^{(pu)}}{dt} = v^{(pu)}\delta_1 I_q + v^{(pu)}\delta_2 E_q$$

$$-\left[\varepsilon_1\omega\phi\left(1-s_q^{(pu)}\right) + \left(\varepsilon_1\omega\phi s_q^{(pu)}\right)_q + (1-\omega\varepsilon_1)\phi s_q^{(pu)} + \varphi + \mu_1 + \mu_2\right] Dx_q^{(pu)} \quad (4)$$

$$\frac{dDx_q^{(s)}}{dt} = v^{(s)}\delta_1 I_q + v^{(s)}\delta_2 E_q$$

$$-\left[\varepsilon_3\omega\phi(1-s_q^{(pr0)}) + (\varepsilon_3\omega\phi s_q^{(pr0)})_q + (1-\omega\varepsilon_3)\phi s_q^{(pr0)} + \varphi + \mu_1 + \mu_2\right] Dx_q^{(s)} \quad (5)$$

For peer review only

$$\frac{dDx_q^{(h)}}{dt} = v^{(h)}\delta_1 I_q + v^{(h)}\delta_2 E_q$$

$$-\left[\varepsilon_2\omega\phi(1 - s_q^{(pr1)}) + (\varepsilon_2\omega\phi s_q^{(pr1)})_q + (1 - \omega\varepsilon_2)\phi s_q^{(pr1)} + \varphi + \mu_1 + \mu_2\right] Dx_q^{(h)} \quad (6)$$

Where superscript (s) represents either pr1, pr2, pr5 or pr6 and superscript (h) represents either pr3 or pr4 as defined in table S4.

Patients that are misdiagnosed (as false-negative) enter the compartment E, to re-initiate careseeking after a delay:

Temporarily dropped out of careseeking

$$\frac{dE_q}{dt} = (1 - \omega\varepsilon_1)\phi Dx_q^{(pu)} + \sum_{i\in S_3}[(1 - \omega\varepsilon_2)\phi]Dx_q^{(i)} - (\varphi + \delta_2 + \mu_1 + \mu_2)E_q$$

when q=0 (7)

$$\frac{dE_q}{dt} = (1 - \omega\varepsilon_1)\phi s_{pu} Dx_q^{(pu)} + \sum_{i\in S_2}[(1 - \omega\varepsilon_3)\phi s_{pr0}]Dx_q^{(i)} + \sum_{i\in S_3}[(1 - \omega\varepsilon_2)\phi s_{pr1}]Dx_q^{(i)}$$

$$+\sum_{i\in S_1}\left[\tau(1 - p_{SL}) + \frac{\tau\chi_i}{1 - \chi_i}\right]Tx_q^{(i)} - (\varphi + \delta_2 + \mu_1 + \mu_2)E_q$$

when q=1 (8)

where the summand represents patients not being cured by first-line treatment, but not being transferred to second-line treatment. Subscript S_1, S_2, and S_3 are defined in table S3.

Treatment

We categorised active private providers by the treatment services offered: adherence support and government supplied FDCs (prAF); adherence support only (prA); government supplied FDCs only (prF); or neither (prZ2) (table S2). The proportion of patients at active providers that receive the different treatment services offered is defined in table S5.

For peer review only

Provider type	Notation	Formula
Adherence support and government supplied FDCs	$v^{(prAF)}$	$v^{(prAF)} = p_{adh1}p_{adh2}p_{fdc}$
Adherence support only	$v^{(prA)}$	$v^{(prA)} = p_{adh1}p_{adh2}(1-p_{fdc})$
Government supplied FDCs only	$v^{(prF)}$	$v^{(prF)} = (1-p_{adh1}p_{adh2})p_{fdc}$
Neither adherence support or government supplied FDCs	$v^{(prZ)}$	$v^{(prZ)} = (1-p_{adh1}p_{adh2})(1-p_{fdc})$

Table S5. Proportion of patients at active providers that receive treatment services.

Governing equations for the different Tx compartments are:

$$\frac{dTx_q^{(FL:y)}}{dt} = \varepsilon_i\phi\omega v^{(pr\_y)}Dx_q^{(y)} - (m_y + \tau + \frac{\tau\chi_y}{1-\chi_y} + \mu_2)Tx_q^{(FL:y)} \quad \text{when q = 0} \quad (9)$$

$$\frac{dTx_q^{(FL:y)}}{dt} = \varepsilon_i\phi\omega v^{(pr\_y)}(1-s_y)Dx_q^{(y)} + m_yTx_{q=0}^{(FL:y)} - (\tau p_{SL} + [\tau(1-p_{SL}) + \frac{\tau\chi_y}{1-\chi_y}] + \mu_2)Tx_q^{(FL:y)} \quad \text{when q = 1} \quad (10)$$

$dTx_q^{(FL:y)}$ represents the different Tx compartments (pu, prZ1, prAF, prA, prF and prz2), defined in table S2. The parameter ε_i is the proportion of patients tested that are correctly diagnosed (in other words, the sensitivity of the test) and is dependent on where the patient is diagnosed: ε_1 when Dx^{pu}; ε_3 when Dx^{pr1}, Dx^{pr2}, Dx^{pr5}, Dx^{pr6}; and ε_2 when Dx^{pr3}, Dx^{pr4}. The parameter ϕ is the treatment initiation delay (assumed conservatively to be 1 week across all providers) and ω is the proportion of diagnosed TB cases initiating TB treatment (assumed to be 0.87 across all providers) (9). $v^{(pr\_y)}$, described above, applies only to TB patients that are diagnosed by active private providers. s_y is the proportion of MDR-TB cases receiving drug susceptibility testing at the point of TB diagnosis and is dependent on the amount of Xpert testing. We assume the proportion is 0.45, 1.00 and 0 in the public sector ($s^{(pu)}$), respectively amongst private providers that always use Xpert, and amongst private providers that do not use Xpert, respectively (10). Thus, $1-s_y$ represents TB patients with DR-TB that are not tested for drug resistance and are therefore mistakenly initiated onto first-line treatment. The parameter m_y is the rate of MDR acquisition while on first-line

For peer review only

treatment and is assumed to be higher amongst all private providers (m_b) except active providers that offer government supplied FDCs and adherence support, than public sector providers (m_a) (11). The parameter τ is the per-capita rate of treatment completion for first line treatment and its value corresponds to a duration of 6 months.

The parameter p_{SL} applies only to TB patients with DR-TB and is defined as the proportion of DR-TB cases that are successfully transferred to second-line treatment following failure of first-line treatment. The parameter χ_y is the proportion of TB cases that do not complete, or are non-adherent to, first line treatment; it is dependent on the quality of treatment services offered by providers and is informed by programmatic data (tables 2-5).

The following equation represents the number of DR-TB patients receiving second-line treatment:

$$\frac{dTx_q^{(SL)}}{dt} = \varepsilon_1\omega\phi s_{pu}Dx_q^{(pu)} + \varepsilon_3\omega\phi s_{pro}\sum_{i\in S_2} Dx_q^{(i)} + \varepsilon_2\omega\phi s_{pr1}\sum_{i\in S_3} Dx_q^{(i)}$$

$$+\tau p_{SL}\sum_{i\in S_1} Tx_q^{(i)} - (\tau_{mdr} + \frac{\tau_{mdr}\chi_{\mathrm{mdr}}}{1-\chi_{\mathrm{mdr}}} + \mu_2)Tx_q^{(SL)} \qquad (11)$$

Where S_1, S_2 and S_3 are sets of indices defined in table S3.

TB patients that successfully complete TB treatment, recover with a low risk of relapse. Governing equations for those with low risk of relapse are:

Recovery, low risk of relapse

$$\frac{dR^{(L)}}{dt} = \tau\sum_{i\in S_1} Tx_q^{(i)} + \varsigma R^{(H)} - (r_2 + \mu_2 + \lambda_q)R^{(L)} \qquad (12)$$

Where S_1 is the set of indices defined in table S3.

On the other hand, those recovered from TB with a high risk of relapse (either from previous inadequate treatment or self-cure) have governing equations:

For peer review only

Recovery, high risk of relapse

$$\frac{dR^{(H)}}{dt} = \frac{\tau\chi_{pu}}{1-\chi_{pu}} Tx_q^{(FL:pu)} + \sum_{i\in S_1} (\frac{\tau\chi_{(i)}}{1-\chi_{(i)}}) Tx_q^{(i)} + \varphi \sum_{i\in[S_{2,}S_3]} [Dx_q^{(pu)} + Dx_q^{(i)} + E_q + I_q]$$

$$-(\varsigma + r_1 + \mu_2 + \lambda_q) R^{(H)} \quad (13)$$

Where S_1, S_2 and S_3 are sets of indices defined in table S3.

The force of infection can be written as:

$$\lambda_q = \frac{\beta\left\{\sum_{i\in[S_2,S_3]}[I_q + Dx_q^{pu} + Dx_q^{(i)} + E_q]\right\} + d_q\beta\left\{\sum_{i\in[S_2,S_3]}[I_q + Dx_q^{pu} + Dx_q^{(i)} + E_q]\right\}}{N} \quad (14)$$

Where S_2 and S_3 are sets of indices defined in table S3.

Non-TB symptomatic population

To count the number of false positive diagnoses that arise, we modelled a non-TB symptomatic population (fig 1). We defined non-TB symptomatics as patients who do not have TB, but who have symptoms that would make them eligible for testing for TB. We assumed this population to be independent of the TB population described above.

Non-TB symptomatics seeking care

$$\frac{dU^{(NTB)}}{dt} = y_i \phi \omega Dx^{(NTB)} + \tau Tx^{(NTB)} - \delta_1 v^{(x)} U^{(NTB)} \quad (15)$$

where $v^{(x)}$ is drawn from equations 4 to 10, depending on the type of provider a patient seeks care from, and where y_i represents either the specificity of TB diagnosis in the public sector (y_1), specificity of Xpert (y_2) or the specificity of clinical diagnosis (y_3).

Awaiting diagnosis

$$\frac{dDx^{(NTB)}}{dt} = \delta_1 v^{(x)} U^{(NTB)} - \phi \omega Dx^{(NTB)} \quad (16)$$

For peer review only

Undergoing (unnecessary) first-line TB treatment

$$\frac{dTx^{(NTB)}}{dt} = (1 - y_i)\phi\omega Dx^{(NTB)} - \tau Tx^{(NTB)} \qquad (17)$$

We assumed the total population size of the non-TB symptomatic population remains constant; we determined the size of this population so that for every true positive TB diagnosis, approximately nine uninfected individuals who present for diagnosis with symptoms of TB are also tested for TB (9,12–16).

1.3. Model calibration

We performed calibrations using Adaptive Bayesian MCMC (table 1 -5)(17), allowing propagation of uncertainty from model inputs to projections in a systematic way.

We define the posterior distribution as:

$$p(\theta|D) \propto L(D|\theta)\,.\,\pi(\theta) \qquad (18)$$

where D is data; θ is a vector of model inputs subject to uncertainty; L is the likelihood of the data given θ, and π is the prior distribution. We assumed uniform distributions for the prior distributions. We defined the likelihood by fitting log-normal distributions to the calibration targets (see table 1).

Once the MCMC converged, we removed the burn-in period (10,000 iterations) and selected every 50th sample to reduce any autocorrelation. This provided 1200 samples from the posterior distribution.

For peer review only

2. Supporting figures

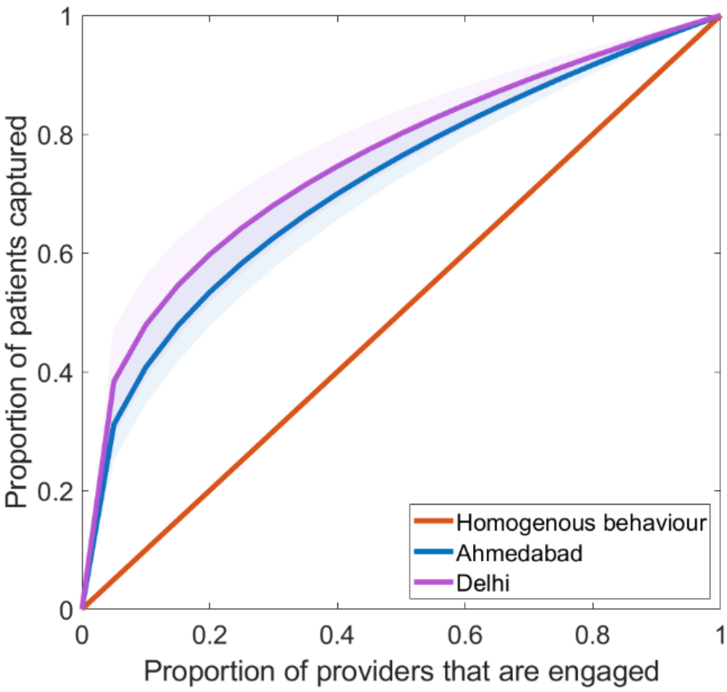

Figure S1. Heterogeneity in the patient share of private providers across Ahmedabad and Delhi. Homogenous patient share (red line) corresponds to a scenario where all private providers have the same numbers of patients. Under this assumption, increasing the proportion of engaged providers will linearly increase the proportion of patients benefiting from a PPSA. In reality, there are a few large private providers that manage most TB patients, and many smaller providers that manage few patients. The larger the area under the curve, the greater the heterogeneity. If the providers that capture the most patients are already engaged by JEET, increasing the proportion of engaged providers further will have little incremental benefit. Therefore, assuming homogenous patient share can overestimate the impact on TB epidemiology. As described in the main text, the 'heterogeneity index' controls the curvature of these plots. Table S7 shows the heterogeneity index calculated for each city.

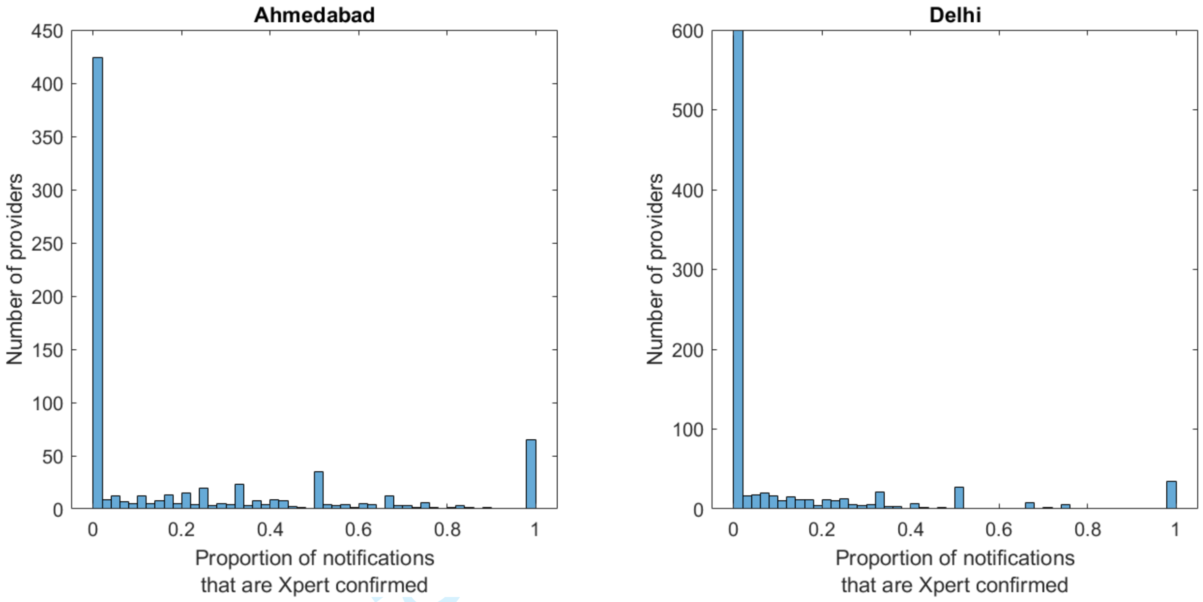

Figure S2. Xpert use amongst active private providers in Ahmedabad and Delhi in 2019. From this data, we classified active providers into three categories: those that always use Xpert, those that use Xpert occasionally, and those that never use Xpert. To be classified as occasionally using Xpert, we assume that providers need to use Xpert for at least 1% of notifications and less than 100% of notifications.

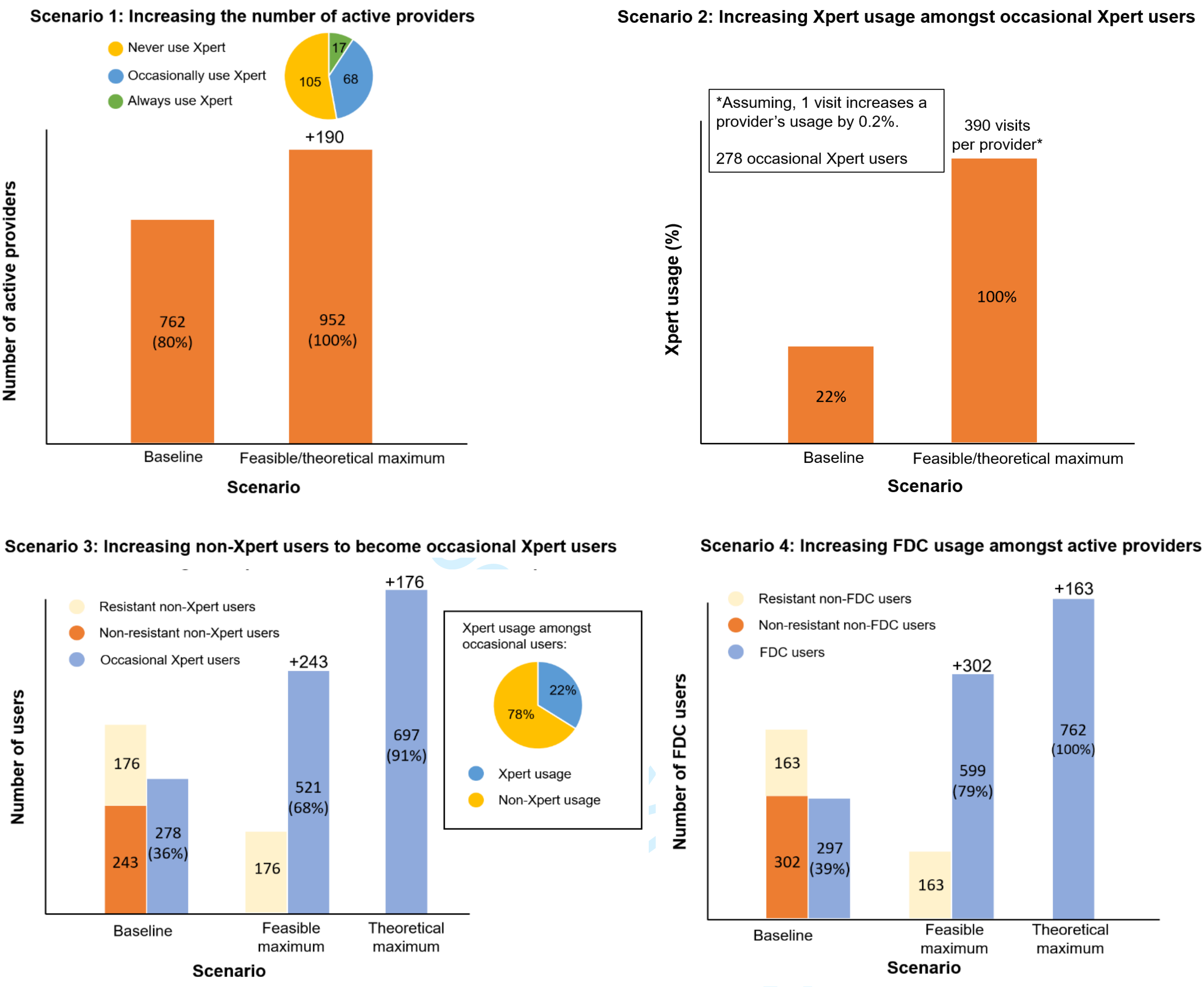

Figure S3. Feasible and theoretical maxima for each type of service provision, Ahmedabad. *Scenario 1:* We assume all inactive providers (e.g., 190) are converted into active providers. We assume no inactive providers are resistant, thus the feasible and theoretical maximum scenarios are the same. Newly active providers are assumed to have the same behaviour as pre-existing active providers; in the case of Ahmedabad, 9% use Xpert 100% of the time, 36% use Xpert occasionally (with an average Xpert usage of 22%), 55% never use Xpert, 98% offer adherence support and 39% consistently use governmental FDCs. For example, of the 190 newly active providers, 17 providers use Xpert 100% of the time, 68 use Xpert occasionally, and 105 never use Xpert. *Scenario 2:* At baseline, occasional Xpert users use Xpert 22% of the time; this is increased to 100% under the feasible and theoretical maximum scenario. We assume no occasional users are resistant to increasing their Xpert usage. It is estimated that one visit by a field officer increases a provider's usage of Xpert by 0.2%. At baseline, Ahmedabad has 278 occasional Xpert users. *Scenario 3:* At baseline, there are 419 non-Xpert users and 278 occasional users. Of the 419 non-Xpert users, it is estimated that 42% are resistant to using Xpert; thus, only 243 non-Xpert users can be converted to become an occasional user. At the theoretical maximum, the percentage of active providers that use Xpert occasionally is 91%; the remaining 9% are providers that always use Xpert. *Scenario 4:* At baseline, there are 465 non-FDC users and 297 consistent FDC users. Of the 465, 35% are resistant to FDCs.

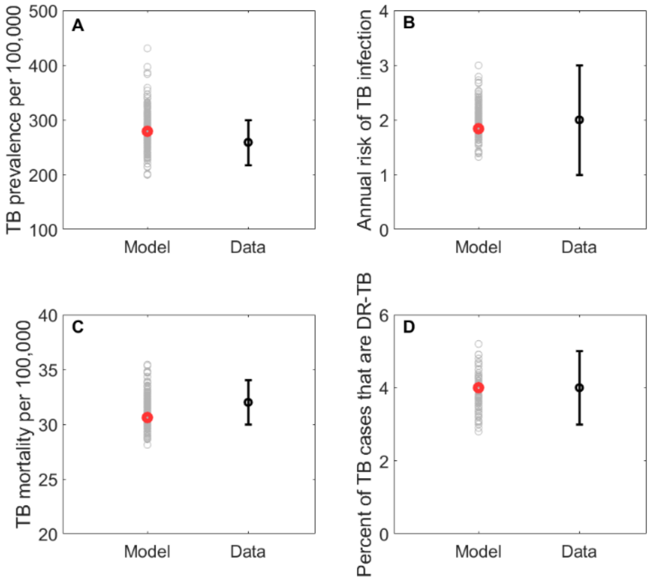

Figure S4. Model fits to data. Data points are described in table 1. Panel A – TB prevalence per 100,000 in a typical urban Indian setting; B – Annual risk of TB infection in a typical urban Indian setting; C – TB deaths per 100,000 in 2019, India; D – Percent of TB cases that are drug-resistant TB in 2019

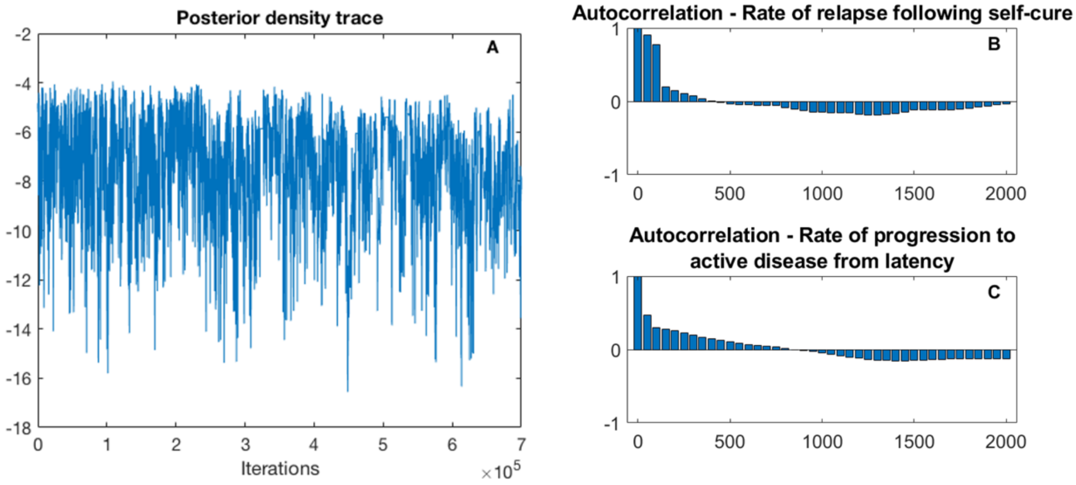

Figure S5. MCMC diagnostics. Panel A shows the posterior density trace, excluding the 'burn-in' period. Panel B and C show autocorrelation plots for two selected parameters (rate of relapse following self-cure and the rate of progression to active disease from latency).

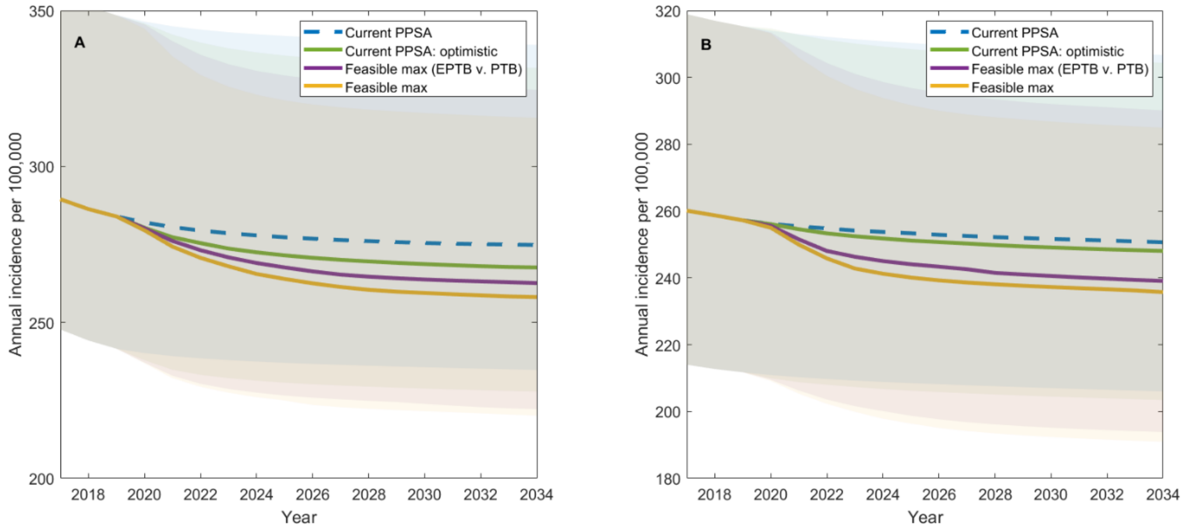

Figure S6. Sensitivity analysis on model projections for the impact of increasing all service provisions to their feasible maximum has on TB incidence in Ahmedabad and Delhi between 2020 and 2035. Panel A shows the projected trajectory of TB incidence for Ahmedabad and Panel B for Delhi, assuming current PPSA scale up between 2017 and 2020 (blue dashed line) and its maintenance indefinitely; assuming current PPSA scale up between 2017 and 2020 whilst optimistically assuming that active providers are continuously active (green line); service provisions are increased to their feasible maximum from 2020 to 2021 and maintained indefinitely, but assuming that 50% of TB cases that present to care are extra-pulmonary TB (purple line); that service provisions are increased to their feasible maximum from 2020 to 2021 and maintained indefinitely, as presented in the main text (yellow line).

For peer review only

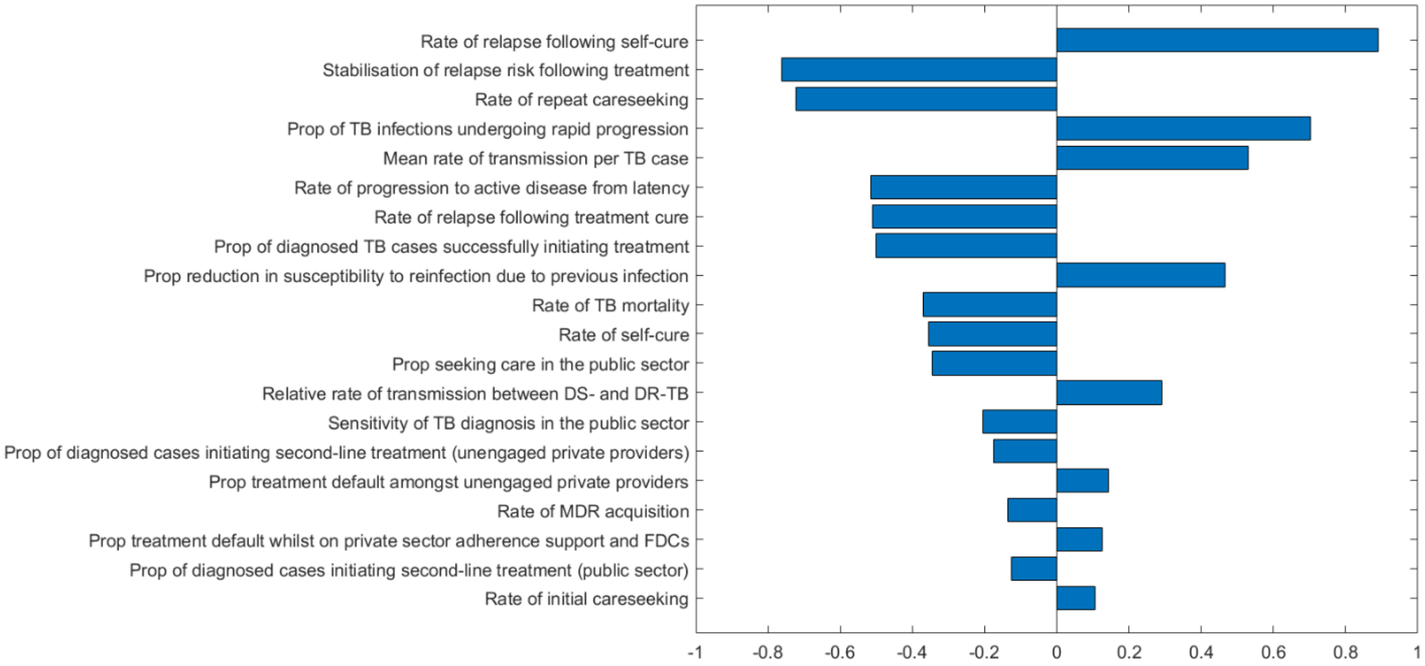

Figure S7. Multivariate sensitivity analysis of impact for Ahmedabad. We used partial rank correlation coefficient (PRCC) to examine which parameter listed in tables 2-5 the output cases averted is most sensitive towards. Larger bars represent more sensitive parameters. Shown are the 20 most influential model parameters, in decreasing order of sensitivity from top to bottom.

For peer review only

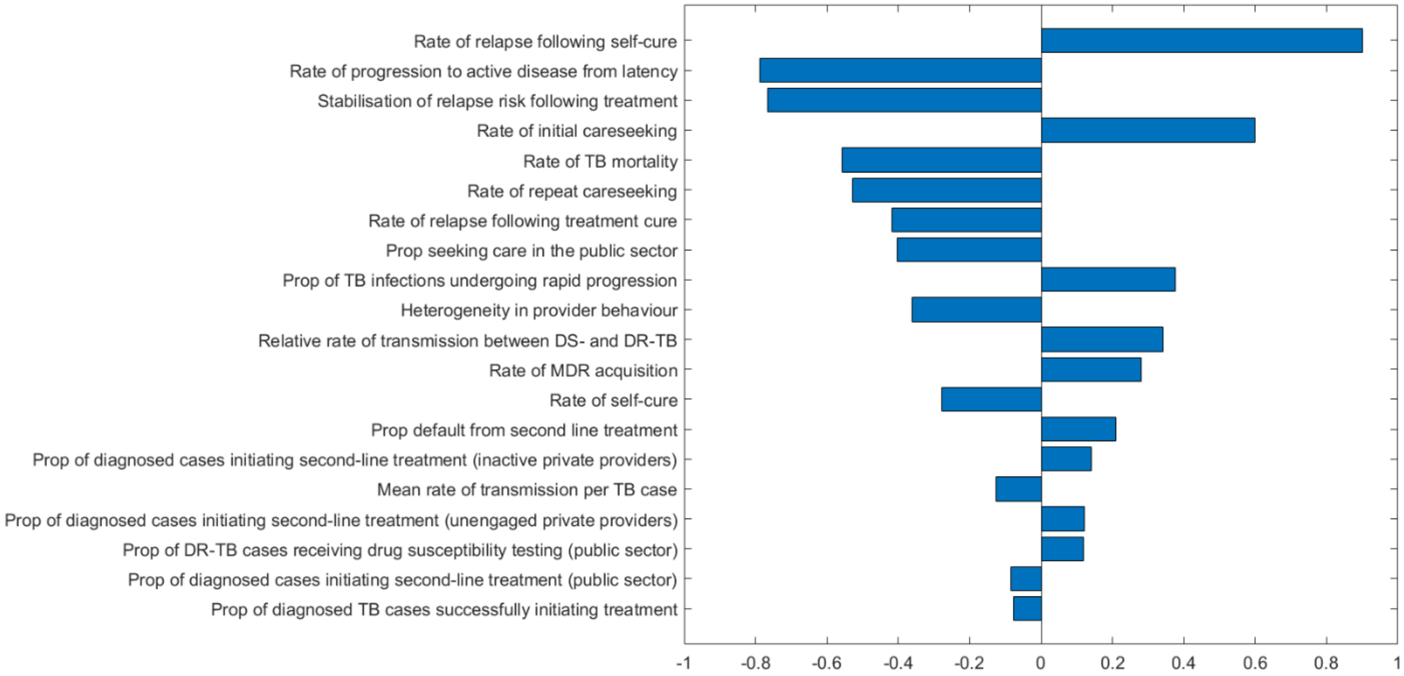

Figure S8. Multivariate sensitivity analysis of impact for Delhi. We used partial rank correlation coefficient (PRCC) to examine which parameter listed in tables 2-5 the output cases averted is most sensitive towards. Larger bars represent more sensitive parameters. Shown are the 20 most influential model parameters, in decreasing order of sensitivity from top to bottom.

3. Supporting tables

City	Number of identified private providers	Number of engaged private providers	Number of active private providers
Ahmedabad	1899	952	762
Delhi	2780	2016	894

Table S6. Number of identified, engaged, and active private providers in 2019 in Ahmedabad and Delhi. We assume that all private providers have been identified.

City	Heterogeneity index (α)
Ahmedabad	0.39 [0.32-0.46]
Delhi	0.32 [0.25-0.41]

Table S7. Heterogeneity index of private providers across Ahmedabad and Delhi. A heterogeneity index of 1 suggests that each provider sees the same number of patients (represented by the red line in fig S1). A smaller heterogeneity index indicates that a smaller number of providers are responsible for a larger number of patients. To calculate the heterogeneity, we divided the logarithm of the proportion of notifications by the logarithm of the proportion of providers responsible for those notifications.

Cost item	Total cost in 2019 (USD)	
	Ahmedabad	Delhi
Field officer salary	31,433	55,936
Hub agent salary	113,808	281,373
SCT agent salary	2055	5287
Treatment coordinator salary	8569	13,445
CMEs	460	6095
Promotional material	10	10
Sputum sample logistics	2020	5090
Xpert tests	83,886	98,088
FDCs**	N/A	

Table S8. Total costs of PPSA activities in Ahmedabad and Delhi in 2019. We assume a conversion rate of USD 1 = 73 INR. *We assume a fix cost per FDC of 45 USD.

For peer review only

Category	Description
Provider activation	Costs associated with the engagement of inactive providers to encourage them to become active and start notifying their TB cases
Provider retention	Costs associated with the engagement of active providers to encourage them to remain active and keep notifying their TB cases
Diagnostic engagement	Costs associated with encouraging active providers to start using Xpert, to increase their use of Xpert and maintain their use of Xpert
Diagnostic logistics	All other costs associated with Xpert testing that are not to do with engagement. Includes cost of transporting sputum samples, cost of staff and cost of the Xpert tests.
Treatment engagement	Costs associated with encouraging active providers to start using, increase or maintain their use of government supplied FDCs and adherence support.
Treatment logistics	Cost of government supplied FDCs and adherence support

Table S9. Costing categories. We categorise each cost item into one of the categories listed in this table to help identify the cost items that will increase as the coverage of the different interventions increase.

PPSA activity	Effort needed
Provider activation	5 (4-7) visits needed to onboard an inactive provider
Increasing Xpert usage amongst occasional users	One visit increases a provider's Xpert use by 0.2%. Assumed the same across all cities.
Converting non-Xpert users to occasionally use Xpert	7 (5-8) visits needed to convert a non-Xpert user. Assumed the same across all cities
Converting non-FDC users to use FDC	6 (4-7) visits needed to convert a non-FDC user. Assumed the same across all cities.

Table S10. Engagement effort needed for each PPSA service provision in Ahmedabad and Delhi in 2019. As described in the text, values shown in these tables are essentially expert opinion, elicited through in-dept discussion with implementing teams from CHAI.

For peer review only

Ahmedabad						
**Cost item**	**Cost category**					
	Provider activation	Provider retention	Diagnostic engagement	Diagnostic logistics	Treatment engagement	Treatment logistics
Field officer	10%	42%	24%	-	24%	-
Hub agent	-	51%	-	24%	-	25%
SCT agent	-	-	-	100%	-	-
Treatment coordinator	-	-	-	-	-	100%
CMEs	-	40%	30%	-	30%	-
Promotional material	-	52%	29%	-	19%	-
Sputum sample logistics	-	-	-	100%	-	-
Xpert tests	-	-	-	100%	-	-
FDCs	-	-	-	-	-	100%
**Delhi**						
**Cost item**	**Cost category**					
	Provider activation	Provider retention	Diagnostic engagement	Diagnostic logistics	Treatment engagement	Treatment logistics
Field officer	13%	28%	32%	-	27%	-
Hub agent	-	74%	-	18%	-	8%
SCT agent	-	-	-	100%	-	-
Treatment coordinator	-	-	-	-	-	100%
CMEs	-	40%	30%	-	30%	-
Promotional material	-	52%	29%	-	19%	-
Sputum sample logistics	-	-	-	100%	-	-
Xpert tests	-	-	-	100%	-	-
FDCs	-	-	-	-	-	100%

Table S11. Costing split across PPSA activities for Ahmedabad and Delhi in 2019. The field officer split was determined by the percentage of calls that were made by a field officer for provider activation, provider retention, diagnostic engagement and treatment engagement; similarly, the hub agent split was determined by the proportion of time hub agents estimated they had spent on provider retention, diagnostic logistics and treatment logistics; the CMEs and promotional materials splits were determined by the amount of engagement that was aimed at provider retention, diagnostic engagement and treatment engagement, which was assumed constant across all cities. Each row adds up to 100%.

For peer review only

Cost item	Total cost	Category split	Unit description	No. of units	Cost per unit (USD)
Field officer	31,433	10%	No. of visits in 2019 made to onboard an inactive provider	1548	2.08
**PPSA activity: Provider retention**					
Field officer	31,433	42%	No. of active providers in 2019	762	17.77
Hub agent	113,808	51%	No. of active providers in 2019	762	78.12
CMEs	460	40%	No. of providers that attended CMEs in 2019	51	3.70
Promotional material	10	52%	No. of active providers in 2019	762	0.007
**PPSA activity: Diagnostic engagement**					
Field officer	31,433	24%	No. of visits in 2019 made to active providers to increase Xpert usage	3572	2.17
			No. of active providers in 2019	762	10.15
CMEs	460	30%	No. of providers that attended CMEs in 2019	51	2.78
Promotional material	10	29%	No. of active providers in 2019	762	0.004
**PPSA activity: Diagnostic logistics**					
Hub agent	113,808	24%	No. of Xpert tests performed in 2019	3615	7.75
SCT agent	2055	100%	No. of Xpert tests performed in 2019	3615	0.58
Sputum sample logistics	2020	100%	No. of Xpert tests performed in 2019	3615	0.57
Xpert tests	83,886	100%	No. of Xpert tests performed in 2019	3615	23.80
**PPSA activity: Treatment engagement**					
Field officer	31,433	24%	No. of visits in 2019 made to active providers to use FDCs	3619	2.14
			No. of active providers in 2019	762	10.15
CMEs	460	30%	No. of providers that attended CMEs in 2019	51	2.78
Promotional material	10	19%	No. of active providers in 2019	762	0.003
**PPSA activity: Treatment logistics**					
Treatment coordinator	8569	100%	No. of patient treatment months in 2019	57,868	0.15
Hub agent	113,808	25%	No. of FDC initiations in 2019	3445	8.47
FDCs	-	-	-	-	45

Table S12. Unit costs for Ahmedabad

Cost item	Total cost	Category split	Unit description	No. of units	Cost per unit (USD)
**PPSA activity: Provider activation**					
Field officer	55,936	13%	No. of visits in 2019 made to onboard an inactive provider	2984	2.50
**PPSA activity: Provider retention**					
Field officer	55,936	28%	No. of active providers in 2019	894	17.97
Hub agent	281,373	74%	No. of active providers in 2019	894	238.88
CMEs	6095	40%	No. of providers that attended CMEs in 2019	1469	1.70
Promotional material	10	52%	No. of active providers in 2019	894	0.006
**PPSA activity: Diagnostic engagement**					
Field officer	55,936	32%	No. of visits in 2019 made to active providers to increase Xpert usage	7219	2.54
			No. of active providers in 2019	894	20.54
CMEs	6095	30%	No. of providers that attended CMEs in 2019	1469	1.28
Promotional material	10	29%	No. of active providers in 2019	894	0.003
**PPSA activity: Diagnostic logistics**					
Hub agent	281,373	18%	No. of Xpert tests performed in 2019	4227	12.29
SCT agent	5287	100%	No. of Xpert tests performed in 2019	4227	1.28
Sputum sample logistics	5090	100%	No. of Xpert tests performed in 2019	4227	1.23
Xpert tests	98,088	100%	No. of Xpert tests performed in 2019	4227	23.80
**PPSA activity: Treatment engagement**					
Field officer	55,936	27%	No. of visits in 2019 made to active providers to use FDCs	5863	2.64
			No. of active providers in 2019	894	17.33
CMEs	6095	30%	No. of providers that attended CMEs in 2019	1469	1.28
Promotional material	10	19%	No. of active providers in 2019	894	0.002
**PPSA activity: Treatment logistics**					
Treatment coordinator	13,445	100%	No. of patient treatment months in 2019	108,915	0.13
Hub agent	281,373	8%	No. of FDC initiations in 2019	1480	13.65
FDCs	-	-	-	-	45

Table S13. Unit costs for Delhi.

Intervention	Incremental cost (2020-2035, USD)	Percentage of cumulative TB cases averted (2020-2035)	Cost-efficiency
**Individual interventions**			
Increasing the number of active providers (assuming newly active providers adopt average behaviour)	2,269,300 (1,477,000 – 3,723,000)	3.00% (2.59-3.37)	0.0032 (0.0019- 0.0050)
Increasing the number of active providers (assuming newly active providers' behaviour is 25% worse than average)	2,224,400 (1,435,200 – 3,672,300)	2.98% (2.58-3.35)	0.0032 (0.0019- 0.0051)
Increasing the number of active providers (assuming newly active providers' behaviour is 50% worse than average)	2,120,000 (1,339,000 – 3,555,300)	2.89% (2.48-3.24)	0.0033 (0.0019- 0.0053)
Increasing Xpert use amongst occasional users	9,005,000 (6,801,000 – 13,190,000)	2.83% (2.42-3.33)	0.0008 (0.0006- 0.0010)
Increasing the number of occasional Xpert users	2,211,700 (1,683,500 – 3,275,100)	2.32% (1.97-2.67)	0.0025 (0.0018- 0.0033)
Increasing the number of FDC users	1,787,700 (1,438,900 – 2,503,800)	2.53% (2.13-2.91)	0.0031 (0.0024- 0.0041)
**Combination of the two most cost-efficient interventions: Increasing the number of active providers* + …**			
+Increasing the number of occasional Xpert users	5,020,300 (3,590,000 – 7,512,900)	3.21% (2.84-3.61)	0.0015 (0.0010- 0.0022)
+ Increasing Xpert use amongst occasional users	13,427,000 (10,019,000 – 19,839,000)	3.81% (3.40-4.45)	0.0007 (0.0005- 0.0009)
+ Increasing the number of FDC users	5,217,100 (3,791,600 – 8,182,300)	3.46% (2.96-3.91)	0.0016 (0.0010- 0.0022)
**Combination of the three most cost-efficient interventions: Increasing the number of active providers* + the number of FDC-users + …**			
+Increasing the number of occasional Xpert users	7,163,000 (5,671,000 – 10,478,000)	3.67% (3.18-4.16)	0.0012 (0.0008- 0.0017)
+ Increasing Xpert use amongst occasional users	15,467,000 (11,921,000 – 22,411,000)	4.27% (3.81-4.87)	0.0007 (0.0005- 0.0009)
**Combination of all interventions***			
+ Increasing Xpert use amongst occasional users	27,976,000 (21,331,000 – 40,913,000)	5.17% (4.49-6.17)	N/A

Table S14. Cost efficiency of service provisions in Ahmedabad. We also conducted a sensitivity analysis on the behaviour of newly active providers, and whether increasing the number of active providers remains the most cost-efficient individual intervention if the behaviour of newly active providers is assumed to be 25% and 50% worse than pre-existing active providers. Indeed, even if the behaviour of newly active providers is 50% worse, this intervention is still the most cost-efficient individual intervention when scaled up to its feasible maximum. *We assume that these newly active providers adopt average behaviour.

For peer review only - http://bmjopen.bmj.com/site/about/guidelines.xhtml

Intervention	Incremental cost (2020-2035, USD)	Percentage of cumulative TB cases averted (2020-2035)	Cost-efficiency
**Individual interventions**			
Increasing the number of active providers (assuming newly active providers adopt average behaviour)	20,760,000 (12,478,000 – 36,094,000)	3.06% (2.43-3.98)	0.0003 (0.0002- 0.0006)
Increasing Xpert use amongst occasional users	22,249,000 (13,546,000 – 30,724,000)	1.11% (0.97-1.27)	0.0001 (0.0001- 0.0002)
Increasing the number of occasional Xpert users	4,920,900 (2,985,500 – 6,819,600)	0.86% (0.73-1.03)	0.0004 (0.0003- 0.0006)
Increasing the number of FDC users	4,496,400 (3,283,400 – 5,866,200)	1.13% (0.97-1.34)	0.0006 (0.0004- 0.0007)
**Combination of the two most cost-efficient interventions: Increasing the number of FDC-users + …**			
+Increasing the number of active providers	30,981,000 (21,744,000-46,622,000)	3.80% (2.92-5.06)	0.00026 (0.00017- 0.0046)
+Increasing the number of occasional Xpert users	9,250,000 (6,796,000 – 12,272,000)	1.21% (1.05-1.41)	0.00028 (0.00021-0.00038)
+ Increasing Xpert use amongst occasional users	26,794,000 (17,716,000 – 36,310,000)	1.47% (1.29-1.72)	0.00012 (0.00008-0.00017)
**Combination of the three most cost-efficient interventions: Increasing the number of FDC users + increasing the number of occasional Xpert users + …**			
+ Increasing the number of active providers	41,648,000 (30,441,000 - 60,488,000)	3.95% (3.05-5.22)	0.00020 (0.00013 – 0.00033)
+ Increasing Xpert use amongst occasional users	60,238,000 (37,969,000 – 82,081,000)	1.96% (1.64-2.35)	0.00007 (0.00005 – 0.00010)
**Combination of all interventions**			
+ Increasing Xpert use amongst occasional users	157,450,000 (100,600,000 – 213,200,000)	5.35% (4.30-7.00)	N/A

Table S15. Cost efficiency of service provisions in Delhi. *We assume that these newly active providers adopt average behaviour.

For peer review only

Cost category	Baseline	Increasing no. of active providers	Increasing Xpert usage amongst occasional Xpert users	Increasing the no. of occasional Xpert users	Increasing the no. of providers offering FDCs
**Provider retention**	1.2 million	1.5 million	1.2 million	1.2 million	1.2 million
**Onboarding engagement**	N/A	2,000	N/A	N/A	N/A
**Xpert engagement**	12,000	15,000	0.3 million	14,000	12,000
**FDC engagement**	11,000	14,000	11,000	11,000	17,000
**Xpert logistics**	6.0 million (4.6 – 8.9 million)	7.6 million (5.7 – 11.0 million)	15.0 million (12.2 – 23.8 million)	8.5 million (6.4 – 12.7 million)	6.0 million (4.7 – 8.9 million)
**Adherence support**	0.1 million (0.1 – 0.2 million)	0.2 million (0.1 – 0.3 million)	0.1 million (0.1 – 0.2 million)	0.1 million (0.1 – 0.2 million)	0.1 million (0.1 – 0.2 million)
**FDCs**	5.5 million (3.2 – 9.0 million)	6.7 million (4.1 – 11.0 million)	4.3 million (2.7 – 7.0 million)	5.2 million (3.1 – 8.4 million)	7.4 million (4.9 – 11.6 million)
**Public sector diagnoses**	35.0 million (26.6 – 50.4 million)	35.0 million (25.2 – 50.4 million)	35.0 million (25.2 – 50.4 million)	35.0 million (26.6 – 50.4 million)	35.0 million (26.6 – 50.4 million)
**Public sector treatment**	68.6 million (53.2– 93.8 million)	68.6 million (53.2– 92.4 million)	68.6 million (53.2– 93.8 million)	68.6 million (53.2– 93.8 million)	68.6 million (53.2– 93.8 million)

Table S16. Breakdown of total costs across each intervention between 2020 and 2035 for Ahmedabad.

For peer review only

Cost category	Baseline	Increasing no. of active providers	Increasing Xpert usage amongst occasional Xpert users	Increasing the no. of occasional Xpert users	Increasing the no. of providers offering FDCs
**Provider retention**	3.5 million	8.0 million	3.5 million	3.5 million	3.5 million
**Onboarding engagement**	N/A	14,000	N/A	N/A	N/A
**Xpert engagement**	22,000	50,000	0.3 million	32,000	22,000
**FDC engagement**	18,000	41,000	18,000	18,000	32,000
**Xpert logistics**	8.1 million (4.9 – 11.0 million)	18.0 million (11.2 – 25.2 million)	32.2 million (19.6 – 44.8 million)	13.5 million (8.3 – 18.2 million)	8.1 million (4.9 – 11.0 million)
**Adherence support**	0.3 million (0.2 – 0.4 million)	0.6 million (0.3 – 0.9 million)	0.2 million (0.1 – 0.4 million)	0.3 million (0.2 – 0.4 million)	0.3 million (0.2 – 0.4 million)
**FDCs**	10.2 million (5.3 – 19.6 million)	23.8 million (11.9 – 44.8 million)	7.7 million (4.2 – 15.4 million)	9.7 million (5.0 – 19.6 million)	15.4 million (9.8 – 25.2 million)
**Public sector diagnoses**	117.6 million (74.2 – 154 million)	116.2 million (72.8 – 154 million)	116.2 million (72.8 – 154 million)	117.6 million (74.2 – 154 million)	117.6 million (74.2 – 154 million)
**Public sector treatment**	224 million (168 million – 294 million)	224 million (168 million – 294 million)	224 million (168 million – 294 million)	224 million (168 million – 294 million)	224 million (168 million – 294 million)

Table S17. Breakdown of total costs across each intervention between 2020 and 2035 for Delhi.

For peer review only

Intervention	Maintenance costs sensitivity analysis	Incremental cost (2020-2035, USD)	Percentage of cumulative TB cases averted (2020-2035)	Cost-efficiency
Increasing the number of active providers	*-20%:*	1,963,400 (1,170,200-3,416,300)	3.00% (2.59-3.37)	0.0037 (0.0021-0.0063)
	+20%	2,575,300 (1,782,200-4,028,200)		0.0028 (0.0017-0.0041)
Increasing the number of occasional Xpert users	*-20%:*	1,965,900 (1,440,400-3,030,200)	2.32% (1.97-2.67)	0.0028 (0.0020-0.0038)
	+20%	2,455,700 (1,930,200-3,520,000)		0.0023 (0.0017-0.0029)
Increasing Xpert use amongst occasional users	*-20%:*	8,743,000 (6,671,000-13,031,000)	2.83% (2.42-3.33)	0.0008 (0.0006-0.0010)
	+20%	9,232,000 (7,160,000-13,521,000)		0.0007 (0.0006-0.0009)
Increasing the number of FDC users	*-20%:*	1,541,800 (1,194,000-2,260,900)	2.53% (2.13-2.91)	0.0039 (0.0026-0.0050)
	+20%	2,031,600 (1,683,800-2,750,700)		0.0027 (0.0022-0.0036)

Table S18. Sensitivity analysis on the cost of maintenance in Ahmedabad. We explored whether increasing or decreasing maintenance cost by 20% effected the cost-efficiency order of individual service provisions. Maintenance costs include the cost of provider retention, the cost of Xpert engagement to maintain Xpert use, and the cost of FDC engagement to maintain FDC use.

For peer review only

Intervention	Maintenance costs sensitivity analysis	Incremental cost (2020-2035, USD)	Percentage of cumulative TB cases averted (2020-2035)	Cost-efficiency
Increasing the number of active providers	*-20%:*	19,145,000 (10,865,000 – 34,481,000)	3.06% (2.43-3.98)	0.0003 (0.0002-0.0007)
	+20%	22,375,000 (14,095,000 – 37,712,000)		0.0003 (0.0002 – 0.0006)
Increasing the number of occasional Xpert users	*-20%:*	4,205,900 (2,272,200 – 6,104,300)	0.86% (0.73-1.03)	0.0004 (0.0003-0.0008)
	+20%	5,638,400 (3,704,700 – 7,536,800)		0.0003 (0.0002-0.0005)
Increasing Xpert use amongst occasional users	*-20%:*	21,455,000 (12,823,000 – 29,889,000)	1.11% (0.97-1.27)	0.0001 (0.0001-0.0002)
	+20%	22,887,000 (14,255,000– 31,321,000)		0.0001 (0.0001-0.0002)
Increasing the number of FDC users	*-20%:*	3,782,600 (2,569,100 – 5,154,300)	1.13% (0.97-1.34)	0.0007 (0.0005-0.0009)
	+20%	5,215,100 (4,001,600 – 6,586,800)		0.0005 (0.0004-0.0006)

Table S19. Sensitivity analysis on the cost of maintenance in Delhi. We explored whether increasing or decreasing maintenance cost by 20% effected the cost-efficiency order of individual service provisions. Maintenance costs include the cost of provider retention, the cost of Xpert engagement to maintain Xpert use, and the cost of FDC engagement to maintain FDC use.

For peer review only

4. Bibliography

1. Mistry N, Lobo E, Shah S, Rangan S, Dholakia Y. Pulmonary Tuberculosis in Patna, India: Durations, Delays, and Health Care Seeking Behaviour Among Patients Identified Through Household Surveys. *Journal of Epidemiology and Global Health*. 2017;7(4): 241–248.

2. Veesa KS, John KR, Moonan PK, Kaliappan SP, Manjunath K, Sagili K, et al. Diagnostic Pathways and Direct Medical Costs Incurred by New Adult Pulmonary Tuberculosis Patients Prior to Anti-Tuberculosis Treatment - Tamil Nadu, India. *PLOS ONE*. 2018;13(2): e0191591.

3. Pandey A, Ploubidis GB, Clarke L, Dandona L. Hospitalisation Trends in India From Serial Cross-Sectional Nationwide Surveys: 1995 to 2014. *BMJ Open*. 2017;7(12): e014188.

4. Sachdeva KS, Satyanarayana S, Dewan P, Nair SA, Reddy R, Kundu D, et al. Source of Previous Treatment for Re-Treatment TB Cases Registered under the National TB Control Programme, India, 2010. *PLOS ONE*. 2011;6(7): e22061.

5. Satyanarayana S, Nair SA, Chadha SS, Shivashankar R, Sharma G, Yadav S, et al. From Where Are Tuberculosis Patients Accessing Treatment in India? Results From a Cross-Sectional Community Based Survey of 30 Districts. *PLOS ONE*. 2011;6(9): e24160.

6. Sharma G, Sinha A, Bastian S, Santha T. Knowledge and behaviour of chest symptomatics in urban slum populations of two states in India towards care-seeking. *The Indian Journal of Tuberculosis*. 2013;60(2): 95–106.

7. Satyanarayana S, Subbaraman R, Shete P, Gore G, Das J, Cattamanchi A, et al. Quality of Tuberculosis Care in India: A Systematic Review. *International Journal of Tuberculosis and Lung Disease*. 2015;19(7): 751–763.

8. World Health Organisation. *A brief history of tuberculosis control in India.* [Online] World Health Organisation, 2010. Available from: https://apps.who.int/iris/bitstream/handle/10665/44408/9789241500159_eng.pdf?sequence=1

9. Subbaraman R, Nathavitharana RR, Satyanarayana S, Pai M, Thomas BE, Chadha VK, et al. The Tuberculosis Cascade of Care in India's Public Sector: A Systematic Review and Meta-analysis. *PLOS Medicine*. [Online] 2016;13(10): e1002149. Available from: doi:10.1371/journal.pmed.1002149

10. World Health Organisation. *Global Tuberculosis Report 2020*. [Online] World Health Organisation, 2020. Available from: https://www.who.int/publications/i/item/9789240013131

11. Menzies D, Benedetti A, Paydar A, Martin I, Royce S, Pai M, et al. Effect of Duration and Intermittency of Rifampin on Tuberculosis Treatment Outcomes: A Systematic Review and Meta-Analysis. *PLOS Med*. [Online] 2009;6(9): e1000146. Available from: doi:10.1371/journal.pmed.1000146

12. Houben RMGJ, Lalli M, Kranzer K, Menzies NA, Schumacher SG, Dowdy DW. What if they don't have tuberculosis? The consequences and trade-offs involved in false-positive diagnoses of tuberculosis. *Clinical Infectious Diseases*. [Online] 2019;68(1): 150–156. Available from: doi:10.1093/cid/ciy544

13. Fochsen G, Deshpande K, Diwan V, Mishra A, Diwan VK, Thorson A. Health care seeking among individuals with cough and tuberculosis: a population-based study from rural India. *International Journal of Tuberculosis and Lung Disease*. 2006;10(9): 995–1000.

14. Gopi PG, Subramani R, Narayanan PR. Evaluation of different types of chest symptoms for diagnosing pulmonary tuberculosis cases in community surveys. *Indian Journal of Tuberculosis*. 2008;55(3): 116–121.

15. Rao VG, Bhat J, Yadav R, Gopalan GP, Nagamiah S, Bhondeley MK, et al. Prevalence of pulmonary tuberculosis - a baseline survey in central India. *PLOS One*. [Online] 2012;7(8): e43225. Available from: doi:10.1371/journal.pone.0043225

For peer review only

16. Sachdeva KS, Raizada N, Sreenivas A, Van't Hoog A, van den Hof S, Dewan PK, et al. Use of Xpert MTB/RIF in decentralized public health settings and its effect on pulmonary TB and DR-TB case findings in India. *PLOS ONE*. [Online] 2015;10(5): e0126065. Available from: doi:10.1371/journal.pone.0126065

17. Haario H, Saksman E, Tamminen J. An adaptive metropolis algorithm. *Bernoulli*. 2001;7(2): 223–242.

For peer review only

CHEERS 2022 Checklist

	Item	Guidance for Reporting	Reported in section
**TITLE**			
Title	1	Identify the study as an economic evaluation and specify the interventions being compared.	N/A
**ABSTRACT**			
Abstract	2	Provide a structured summary that highlights context, key methods, results and alternative analyses.	Page 1
**INTRODUCTION**			
Background and objectives	3	Give the context for the study, the study question and its practical relevance for decision making in policy or practice.	Page, 1-2
**METHODS**			
Health economic analysis plan	4	Indicate whether a health economic analysis plan was developed and where available.	Page 5-6
Study population	5	Describe characteristics of the study population (such as age range, demographics, socioeconomic, or clinical characteristics).	Table 1-2
Setting and location	6	Provide relevant contextual information that may influence findings.	Table 1and page 3-4
Comparators	7	Describe the interventions or strategies being compared and why chosen.	Page 6
Perspective	8	State the perspective(s) adopted by the study and why chosen.	Page 5
Time horizon	9	State the time horizon for the study and why appropriate.	Page 5
Discount rate	10	Report the discount rate(s) and reason chosen.	Page 6
Selection of outcomes	11	Describe what outcomes were used as the measure(s) of benefit(s) and harm(s).	Page 6
Measurement of outcomes	12	Describe how outcomes used to capture benefit(s) and harm(s) were measured.	Page 6
Valuation of outcomes	13	Describe the population and methods used to measure and value outcomes.	N/A
Measurement and valuation of resources and costs	14	Describe how costs were valued.	Table S8-13
Currency, price date, and conversion	15	Report the dates of the estimated resource quantities and unit costs, plus the currency and year of conversion.	Table S8
Rationale and description of model	16	If modelling is used, describe in detail and why used. Report if the model is publicly available and where it can be accessed.	Model is fully specified in the manuscript materials
Analytics and assumptions	17	Describe any methods for analysing or statistically transforming data, any extrapolation methods, and approaches for validating any model used.	Figure S1, table S10, page 6
Characterizing heterogeneity	18	Describe any methods used for estimating how the results of the study vary for sub-groups.	Page 6-7
Characterizing distributional effects	19	Describe how impacts are distributed across different individuals or adjustments made to reflect priority populations.	N/A
Characterizing uncertainty	20	Describe methods to characterize any sources of uncertainty in the analysis.	Page 6-7
Approach to engagement with patients and others affected by the study	21	Describe any approaches to engage patients or service recipients, the general public, communities, or stakeholders (e.g., clinicians or payers) in the design of the study.	N/A
**RESULTS**			
Study parameters	22	Report all analytic inputs (e.g., values, ranges, references) including uncertainty or distributional assumptions.	Table 1-2
Summary of main results	23	Report the mean values for the main categories of costs and outcomes of interest and summarise them in the most appropriate overall measure.	Table 3, S14-15
Effect of uncertainty	24	Describe how uncertainty about analytic judgments, inputs, or projections affect findings. Report the effect of choice of discount rate and time horizon, if applicable.	Page 9-10, fig S6-8
Effect of engagement with patients and others affected by the study	25	Report on any difference patient/service recipient, general public, community, or stakeholder involvement made to the approach or findings of the study	N/A
**DISCUSSION**			
Study findings, limitations, generalizability, and current knowledge	26	Report key findings, limitations, ethical or equity considerations not captured, and how these could impact patients, policy, or practice.	Page 8
**OTHER RELEVANT INFORMATION**			
Source of funding	27	Describe how the study was funded and any role of the funder in the identification, design, conduct, and reporting of the analysis	
Conflicts of interest	28	Report authors conflicts of interest according to journal or International Committee of Medical Journal Editors requirements.	

Husereau D, Drummond M, Augustovski F, de Bekker-Grob E, Briggs AH, Carswell C, Caulley L, Chaiyakunapruk N, Greenberg D, Loder E, Mauskopf J, Mullins CD, Petrou S, Pwu RF, Staniszewska S; CHEERS 2022 ISPOR Good Research Practices Task Force. Consolidated Health Economic Evaluation Reporting Standards 2022 (CHEERS 2022) Statement: Updated Reporting Guidance for Health Economic Evaluations. BMJ. 2022;376:e067975.
The checklist is Open Access distributed in accordance with the terms of the Creative Commons Attribution (CC BY 4.0) license, which permits others to distribute, remix, adapt and build upon this work, for commercial use, provided the original work is properly cited. See: http://creativecommons.org/licenses/by/4.0/.

For peer review only

BMJ Open

Operational priorities for engaging with India’s private healthcare sector for the control of tuberculosis: a modelling study

Journal:	*BMJ Open*
Manuscript ID	bmjopen-2022-069304.R1
Article Type:	Original research
Date Submitted by the Author:	23-May-2023
Complete List of Authors:	Ricks, Saskia ; Imperial College London,<br>Singh, Ananya; Clinton Health Access Initiative<br>Sodhi, Ridhima; Clinton Health Access Initiative<br>Pal, Arnab; Clinton Health Access Initiative<br>Arinaminpathy, Nimalan; Imperial College London School of Public Health
**Primary Subject Heading**:	Epidemiology
Secondary Subject Heading:	Health economics
Keywords:	Tuberculosis < INFECTIOUS DISEASES, Epidemiology < INFECTIOUS DISEASES, HEALTH ECONOMICS

SCHOLARONE™
Manuscripts

I, the Submitting Author has the right to grant and does grant on behalf of all authors of the Work (as defined in the below author licence), an exclusive licence and/or a non-exclusive licence for contributions from authors who are: i) UK Crown employees; ii) where BMJ has agreed a CC-BY licence shall apply, and/or iii) in accordance with the terms applicable for US Federal Government officers or employees acting as part of their official duties; on a worldwide, perpetual, irrevocable, royalty-free basis to BMJ Publishing Group Ltd ("BMJ") its licensees and where the relevant Journal is co-owned by BMJ to the co-owners of the Journal, to publish the Work in this journal and any other BMJ products and to exploit all rights, as set out in our licence.

The Submitting Author accepts and understands that any supply made under these terms is made by BMJ to the Submitting Author unless you are acting as an employee on behalf of your employer or a postgraduate student of an affiliated institution which is paying any applicable article publishing charge ("APC") for Open Access articles. Where the Submitting Author wishes to make the Work available on an Open Access basis (and intends to pay the relevant APC), the terms of reuse of such Open Access shall be governed by a Creative Commons licence – details of these licences and which Creative Commons licence will apply to this Work are set out in our licence referred to above.

Other than as permitted in any relevant BMJ Author's Self Archiving Policies, I confirm this Work has not been accepted for publication elsewhere, is not being considered for publication elsewhere and does not duplicate material already published. I confirm all authors consent to publication of this Work and authorise the granting of this licence.

For peer review only

Operational priorities for engaging with India's private healthcare sector for the control of tuberculosis: a modelling study

Saskia Ricks*[1], Ananya Singh[2], Ridhima Sodhi[2], Arnab Pal[2] and Nimalan Arinaminpathy[1]

*Corresponding author; saskia.ricks12@imperial.ac.uk

[1]Imperial College London, Praed Street, London, W2 1NY, UK

[2]Clinton Health Access Initiative, New Delhi, India

For peer review only

Key words: tuberculosis, epidemiology, health economics

Word count: 4670

For peer review only - http://bmjopen.bmj.com/site/about/guidelines.xhtml

Abstract

Objectives: The Joint Effort for Elimination of Tuberculosis ("JEET") is the largest private sector engagement initiative for tuberculosis (TB) in India. Considering the potential impact of such an intervention, it is important to anticipate how the different services offered by JEET should be expanded in the future.

Design: We developed a mathematical model of TB transmission dynamics, coupled with a cost model.

Setting: Ahmedabad and New Delhi, two cities with contrasting levels of JEET coverage.

Participants: Estimated patients with TB in Ahmedabad and New Delhi.

Interventions: We investigated the epidemiological impact of expanding three different PPSA services: provider recruitment, uptake of CB-NAAT (cartridge-based nucleic acid amplification tests) and uptake of adherence support mechanisms (specifically government supplied fixed-dose combination drugs), all compared to a continuation of current TB services.

Results: Our results suggest that in Delhi, increasing the use of adherence support mechanisms amongst private providers should be prioritised, having the lowest incremental cost-per-case-averted between 2020 and 2035 of $170,000 (110,000 – 310,000). Likewise in Ahmedabad, increasing provider recruitment should be prioritised, having the lowest incremental cost-per-case averted of $18,000 (12,000-29,000).

Conclusion: Results illustrate how intervention priorities may vary in different settings across India, depending on local conditions, and the existing degree of uptake of PPSA services. Modelling can be a useful tool for identifying these priorities for any given setting.

Strengths and limitations

- We have used detailed programmatic data to inform costs, effectiveness, and real-world coverage of the different interventions discussed in this analysis to help identify operational priorities.
- However, due to a lack of city-specific data for Delhi and Ahmedabad, the mathematical model was instead calibrated to prevalence and infection surveys from Chennai, another large Indian city.
- Assumptions were also made to account for healthcare provider behaviour.

For peer review only

Introduction

Tuberculosis (TB) is an airborne infectious disease (1) and a major cause of death worldwide, responsible for 10.6 million cases and 1.6 million deaths in 2021 (2). India accounts for over a quarter of global TB burden, with 2.9 million cases in 2021 (2). A major challenge in India is the presence of a vast, fragmented private healthcare sector, which manages the majority of TB patients in the country (3–5). The past decade has seen increasing recognition of the importance of engaging effectively with this sector (6–10): not only in notifying TB to public health authorities, but also in offering high-quality diagnostics and treatment adherence support for patients being managed by private providers. Early efforts to engage effectively with the private sector faced substantial challenges, principally arising from a lack of trust (11). Despite TB being made a notifiable disease in 2012 in India (12) few private providers reported their TB patients to public health authorities. However, from 2013 onwards, pilots of 'public-private support agencies' (PPSAs) showed how private providers could be successfully engaged, through supporting private providers to notify TB, as well as making high-quality diagnostics and treatment support available for patients being managed by the private sector (7,9).

To take the PPSA model to scale across the country, the Joint Effort for Elimination of Tuberculosis (JEET) was launched by 3 partner organisations (the Clinton Health Access Initiative; Centre for Health, Research and Innovation; and Foundation for Innovative New Diagnostics) in 2018 (13). Supported by the Global Fund and coordinated by the Central TB Division of India, it is the largest private sector engagement initiative for TB in India to date (14). The main activities include increasing case notifications by engaging with private providers, improving the quality of TB diagnosis by providing free access to molecular diagnostics, and improving treatment outcomes by providing free fixed dose combination (FDCs) and adherence support (13). By, 2020 JEET was operating in 485 cities across India (13); these developments accompanied a substantial increase in notifications from private providers, accounting in 2022 for around 30% of total TB notifications in India (15).

Despite these achievements, there remains much ground to be covered. Moreover, progress in PPSA implementation has varied across the country, with greater uptake of available services in some cities than in others. One important question is what kinds of PPSA services should be promoted in any given setting, depending on what levels of uptake have already been achieved. For example, in a given city, should future efforts for strengthening PPSA efforts place greater priority on increasing the uptake of molecular diagnostics, or of adherence support mechanisms? Addressing such questions can benefit from an understanding of the potential epidemiological impact of different strategies, along with projected costs and cost-effectiveness. Here, we aimed to meet this need using mathematical models of TB transmission, to identify the most cost-effective strategies for future PPSA expansion.

For peer review only - http://bmjopen.bmj.com/site/about/guidelines.xhtml

Method

Model overview

We focused on two urban settings, Ahmedabad and Delhi, with contrasting levels of uptake of PPSA services. Ahmedabad has population of around 7.7 million and is the largest city in the Indian state of Gujarat; Delhi has a population of around 33 million and is the capital city of the country. PPSA operations in both cities began in 2018 (16), and have followed different trajectories with, for example, services in Delhi seeing substantially less uptake of fixed dose combinations (FDCs) than Ahmedabad. In the present analysis, therefore, they provide helpful examples of sites that may face different priorities in their future expansion of PPSA efforts.

We developed a deterministic, compartmental model of TB transmission amongst adults (>15 years old) consistent with an urban setting in India (fig 1). The model captures routine TB services, including the scale-up of the Revised National TB Control Programme (RNTCP) between 1997 and 2007 (17), and the scale-up of private sector engagement from 2017 onwards (18). We incorporated the acquisition and transmission of drug-resistant TB (DR-TB), including both rifampicin-resistant and multi-drug resistant TB. For simplicity, we ignored HIV status, and the distinction between pulmonary and extrapulmonary TB, although we conducted a sensitivity analysis on the latter. We separately simulated a non-TB symptomatic population (fig 1) to count the number of TB diagnoses and false positive treatments amongst those with symptoms suggestive of TB, but without TB infection. We calibrated the size of the non-TB symptomatic population to give a 10% prevalence of TB amongst those presenting for care, in either the public or private sector (19–21). The supplementary information provides further details on the model, including governing equations and parameter values (table S1-table S7), and a breakdown of the proportion of patients visiting different providers for diagnosis and treatment (table S8- table S9, respectively).

Data sources

To reflect a typical urban setting in India (such as Ahmedabad and Delhi), we used survey data for the annual risk of TB infection (ARTI) and the prevalence of TB (22,23). Table 1 summarises the data from these sources. Table S2- table S5 list parameter values used in the model (TB natural history, diagnosis, treatment and costs, respectively).

For peer review only - http://bmjopen.bmj.com/site/about/guidelines.xhtml

Indicator	Value	Source
Annual risk of TB infection	2% [1-3]	(22)
TB prevalence	259 per 100,000 [217-299]	(23)
TB mortality	32 per 100,000 [30-34]	(24)
Percent of TB cases that are MDR	4% [3-5]	(24)

Table 1. Calibration targets used to estimate model parameters. We assumed the same prevalence, annual risk of TB infection, TB mortality and percent of TB cases that are MDR across Ahmedabad and Delhi.

We incorporated uncertainty in parameter values and model inputs through Adaptive Bayesian Markov chain Monte Carlo (MCMC) (25). Thus, the uncertainty in model projections reflects the uncertainty in the model inputs; we refer to the uncertainty in model projections as the Bayesian credible intervals, using the 2.5th and 97.5th percentiles to reflect the lower and upper bound of an interval.

We also drew a range of operational data from CHAI, to inform elements such as existing uptake of JEET services in Ahmedabad and Delhi; provider behaviour over time in response to CHAI engagement efforts; and treatment outcomes under different types of adherence support in the private sector. Further details are provided below ('Modelling existing PPSA services').

Care cascade

We assumed that upon development of active disease, there is a delay (determined by calibration to the available data) before individuals seek care (10,26). We modelled public and private providers separately, assuming: (i) the quality of diagnosis to be lower in the latter than the former, and (ii) once individuals are diagnosed with TB and initiated on treatment, a lower rate of treatment completion in the private sector, due to the general lack of adherence support (27,28). Owing to a lack of systematic data for the private sector when not engaged with a PPSA, we allowed for a wide range of parameter values relating to both diagnosis and treatment in this sector (table S3- table S4).

Modelling existing PPSA services

Provider recruitment

When commencing operations in any new setting, JEET conducts provider mapping to identify the highest-volume providers that should be prioritised. 'Engagement' with providers is achieved by field officers to encourage providers to notify their TB cases. However, even after these efforts, not all engaged providers notify TB, and require further engagement by field officers to do so, coupled with a strong enforcement of regulations. Accordingly, we distinguished different types of private providers: those who are not engaged; those who are engaged but not notifying TB ('inactive' providers); and those who are notifying TB ('active' providers) (table S10). We define 'active' providers as those notifying TB at least once a month. Active providers may, over time, turn

For peer review only - http://bmjopen.bmj.com/site/about/guidelines.xhtml

inactive, for three main reasons: first, some providers may not have a sufficient number of TB patients every month and thus may notify infrequently depending on when they see a new TB patient; second, some providers, especially those that handle a smaller volume of TB patients, are seen less regularly by the field team and are therefore less likely to notify; and third, some providers may be dissatisfied with the services offered by JEET, and therefore intentionally stop notifying patients. If 'active' providers are defined as those notifying TB at least once a month, we accounted for this rate of attrition by weighting the amount of CB-NAAT (cartridge-based nucleic acid amplification testing) and FDC issued by the number of months providers are active for.

The transmission model follows the patient perspective of the careseeking journey; an important parameter relating to PPSA activities is the probability that a patient, when seeking care for their symptoms, visits a private provider amongst the three categories listed above. To estimate these probabilities, we modelled a 'heterogeneity index' α, such that (fig S1, table S11):

$$\text{Proportion of patients visiting an engaged provider} = (\text{Proportion of providers engaged})^{\alpha}$$

Thus, values of $\alpha < 1$ correspond with the situation where high-volume providers are preferentially engaged. We estimated the value of α using data provided by CHAI for both Ahmedabad and Delhi.

TB diagnosis

Field officers engage with active providers to encourage them to offer CB-NAAT to their presumptive TB patients. Specimen collection and transportation (SCT) agents collect the sputum samples from the provider clinic and transport them to laboratories where CB-NAAT testing is performed for free. While efforts are made to encourage active providers to use these services, the services are not mandated under Project JEET, in the interest of a collaborative relationship.

There is a multimodal distribution for the uptake of this CB-NAAT service amongst active private providers, including those who never use it; those who use it occasionally; and those for whom all notifications are CB-NAAT confirmed (fig S2). In 2019, 55% and 66% of active providers in Ahmedabad and Delhi did not use CB-NAAT confirmation when notifying TB. Providers who do not confirm a TB notification with CB-NAAT instead evaluate patients using chest x-ray and clinical judgement (18,19,29). We assumed that inactive and unengaged private providers diagnose TB in the same way. We drew from the literature for the sensitivity and specificity of X-ray and clinical judgement in diagnosing TB (30,31) (table S3).

Motivated by fig S2, we divided active providers into three categories depending on their use of CB-NAAT: providers who always confirm TB notifications with CB-NAAT, providers who occasionally use CB-NAAT, and providers who never use CB-NAAT. Table 2 shows the number of active providers in each of these categories for Ahmedabad and Delhi in 2019. Once active providers become an CB-NAAT user, field officers continue visits to encourage these providers to continue confirming TB diagnoses with CB-NAAT.

For peer review only - http://bmjopen.bmj.com/site/about/guidelines.xhtml

	City	
	Ahmedabad	*Delhi*
Total no. of active providers	762	894
No. of active providers that always use CB-NAAT	65	35
No. of active providers that occasionally use CB-NAAT	278	265
No. of active providers that never use CB-NAAT	419	594
Percent of notifications that are CB-NAAT confirmed by occasional users	22%	15%

Table 2. CB-NAAT use amongst active providers in Ahmedabad and Delhi in 2019. To be classified as occasionally using CB-NAAT, we assumed that providers use CB-NAAT for at least 1% of notifications and less than 100% of notifications. Data provided by CHAI.

TB treatment

JEET offers two different adherence services for TB treatment via treatment coordinators: government supplied fixed-dose combinations (FDCs) and adherence support (table 3). Government supplied FDCs are supplied for free to the patient by the government, whereas patients on adherence support receive regular text messages and calls to ensure treatment adherence. Field officers visit active providers regularly to ensure they are continuing to recommend government supplied FDCs and adherence support.

	City	
	Ahmedabad	*Delhi*
Total no. of active providers	762	894
No. of active providers that offer adherence support	712	835
Percent of patients that agree to adherence support	92%	92%
No. of active providers that agree to government supplied FDCs	529	242
Percent of active providers that consistently use FDCs, unweighted	45%	10%
Percent of active providers that consistently use FDCs, weighted	39%	7%

Table 3. Number of active providers that offer government supplied FDCs and adherence support in Ahmedabad and Delhi in 2019. Data provided by CHAI.

We considered 4 categories of active providers in the model: active providers that use government supplied FDCs and offer adherence support, those that use government supplied FDCs but do not offer adherence support, those that offer adherence support but do not use government supplied FDCs, and those that offer neither service. We drew from data provided by CHAI to inform treatment completion rates associated with each of these categories, active providers offering both services having the highest rate of treatment completion and those offering neither having the lowest rate

For peer review only - http://bmjopen.bmj.com/site/about/guidelines.xhtml

(table S4). Finally, we assumed that inactive and unengaged providers do not offer adherence support and thus patients seeking treatment from these providers have the lowest treatment completion rates.

All patients diagnosed with DR-TB by a private provider are transferred to the public sector and initiated onto the second line treatment.

Interventions

We focused on the impact of the following four interventions:

- *Activate providers:* increasing the number of active providers amongst those already engaged but not notifying TB
- *Promote CB-NAAT (occasional users)* – increasing CB-NAAT use amongst occasional CB-NAAT users
- *Promote CB-NAAT (non-users)* – encouraging active providers that never use CB-NAAT into occasionally using CB-NAAT,
- *Promote adherence mechanisms* - increasing the use of government supplied FDCs amongst active providers.

We did not examine the impact of increasing adherence support, as current uptake is already high (above 90%) in Ahmedabad and Delhi.

We modelled a 'theoretical maximum coverage' scenario where all providers are amenable to intervention. In practice, however, some providers may remain uninterested in taking up the services offered despite sustained engagement efforts by field officers. To reflect these limits, we also modelled a 'feasible maximum' scenario where some proportion of providers remain unresponsive to the interventions listed above. Informed by expert consultation with CHAI, we assumed that: all inactive providers and occasional CB-NAAT users are interested in notifying and increasing their CB-NAAT use, whereas 40% of non-CB-NAAT users are uninterested in using CB-NAAT, and 35% of non-government supplied FDC users are uninterested in using government supplied FDCs (see fig S3 for more detail).

For both cities, we examined the epidemiological impact of increasing the four activities to their feasible and theoretical maxima, compared to an indefinite continuation of current levels of routine (publicly offered) TB and PPSA services. We modelled the scale-up of existing PPSA activities from 2017 to current levels in 2020. We assumed the interventions are scaled up linearly between 2020 and 2021, and simulated their impact until 2035.

Economic evaluation

We took the programmatic perspective, i.e., the costs that the national TB programme would have to pay. To examine which PPSA service should be prioritised in the future, we calculated the cost of

For peer review only - http://bmjopen.bmj.com/site/about/guidelines.xhtml

expanding the different services to their feasible and theoretical maximum using operational cost data from 2019 for Ahmedabad and Delhi. Table S12 summarises the total cost in 2019 for the different activities involved in the service provisions offered by JEET. These activities include the salaries of the field officer, treatment coordinators, hub agents, and SCT agents; the cost of continuing medical education (CMEs; seminars organised for providers to increase engagement); the cost of promotional materials which includes the cost of designing, printing and dissemination; the cost of transporting sputum samples to the laboratories, (including the cost of procuring falcon tubes, carrier boxes, masks and hand sanitizer); the cost of CB-NAAT tests, including test cartridges and manpower; and the cost of government supplied FDCs. We ignored sunk costs (office expenditure and state programme management costs) that have already been incurred in Ahmedabad and Delhi, and thus would not influence decisions for future interventions.

We categorised each cost item listed into table S12 into six different categories: provider activation, provider retention, diagnostic engagement, diagnostic logistics, treatment engagement and treatment logistics (described in further detail in table S13). Table S14 lists assumptions following expert consultation with CHAI, for the level of effort needed to engage providers and achieve increased uptake (subject to uncertainty analysis). If a cost was associated with more than one category, the cost was subdivided across the relevant categories based on estimations from CHAI on the percentage of time spent on each category (table S15). Next, we calculated the per-unit cost of each cost item (table S16-table S17). For example, to calculate the cost of field officers to retain one provider per year, we multiplied the total cost of field officers in 2019, by the percentage of time spent by field officers on provider retention in 2019, divided by the number of active providers in 2019.

In practice, increasing the coverage of intervention activities is achieved through field officer visits, to engage directly with the providers. Finally, we also considered costs of routine TB services provided by the public sector, including the cost of diagnosis and the cost of treating DS- and DR-TB (table S5).

We calculated the incremental cost-effectiveness ratio (ICER) by dividing the incremental cost of increasing the coverage of PPSA services by the number of TB cases averted between 2020 and 2035, relative to a baseline of indefinite continuation of current levels of routine TB and JEET PPSA services.

Sensitivity analyses

We conducted various sensitivity analyses to explore the impact of certain model assumptions on model results. Firstly, we conducted multivariate sensitivity analysis using partial rank correlation coefficient to assess which model parameters are most influential for model outputs.

We also conducted sensitivity analyses on provider behaviour. In the main analysis, we accounted for the fact that active providers may revert to an inactive state, and we assumed that the behaviour of newly active providers is the same as pre-existing active behaviours. First, we considered an

For peer review only - http://bmjopen.bmj.com/site/about/guidelines.xhtml

optimistic PPSA scale-up baseline that assumes active providers remain active once activated. Second, we assumed that newly active providers have 25% and 50% less uptake than pre-existing active providers (i.e., using Ahmedabad as an example, 9% of pre-existing active providers use CB-NAAT 100% of the time; we modelled scenarios where this proportion is 4.5% and 2.3%). We did not differentiate extrapulmonary and pulmonary TB in the model; however, CB-NAAT is unable to diagnose TB amongst patients with exclusively extrapulmonary disease. Thus, high levels of extrapulmonary TB can reduce the incremental benefit of CB-NAAT testing. In Ahmedabad and Delhi, it is estimated that 32% and 38% of patients diagnosed by JEET PPSA services between 2019 and 2022 had extrapulmonary TB, respectively. To account for this in a simple way, we reduced the proportion of TB patients presenting to care that are correctly diagnosed by CB-NAAT by 50%. For the purpose of sensitivity analysis, this reflects an extreme scenario where 50% of patients that present to care have extrapulmonary TB.

Finally, we examined the impact of varying maintenance costs by +/- 20% on the ICER of the different interventions. We defined maintenance costs as costs that are associated with maintaining good engagement and uptake amongst active providers (i.e., maintaining notifications, FDC use and CB-NAAT use).

Patient and public involvement

Patients or the public were not involved in the design, or conduct, or reporting, or dissemination plans of our research.

Results

Figs S4-S5 show results of model calibration, displaying the model fit against each of the calibration targets listed in table 1.

Figs 2 shows model projections for the impact on TB incidence between 2017 and 2035 in Ahmedabad and Delhi across varying levels of PPSA coverage, with cumulative impact summarised in table 4. Relative to a comparator where current TB services are maintained, increasing the four PPSA activities simultaneously to their feasible maximum coverage would avert 5.1% (95% CrI 4.4-6.2%) of cumulative TB cases between 2020 and 2035, whereas increasing them to their theoretical maximum would avert 7.4% (95% CrI 6.2-9.2%) of cumulative TB cases between 2020 and 2035 in Ahmedabad. In Delhi, 5.4% (95% CrI 4.4-6.9%) and 6.7% (95% CrI 5.3-8.9%) of cumulative TB cases would be averted under feasible and theoretical maximum coverages, respectively. There would be a larger impact on DR-TB cases and deaths averted as three of the four PPSA activities involved increasing the use of CB-NAAT, thus increasing the early detection of rifampicin resistance. For example, assuming PPSA activities are increased to their theoretical maximum coverage 14% (95% CrI 11-16%) and 35% (95% CrI 30-39%) of cumulative DR-TB cases and deaths would be averted between 2020 and 2035, respectively. This impact increases to 18% (95% CrI 15-22%) and 41% (95%

For peer review only

CrI 35-47%) DR-TB cases and deaths averted in Delhi, respectively, due to the lower current levels of CB-NAAT uptake in Delhi compared to Ahmedabad.

Percent reduction in cumulative TB burden, 2020-2035	**Ahmedabad**	**Delhi**
**Feasible maximum**		
TB incidence	5.1% (4.4-6.2)	5.4% (4.4-6.9)
TB deaths	4.1% (2.8-5.8)	6.4% (4.9-8.2)
DR-TB incidence	8.0% (6.2-9.7)	14% (11-17)
DR-TB deaths averted	20% (18-24)	32% (27-37)
**Theoretical maximum**		
TB incidence	7.4% (6.2-9.2)	6.7% (5.3-8.9)
TB deaths	7.9% (5.7-11)	8.7% (6.8-11.3)
DR-TB incidence	14% (11-16)	18% (15-22)
DR-TB deaths averted	35% (30-39)	41% (35-47)

Table 4. Projected epidemiological impact. Impact is measured as the cumulative impact on TB cases and deaths in Ahmedabad and Delhi between 2020 and 2035 relative to a baseline of current PPSA scale up.

In Ahmedabad, the interventions with the lowest ICERS were provider activation and promoting FDC use, costing an estimated $18,000 (12,000-29,000) and $36,000 (26,000-56,000) per TB case averted between 2020 and 2035, assuming the interventions were scaled up to their feasible maximum. Despite their low ICERs, these interventions were also the least epidemiologically impactful (table S18; fig 3). The interventions with the highest ICERS were those promoting CB-NAAT, costing $120,000 (96,000-180,000) and $46,000 (37,000-68,000) per TB case averted depending on whether CB-NAAT use was promoted amongst occasional users and non-users, respectively. For Delhi, promoting FDC use also had the lowest ICER costing an estimated $170,000 (110,000-310,000) per TB case averted between 2020 and 2035 (table S19, fig 3). However, promoting the use of CB-NAAT amongst non-users and provider activation had similar ICERs at $210,000 (150,000-320,000) and $280,000 (200,000-400,000) per TB case averted, respectively. Similar to Ahmedabad, promoting the use of CB-NAAT amongst occasional users had the highest ICER at $780,000 (500,000-900,000) per TB case averted. Fig 3 summarises the order in which the different interventions should be prioritised in Ahmedabad and Delhi, and table S20-table S21 provide a breakdown of the total costs.

Figures S6 – S8 show results of sensitivity analyses. Alternate assumptions for the proportion of active providers that remain consistently active and the proportion of TB patients with exclusively extrapulmonary TB reduced the epidemiological impact of increasing PPSA service provisions (fig S6) The qualitative findings shown in fig 3 were unchanged by alternative assumptions on the uptake of CB-NAAT and treatment adherence amongst newly recruited providers (table S18). However, assumptions relating to maintenance costs (the cost associated with maintaining good behaviour

For peer review only - http://bmjopen.bmj.com/site/about/guidelines.xhtml

amongst active providers) showed some influence on the prioritisation of interventions. For Ahmedabad, decreasing maintenance cost by 20% resulted in the promotion of government supplied FDCs being the priority intervention (table S22). On the other hand, increasing the proportion of active providers remained the individual intervention with the lowest ICER if maintenance cost increased by 20%. For Delhi, increasing the proportion of FDC users remained the intervention with the lowest ICER regardless of a 20% increase or decrease in maintenance cost (table S23).

Finally, we conducted multivariate sensitivity analysis to identify the parameters that are most influential for model outputs. Model projections for TB cases averted in Ahmedabad were influenced most strongly by the following three parameters: the rate of relapse following self-cure, the rate of stabilisation of the risk of relapse following treatment, and the rate of repeat care-seeking (fig S7). For Delhi, the three most influential parameters were also the rate of relapse following self-cure and the rate of stabilisation of the risk of relapse following treatment, as well as the rate of progression to active disease from latency (fig S8).

Discussion

Our analysis focuses on settings that have already seen an introduction of JEET services and addresses how these services should be expanded in future, to achieve reductions in TB burden in the most cost-effective way. Focusing on Ahmedabad and Delhi, two cities with different coverage levels of PPSA services, our analysis suggests that JEET operations should prioritise working with already-engaged providers to encourage them to participate more actively in Ahmedabad and increasing the use of government supplied FDCs amongst already-active providers in Delhi. Intervention priorities may vary in different settings across India, depending on local conditions, and the existing degree of uptake of PPSA services. For example, a key reason for the importance of increasing the use of FDCs in Delhi is the relatively low uptake of FDCs there so far. Overall, this work illustrates how programmatic data could be analysed using transmission modelling, to identify priorities for any given setting.

Previous modelling work estimated the potential epidemiological impact of scaling up PPSA operations in Mumbai and Patna, the cities where the PPSA pilots were first implemented (10). Subsequent analysis used a relatively simple costing model to illustrate how PPSA scaleup would be cost-effective in both settings (7). These findings echoed earlier analysis that similarly showed how public-private mix in India would be cost-effective (32). However, all these analyses were performed prospectively, and thus we had to make assumptions about the effectiveness of different interventions, in cities where PPSA operations did not already exist at scale. Now that JEET has been operating at scale in India, there is detailed programmatic data available for the costs, effectiveness, and real-world coverage of different interventions: our analysis thus adds to previous work by

For peer review only

analysing this data in a systematic way, to identify operational priorities for future expansion of PPSA activities, in given cities.

Overall, the interventions with the highest ICERs are those that involve CB-NAAT. Although these interventions are epidemiologically impactful, they are costly, due to the high cost of an CB-NAAT test; the cost of testing symptomatic non-TB infected care-seekers; and the cost of engaging with the active providers to increase their use of CB-NAAT. Reducing the effort needed to convince private providers to increase CB-NAAT use will be key in decreasing the overall cost. A recent study found that larger providers may be more willing to change their behaviour and adopt the use of CB-NAAT compared to smaller providers; thus, targeting these larger providers may provide a more cost-efficient solution and help bring down the cost of increasing the use of CB-NAAT amongst private providers (8). On the other hand, promoting uptake of FDCs – although having modest impact – also consistently showed the lowest estimated ICERs, because of its low cost.

The epidemiological impact is likely to be reduced if there is a high rate of attrition from an active to an inactive status amongst private providers. There are several reasons behind this attrition: first, some providers may not have a sufficient load of TB patients every month and thus may notify infrequently depending on when they see a new TB patient; second, some providers, especially those handling a smaller volume of TB patients, are seen less regularly by the field team and are therefore less likely to notify; third, some providers remain dissatisfied with the services offered by JEET, and therefore intentionally stop notifying patients; fourth, providers may be wary of the scrutiny they receive once they become active and involved with the programme. Understanding the reasons behind this behaviour will be important to ensure engagement efforts do not go to waste.

Limitations of this work can be divided into two categories: transmission model limitations and economic limitations. Owing to a lack of city-specific data, we drew from prevalence and infection surveys from Chennai, another large Indian city. This analysis focusses on urban settings and does not address rural settings, that may have different care-seeking pathways, different rates of TB infection and different provider behaviour (3,19). While JEET activities have so far focused on urban India, addressing needs for private sector engagement in rural settings is an important area for future work. Another area for future work will be to extend this analysis to other cities where PPSA activities have been ongoing. With over 480 such cities to draw from, any future analysis could prioritise cities that capture the geographical and cultural variations across the country. On the cost side, we had to make certain assumptions to fill data gaps. For example, there is no systematic data for the number of visits needed to change the behaviour of a provider; this parameter was estimated by the CHAI field team as a form of expert opinion. Another assumption we made is that the field team visits providers resistant to engagement efforts the same amount of time as non-resistant providers; in reality, after a couple of visits, field officers know which providers are resistant, and thus, may choose instead to focus their efforts on non-resistant providers. Similarly, we do not consider the fact that once a provider has been visited by field staff a certain number of times, any subsequent visits are likely to be ineffective; for example, data from the field team

For peer review only - http://bmjopen.bmj.com/site/about/guidelines.xhtml

suggests that the increase in a provider's uptake of CB-NAAT becomes negligible after approximately the 25th visit by a field officer. Finally, while our work concentrates on PPSA activities, there are other important interventions not incorporated in the analysis, for example Nikshay Poshan Yojana (ref), an initiative to provide TB patients with financial assistance towards nutritional support. While such measures are likely to have had important benefits to TB patients, they are equally available to those reported from both public and private sectors: thus, they may not substantially change our model projections for future PPSA priorities. Nonetheless, another important area for future work is to incorporate data for these and other initiatives, to achieve more refined estimates for the potential impact of future PPSA activities.

In summary, as India accelerates efforts for drastically reducing TB burden, engaging effectively with the private sector will play a critical role. Modelling analysis can play a useful role in supporting these efforts, not only in informing strategic planning, but also in addressing operational priorities in different settings. Identifying the most cost-efficient ways of achieving PPSA impact will be critical in benefiting TB patients, in the future of India's TB response.

Contributorship statement

NA conceptualized and supervised the study. All authors contributed to the methodology. SR conducted the formal analysis. NA and SR wrote the original draft. AP, RS and AS contributed to data curation and reviewed the draft.

Competing interests

None declared.

Funding

SR was supported by the Wellcome Trust, grant number 203839/A/16/Z.

Data sharing statement

All data relevant to the study are included in the article or uploaded as supplementary information.

Ethics approval

This study does not involve human participants or animal subjects.

For peer review only

References

1. Pai M, Behr MA, Dowdy D, Dheda K, Divangahi M, Boehme CC, et al. Tuberculosis. *Nature Reviews. Disease Primers*. 2016;2: 16076. https://doi.org/10.1038/nrdp.2016.76.

2. World Health Organisation. *Global tuberculosis report 2022*. 2022 [Accessed 11th May 2023]. https://www.who.int/publications-detail-redirect/9789240061729 [Accessed 11th May 2023].

3. Satyanarayana S, Nair SA, Chadha SS, Shivashankar R, Sharma G, Yadav S, et al. From Where Are Tuberculosis Patients Accessing Treatment in India? Results from a Cross-Sectional Community Based Survey of 30 Districts. *PLOS ONE*. 2011;6(9): e24160. https://doi.org/10.1371/journal.pone.0024160.

4. Sreeramareddy CT, Qin ZZ, Satyanarayana S, Subbaraman R, Pai M. Delays in diagnosis and treatment of pulmonary tuberculosis in India: a systematic review. *The International Journal of Tuberculosis and Lung Disease: The Official Journal of the International Union Against Tuberculosis and Lung Disease*. 2014;18(3): 255–266. https://doi.org/10.5588/ijtld.13.0585.

5. Arinaminpathy N, Batra D, Khaparde S, Vualnam T, Maheshwari N, Sharma L, et al. The number of privately treated tuberculosis cases in India: an estimation from drug sales data. *The Lancet Infectious Diseases*. 2016;16(11): 1255–1260. https://doi.org/10.1016/S1473-3099(16)30259-6.

6. Kundu D, Chopra K, Khanna A, Babbar N, Padmini TJ. Accelerating TB notification from the private health sector in Delhi, India. *The Indian Journal of Tuberculosis*. 2016;63(1): 8–12. https://doi.org/10.1016/j.ijtb.2016.02.002.

7. Deo S, Jindal P, Gupta D, Khaparde S, Rade K, Sachdeva KS, et al. What would it cost to scale-up private sector engagement efforts for tuberculosis care? Evidence from three pilot programs in India. *PLOS ONE*. 2019;14(6): e0214928.

8. Deo S, Jindal P, Papineni S. Integrating Xpert MTB/RIF for TB diagnosis in the private sector: evidence from large-scale pilots in Patna and Mumbai, India. *BMC Infectious Diseases*. 2021;21(123). https://doi.org/10.1186/s12879-021-05817-1.

9. Sachdeva KS, Kumar A, Dewan P, Kumar A, Satyanarayana S. New vision for Revised National Tuberculosis Control Programme (RNTCP): Universal access - 'reaching the un-reached'. *The Indian Journal of Medical Research*. 2012;135(5): 690–694.

10. Arinaminpathy N, Deo S, Singh S, Khaparde S, Rao R, Vadera B, et al. Modelling the Impact of Effective Private Provider Engagement on Tuberculosis Control in Urban India. *Scientific Reports*. 2019;9(3810).

11. Chadha S, Burugina Nagaraja S, Trivedi A, Satapathy S, N M D, Devi Sagili K. Mandatory TB notification in Mysore city, India: Have we heard the private practitioner's plea? *BMC Health Services Research*. 2017;17: 1. https://doi.org/10.1186/s12913-016-1943-z.

12. Central TB Division. *Guidance for TB notification in India*. 2012.

13. Joint Effort for Elimination of Tuberculosis. *Project JEET*. 2019. https://www.projectjeet.in

14. Joint Effort for Elimination of Tuberculosis. *2019 Annual Report*. 2020. https://www.projectjeet.in/wp-content/uploads/2020/10/JEET-Annual-Report-2019_Low-Res.pdf

For peer review only - http://bmjopen.bmj.com/site/about/guidelines.xhtml

15. Central TB Division. *Leading the way. India TB Report 2023.* 2023.

16. Joint Effort for Elimination of Tuberculosis. *2018 Annual Report*. 2019. https://projectjeet.in/wp-content/uploads/2019/08/JEET-Annual-Report-2018_v4.0_10MAY19.pdf

17. World Health Organisation. *A brief history of tuberculosis control in India.* 2010. https://apps.who.int/iris/bitstream/handle/10665/44408/9789241500159_eng.pdf?sequence=1

18. Central TB Division, Directorate General of Health Services, Ministry of Health with Family Welfare. *National Strategic Plan for Tuberculosis Elimination 2017-2025*. 2017.

19. Subbaraman R, Nathavitharana RR, Satyanarayana S, Pai M, Thomas BE, Chadha VK, et al. The Tuberculosis Cascade of Care in India's Public Sector: A Systematic Review and Meta-analysis. *PLOS Medicine*. 2016;13(10): e1002149. https://doi.org/10.1371/journal.pmed.1002149.

20. Houben RMGJ, Lalli M, Kranzer K, Menzies NA, Schumacher SG, Dowdy DW. What if they don't have tuberculosis? The consequences and trade-offs involved in false-positive diagnoses of tuberculosis. *Clinical Infectious Diseases*. 2019;68(1): 150–156. https://doi.org/10.1093/cid/ciy544.

21. Sachdeva KS, Raizada N, Sreenivas A, Van't Hoog A, van den Hof S, Dewan PK, et al. Use of Xpert MTB/RIF in decentralized public health settings and its effect on pulmonary TB and DR-TB case findings in India. *PLOS ONE*. 2015;10(5): e0126065. https://doi.org/10.1371/journal.pone.0126065.

22. Gopi PG, Prasad VV, Vasantha M, Subramani R, Tholkappian AS, Sargunan D, et al. Annual risk of tuberculosis infection in Chennai city. *Indian Journal of Tuberculosis*. 2008;55(3): 157–161.

23. Dhanaraj B, Papanna MK, Adinarayanan S, Vedachalam C, Sundaram V, Shanmugam S, et al. Prevalence and risk factors for adult pulmonary tuberculosis in a metropolitan city of South India. *PloS One*. 2015;10(4): e0124260. https://doi.org/10.1371/journal.pone.0124260.

24. World Health Organisation. *Global Tuberculosis Report 2020*. 2020. https://www.who.int/publications/i/item/9789240013131

25. Haario H, Saksman E, Tamminen J. An adaptive metropolis algorithm. *Bernoulli*. 2001;7(2): 223–242.

26. Ku CC, MacPherson P, Khundi M, Soko RHN, Feasey HRA, Nliwasa M, et al. Durations of asymptomatic, symptomatic, and care-seeking phases of tuberculosis disease with a Bayesian analysis of prevalence survey and notification data. *BMC Medicine*. 2021;19(1): 298. https://doi.org/10.1186/s12916-021-02128-9.

27. Stallworthy G, Dias HM, Pai M. Quality of tuberculosis care in the private health sector. *Journal of Clinical Tuberculosis and Other Mycobacterial Diseases*. 2020;20: 100171. https://doi.org/10.1016/j.jctube.2020.100171.

28. Pai M, Daftary A, Satyanarayana S. TB control: challenges and opportunities for India. *Transactions of the Royal Society of Tropical Medicine and Hygiene*. 2016;110(3): 158–160.

For peer review only - http://bmjopen.bmj.com/site/about/guidelines.xhtml

29. Das J, Kwan A, Daniels B, Satyanarayana S, Subbaraman R, Bergkvist S, et al. Use of standardised patients to assess quality of tuberculosis care: a pilot, cross-sectional study. *Lancet Infectious Diseases*. 2015;15(11): 1305–1313. https://doi.org/10.1016/S1473-3099(15)00077-8.

30. Van't Hoog A, Langendam MW, Mitchell E, Cobelens FG, Sinclair D, Leeflang MMG, et al. *A sytematic review of the sensitivity and specificity of symptom- and chest-radiography screening for active pulmonary tuberculosis in HIV-negative persons and persons with unknown HIV status*. 2013. https://www.who.int/tb/Review2Accuracyofscreeningtests.pdf

31. Steingart KR, Horne DJ, Pai M, Boehme CC, Dendukuri N. Xpert MTB/RIF assay for pulmonary tuberculosis and rifampicin resistance in adults (Review). *Cochrane Database of Systematic Reviews*. 2014;(1).

32. Pantoja A, Lönnroth K, Lal SS, Chauhan LS, Uplekar M, Padma MR, et al. Economic evaluation of public-private mix for tuberculosis care and control, India. Part II. Cost and cost-effectiveness. *The International Journal of Tuberculosis and Lung Disease: The Official Journal of the International Union Against Tuberculosis and Lung Disease*. 2009;13(6): 705–712.

Figures

Figure 1. Schematic illustration of the model structure. Panel A illustrates the TB transmission model structure**.** Model compartments are as follows: uninfected with TB (U), latent infection (L), active TB disease (I), presented for care and awaiting diagnosis (Dx), on TB treatment (Tx), temporarily dropped out of the TB care cascade due to missed diagnosis or pre-treatment loss-to-follow-up (E), and recovered with a low risk of relapse ($R^{(L)}$) or with a high risk of relapse ($R^{(H)}$). Dx and Tx are stratified by healthcare sector: private (pr) and public (pu) sector. The private sector is further subdivided into several types of providers as described in the main text. Not shown for simplicity is the acquisition and transmission of DR-TB. See supplementary information for further technical details, including model equations and calibration. Panel B illustrates the non-infected TB-symptomatic model structure. Model compartments are as follows: uninfected with TB but TB symptomatic ($U^{(NTB)}$), presented for care and awaiting diagnosis in the public sector ($Dx^{(NTB\text{-}Pu)}$) or private sector ($Dx^{(NTB\text{-}Pr)}$), and TB treatment after a false-positive diagnosis in the public sector ($Tx^{(NTB\text{-}Pu)}$) or private sector ($Tx^{(NTB\text{-}Pr)}$). The private sector is further subdivided into several types of providers as described in the main text. In order to maintain a constant population size amongst the non-infected TB-symptomatic population, we assume that upon treatment completion, individuals return to the uninfected TB-symptomatic state. The size of this population was determined so that amongst all patients presenting for care, there is a 10% prevalence of TB, in either the public or private sector (19–21).

Figure 2. Model projections for the impact of increasing all PPSA service provisions to their feasible or theoretical maximum has on TB incidence in Ahmedabad and Delhi between 2020 and 2035. Panel A and B

For peer review only - http://bmjopen.bmj.com/site/about/guidelines.xhtml

show the projected trajectory of TB incidence in Ahmedabad and Delhi, respectively, assuming current PPSA scale up between 2017 and 2020, and its maintenance indefinitely (blue-dashed line), assuming that PPSA service provisions are increased to their feasible or theoretical maximum from 2020 to 2021 and maintained indefinitely (green and purple line, respectively). Shaded areas show 95% credible intervals.

Figure 3. Expansion pathway of PPSA service provisions in Ahmedabad (panel A) and Delhi (panel B). The first point on the graph represents the intervention that had the lowest ICER on its own when scaled up to its feasible maximum coverage; the second point represents the additional PPSA, in combination with the first point, had the lowest ICER; and so on, until all interventions are included. Shaded area shows the 95% credible interval. See tables S18-S19 for further details.

For peer review only

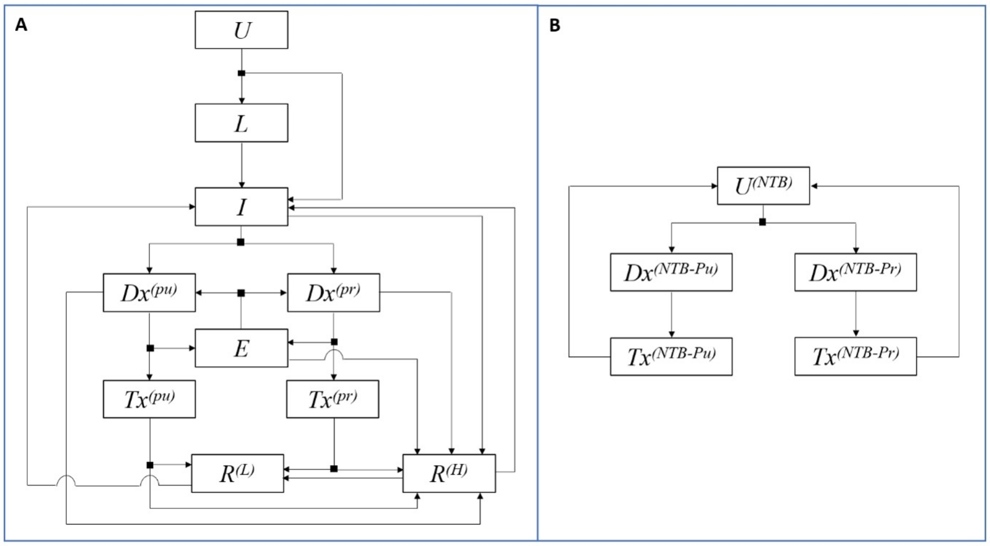

Figure 1. Schematic illustration of the model structure. Panel A illustrates the TB transmission model structure. Model compartments are as follows: uninfected with TB (U), latent infection (L), active TB disease (I), presented for care and awaiting diagnosis (Dx), on TB treatment (Tx), temporarily dropped out of the TB care cascade due to missed diagnosis or pre-treatment loss-to-follow-up (E), and recovered with a low risk of relapse (R(L)) or with a high risk of relapse (R(H)). Dx and Tx are stratified by healthcare sector: private (pr) and public (pu) sector. The private sector is further subdivided into several types of providers as described in the main text. Not shown for simplicity is the acquisition and transmission of DR-TB. See supplementary information for further technical details, including model equations and calibration. Panel B illustrates the non-infected TB-symptomatic model structure. Model compartments are as follows: uninfected with TB but TB symptomatic (U(NTB)), presented for care and awaiting diagnosis in the public sector (Dx(NTB-Pu)) or private sector (Dx(NTB-Pr)), and TB treatment after a false-positive diagnosis in the public sector (Tx(NTB-Pu)) or private sector (Tx(NTB-Pr)). The private sector is further subdivided into several types of providers as described in the main text. In order to maintain a constant population size amongst the non-infected TB-symptomatic population, we assume that upon treatment completion, individuals return to the uninfected TB-symptomatic state. The size of this population was determined so that amongst all patients presenting for care, there is a 10% prevalence of TB, in either the public or private sector (12–14).

159x90mm (220 x 220 DPI)

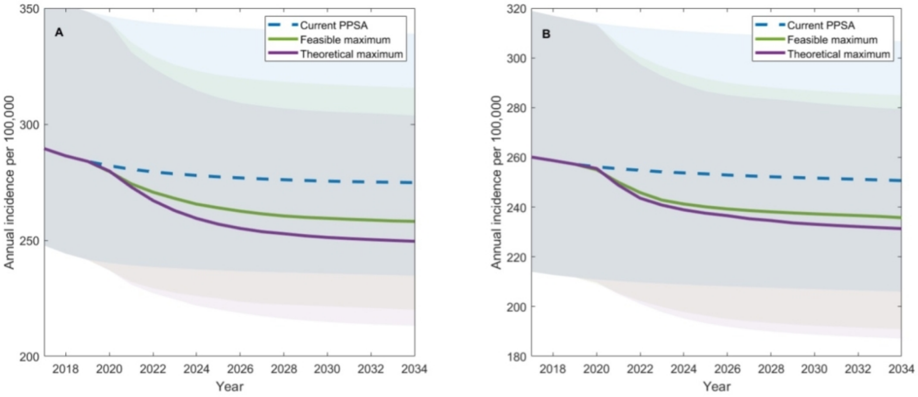

Figure 2. Model projections for the impact of increasing all PPSA service provisions to their feasible or theoretical maximum has on TB incidence in Ahmedabad and Delhi between 2020 and 2035. Panel A and B show the projected trajectory of TB incidence in Ahmedabad and Delhi, respectively, assuming current PPSA scale up between 2017 and 2020, and its maintenance indefinitely (blue-dashed line), assuming that PPSA service provisions are increased to their feasible or theoretical maximum from 2020 to 2021 and maintained indefinitely (green and purple line, respectively). Shaded areas show 95% credible intervals.

189x81mm (220 x 220 DPI)

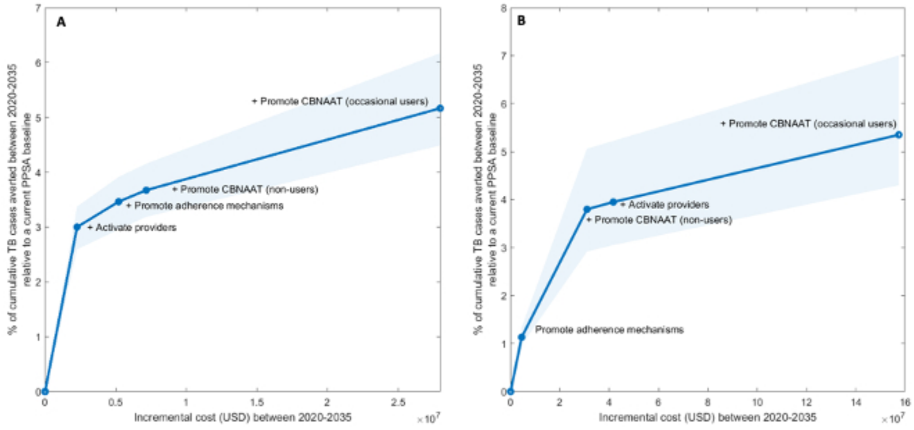

Caption : Figure 3. Expansion pathway of PPSA service provisions in Ahmedabad (panel A) and Delhi (panel B). The first point on the graph represents the intervention that had the lowest ICER on its own when scaled up to its feasible maximum coverage; the second point represents the additional PPSA, in combination with the first point, had the lowest ICER; and so on, until all interventions are included. Shaded area shows the 95% credible interval. See tables S18-19 for further details.

338x190mm (54 x 54 DPI)

Appendix 3

The value and cost efficiency of private sector engagement

1. Materials and methods

Below, we describe the model, including its structure, governing equations, and table of parameters. The model was implemented in MATLAB.

1.1. Model structure

We modelled TB transmission amongst adults (>15 years old) in a typical urban setting in India. We modelled the fragmented healthcare system, by differentiating between the public and private sector (1–7). We further divided the private sector by engagement status, CB-NAAT use and FDC use (see main text for more details on these categories). We calibrated the model to TB prevalence, annual risk of infection, and mortality (table 1). We also modelled the acquisition and transmission of DR-TB, but ignored HIV status as well as distinctions between pulmonary and extrapulmonary TB. The overall model structure is illustrated schematically in fig 1.

For a given set of model parameters, we initially simulated the model to equilibrium after introducing TB into a disease-free population. From 1997 to 2007 and its maintenance until the present day, we incorporated the scale-up of RNTCP (India's national TB programme) and its impact on the TB epidemic (8). From 1997, we also incorporated population growth. From 2017 to 2020, we simulated the scale-up of PPSA services to current 2019 levels. We then simulated the model forward from 2020 to 2035.

1.2. Governing equations for the mathematical transmission model

The equations correspond to the model described in fig 1. State variables (capital letters) are as listed in table S1, while model parameters (lower-case and Greek letters) are as listed in tables S2-S5.

For peer review only

Model stage	Description
U	Uninfected
L	Latent infection
I_q	Active TB
$Dx_q^{(pu)}$	Sought care, awaiting TB diagnosis, public sector
$Dx_q^{(pr)}$	Sought care, awaiting TB diagnosis, private sector. This is split further depending on engagement status and CB-NAAT use (table S6)
$Tx_q^{(FL:pu)}$	Undergoing first line TB treatment, public sector
$Tx_q^{(FL:pr)}$	Undergoing first line TB treatment, private sector. This is split further depending on FDC use and adherence support offered (table S6).
$Tx^{(SL)}$	Undergoing second line TB treatment.
E_q	Between seeking care, after misdiagnosis or loss-to-follow-up
$R^{(L)}$	Recovered after treatment completion, low risk of relapse
$R^{(H)}$	Recovered after self-cure or incomplete treatment, high risk of relapse
**Subscript**	**Description**
q	Drug resistance status (0 = DS-TB, 1 = DR-TB)

Table S1. Model stages and subscript description

Parameter	Symbol	Value		Source
**TB natural history**				
Mean rate of transmission per TB case	β	10.42 yr^{-1} [7.28-17.24]		Model estimate
Relative rate of transmission between DS- and DR-TB	d	0.61 yr^{-1} [0.49-0.79]		Model estimate
Rate of MDR acquisition	m_a	Public sector/active providers that offer either adherence support of government supplied FDCs	0.01 [0.008-0.012]	(9)
	m_b	All private providers that are unengaged, inactive, or active that do not offer government supplied FDCs or adherence support	0.05 [0.04-0.06]	
Proportion of TB infections undergoing rapid progression	θ	0.11 [0.09-0.15]		(10,11)
Per capita hazard rate of progression to active disease from latency	ρ	0.001 yr^{-1} [0.0003-0.0024]		(11,12)
Per-capita hazard rate of relapse	r_1	Following self-cure/treatment default	0.14 yr^{-1} [0.10-0.18]	(9,13)

	r_2	Following treatment cure, >2 years	0.002 yr^{-1} [0.001-0.003]	
Stabilisation of relapse risk following treatment	ς	0.5 yr^{-1} [0.4-0.6]		(13): most relapse occurs in the first two years after treatment
Per-capita hazard rate of spontaneous cure	φ	0.17 yr^{-1} [0.13-0.21]		(14)
Per-capita hazard rate of TB mortality	μ_1	0.18 [0.13-0.25]		Model estimate
Proportion reduction in susceptibility to reinfection due to previous infection	π	0.21 [0.15-0.25]		(15)
**Demographics**				
Per-capita hazard rate of background mortality	μ_2	0.015 yr^{-1}		World Bank estimates: corresponds to an average lifespan of 69 years
Per-capita rate of population growth	σ	0.02 [0.01-0.025]		(16)

Table S2. List of model parameters: TB natural history

Parameter	Symbol	Value		Source
**TB services: diagnosis**				
Proportion of TB cases seeking care in the public sector	η	0.50 [0.40-0.60]		(2,17–19)
Proportion of private providers that are engaged	e	Ahmedabad	0.50	Data provided by CHAI
		Delhi	0.73	
Heterogeneity index of the behaviour of engaged private providers	$\propto$	Ahmedabad	0.39 [0.32-0.46]	Data provided by CHAI
		Delhi	0.32 [0.25-0.41]	
Proportion of engaged private providers that are active	a	Ahmedabad	0.80	Data provided by CHAI
		Delhi	0.44	
Per-capita hazard rate of initial careseeking for TB symptoms	δ_1	1.38 yr^{-1} [0.88-2.38]		Model estimate
Per-capita hazard rate of repeat careseeking for TB symptoms (following missed diagnosis)	δ_2	12 yr^{-1} [6-26]		Assumption: corresponds to range of 2 weeks to 2 months

For peer review only

Proportion of TB cases diagnosed correctly, per careseeking attempt in routine public sector TB services	ε_{sn}	0.84 [0.80-0.85]		(20,21)
Specificity of diagnosis in routine public sector TB services	ε_{sp}	0.98 [0.97-0.99]		(20)
CB-NAAT sensitivity	x_{sn}	0.89 [0.85-0.92]		(22)
CB-NAAT specificity	x_{sp}	0.99 [0.98-1.00]		(22)
Proportion of active private providers that send samples for confirmatory CB-NAAT testing 100% of the time	p_{Xpert1}	Ahmedabad	0.09	Data provided by CHAI
		Delhi	0.04	
Proportion of active private providers that never send samples for confirmatory CB-NAAT testing	p_{Xpert0}	Ahmedabad	0.55	Data provided by CHAI
		Delhi	0.66	
Proportion of samples that are sent off for confirmatory CB-NAAT testing amongst active private providers that occasionally send samples off for confirmatory CB-NAAT testing	x_p	Ahmedabad	0.23	Data provided by CHAI
		Delhi	0.15	
Clinical diagnosis sensitivity (including chest X-ray)	c_{sn}	0.75 [0.65-0.80]		(21)
Clinical diagnosis specificity (including chest x-ray)	c_{sp}	0.85 [0.80-0.95]		(21)
Proportion of DR-TB cases receiving drug susceptibility testing at point of TB diagnosis	$\text{Þ}^{(pu)}$	Public sector	0.45 [0.40-0.50]	(23)
	$\text{Þ}^{(pr0)}$	Private sector (providers not using CB-NAAT)	0	
	$\text{Þ}^{(pr1)}$	Private sector (providers using CB-NAAT)	1.00	

Table S3. List of model parameters: TB diagnosis

For peer review only - http://bmjopen.bmj.com/site/about/guidelines.xhtml

Parameter	Symbol	Value		Source
**TB services: treatment**				
Proportion of diagnosed TB cases initiating TB treatment	ω	0.87 [0.85-0.90]		(20). Assumed that patients from active providers are referred to the public sector to initiate second line treatment.
Amongst DR-TB cases failing first-line treatment, the proportion successfully transferred to second-line treatment, public sector	p_{SL}	0.87 [0.85-0.92]		Assumption
TB treatment initiation delay	ϕ	52 yr^{-1}		Assumed: corresponds to a mean treatment delay of 1 week in routine TB care
Proportion of patients that agree to adherence support	p_{adh1}	0.92		Data provided by CHAI
Proportion of active providers that agree to adherence support	p_{adh2}	Ahmedabad	0.93	Data provided by CHAI
		Delhi	0.93	
Proportion of active providers that agree to government supplied FDCs	p_{fdc}	Ahmedabad	0.39	Data provided by CHAI
		Delhi	0.07	
Per-capita hazard rate of treatment completion	τ	First line	2 yr^{-1}	Corresponds to a duration of 6 months
	τ_{mdr}	Second line	0.5 yr^{-1}	Corresponds to a duration of 2 years
Proportion of TB patients that do not complete treatment	χ_{Pu}	First line, public sector	0.15 [0.13-0.17]	(23,24), data provided by CHAI
	χ_{Pr_Z}	First line, private sector: unengaged/inactive/active but no adherence support of government supplied FDCs offered	0.48 [0.45-0.55]	
	χ_{Pr_F}	First line, private sector: active (government	0.17 [0.16 -0.19]	

		supplied FDCs offered, no adherence support offered)		
	$\chi_{\mathrm{Pr\_A}}$	First line, private sector: active (no government supplied FDCs offered, adherence support offered)	0.21 [0.20-0.25]	
	$\chi_{\mathrm{Pr\_AF}}$	First line, private sector: active (government supplied FDCs and adherence support offered)	0.15 [0.13-0.17]	
	χ_{mdr}	Second line	0.50 [0.45-0.60]	

Table S4. List of model parameters: TB treatment

Parameter	Symbol	Value	Source
		**Costs**	
Cost per active TB disease diagnosis (passive case finding)	C_D	USD 18.30	(25)
Cost of first line treatment for active TB disease per person	C_T	USD 23.70 per treatment-month	(25)

Table S5. List of model parameters: TB costs

Label	Description
	**Private sector diagnosis classification**
pr1	Unengaged private providers
pr2	Engaged, inactive private providers
pr3	Engaged, active private providers that send samples for confirmatory CB-NAAT testing 100% of the time
pr4	Engaged, active private providers that send samples for confirmatory CB-NAAT testing occasionally, CB-NAAT
pr5	Engaged, active private providers that send samples for confirmatory CB-NAAT testing occasionally, no CB-NAAT
pr6	Engaged, active private providers that never send samples for confirmatory CB-NAAT testing
	**Private sector first-line treatment classification**
prZ1	Treatment offered by inactive and unengaged private providers
prAF	Engaged, active private providers that offer adherence support and government supplied FDCs
prA	Engaged, active private providers that only offer adherence support
prF	Engaged, active private providers that only offer government supplied FDCs
prZ2	Engaged, active private providers that offer neither adherence support or government supplied FDCs

Table S6. Private sector classification

Label	Classes
S_1	pu, prZ1, prZ2, prAF, prA, prF
S_2	pr1, pr2, pr5, pr6
S_3	pr3, pr4

Table S7. Notation

Uninfected stage

$$\frac{dU}{dt} = T - (\lambda_q + \mu_2)U \quad (1)$$

where term T represents births. To maintain a constant population for simplicity, we assumed that T is equivalent to the number of deaths occurring in the model.

Latent TB infection

$$\frac{dL_q}{dt} = (1-\theta)\lambda_q U + \pi\lambda_q(1-\theta) - (\rho + \mu_2 + \lambda_q)L_q \quad (2)$$

Active disease, pre-careseeking

$$\frac{dI_q}{dt} = \theta\lambda_q U + \pi\lambda_q\theta + \rho L_q + r_1 R^{(H)} + r_2 R^{(L)} - (\varphi + \delta_1 + \mu_1 + \mu_2)I_q \quad (3)$$

where the subscript q denotes drug resistance status:

$$q = \begin{cases} 0 & DS\ TB \\ 1 & DR\ TB \end{cases}$$

We assumed that, when presenting for care, a proportion η of patients visit the public sector, the remainder $(1-\eta)$ visiting the private sector. We divided the private sector further depending on whether private providers are engaged by JEET or not, and whether engaged providers are active or inactive, in notifying TB. Finally, active providers were categorised by their use of CB-NAAT (table S6-S7). The proportion of patients that visit each different type of provider is summarised in table S8.

For peer review only

Provider type	Notation	Formula
Public	$v^{(pu)}$	$v^{(pu)} = \eta$
Unengaged, private	$v^{(pr1)}$	$v^{(pr1)} = (1-\eta)(1-h^{\propto})^{*}$
Engaged, inactive, private	$v^{(pr2)}$	$v^{(pr2)} = (1-\eta)h^{\propto}(1-a)$
Engaged, active, private (always use CB-NAAT)	$v^{(pr3)}$	$v^{(pr3)} = (1-\eta)h^{\propto}ap_{CBNAAT1}$
Engaged, active, private (occasionally use CB-NAAT, patient receives an CB-NAAT test)	$v^{(pr4)}$	$v^{(pr4)} = (1-\eta)h^{\propto}a(1-p_{CBNAAT1}-p_{CBNAAT0})x_p$
Engaged, active, private (occasionally use CB-NAAT, patient does not receive an CB-NAAT test)	$v^{(pr5)}$	$v^{(pr5)} = (1-\eta)h^{\propto}a(1-p_{CBNAAT1}-p_{CBNAAT0})(1-x_p)$
Engaged, active, private (non CB-NAAT user)	$v^{(pr6)}$	$v^{(pr6)} = (1-\eta)h^{\propto}ap_{CBNAT0}$

Table S8. Proportion of patients that visit different providers. Private providers under the classifications pr1, pr2, pr5 and pr6 rely on clinical diagnosis, whereas private providers under the classification pr3 and pr4 employ CB-NAAT testing. *h is the proportion of private providers that have been engaged by the PPSA, and α is a 'heterogeneity index' governing the distribution of patient load across all providers (see fig S1).

The following equations describe the different pathways for patients that seek care with the different providers described above:

Seeking care, awaiting TB diagnosis

$$\frac{dDx_q^{(pu)}}{dt} = v^{(pu)}\delta_1 I_q + v^{(pu)}\delta_2 E_q$$

$$-\left[\varepsilon_1\omega\phi\left(1-s_q^{(pu)}\right) + \left(\varepsilon_1\omega\phi s_q^{(pu)}\right)_q + (1-\omega\varepsilon_1)\phi s_q^{(pu)} + \varphi + \mu_1 + \mu_2\right]Dx_q^{(pu)} \quad (4)$$

$$\frac{dDx_q^{(s)}}{dt} = v^{(s)}\delta_1 I_q + v^{(s)}\delta_2 E_q$$

$$-\left[\varepsilon_3\omega\phi(1-s_q^{(pr0)}) + (\varepsilon_3\omega\phi s_q^{(pr0)})_q + (1-\omega\varepsilon_3)\phi s_q^{(pr0)} + \varphi + \mu_1 + \mu_2\right]Dx_q^{(s)} \quad (5)$$

For peer review only

$$\frac{dDx_q^{(h)}}{dt} = v^{(h)}\delta_1 I_q + v^{(h)}\delta_2 E_q$$

$$-\left[\varepsilon_2\omega\phi(1 - s_q^{(pr1)}) + (\varepsilon_2\omega\phi s_q^{(pr1)})_q + (1 - \omega\varepsilon_2)\phi s_q^{(pr1)} + \varphi + \mu_1 + \mu_2\right] Dx_q^{(h)} \quad (6)$$

Where superscript (s) represents either pr1, pr2, pr5 or pr6 and superscript (h) represents either pr3 or pr4 as defined in table S8.

Patients that are misdiagnosed (as false-negative) enter the compartment E, to re-initiate careseeking after a delay:

Temporarily dropped out of careseeking

$$\frac{dE_q}{dt} = (1 - \omega\varepsilon_1)\phi Dx_q^{(pu)} + \sum_{i \in S_3} [(1 - \omega\varepsilon_2)\phi] Dx_q^{(i)} - (\varphi + \delta_2 + \mu_1 + \mu_2) E_q$$

when q=0 (7)

$$\frac{dE_q}{dt} = (1 - \omega\varepsilon_1)\phi s_{pu} Dx_q^{(pu)} + \sum_{i \in S_2} [(1 - \omega\varepsilon_3)\phi s_{pro}] Dx_q^{(i)} + \sum_{i \in S_3} [(1 - \omega\varepsilon_2)\phi s_{pr1}] Dx_q^{(i)}$$

$$+ \sum_{i \in S_1} \left[\tau(1 - p_{SL}) + \frac{\tau\chi_i}{1 - \chi_i}\right] Tx_q^{(i)} - (\varphi + \delta_2 + \mu_1 + \mu_2) E_q$$

when q=1 (8)

where the summand represents patients not being cured by first-line treatment, but not being transferred to second-line treatment. Subscript S_1, S_2, and S_3 are defined in table S7.

Treatment

We categorised active private providers by the treatment services offered: adherence support and government supplied FDCs (prAF); adherence support only (prA); government supplied FDCs only (prF); or neither (prZ2) (table S6). The proportion of patients at active providers that receive the different treatment services offered is defined in table S9.

For peer review only

Provider type	Notation	Formula
Adherence support and government supplied FDCs	$v^{(prAF)}$	$v^{(prAF)} = p_{adh1}p_{adh2}p_{fdc}$
Adherence support only	$v^{(prA)}$	$v^{(prA)} = p_{adh1}p_{adh2}(1 - p_{fdc})$
Government supplied FDCs only	$v^{(prF)}$	$v^{(prF)} = (1 - p_{adh1}p_{adh2})p_{fdc}$
Neither adherence support or government supplied FDCs	$v^{(prZ)}$	$v^{(prZ)} = (1 - p_{adh1}p_{adh2})(1 - p_{fdc})$

Table S9. Proportion of patients at active providers that receive treatment services.

Governing equations for the different Tx compartments are:

$$\frac{dTx_q^{(FL:y)}}{dt} = \varepsilon_i \phi \omega v^{(pr\_y)} Dx_q^{(y)} - (m_y + \tau + \frac{\tau \chi_y}{1-\chi_y} + \mu_2) Tx_q^{(FL:y)} \quad \text{when q = 0} \quad (9)$$

$$\frac{dTx_q^{(FL:y)}}{dt} = \varepsilon_i \phi \omega v^{(pr\_y)} (1 - s_y) Dx_q^{(y)} + m_y Tx_{q=0}^{(FL:y)} - (\tau p_{SL} + [\tau(1 - p_{SL}) + \frac{\tau \chi_y}{1-\chi_y}] + \mu_2) Tx_q^{(FL:y)} \quad \text{when q = 1} \quad (10)$$

$dTx_q^{(FL:y)}$ represents the different Tx compartments (pu, prZ1, prAF, prA, prF and prz2), defined in table S6. The parameter ε_i is the proportion of patients tested that are correctly diagnosed (in other words, the sensitivity of the test) and is dependent on where the patient is diagnosed: ε_1 when Dx^{pu}; ε_3 when Dx^{pr1}, Dx^{pr2}, Dx^{pr5}, Dx^{pr6}; and ε_2 when Dx^{pr3}, Dx^{pr4}. The parameter ϕ is the treatment initiation delay (assumed conservatively to be 1 week across all providers) and ω is the proportion of diagnosed TB cases initiating TB treatment (assumed to be 0.87 across all providers) (20). $v^{(pr\_y)}$, described above, applies only to TB patients that are diagnosed by active private providers. s_y is the proportion of MDR-TB cases receiving drug susceptibility testing at the point of TB diagnosis and is dependent on the amount of CB-NAAT testing. We assume the proportion is 0.45, 1.00 and 0 in the public sector ($s^{(pu)}$), respectively amongst private providers that always use CB-NAAT, and amongst private providers that do not use CB-NAAT, respectively (23). Thus, $1 - s_y$ represents TB patients with DR-TB that are not tested for drug resistance and are therefore mistakenly initiated onto first-line treatment. The parameter m_y is the rate of MDR acquisition while on first-line treatment and is assumed to be higher amongst all private providers (m_b) except active providers that offer government supplied FDCs and adherence support, than public sector providers (m_a) (9). The parameter τ is the per-capita rate of treatment completion for first line treatment and

For peer review only

its value corresponds to a duration of 6 months.

The parameter p_{SL} applies only to TB patients with DR-TB and is defined as the proportion of DR-TB cases that are successfully transferred to second-line treatment following failure of first-line treatment. The parameter χ_y is the proportion of TB cases that do not complete, or are non-adherent to, first line treatment; it is dependent on the quality of treatment services offered by providers and is informed by programmatic data (tables 2-5).

The following equation represents the number of DR-TB patients receiving second-line treatment:

$$\frac{dTx_q^{(SL)}}{dt} = \varepsilon_1 \omega \phi s_{pu} Dx_q^{(pu)} + \varepsilon_3 \omega \phi s_{pr0} \sum_{i \in S_2} Dx_q^{(i)} + \varepsilon_2 \omega \phi s_{pr1} \sum_{i \in S_3} Dx_q^{(i)}$$

$$+\tau p_{SL} \sum_{i \in S_1} Tx_q^{(i)} - \left(\tau_{mdr} + \frac{\tau_{mdr} \chi_{\text{mdr}}}{1 - \chi_{\text{mdr}}} + \mu_2\right) Tx_q^{(SL)} \qquad (11)$$

Where S_1, S_2 and S_3 are sets of indices defined in table S7.

TB patients that successfully complete TB treatment, recover with a low risk of relapse. Governing equations for those with low risk of relapse are:

Recovery, low risk of relapse

$$\frac{dR^{(L)}}{dt} = \tau \sum_{i \in S_1} Tx_q^{(i)} + \varsigma R^{(H)} - \left(r_2 + \mu_2 + \lambda_q\right) R^{(L)} \qquad (12)$$

Where S_1 is the set of indices defined in table S7.

On the other hand, those recovered from TB with a high risk of relapse (either from previous inadequate treatment or self-cure) have governing equations:

For peer review only

Recovery, high risk of relapse

$$\frac{dR^{(H)}}{dt} = \frac{\tau\chi_{pu}}{1-\chi_{pu}} Tx_q^{(FL:pu)} + \sum_{i\in S_1} (\frac{\tau\chi_{(i)}}{1-\chi_{(i)}}) Tx_q^{(i)} + \varphi \sum_{i\in[S_2,S_3]} [Dx_q^{(pu)} + Dx_q^{(i)} + E_q + I_q]$$

$$-(\varsigma + r_1 + \mu_2 + \lambda_q)R^{(H)} \quad (13)$$

Where S_1, S_2 and S_3 are sets of indices defined in table S7.

The force of infection can be written as:

$$\lambda_q = \frac{\beta\{\sum_{i\in[S_2,S_3]}[I_q + Dx_q^{pu} + Dx_q^{(i)} + E_q]\} + d_q\beta\{\sum_{i\in[S_2,S_3]}[I_q + Dx_q^{pu} + Dx_q^{(i)} + E_q]\}}{N} \quad (14)$$

Where S_2 and S_3 are sets of indices defined in table S7.

Non-TB symptomatic population

To count the number of false positive diagnoses that arise, we modelled a non-TB symptomatic population (fig 1). We defined non-TB symptomatics as patients who do not have TB, but who have symptoms that would make them eligible for testing for TB. We assumed this population to be independent of the TB population described above.

Non-TB symptomatics seeking care

$$\frac{dU^{(NTB)}}{dt} = y_i\phi\omega Dx^{(NTB)} + \tau Tx^{(NTB)} - \delta_1 v^{(x)} U^{(NTB)} \quad (15)$$

where $v^{(x)}$ is drawn from equations 4 to 10, depending on the type of provider a patient seeks care from, and where y_i represents either the specificity of TB diagnosis in the public sector (y_1), specificity of CB-NAAT (y_2) or the specificity of clinical diagnosis (y_3).

Awaiting diagnosis

$$\frac{dDx^{(NTB)}}{dt} = \delta_1 v^{(x)} U^{(NTB)} - \phi\omega Dx^{(NTB)} \quad (16)$$

For peer review only

Undergoing (unnecessary) first-line TB treatment

$$\frac{dTx^{(NTB)}}{dt} = (1 - y_i)\phi\omega Dx^{(NTB)} - \tau Tx^{(NTB)} \quad (17)$$

We assumed the total population size of the non-TB symptomatic population remains constant; we determined the size of this population so that for every true positive TB diagnosis, approximately nine uninfected individuals who present for diagnosis with symptoms of TB are also tested for TB (20,26–30).

1.3. Model calibration

We performed calibrations using Adaptive Bayesian MCMC (table 1 -5)(31), allowing propagation of uncertainty from model inputs to projections in a systematic way.

We define the posterior distribution as:

$$p(\theta|D) \propto L(D|\theta) \, . \, \pi(\theta) \quad (18)$$

where D is data; θ is a vector of model inputs subject to uncertainty; L is the likelihood of the data given θ, and π is the prior distribution. We assumed uniform distributions for the prior distributions. We defined the likelihood by fitting log-normal distributions to the calibration targets (see table 1).

Once the MCMC converged, we removed the burn-in period (10,000 iterations) and selected every 50th sample to reduce any autocorrelation. This provided 1200 samples from the posterior distribution.

For peer review only

2. Supporting figures

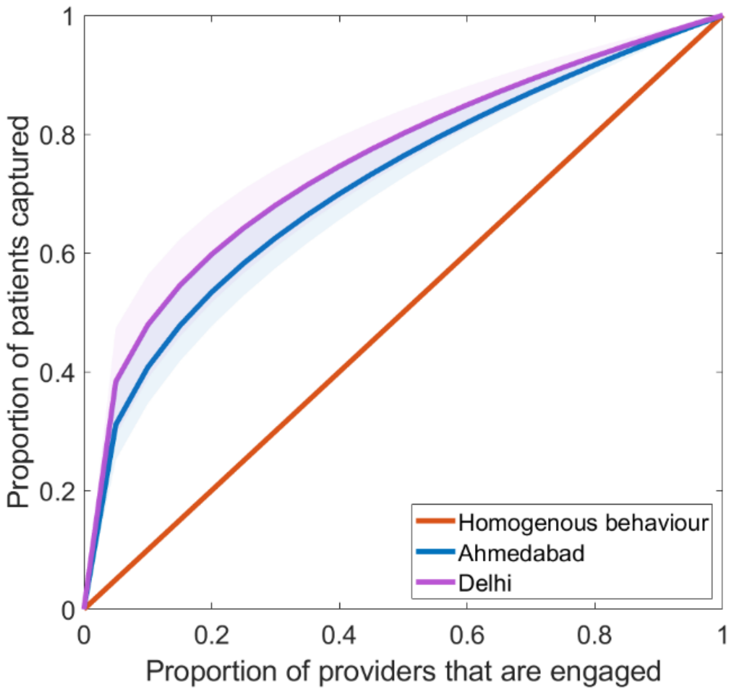

Figure S1. Heterogeneity in the patient share of private providers across Ahmedabad and Delhi. Homogenous patient share (red line) corresponds to a scenario where all private providers have the same numbers of patients. Under this assumption, increasing the proportion of engaged providers will linearly increase the proportion of patients benefiting from a PPSA. In reality, there are a few large private providers that manage most TB patients, and many smaller providers that manage few patients. The larger the area under the curve, the greater the heterogeneity. If the providers that capture the most patients are already engaged by JEET, increasing the proportion of engaged providers further will have little incremental benefit. Therefore, assuming homogenous patient share can overestimate the impact on TB epidemiology. As described in the main text, the 'heterogeneity index' controls the curvature of these plots. Table S11 shows the heterogeneity index calculated for each city.

review only

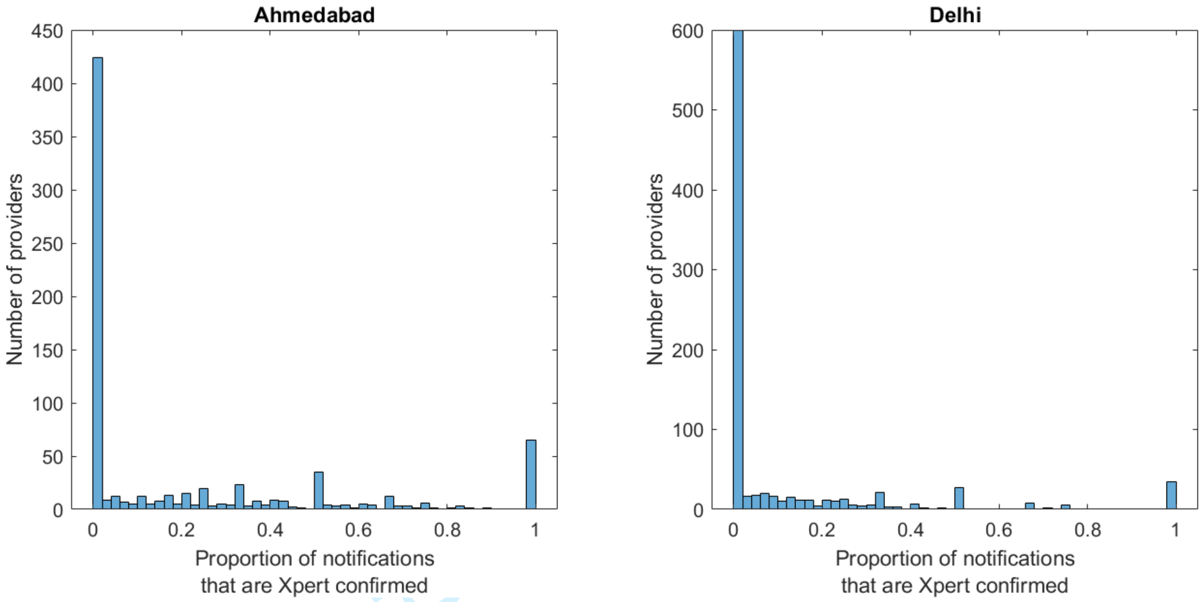

Figure S2. CB-NAAT use amongst active private providers in Ahmedabad and Delhi in 2019. From this data, we classified active providers into three categories: those that always use CB-NAAT, those that use CB-NAAT occasionally, and those that never use CB-NAAT. To be classified as occasionally using CB-NAAT, we assume that providers need to use CB-NAAT for at least 1% of notifications and less than 100% of notifications.

For peer review only

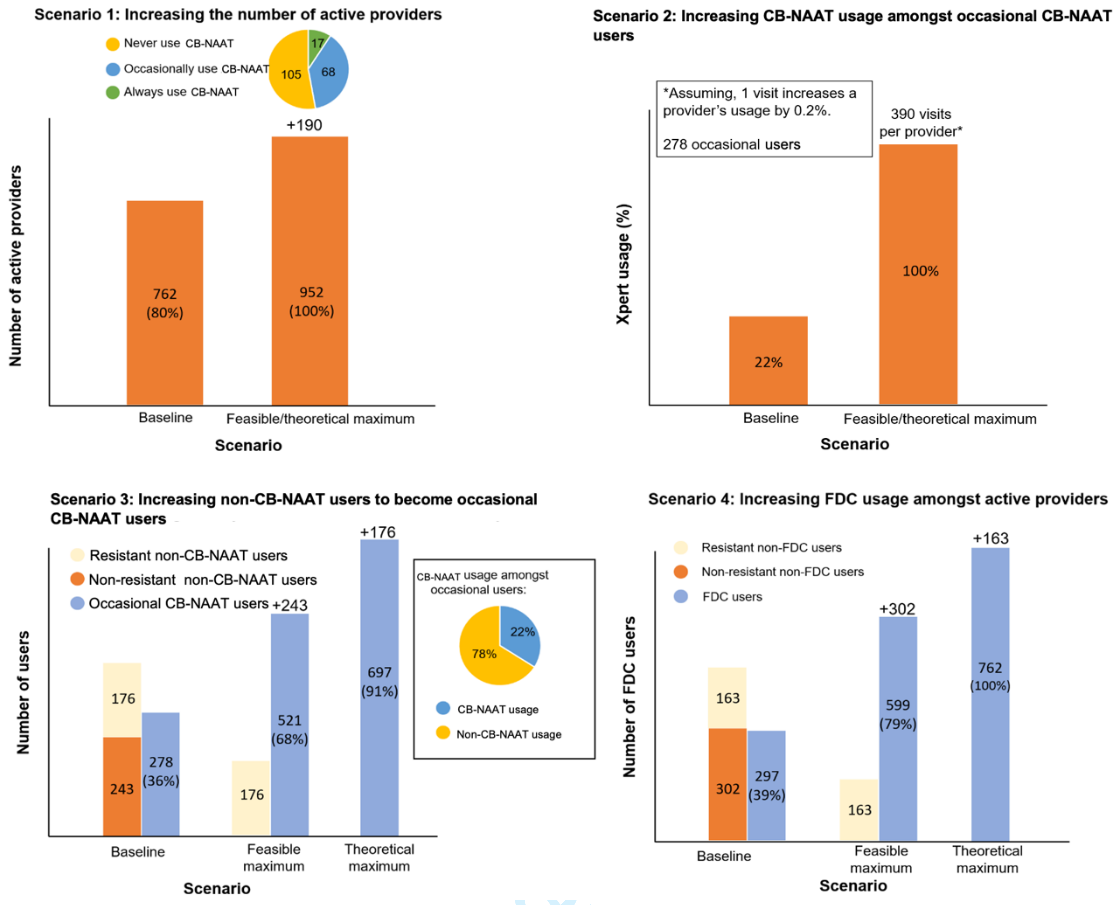

Figure S3. Feasible and theoretical maxima for each type of service provision, Ahmedabad. *Scenario 1:* We assume all inactive providers (e.g., 190) are converted into active providers. We assume no inactive providers are resistant, thus the feasible and theoretical maximum scenarios are the same. Newly active providers are assumed to have the same behaviour as pre-existing active providers; in the case of Ahmedabad, 9% use CB-NAAT 100% of the time, 36% use CB-NAAT occasionally (with an average CB-NAAT usage of 22%), 55% never use CB-NAAT, 98% offer adherence support and 39% consistently use governmental FDCs. For example, of the 190 newly active providers, 17 providers use CB-NAAT 100% of the time, 68 use CB-NAAT occasionally, and 105 never use CB-NAAT. *Scenario 2:* At baseline, occasional CB-NAAT users use CB-NAAT 22% of the time; this is increased to 100% under the feasible and theoretical maximum scenario. We assume no occasional users are resistant to increasing their CB-NAAT usage. It is estimated that one visit by a field officer increases a provider's usage of CB-NAAT by 0.2%. At baseline, Ahmedabad has 278 occasional CB-NAAT users. *Scenario 3:* At baseline, there are 419 non-CB-NAAT users and 278 occasional users. Of the 419 non-CB-NAAT users, it is estimated that 42% are resistant to using CB-NAAT; thus, only 243 non-CB-NAAT users can be converted to become an occasional user. At the theoretical maximum, the percentage of active providers that use CB-NAAT occasionally is 91%; the remaining 9% are providers that always use CB-NAAT. *Scenario 4:* At baseline, there are 465 non-FDC users and 297 consistent FDC users. Of the 465, 35% are resistant to FDCs.

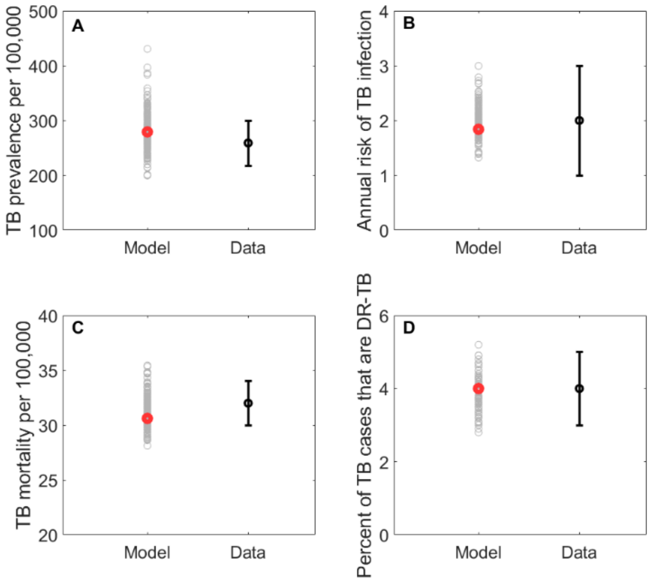

Figure S4. Model fits to data. Data points are described in table 1. Panel A – TB prevalence per 100,000 in a typical urban Indian setting; B – Annual risk of TB infection in a typical urban Indian setting; C – TB deaths per 100,000 in 2019, India; D – Percent of TB cases that are drug-resistant TB in 2019

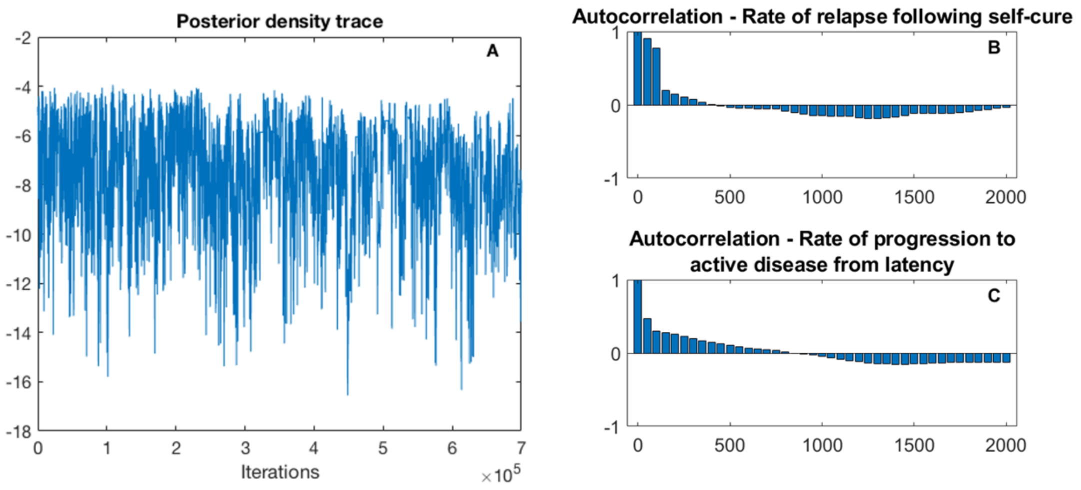

Figure S5. MCMC diagnostics. Panel A shows the posterior density trace, excluding the 'burn-in' period. Panel B and C show autocorrelation plots for two selected parameters (rate of relapse following self-cure and the rate of progression to active disease from latency).

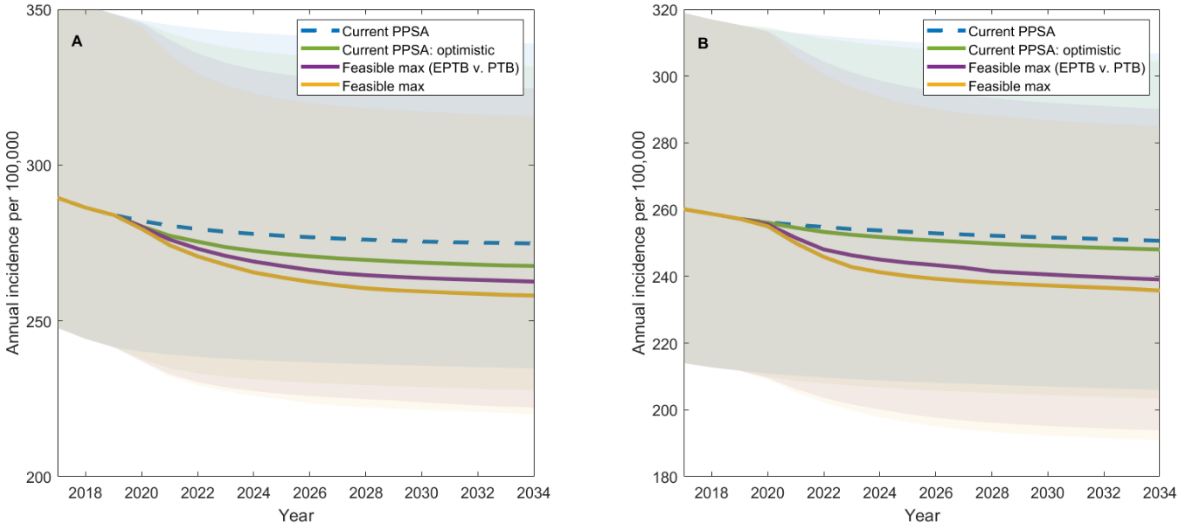

Figure S6. Sensitivity analysis on model projections for the impact of increasing all service provisions to their feasible maximum has on TB incidence in Ahmedabad and Delhi between 2020 and 2035. Panel A shows the projected trajectory of TB incidence for Ahmedabad and Panel B for Delhi, assuming current PPSA scale up between 2017 and 2020 (blue dashed line) and its maintenance indefinitely; assuming current PPSA scale up between 2017 and 2020 whilst optimistically assuming that active providers are continuously active (green line); service provisions are increased to their feasible maximum from 2020 to 2021 and maintained indefinitely, but assuming that 50% of TB cases that present to care are extra-pulmonary TB (purple line); that service provisions are increased to their feasible maximum from 2020 to 2021 and maintained indefinitely, as presented in the main text (yellow line).

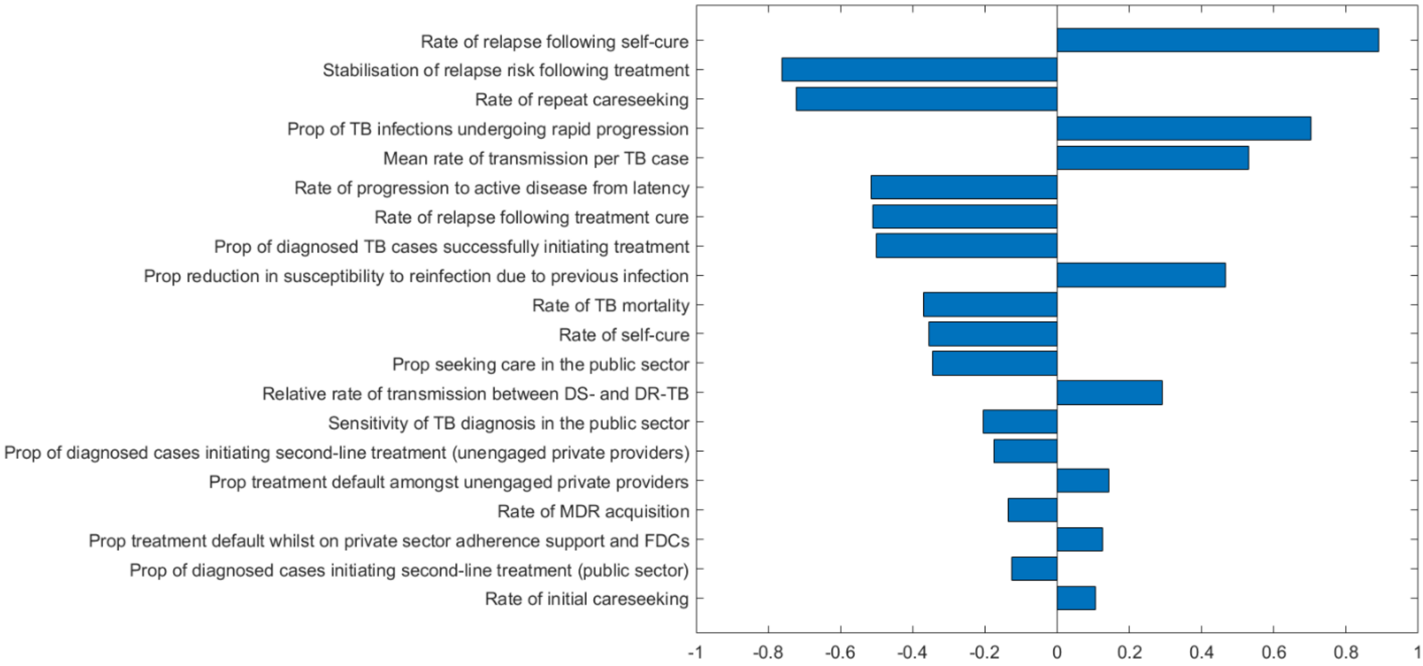

Figure S7. Multivariate sensitivity analysis of impact for Ahmedabad. We used partial rank correlation coefficient (PRCC) to examine which parameter listed in tables 2-5 the output cases averted is most sensitive towards. Larger bars represent more sensitive parameters. Shown are the 20 most influential model parameters, in decreasing order of sensitivity from top to bottom.

For peer review only

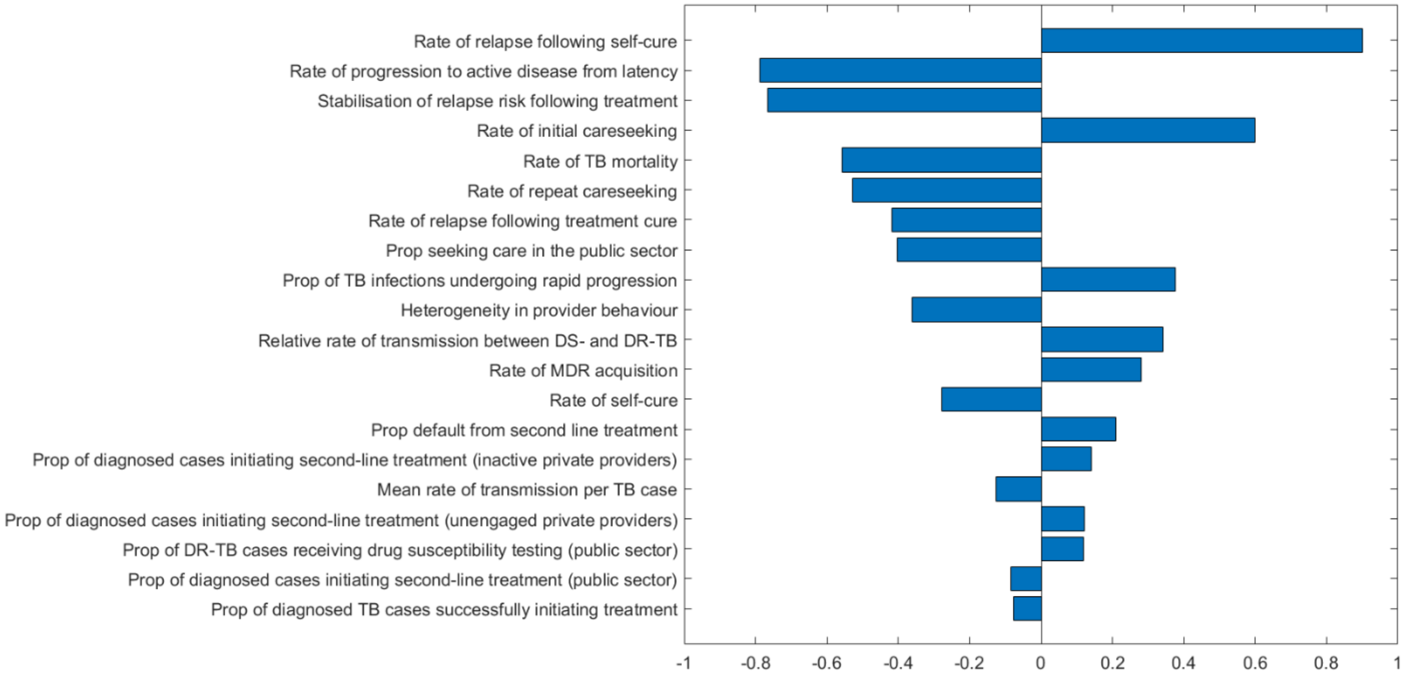

Figure S8. Multivariate sensitivity analysis of impact for Delhi. We used partial rank correlation coefficient (PRCC) to examine which parameter listed in tables 2-5 the output cases averted is most sensitive towards. Larger bars represent more sensitive parameters. Shown are the 20 most influential model parameters, in decreasing order of sensitivity from top to bottom.

For peer review only

3. Supporting tables

City	Number of identified private providers	Number of engaged private providers	Number of active private providers
Ahmedabad	1899	952	762
Delhi	2780	2016	894

Table S10. Number of identified, engaged, and active private providers in 2019 in Ahmedabad and Delhi. We assume that all private providers have been identified.

City	Heterogeneity index (α)
Ahmedabad	0.39 [0.32-0.46]
Delhi	0.32 [0.25-0.41]

Table S11. Heterogeneity index of private providers across Ahmedabad and Delhi. A heterogeneity index of 1 suggests that each provider sees the same number of patients (represented by the red line in fig S1). A smaller heterogeneity index indicates that a smaller number of providers are responsible for a larger number of patients. To calculate the heterogeneity, we divided the logarithm of the proportion of notifications by the logarithm of the proportion of providers responsible for those notifications.

Cost item	Total cost in 2019 (USD)	
	Ahmedabad	Delhi
Field officer salary	31,433	55,936
Hub agent salary	113,808	281,373
SCT agent salary	2055	5287
Treatment coordinator salary	8569	13,445
CMEs	460	6095
Promotional material	10	10
Sputum sample logistics	2020	5090
CB-NAAT tests	83,886	98,088
FDCs**	N/A	

Table S12. Total costs of PPSA activities in Ahmedabad and Delhi in 2019. We assume a conversion rate of USD 1 = 73 INR. *We assume a fix cost per FDC of 45 USD.

For peer review only

Category	Description
Provider activation	Costs associated with the engagement of inactive providers to encourage them to become active and start notifying their TB cases
Provider retention	Costs associated with the engagement of active providers to encourage them to remain active and keep notifying their TB cases
Diagnostic engagement	Costs associated with encouraging active providers to start using CB-NAAT, to increase their use of CB-NAAT and maintain their use of CB-NAAT
Diagnostic logistics	All other costs associated with CB-NAAT testing that are not to do with engagement. Includes cost of transporting sputum samples, cost of staff and cost of the CB-NAAT tests.
Treatment engagement	Costs associated with encouraging active providers to start using, increase or maintain their use of government supplied FDCs and adherence support.
Treatment logistics	Cost of government supplied FDCs and adherence support

Table S13. Costing categories. We categorise each cost item into one of the categories listed in this table to help identify the cost items that will increase as the coverage of the different interventions increase.

PPSA activity	Effort needed
Provider activation	5 (4-7) visits needed to onboard an inactive provider
Increasing CB-NAAT usage amongst occasional users	One visit increases a provider's CB-NAAT use by 0.2%. Assumed the same across all cities.
Converting non-CB-NAAT users to occasionally use CB-NAAT	7 (5-8) visits needed to convert a non-CB-NAAT user. Assumed the same across all cities
Converting non-FDC users to use FDC	6 (4-7) visits needed to convert a non-FDC user. Assumed the same across all cities.

Table S14. Engagement effort needed for each PPSA service provision in Ahmedabad and Delhi in 2019. As described in the text, values shown in these tables are essentially expert opinion, elicited through in-dept discussion with implementing teams from CHAI.

For peer review only

Ahmedabad						
**Cost item**	**Cost category**					
	Provider activation	Provider retention	Diagnostic engagement	Diagnostic logistics	Treatment engagement	Treatment logistics
Field officer	10%	42%	24%	-	24%	-
Hub agent	-	51%	-	24%	-	25%
SCT agent	-	-	-	100%	-	-
Treatment coordinator	-	-	-	-	-	100%
CMEs	-	40%	30%	-	30%	-
Promotional material	-	52%	29%	-	19%	-
Sputum sample logistics	-	-	-	100%	-	-
CB-NAAT	-	-	-	100%	-	-
FDCs	-	-	-	-	-	100%
**Delhi**						
**Cost item**	**Cost category**					
	Provider activation	Provider retention	Diagnostic engagement	Diagnostic logistics	Treatment engagement	Treatment logistics
Field officer	13%	28%	32%	-	27%	-
Hub agent	-	74%	-	18%	-	8%
SCT agent	-	-	-	100%	-	-
Treatment coordinator	-	-	-	-	-	100%
CMEs	-	40%	30%	-	30%	-
Promotional material	-	52%	29%	-	19%	-
Sputum sample logistics	-	-	-	100%	-	-
CB-NAAT	-	-	-	100%	-	-
FDCs	-	-	-	-	-	100%

Table S15. Costing split across PPSA activities for Ahmedabad and Delhi in 2019. The field officer split was determined by the percentage of calls that were made by a field officer for provider activation, provider retention, diagnostic engagement and treatment engagement; similarly, the hub agent split was determined by the proportion of time hub agents estimated they had spent on provider retention, diagnostic logistics and treatment logistics; the CMEs and promotional materials splits were determined by the amount of engagement that was aimed at provider retention, diagnostic engagement and treatment engagement, which was assumed constant across all cities. Each row adds up to 100%.

Cost item	Total cost	Category split	Unit description	No. of units	Cost per unit (USD)
Field officer	31,433	10%	No. of visits in 2019 made to onboard an inactive provider	1548	2.08
**PPSA activity: Provider retention**					
Field officer	31,433	42%	No. of active providers in 2019	762	17.77
Hub agent	113,808	51%	No. of active providers in 2019	762	78.12
CMEs	460	40%	No. of providers that attended CMEs in 2019	51	3.70
Promotional material	10	52%	No. of active providers in 2019	762	0.007
**PPSA activity: Diagnostic engagement**					
Field officer	31,433	24%	No. of visits in 2019 made to active providers to increase CB-NAAT usage	3572	2.17
			No. of active providers in 2019	762	10.15
CMEs	460	30%	No. of providers that attended CMEs in 2019	51	2.78
Promotional material	10	29%	No. of active providers in 2019	762	0.004
**PPSA activity: Diagnostic logistics**					
Hub agent	113,808	24%	No. of CB-NAAT tests performed in 2019	3615	7.75
SCT agent	2055	100%	No. of CB-NAAT tests performed in 2019	3615	0.58
Sputum sample logistics	2020	100%	No. of CB-NAAT tests performed in 2019	3615	0.57
CB-NAAT tests	83,886	100%	No. of CB-NAAT tests performed in 2019	3615	23.80
**PPSA activity: Treatment engagement**					
Field officer	31,433	24%	No. of visits in 2019 made to active providers to use FDCs	3619	2.14
			No. of active providers in 2019	762	10.15
CMEs	460	30%	No. of providers that attended CMEs in 2019	51	2.78
Promotional material	10	19%	No. of active providers in 2019	762	0.003
**PPSA activity: Treatment logistics**					
Treatment coordinator	8569	100%	No. of patient treatment months in 2019	57,868	0.15
Hub agent	113,808	25%	No. of FDC initiations in 2019	3445	8.47
FDCs	-	-	-	-	45

Table S16. Unit costs for Ahmedabad

Cost item	Total cost	Category split	Unit description	No. of units	Cost per unit (USD)
**PPSA activity: Provider activation**					
Field officer	55,936	13%	No. of visits in 2019 made to onboard an inactive provider	2984	2.50
**PPSA activity: Provider retention**					
Field officer	55,936	28%	No. of active providers in 2019	894	17.97
Hub agent	281,373	74%	No. of active providers in 2019	894	238.88
CMEs	6095	40%	No. of providers that attended CMEs in 2019	1469	1.70
Promotional material	10	52%	No. of active providers in 2019	894	0.006
**PPSA activity: Diagnostic engagement**					
Field officer	55,936	32%	No. of visits in 2019 made to active providers to increase CB-NAAT usage	7219	2.54
			No. of active providers in 2019	894	20.54
CMEs	6095	30%	No. of providers that attended CMEs in 2019	1469	1.28
Promotional material	10	29%	No. of active providers in 2019	894	0.003
**PPSA activity: Diagnostic logistics**					
Hub agent	281,373	18%	No. of CB-NAAT tests performed in 2019	4227	12.29
SCT agent	5287	100%	No. of CB-NAAT tests performed in 2019	4227	1.28
Sputum sample logistics	5090	100%	No. of CB-NAAT tests performed in 2019	4227	1.23
CB-NAAT tests	98,088	100%	No. of CB-NAAT tests performed in 2019	4227	23.80
**PPSA activity: Treatment engagement**					
Field officer	55,936	27%	No. of visits in 2019 made to active providers to use FDCs	5863	2.64
			No. of active providers in 2019	894	17.33
CMEs	6095	30%	No. of providers that attended CMEs in 2019	1469	1.28
Promotional material	10	19%	No. of active providers in 2019	894	0.002
**PPSA activity: Treatment logistics**					
Treatment coordinator	13,445	100%	No. of patient treatment months in 2019	108,915	0.13
Hub agent	281,373	8%	No. of FDC initiations in 2019	1480	13.65
FDCs	-	-	-	-	45

Table S17. Unit costs for Delhi.

Intervention	Incremental cost (2020-2035, USD)	Percentage of cumulative TB cases averted (2020-2035)	Cost-efficiency
**Individual interventions**			
Increasing the number of active providers (assuming newly active providers adopt average behaviour)	2,269,300 (1,477,000 – 3,723,000)	3.00% (2.59-3.37)	0.0032 (0.0019- 0.0050)
Increasing the number of active providers (assuming newly active providers' behaviour is 25% worse than average)	2,224,400 (1,435,200 – 3,672,300)	2.98% (2.58-3.35)	0.0032 (0.0019- 0.0051)
Increasing the number of active providers (assuming newly active providers' behaviour is 50% worse than average)	2,120,000 (1,339,000 – 3,555,300)	2.89% (2.48-3.24)	0.0033 (0.0019- 0.0053)
Increasing CB-NAAT use amongst occasional users	9,005,000 (6,801,000 – 13,190,000)	2.83% (2.42-3.33)	0.0008 (0.0006- 0.0010)
Increasing the number of occasional CB-NAAT users	2,211,700 (1,683,500 – 3,275,100)	2.32% (1.97-2.67)	0.0025 (0.0018- 0.0033)
Increasing the number of FDC users	1,787,700 (1,438,900 – 2,503,800)	2.53% (2.13-2.91)	0.0031 (0.0024- 0.0041)
**Combination of the two most cost-efficient interventions: Increasing the number of active providers* + …**			
+Increasing the number of occasional CB-NAAT users	5,020,300 (3,590,000 – 7,512,900)	3.21% (2.84-3.61)	0.0015 (0.0010- 0.0022)
+ Increasing CB-NAAT use amongst occasional users	13,427,000 (10,019,000 – 19,839,000)	3.81% (3.40-4.45)	0.0007 (0.0005- 0.0009)
+ Increasing the number of FDC users	5,217,100 (3,791,600 – 8,182,300)	3.46% (2.96-3.91)	0.0016 (0.0010- 0.0022)
**Combination of the three most cost-efficient interventions: Increasing the number of active providers* + the number of FDC-users + …**			
+Increasing the number of occasional CB-NAAT users	7,163,000 (5,671,000 – 10,478,000)	3.67% (3.18-4.16)	0.0012 (0.0008- 0.0017)
+ Increasing CB-NAAT use amongst occasional users	15,467,000 (11,921,000 – 22,411,000)	4.27% (3.81-4.87)	0.0007 (0.0005- 0.0009)
**Combination of all interventions***			
+ Increasing CB-NAAT use amongst occasional users	27,976,000 (21,331,000 – 40,913,000)	5.17% (4.49-6.17)	N/A

Table S18. Cost efficiency of service provisions in Ahmedabad. We also conducted a sensitivity analysis on the behaviour of newly active providers, and whether increasing the number of active providers remains the most cost-efficient individual intervention if the behaviour of newly active providers is assumed to be 25% and 50% worse than pre-existing active providers. Indeed, even if the behaviour of newly active providers is 50% worse, this intervention is still the most cost-efficient individual intervention when scaled up to its feasible maximum.
*We assume that these newly active providers adopt average behaviour.

For peer review only

Intervention	Incremental cost (2020-2035, USD)	Percentage of cumulative TB cases averted (2020-2035)	Cost-efficiency
**Individual interventions**			
Increasing the number of active providers (assuming newly active providers adopt average behaviour)	20,760,000 (12,478,000 – 36,094,000)	3.06% (2.43-3.98)	0.0003 (0.0002- 0.0006)
Increasing CB-NAAT use amongst occasional users	22,249,000 (13,546,000 – 30,724,000)	1.11% (0.97-1.27)	0.0001 (0.0001- 0.0002)
Increasing the number of occasional CB-NAAT users	4,920,900 (2,985,500 – 6,819,600)	0.86% (0.73-1.03)	0.0004 (0.0003- 0.0006)
Increasing the number of FDC users	4,496,400 (3,283,400 – 5,866,200)	1.13% (0.97-1.34)	0.0006 (0.0004- 0.0007)
**Combination of the two most cost-efficient interventions: Increasing the number of FDC-users + …**			
+Increasing the number of active providers	30,981,000 (21,744,000-46,622,000)	3.80% (2.92-5.06)	0.00026 (0.00017- 0.0046)
+Increasing the number of occasional CB-NAAT users	9,250,000 (6,796,000 – 12,272,000)	1.21% (1.05-1.41)	0.00028 (0.00021-0.00038)
+ Increasing CB-NAAT use amongst occasional users	26,794,000 (17,716,000 – 36,310,000)	1.47% (1.29-1.72)	0.00012 (0.00008-0.00017)
**Combination of the three most cost-efficient interventions: Increasing the number of FDC users + increasing the number of occasional CB-NAAT users + …**			
+ Increasing the number of active providers	41,648,000 (30,441,000 - 60,488,000)	3.95% (3.05-5.22)	0.00020 (0.00013 – 0.00033)
+ Increasing CB-NAAT use amongst occasional users	60,238,000 (37,969,000 – 82,081,000)	1.96% (1.64-2.35)	0.00007 (0.00005 – 0.00010)
**Combination of all interventions**			
+ Increasing CB-NAAT use amongst occasional users	157,450,000 (100,600,000 – 213,200,000)	5.35% (4.30-7.00)	N/A

Table S19. Cost efficiency of service provisions in Delhi. *We assume that these newly active providers adopt average behaviour.

Cost category	Baseline	Increasing no. of active providers	Increasing CB-NAAT usage amongst occasional CB-NAAT users	Increasing the no. of occasional CB-NAAT users	Increasing the no. of providers offering FDCs
**Provider retention**	1.2 million	1.5 million	1.2 million	1.2 million	1.2 million
**Onboarding engagement**	N/A	2,000	N/A	N/A	N/A
**CB-NAAT engagement**	12,000	15,000	0.3 million	14,000	12,000
**FDC engagement**	11,000	14,000	11,000	11,000	17,000
**CB-NAAT logistics**	6.0 million (4.6 – 8.9 million)	7.6 million (5.7 – 11.0 million)	15.0 million (12.2 – 23.8 million)	8.5 million (6.4 – 12.7 million)	6.0 million (4.7 – 8.9 million)
**Adherence support**	0.1 million (0.1 – 0.2 million)	0.2 million (0.1 – 0.3 million)	0.1 million (0.1 – 0.2 million)	0.1 million (0.1 – 0.2 million)	0.1 million (0.1 – 0.2 million)
**FDCs**	5.5 million (3.2 – 9.0 million)	6.7 million (4.1 – 11.0 million)	4.3 million (2.7 – 7.0 million)	5.2 million (3.1 – 8.4 million)	7.4 million (4.9 – 11.6 million)
**Public sector diagnoses**	35.0 million (26.6 – 50.4 million)	35.0 million (25.2 – 50.4 million)	35.0 million (25.2 – 50.4 million)	35.0 million (26.6 – 50.4 million)	35.0 million (26.6 – 50.4 million)
**Public sector treatment**	68.6 million (53.2– 93.8 million)	68.6 million (53.2– 92.4 million)	68.6 million (53.2– 93.8 million)	68.6 million (53.2– 93.8 million)	68.6 million (53.2– 93.8 million)

Table S20. Breakdown of total costs across each intervention between 2020 and 2035 for Ahmedabad.

For peer review only

Cost category	Baseline	Increasing no. of active providers	Increasing CB-NAAT usage amongst occasional CB-NAAT users	Increasing the no. of occasional CB-NAAT users	Increasing the no. of providers offering FDCs
**Provider retention**	3.5 million	8.0 million	3.5 million	3.5 million	3.5 million
**Onboarding engagement**	N/A	14,000	N/A	N/A	N/A
**CB-NAAT engagement**	22,000	50,000	0.3 million	32,000	22,000
**FDC engagement**	18,000	41,000	18,000	18,000	32,000
**CB-NAAT logistics**	8.1 million (4.9 – 11.0 million)	18.0 million (11.2 – 25.2 million)	32.2 million (19.6 – 44.8 million)	13.5 million (8.3 – 18.2 million)	8.1 million (4.9 – 11.0 million)
**Adherence support**	0.3 million (0.2 – 0.4 million)	0.6 million (0.3 – 0.9 million)	0.2 million (0.1 – 0.4 million)	0.3 million (0.2 – 0.4 million)	0.3 million (0.2 – 0.4 million)
**FDCs**	10.2 million (5.3 – 19.6 million)	23.8 million (11.9 – 44.8 million)	7.7 million (4.2 – 15.4 million)	9.7 million (5.0 – 19.6 million)	15.4 million (9.8 – 25.2 million)
**Public sector diagnoses**	117.6 million (74.2 – 154 million)	116.2 million (72.8 – 154 million)	116.2 million (72.8 – 154 million)	117.6 million (74.2 – 154 million)	117.6 million (74.2 – 154 million)
**Public sector treatment**	224 million (168 million – 294 million)	224 million (168 million – 294 million)	224 million (168 million – 294 million)	224 million (168 million – 294 million)	224 million (168 million – 294 million)

Table S21. Breakdown of total costs across each intervention between 2020 and 2035 for Delhi.

For peer review only

Intervention	Maintenance costs sensitivity analysis	Incremental cost (2020-2035, USD)	Percentage of cumulative TB cases averted (2020-2035)	Cost-efficiency
Increasing the number of active providers	*-20%:*	1,963,400 (1,170,200-3,416,300)	3.00% (2.59-3.37)	0.0037 (0.0021-0.0063)
	+20%	2,575,300 (1,782,200-4,028,200)		0.0028 (0.0017-0.0041)
Increasing the number of occasional CB-NAAT users	*-20%:*	1,965,900 (1,440,400-3,030,200)	2.32% (1.97-2.67)	0.0028 (0.0020-0.0038)
	+20%	2,455,700 (1,930,200-3,520,000)		0.0023 (0.0017-0.0029)
Increasing CB-NAAT use amongst occasional users	*-20%:*	8,743,000 (6,671,000-13,031,000)	2.83% (2.42-3.33)	0.0008 (0.0006-0.0010)
	+20%	9,232,000 (7,160,000-13,521,000)		0.0007 (0.0006-0.0009)
Increasing the number of FDC users	*-20%:*	1,541,800 (1,194,000-2,260,900)	2.53% (2.13-2.91)	0.0039 (0.0026-0.0050)
	+20%	2,031,600 (1,683,800-2,750,700)		0.0027 (0.0022-0.0036)

Table S22. Sensitivity analysis on the cost of maintenance in Ahmedabad. We explored whether increasing or decreasing maintenance cost by 20% effected the cost-efficiency order of individual service provisions. Maintenance costs include the cost of provider retention, the cost of CB-NAAT engagement to maintain CB-NAAT use, and the cost of FDC engagement to maintain FDC use.

For peer review only

Intervention	Maintenance costs sensitivity analysis	Incremental cost (2020-2035, USD)	Percentage of cumulative TB cases averted (2020-2035)	Cost-efficiency
Increasing the number of active providers	*-20%:*	19,145,000 (10,865,000 – 34,481,000)	3.06% (2.43-3.98)	0.0003 (0.0002-0.0007)
	+20%	22,375,000 (14,095,000 – 37,712,000)		0.0003 (0.0002 – 0.0006)
Increasing the number of occasional CB-NAAT users	*-20%:*	4,205,900 (2,272,200 – 6,104,300)	0.86% (0.73-1.03)	0.0004 (0.0003-0.0008)
	+20%	5,638,400 (3,704,700 – 7,536,800)		0.0003 (0.0002-0.0005)
Increasing CB-NAAT use amongst occasional users	*-20%:*	21,455,000 (12,823,000 – 29,889,000)	1.11% (0.97-1.27)	0.0001 (0.0001-0.0002)
	+20%	22,887,000 (14,255,000–31,321,000)		0.0001 (0.0001-0.0002)
Increasing the number of FDC users	*-20%:*	3,782,600 (2,569,100 – 5,154,300)	1.13% (0.97-1.34)	0.0007 (0.0005-0.0009)
	+20%	5,215,100 (4,001,600 – 6,586,800)		0.0005 (0.0004-0.0006)

Table S23. Sensitivity analysis on the cost of maintenance in Delhi. We explored whether increasing or decreasing maintenance cost by 20% effected the cost-efficiency order of individual service provisions. Maintenance costs include the cost of provider retention, the cost of CB-NAAT engagement to maintain CB-NAAT use, and the cost of FDC engagement to maintain FDC use.

For peer review only

4. Bibliography

1. Mistry N, Lobo E, Shah S, Rangan S, Dholakia Y. Pulmonary Tuberculosis in Patna, India: Durations, Delays, and Health Care Seeking Behaviour Among Patients Identified Through Household Surveys. *Journal of Epidemiology and Global Health*. 2017;7(4): 241–248.

2. Veesa KS, John KR, Moonan PK, Kaliappan SP, Manjunath K, Sagili K, et al. Diagnostic Pathways and Direct Medical Costs Incurred by New Adult Pulmonary Tuberculosis Patients Prior to Anti-Tuberculosis Treatment - Tamil Nadu, India. *PLOS ONE*. 2018;13(2): e0191591.

3. Pandey A, Ploubidis GB, Clarke L, Dandona L. Hospitalisation Trends in India From Serial Cross-Sectional Nationwide Surveys: 1995 to 2014. *BMJ Open*. 2017;7(12): e014188.

4. Sachdeva KS, Satyanarayana S, Dewan P, Nair SA, Reddy R, Kundu D, et al. Source of Previous Treatment for Re-Treatment TB Cases Registered under the National TB Control Programme, India, 2010. *PLOS ONE*. 2011;6(7): e22061.

5. Satyanarayana S, Nair SA, Chadha SS, Shivashankar R, Sharma G, Yadav S, et al. From Where Are Tuberculosis Patients Accessing Treatment in India? Results From a Cross-Sectional Community Based Survey of 30 Districts. *PLOS ONE*. 2011;6(9): e24160.

6. Sharma G, Sinha A, Bastian S, Santha T. Knowledge and behaviour of chest symptomatics in urban slum populations of two states in India towards care-seeking. *The Indian Journal of Tuberculosis*. 2013;60(2): 95–106.

7. Satyanarayana S, Subbaraman R, Shete P, Gore G, Das J, Cattamanchi A, et al. Quality of Tuberculosis Care in India: A Systematic Review. *International Journal of Tuberculosis and Lung Disease*. 2015;19(7): 751–763.

8. World Health Organisation. *A brief history of tuberculosis control in India.* 2010. https://apps.who.int/iris/bitstream/handle/10665/44408/9789241500159_eng.pdf?sequence=1

9. Menzies D, Benedetti A, Paydar A, Martin I, Royce S, Pai M, et al. Effect of Duration and Intermittency of Rifampin on Tuberculosis Treatment Outcomes: A Systematic Review and Meta-Analysis. *PLOS Med*. 2009;6(9): e1000146. https://doi.org/10.1371/journal.pmed.1000146.

10. Vynnycky E, Fine PE. The natural history of tuberculosis: the implications of age-dependent risks of disease and the role of reinfection. *Epidemiology and Infection*. 1997;119(2): 183–201.

11. Dye C, Garnett GP, Sleeman K, Williams BG. Prospects for worldwide tuberculosis control under the WHO DOTS strategy. Directly observed short-course therapy. *Lancet (London, England)*. 1998;352(9144): 1886–1891.

12. Horsburgh CR, O'Donnell M, Chamblee S, Moreland JL, Johnson J, Marsh BJ, et al. Revisiting rates of reactivation tuberculosis: a population-based approach. *American Journal of Respiratory and Critical Care Medicine*. 2010;182(3): 420–425. https://doi.org/10.1164/rccm.200909-1355OC.

13. Thomas A, Gopi PG, Santha T, Chandrasekaran V, Subramani R, Selvakumar N, et al. Predictors of relapse among pulmonary tuberculosis patients treated in a DOTS programme in South India. *International Journal of Tuberculosis and Lung Disease*. 2005;9(5): 556–561.

14. Tiemersma EW, van der Werf MJ, Borgdorff MW, Williams BG, Nagelkerke NJD. Natural history of tuberculosis: duration and fatality of untreated pulmonary tuberculosis in HIV negative patients: a systematic review. *PloS One*. 2011;6(4): e17601. https://doi.org/10.1371/journal.pone.0017601.

15. Andrews JR, Noubary F, Walensky RP, Cerda R, Losina E, Horsburgh CR. Risk of progression to active tuberculosis following reinfection with Mycobacterium tuberculosis. *Clinical Infectious Diseases*. 2012;54(6): 784–791.

16. World Bank. *Data: India*. 2015 [Accessed 12th May 2016]. http://data.worldbank.org/country/india [Accessed 12th May 2016].

For peer review only

17. Pandey S, Chadha VK, Laxminarayan R, Arinaminpathy N. Estimating tuberculosis incidence from primary survey data: a mathematical modeling approach. *The International Journal of Tuberculosis and Lung Disease*. 2017;21(4): 366–374. https://doi.org/10.5588/ijtld.16.0182.

18. Sachdeva KS, Satyanarayana S, Dewan P, Nair SA, Reddy R, Kundu D, et al. Source of Previous Treatment for Re-Treatment TB Cases Registered under the National TB Control Programme, India, 2010. *PLOS ONE*. 2011;6(7): e22061.

19. Satyanarayana S, Nair SA, Chadha SS, Shivashankar R, Sharma G, Yadav S, et al. From Where Are Tuberculosis Patients Accessing Treatment in India? Results from a Cross-Sectional Community Based Survey of 30 Districts. *PLOS ONE*. 2011;6(9): e24160. https://doi.org/10.1371/journal.pone.0024160.

20. Subbaraman R, Nathavitharana RR, Satyanarayana S, Pai M, Thomas BE, Chadha VK, et al. The Tuberculosis Cascade of Care in India's Public Sector: A Systematic Review and Meta-analysis. *PLOS Medicine*. 2016;13(10): e1002149. https://doi.org/10.1371/journal.pmed.1002149.

21. Van't Hoog A, Langendam MW, Mitchell E, Cobelens FG, Sinclair D, Leeflang MMG, et al. *A sytematic review of the sensitivity and specificity of symptom- and chest-radiography screening for active pulmonary tuberculosis in HIV-negative persons and persons with unknown HIV status*. 2013. https://www.who.int/tb/Review2Accuracyofscreeningtests.pdf

22. Steingart KR, Horne DJ, Pai M, Boehme CC, Dendukuri N. Xpert MTB/RIF assay for pulmonary tuberculosis and rifampicin resistance in adults (Review). *Cochrane Database of Systematic Reviews*. 2014;(1).

23. World Health Organisation. *Global Tuberculosis Report 2020*. 2020. https://www.who.int/publications/i/item/9789240013131

24. Subbaraman R, Nathavitharana RR, Mayer KH, Satyanarayana S, Chadha VK, Arinaminpathy N, et al. Constructing care cascades for active tuberculosis: A strategy for program monitoring and identifying gaps in quality of care. *PLOS Medicine*. 2019;16(2): e1002754.

25. Menzies NA, Gomez GB, Bozzani F, Chatterjee S, Foster N, Baena IG, et al. Cost-effectiveness and resource implications of aggressive action on tuberculosis in China, India, and South Africa: a combined analysis of nine models. *The Lancet Global Health*. 2016;4(11): e816–e826. https://doi.org/10.1016/S2214-109X(16)30265-0.

26. Houben RMGJ, Lalli M, Kranzer K, Menzies NA, Schumacher SG, Dowdy DW. What if they don't have tuberculosis? The consequences and trade-offs involved in false-positive diagnoses of tuberculosis. *Clinical Infectious Diseases*. 2019;68(1): 150–156. https://doi.org/10.1093/cid/ciy544.

27. Fochsen G, Deshpande K, Diwan V, Mishra A, Diwan VK, Thorson A. Health care seeking among individuals with cough and tuberculosis: a population-based study from rural India. *International Journal of Tuberculosis and Lung Disease*. 2006;10(9): 995–1000.

28. Gopi PG, Subramani R, Narayanan PR. Evaluation of different types of chest symptoms for diagnosing pulmonary tuberculosis cases in community surveys. *Indian Journal of Tuberculosis*. 2008;55(3): 116–121.

29. Rao VG, Bhat J, Yadav R, Gopalan GP, Nagamiah S, Bhondeley MK, et al. Prevalence of pulmonary tuberculosis - a baseline survey in central India. *PLOS One*. 2012;7(8): e43225. https://doi.org/10.1371/journal.pone.0043225.

30. Sachdeva KS, Raizada N, Sreenivas A, Van't Hoog A, van den Hof S, Dewan PK, et al. Use of Xpert MTB/RIF in decentralized public health settings and its effect on pulmonary TB and DR-TB case findings in India. *PLOS ONE*. 2015;10(5): e0126065. https://doi.org/10.1371/journal.pone.0126065.

31. Haario H, Saksman E, Tamminen J. An adaptive metropolis algorithm. *Bernoulli*. 2001;7(2): 223–242.

For peer review only

For peer review only

CHEERS 2022 Checklist

	Item	Guidance for Reporting	Reported in section
**TITLE**			
Title	1	Identify the study as an economic evaluation and specify the interventions being compared.	N/A
**ABSTRACT**			
Abstract	2	Provide a structured summary that highlights context, key methods, results and alternative analyses.	Page 1
**INTRODUCTION**			
Background and objectives	3	Give the context for the study, the study question and its practical relevance for decision making in policy or practice.	Page, 1-2
**METHODS**			
Health economic analysis plan	4	Indicate whether a health economic analysis plan was developed and where available.	Page 5-6
Study population	5	Describe characteristics of the study population (such as age range, demographics, socioeconomic, or clinical characteristics).	Table 1-2
Setting and location	6	Provide relevant contextual information that may influence findings.	Table 1and page 3-4
Comparators	7	Describe the interventions or strategies being compared and why chosen.	Page 6
Perspective	8	State the perspective(s) adopted by the study and why chosen.	Page 5
Time horizon	9	State the time horizon for the study and why appropriate.	Page 5
Discount rate	10	Report the discount rate(s) and reason chosen.	Page 6
Selection of outcomes	11	Describe what outcomes were used as the measure(s) of benefit(s) and harm(s).	Page 6
Measurement of outcomes	12	Describe how outcomes used to capture benefit(s) and harm(s) were measured.	Page 6
Valuation of outcomes	13	Describe the population and methods used to measure and value outcomes.	N/A
Measurement and valuation of resources and costs	14	Describe how costs were valued.	Table S8-13
Currency, price date, and conversion	15	Report the dates of the estimated resource quantities and unit costs, plus the currency and year of conversion.	Table S8
Rationale and description of model	16	If modelling is used, describe in detail and why used. Report if the model is publicly available and where it can be accessed.	Model is fully specified in the manuscript materials
Analytics and assumptions	17	Describe any methods for analysing or statistically transforming data, any extrapolation methods, and approaches for validating any model used.	Figure S1, table S10, page 6
Characterizing heterogeneity	18	Describe any methods used for estimating how the results of the study vary for sub-groups.	Page 6-7
Characterizing distributional effects	19	Describe how impacts are distributed across different individuals or adjustments made to reflect priority populations.	N/A
Characterizing uncertainty	20	Describe methods to characterize any sources of uncertainty in the analysis.	Page 6-7
Approach to engagement with patients and others affected by the study	21	Describe any approaches to engage patients or service recipients, the general public, communities, or stakeholders (e.g., clinicians or payers) in the design of the study.	N/A
**RESULTS**			
Study parameters	22	Report all analytic inputs (e.g., values, ranges, references) including uncertainty or distributional assumptions.	Table 1-2
Summary of main results	23	Report the mean values for the main categories of costs and outcomes of interest and summarise them in the most appropriate overall measure.	Table 3, S14-15
Effect of uncertainty	24	Describe how uncertainty about analytic judgments, inputs, or projections affect findings. Report the effect of choice of discount rate and time horizon, if applicable.	Page 9-10, fig S6-8
Effect of engagement with patients and others affected by the study	25	Report on any difference patient/service recipient, general public, community, or stakeholder involvement made to the approach or findings of the study	N/A
**DISCUSSION**			
Study findings, limitations, generalizability, and current knowledge	26	Report key findings, limitations, ethical or equity considerations not captured, and how these could impact patients, policy, or practice.	Page 8
**OTHER RELEVANT INFORMATION**			
Source of funding	27	Describe how the study was funded and any role of the funder in the identification, design, conduct, and reporting of the analysis	
Conflicts of interest	28	Report authors conflicts of interest according to journal or International Committee of Medical Journal Editors requirements.	

Husereau D, Drummond M, Augustovski F, de Bekker-Grob E, Briggs AH, Carswell C, Caulley L, Chaiyakunapruk N, Greenberg D, Loder E, Mauskopf J, Mullins CD, Petrou S, Pwu RF, Staniszewska S; CHEERS 2022 ISPOR Good Research Practices Task Force. Consolidated Health Economic Evaluation Reporting Standards 2022 (CHEERS 2022) Statement: Updated Reporting Guidance for Health Economic Evaluations. BMJ. 2022;376:e067975.
The checklist is Open Access distributed in accordance with the terms of the Creative Commons Attribution (CC BY 4.0) license, which permits others to distribute, remix, adapt and build upon this work, for commercial use, provided the original work is properly cited. See: http://creativecommons.org/licenses/by/4.0/.

For peer review only

BMJ Open

Operational priorities for engaging with India's private healthcare sector for the control of tuberculosis: a modelling study

Journal:	*BMJ Open*
Manuscript ID	bmjopen-2022-069304.R2
Article Type:	Original research
Date Submitted by the Author:	13-Dec-2023

Complete List of Authors:	Ricks, Saskia ; Imperial College London,<br>Singh, Ananya; Clinton Health Access Initiative<br>Sodhi, Ridhima; Clinton Health Access Initiative<br>Pal, Arnab; Clinton Health Access Initiative<br>Arinaminpathy, Nimalan; Imperial College London School of Public Health

**Primary Subject Heading**:	Epidemiology
Secondary Subject Heading:	Health economics
Keywords:	Tuberculosis < INFECTIOUS DISEASES, Epidemiology < INFECTIOUS DISEASES, HEALTH ECONOMICS

SCHOLARONE™
Manuscripts

I, the Submitting Author has the right to grant and does grant on behalf of all authors of the Work (as defined in the below author licence), an exclusive licence and/or a non-exclusive licence for contributions from authors who are: i) UK Crown employees; ii) where BMJ has agreed a CC-BY licence shall apply, and/or iii) in accordance with the terms applicable for US Federal Government officers or employees acting as part of their official duties; on a worldwide, perpetual, irrevocable, royalty-free basis to BMJ Publishing Group Ltd ("BMJ") its licensees and where the relevant Journal is co-owned by BMJ to the co-owners of the Journal, to publish the Work in this journal and any other BMJ products and to exploit all rights, as set out in our licence.

The Submitting Author accepts and understands that any supply made under these terms is made by BMJ to the Submitting Author unless you are acting as an employee on behalf of your employer or a postgraduate student of an affiliated institution which is paying any applicable article publishing charge ("APC") for Open Access articles. Where the Submitting Author wishes to make the Work available on an Open Access basis (and intends to pay the relevant APC), the terms of reuse of such Open Access shall be governed by a Creative Commons licence – details of these licences and which Creative Commons licence will apply to this Work are set out in our licence referred to above.

Other than as permitted in any relevant BMJ Author's Self Archiving Policies, I confirm this Work has not been accepted for publication elsewhere, is not being considered for publication elsewhere and does not duplicate material already published. I confirm all authors consent to publication of this Work and authorise the granting of this licence.

For peer review only

Operational priorities for engaging with India's private healthcare sector for the control of tuberculosis: a modelling study

Saskia Ricks*[1], Ananya Singh[2], Ridhima Sodhi[2], Arnab Pal[2] and Nimalan Arinaminpathy[1]

***Corresponding author**; saskia.ricks12@imperial.ac.uk

[1]Imperial College London, Praed Street, London, W2 1NY, UK

[2]Clinton Health Access Initiative, New Delhi, India

For peer review only

Key words: tuberculosis, epidemiology, health economics

Word count: 4670

Abstract

Objectives: To estimate the potential impact of expanding services offered by the Joint Effort for Elimination of Tuberculosis ("JEET"), the largest private sector engagement initiative for tuberculosis (TB) in India.

Design: We developed a mathematical model of TB transmission dynamics, coupled with a cost model.

Setting: Ahmedabad and New Delhi, two cities with contrasting levels of JEET coverage.

Participants: Estimated patients with TB in Ahmedabad and New Delhi.

Interventions: We investigated the epidemiological impact of expanding three different PPSA services: provider recruitment, uptake of CB-NAAT (cartridge-based nucleic acid amplification tests) and uptake of adherence support mechanisms (specifically government supplied fixed-dose combination drugs), all compared to a continuation of current TB services.

Results: Our results suggest that in Delhi, increasing the use of adherence support mechanisms amongst private providers should be prioritised, having the lowest incremental cost-per-case-averted between 2020 and 2035 of $170,000 (110,000 – 310,000). Likewise in Ahmedabad, increasing provider recruitment should be prioritised, having the lowest incremental cost-per-case averted of $18,000 (12,000-29,000).

Conclusion: Results illustrate how intervention priorities may vary in different settings across India, depending on local conditions, and the existing degree of uptake of PPSA services. Modelling can be a useful tool for identifying these priorities for any given setting.

Strengths and limitations

- We have used detailed programmatic data to inform costs, effectiveness, and real-world coverage of the different interventions discussed in this analysis to help identify operational priorities.
- However, due to a lack of city-specific data for Delhi and Ahmedabad, the mathematical model was instead calibrated to prevalence and infection surveys from Chennai, another large Indian city.
- Due to data gaps in healthcare provider behaviour, we made assumptions informed partly by expert opinion, to estimate how this behaviour is likely to change through engagement efforts.

Introduction

Tuberculosis (TB) is an airborne infectious disease (1) and a major cause of death worldwide, responsible for 10.6 million cases and 1.6 million deaths in 2021 (2). India accounts for over a

For peer review only - http://bmjopen.bmj.com/site/about/guidelines.xhtml

For peer review only

quarter of global TB burden, with 2.9 million cases in 2021 (2). A major challenge in India is the presence of a vast, fragmented private healthcare sector, which manages the majority of TB patients in the country (3–5). The past decade has seen increasing recognition of the importance of engaging effectively with this sector (6–10): not only in notifying TB to public health authorities, but also in offering high-quality diagnostics and treatment adherence support for patients being managed by private providers. Early efforts to engage effectively with the private sector faced substantial challenges, principally arising from a lack of trust (11). Despite TB being made a notifiable disease in 2012 in India (12) few private providers reported their TB patients to public health authorities. However, from 2013 onwards, pilots of 'public-private support agencies' (PPSAs) showed how private providers could be successfully engaged, through supporting private providers to notify TB, as well as making high-quality diagnostics and treatment support available for patients being managed by the private sector (7,9).

To take the PPSA model to scale across the country, the Joint Effort for Elimination of Tuberculosis (JEET) was launched by 3 partner organisations (the Clinton Health Access Initiative; Centre for Health, Research and Innovation; and Foundation for Innovative New Diagnostics) in 2018 (13). Supported by the Global Fund and coordinated by the Central TB Division of India, it is the largest private sector engagement initiative for TB in India to date (14). The main activities include increasing case notifications by engaging with private providers, improving the quality of TB diagnosis by providing free access to molecular diagnostics, and improving treatment outcomes by providing free fixed dose combination (FDCs) and adherence support (13). By, 2020 JEET was operating in 485 cities across India (13); these developments accompanied a substantial increase in notifications from private providers, accounting in 2022 for around 30% of total TB notifications in India (15).

Despite these achievements, there remains much ground to be covered. Moreover, progress in PPSA implementation has varied across the country, with greater uptake of available services in some cities than in others. One important question is what kinds of PPSA services should be promoted in any given setting, depending on what levels of uptake have already been achieved. In particular, in a given city, should future efforts for strengthening PPSA efforts place greater priority on increasing the uptake of molecular diagnostics, or of adherence support mechanisms? Here, we aimed to address this question using mathematical models of TB transmission, to identify the most cost-effective strategies for future PPSA expansion.

Method

Model overview

As two examples we focused on the urban settings, Ahmedabad and Delhi, with contrasting levels of uptake of PPSA services. Ahmedabad has population of around 7.7 million and is the largest city in the Indian state of Gujarat; Delhi has a population of around 33 million and is the capital city of the

For peer review only - http://bmjopen.bmj.com/site/about/guidelines.xhtml

country. PPSA operations in both cities began in 2018 (16), and have followed different trajectories with, for example, services in Delhi seeing substantially less uptake of fixed dose combinations (FDCs) than Ahmedabad. In the present analysis, therefore, they provide helpful examples of sites that may face different priorities in their future expansion of PPSA efforts.

We developed a deterministic, compartmental model of TB transmission amongst adults consistent with an urban setting in India (fig 1). The model captures routine TB services, including the scale-up of the Revised National TB Control Programme (RNTCP) between 1997 and 2007 (17), and the scale-up of private sector engagement from 2017 onwards (18). We incorporated the acquisition and transmission of drug-resistant TB (DR-TB), including both rifampicin-resistant and multi-drug resistant TB. For simplicity, we ignored HIV status, and the distinction between pulmonary and extrapulmonary TB, although we conducted a sensitivity analysis on the latter. We separately simulated a non-TB symptomatic population (fig 1) to count the overall number of TB diagnoses and false positive treatments amongst presumptives mistakenly diagnosed as having TB. This population has no influence on the 'main' model of TB transmission dynamics, and its purpose is only to quantify volumes of diagnostic tests and treatments. As implemented in previous work, we calibrated the size of the non-TB symptomatic population to give a 10% prevalence of TB amongst those presenting for care, in either the public or private sector (19–21). The supplementary information provides further details on the model, including governing equations and parameter values (table S1-table S7), and a breakdown of the proportion of patients visiting different providers for diagnosis and treatment (table S8- table S9, respectively).

Data sources

To reflect a typical urban setting in India (such as Ahmedabad and Delhi), we used survey data of adults (> 15 years old) for the annual risk of TB infection (ARTI) and the prevalence of TB (22,23). Table 1 summarises the data from these sources. Table S2- table S5 list parameter values used in the model (TB natural history, diagnosis, treatment and costs, respectively).

Indicator	Value	Source
Annual risk of TB infection	2% [1-3]	(22)
TB prevalence	259 per 100,000 [217-299]	(23)
TB mortality	32 per 100,000 [30-34]	(24)
Percent of TB cases that are MDR	4% [3-5]	(24)

Table 1. Calibration targets used to estimate model parameters. We assumed the same prevalence, annual risk of TB infection, TB mortality and percent of TB cases that are MDR across Ahmedabad and Delhi.

For peer review only - http://bmjopen.bmj.com/site/about/guidelines.xhtml

We incorporated uncertainty in parameter values and model inputs through Adaptive Bayesian Markov chain Monte Carlo (MCMC) (25). Thus, the uncertainty in model projections reflects the uncertainty in the model inputs; we refer to the uncertainty in model projections as the Bayesian credible intervals, using the 2.5th and 97.5th percentiles to reflect the lower and upper bound of an interval.

We also drew a range of operational data from CHAI, to inform elements such as existing uptake of JEET services in Ahmedabad and Delhi; provider behaviour over time in response to CHAI engagement efforts; and treatment outcomes under different types of adherence support in the private sector. Further details are provided below ('Modelling existing PPSA services').

Care cascade

We assumed that upon development of active disease, there is a delay (determined by calibration to the available data) before individuals seek care (10,26). We modelled public and private providers separately, assuming: (i) the quality of diagnosis to be lower in the latter than the former, and (ii) once individuals are diagnosed with TB and initiated on treatment, a lower rate of treatment completion in the private sector, due to the general lack of adherence support (27,28). Owing to a lack of systematic data for the private sector when not engaged with a PPSA, we allowed for a wide range of parameter values relating to both diagnosis and treatment in this sector (table S3- table S4). The supplementary information also provides further details on the non-TB symptomatic population care cascade.

Modelling existing PPSA services

Provider recruitment

When commencing operations in any new setting, JEET conducts provider mapping to identify the highest-volume providers that should be prioritised. 'Engagement' with providers is achieved by field officers to encourage providers to notify their TB cases. However, even after these efforts, not all engaged providers notify TB, and require further engagement by field officers to do so, coupled with a strong enforcement of regulations. Accordingly, we distinguished different types of private providers: those who are not engaged; those who are engaged but not notifying TB ('inactive' providers); and those who are notifying TB ('active' providers) (table S10). We define 'active' providers as those notifying TB at least once a month. Active providers may, over time, turn inactive, for three main reasons: first, some providers may not have a sufficient number of TB patients every month and thus may notify infrequently depending on when they see a new TB patient; second, some providers, especially those that handle a smaller volume of TB patients, are seen less regularly by the field team and are therefore less likely to notify; and third, some providers may be dissatisfied with the services offered by JEET, and therefore intentionally stop notifying patients. If 'active' providers are defined as those notifying TB at least once a month, we accounted for this rate of attrition by weighting the amount of CB-NAAT (cartridge-based nucleic acid amplification testing) and FDC issued by the number of months providers are active for.

For peer review only - http://bmjopen.bmj.com/site/about/guidelines.xhtml

For peer review only

The transmission model follows the patient perspective of the careseeking journey; an important parameter relating to PPSA activities is the probability that a patient, when seeking care for their symptoms, visits a private provider amongst the three categories listed above. To estimate these probabilities, we modelled a 'heterogeneity index' α, such that (fig S1, table S11):

$$\text{Proportion of patients visiting an engaged provider} = (\text{Proportion of providers engaged})^{\alpha}$$

Thus, values of $\alpha < 1$ correspond with the situation where high-volume providers are preferentially engaged. We estimated the value of α using data provided by CHAI for both Ahmedabad and Delhi.

TB diagnosis

Field officers engage with active providers to encourage them to offer CB-NAAT to their presumptive TB patients. Specimen collection and transportation (SCT) agents collect the sputum samples from the provider clinic and transport them to laboratories where CB-NAAT testing is performed for free. While efforts are made to encourage active providers to use these services, the services are not mandated under Project JEET, in the interest of a collaborative relationship.

There is a multimodal distribution for the uptake of this CB-NAAT service amongst active private providers, including those who never use it; those who use it occasionally; and those for whom all notifications are CB-NAAT confirmed (fig S2). In 2019, 55% and 66% of active providers in Ahmedabad and Delhi did not use CB-NAAT confirmation when notifying TB. Providers who do not confirm a TB notification with CB-NAAT instead evaluate patients using chest x-ray and clinical judgement (18,19,29). We assumed that inactive and unengaged private providers diagnose TB in the same way. We drew from the literature for the sensitivity and specificity of X-ray and clinical judgement in diagnosing TB (30,31) (table S3).

Motivated by fig S2, we divided active providers into three categories depending on their use of CB-NAAT: providers who always confirm TB notifications with CB-NAAT, providers who occasionally use CB-NAAT, and providers who never use CB-NAAT. Table 2 shows the number of active providers in each of these categories for Ahmedabad and Delhi in 2019. Once active providers become an CB-NAAT user, field officers continue visits to encourage these providers to continue confirming TB diagnoses with CB-NAAT.

	**City**	
	Ahmedabad	*Delhi*
Total no. of active providers	762	894
No. of active providers that always use CB-NAAT	65	35
No. of active providers that occasionally use CB-NAAT	278	265
No. of active providers that never use CB-NAAT	419	594
Percent of notifications that are CB-NAAT confirmed by occasional users	22%	15%

For peer review only - http://bmjopen.bmj.com/site/about/guidelines.xhtml

Table 2. CB-NAAT use amongst active private providers in Ahmedabad and Delhi in 2019. To be classified as occasionally using CB-NAAT, we assumed that providers use CB-NAAT for at least 1% of notifications and less than 100% of notifications. Data provided by CHAI.

TB treatment

JEET offers two different adherence services for TB treatment via treatment coordinators: government supplied fixed-dose combinations (FDCs) and adherence support (table 3). Government supplied FDCs are supplied for free to the patient by the government, whereas patients on adherence support receive regular text messages and calls to ensure treatment adherence. Field officers visit active providers regularly to ensure they are continuing to recommend government supplied FDCs and adherence support.

	City	
	Ahmedabad	*Delhi*
Total no. of active providers	762	894
No. of active providers that offer adherence support	712	835
Percent of patients that agree to adherence support	92%	92%
No. of active providers that agree to government supplied FDCs	529	242
Percent of active providers that consistently use FDCs, unweighted	45%	10%
Percent of active providers that consistently use FDCs, weighted	39%	7%

Table 3. Number of active private providers that offer government supplied FDCs and adherence support in Ahmedabad and Delhi in 2019. Data provided by CHAI, based on existing operations.

We considered 4 categories of active providers in the model: active providers that use government supplied FDCs and offer adherence support, those that use government supplied FDCs but do not offer adherence support, those that offer adherence support but do not use government supplied FDCs, and those that offer neither service. We drew from data provided by CHAI to inform treatment completion rates associated with each of these categories, active providers offering both services having the highest rate of treatment completion and those offering neither having the lowest rate (table S4). Finally, we assumed that inactive and unengaged providers do not offer adherence support and thus patients seeking treatment from these providers have the lowest treatment completion rates.

All patients diagnosed with DR-TB by a private provider are transferred to the public sector and initiated onto the second line treatment.

For peer review only - http://bmjopen.bmj.com/site/about/guidelines.xhtml

Interventions

We focused on the impact of the following four interventions:

- *Activate providers:* increasing the number of active providers amongst those already engaged but not notifying TB
- *Promote CB-NAAT (occasional users)* – increasing CB-NAAT use amongst occasional CB-NAAT users
- *Promote CB-NAAT (non-users)* – encouraging active providers that never use CB-NAAT into occasionally using CB-NAAT,
- *Promote adherence mechanisms* - increasing the use of government supplied FDCs amongst active providers.

We did not examine the impact of increasing adherence support, as current uptake is already high (above 90%) in Ahmedabad and Delhi.

We modelled a 'theoretical maximum coverage' scenario where all providers are amenable to intervention. In practice, however, some providers may remain uninterested in taking up the services offered despite sustained engagement efforts by field officers. To reflect these limits, we also modelled a 'feasible maximum' scenario where some proportion of providers remain unresponsive to the interventions listed above. Informed by expert consultation with CHAI, we assumed that: all inactive providers and occasional CB-NAAT users are interested in notifying and increasing their CB-NAAT use, whereas 40% of non-CB-NAAT users are uninterested in using CB-NAAT, and 35% of non-government supplied FDC users are uninterested in using government supplied FDCs (see fig S3 for more detail).

For both cities, we examined the epidemiological impact of increasing the four activities to their feasible and theoretical maxima, compared to an indefinite continuation of current levels of routine (publicly offered) TB and PPSA services. We modelled the scale-up of existing PPSA activities from 2017 to current levels in 2020. We assumed the interventions are scaled up linearly between 2020 and 2021, and simulated their impact until 2035.

Economic evaluation

We took the programmatic perspective, i.e., the costs that the national TB programme would have to pay. To examine which PPSA service should be prioritised in the future, we calculated the cost of expanding the different services to their feasible and theoretical maximum using operational cost data from 2019 for Ahmedabad and Delhi. Table S12 summarises the total cost in 2019 for the different activities involved in the service provisions offered by JEET. These activities include the salaries of the field officer, treatment coordinators, hub agents, and SCT agents; the cost of continuing medical education (CMEs; seminars organised for providers to increase engagement); the cost of promotional materials which includes the cost of designing, printing and dissemination; the cost of transporting sputum samples to the laboratories, (including the cost of procuring falcon tubes, carrier boxes, masks and hand sanitizer); the cost of CB-NAAT tests, including test cartridges

For peer review only

and manpower; and the cost of government supplied FDCs. We ignored sunk costs (office expenditure and state programme management costs) that have already been incurred in Ahmedabad and Delhi, and thus would not influence decisions for future interventions.

We categorised each cost item listed into table S12 into six different categories: provider activation, provider retention, diagnostic engagement, diagnostic logistics, treatment engagement and treatment logistics (described in further detail in table S13). Table S14 lists assumptions following expert consultation with CHAI, for the level of effort needed to engage providers and achieve increased uptake (subject to uncertainty analysis). If a cost was associated with more than one category, the cost was subdivided across the relevant categories based on estimations from CHAI on the percentage of time spent on each category (table S15). Next, we calculated the per-unit cost of each cost item (table S16-table S17). For example, to calculate the cost of field officers to retain one provider per year, we multiplied the total cost of field officers in 2019, by the percentage of time spent by field officers on provider retention in 2019, divided by the number of active providers in 2019.

In practice, increasing the coverage of intervention activities is achieved through field officer visits, to engage directly with the providers. Finally, we also considered costs of routine TB services provided by the public sector, including the cost of diagnosis and the cost of treating DS- and DR-TB (table S5).

We calculated the incremental cost-effectiveness ratio (ICER) by dividing the incremental cost of increasing the coverage of PPSA services by the number of TB cases averted between 2020 and 2035, relative to a baseline of indefinite continuation of current levels of routine TB and JEET PPSA services.

Sensitivity analyses

We conducted various sensitivity analyses to explore the impact of certain model assumptions on model results. Firstly, we conducted multivariate sensitivity analysis using partial rank correlation coefficient to assess which model parameters are most influential for model outputs.

We also conducted sensitivity analyses on provider behaviour. In the main analysis, we accounted for the fact that active providers may revert to an inactive state, and we assumed that the behaviour of newly active providers is the same as pre-existing active behaviours. First, we considered an optimistic PPSA scale-up baseline that assumes active providers remain active once activated. Second, we assumed that newly active providers have 25% and 50% less uptake than pre-existing active providers (i.e., using Ahmedabad as an example, 9% of pre-existing active providers use CB-NAAT 100% of the time; we modelled scenarios where this proportion is 4.5% and 2.3%). We did not differentiate extrapulmonary and pulmonary TB in the model; however, CB-NAAT is unable to diagnose TB amongst patients with exclusively extrapulmonary disease. Thus, high levels of extrapulmonary TB can reduce the incremental benefit of CB-NAAT testing. In Ahmedabad and Delhi, it is estimated that 32% and 38% of patients diagnosed by JEET PPSA services between 2019 and

For peer review only - http://bmjopen.bmj.com/site/about/guidelines.xhtml

2022 had extrapulmonary TB, respectively. To account for this in a simple way, we reduced the proportion of TB patients presenting to care that are correctly diagnosed by CB-NAAT by 50%. For the purpose of sensitivity analysis, this reflects an extreme scenario where 50% of patients that present to care have extrapulmonary TB.

Finally, we examined the impact of varying maintenance costs by +/- 20% on the ICER of the different interventions. We defined maintenance costs as costs that are associated with maintaining good engagement and uptake amongst active providers (i.e., maintaining notifications, FDC use and CB-NAAT use).

Patient and public involvement

Patients or the public were not involved in the design, or conduct, or reporting, or dissemination plans of our research.

Results

Figs S4-S5 show results of model calibration, displaying the model fit against each of the calibration targets listed in table 1.

Figs 2 shows model projections for the impact on TB incidence between 2017 and 2035 in Ahmedabad and Delhi across varying levels of PPSA coverage, with cumulative impact summarised in table 4. Relative to a comparator where current TB services are maintained, increasing the four PPSA activities simultaneously to their feasible maximum coverage would avert 5.1% (95% CrI 4.4-6.2%) of cumulative TB cases between 2020 and 2035, whereas increasing them to their theoretical maximum would avert 7.4% (95% CrI 6.2-9.2%) of cumulative TB cases between 2020 and 2035 in Ahmedabad. In Delhi, 5.4% (95% CrI 4.4-6.9%) and 6.7% (95% CrI 5.3-8.9%) of cumulative TB cases would be averted under feasible and theoretical maximum coverages, respectively. There would be a larger impact on DR-TB cases and deaths averted as three of the four PPSA activities involved increasing the use of CB-NAAT, thus increasing the early detection of rifampicin resistance. For example, assuming PPSA activities are increased to their theoretical maximum coverage 14% (95% CrI 11-16%) and 35% (95% CrI 30-39%) of cumulative DR-TB cases and deaths would be averted between 2020 and 2035, respectively. This impact increases to 18% (95% CrI 15-22%) and 41% (95% CrI 35-47%) DR-TB cases and deaths averted in Delhi, respectively, due to the lower current levels of CB-NAAT uptake in Delhi compared to Ahmedabad.

Percent reduction in cumulative TB burden, 2020-2035	**Ahmedabad**	**Delhi**
**Feasible maximum**		
TB incidence	5.1% (4.4-6.2)	5.4% (4.4-6.9)
TB deaths	4.1% (2.8-5.8)	6.4% (4.9-8.2)
DR-TB incidence	8.0% (6.2-9.7)	14% (11-17)

For peer review only - http://bmjopen.bmj.com/site/about/guidelines.xhtml

DR-TB deaths averted	20% (18-24)	32% (27-37)
**Theoretical maximum**		
TB incidence	7.4% (6.2-9.2)	6.7% (5.3-8.9)
TB deaths	7.9% (5.7-11)	8.7% (6.8-11.3)
DR-TB incidence	14% (11-16)	18% (15-22)
DR-TB deaths averted	35% (30-39)	41% (35-47)

Table 4. Projected epidemiological impact. Impact is measured as the cumulative impact on TB cases and deaths in Ahmedabad and Delhi between 2020 and 2035 relative to a baseline of current PPSA scale up.

In Ahmedabad, the interventions with the lowest ICERS were provider activation and promoting FDC use, costing an estimated $18,000 (12,000-29,000) and $36,000 (26,000-56,000) per TB case averted between 2020 and 2035, assuming the interventions were scaled up to their feasible maximum. Despite their low ICERs, these interventions were also the least epidemiologically impactful (table S18; fig 3). The interventions with the highest ICERS were those promoting CB-NAAT, costing $120,000 (96,000-180,000) and $46,000 (37,000-68,000) per TB case averted depending on whether CB-NAAT use was promoted amongst occasional users and non-users, respectively. For Delhi, promoting FDC use also had the lowest ICER costing an estimated $170,000 (110,000-310,000) per TB case averted between 2020 and 2035 (table S19, fig 3). However, promoting the use of CB-NAAT amongst non-users and provider activation had similar ICERs at $210,000 (150,000-320,000) and $280,000 (200,000-400,000) per TB case averted, respectively. Similar to Ahmedabad, promoting the use of CB-NAAT amongst occasional users had the highest ICER at $780,000 (500,000-900,000) per TB case averted. Fig 3 summarises the order in which the different interventions should be prioritised in Ahmedabad and Delhi, and table S20-table S21 provide a breakdown of the total costs.

Figures S6 – S8 show results of sensitivity analyses. Alternate assumptions for the proportion of active providers that remain consistently active and the proportion of TB patients with exclusively extrapulmonary TB reduced the epidemiological impact of increasing PPSA service provisions (fig S6) The qualitative findings shown in fig 3 were unchanged by alternative assumptions on the uptake of CB-NAAT and treatment adherence amongst newly recruited providers (table S18). However, assumptions relating to maintenance costs (the cost associated with maintaining good behaviour amongst active providers) showed some influence on the prioritisation of interventions. For Ahmedabad, decreasing maintenance cost by 20% resulted in the promotion of government supplied FDCs being the priority intervention (table S22). On the other hand, increasing the proportion of active providers remained the individual intervention with the lowest ICER if maintenance cost increased by 20%. For Delhi, increasing the proportion of FDC users remained the intervention with the lowest ICER regardless of a 20% increase or decrease in maintenance cost (table S23).

For peer review only

Finally, we conducted multivariate sensitivity analysis to identify the parameters that are most influential for model outputs. Model projections for TB cases averted in Ahmedabad were influenced most strongly by the following three parameters: the rate of relapse following self-cure, the rate of stabilisation of the risk of relapse following treatment, and the rate of repeat care-seeking (fig S7). For Delhi, the three most influential parameters were also the rate of relapse following self-cure and the rate of stabilisation of the risk of relapse following treatment, as well as the rate of progression to active disease from latency (fig S8).

Discussion

Our analysis focuses on settings that have already seen an introduction of JEET services and addresses how these services should be expanded in future, to achieve reductions in TB burden in the most cost-effective way. Given the wide variation in epidemiology and health services across different JEET locations, it is critical for any such analysis to be context-specific, taking account of local data. Our work illustrates how mathematical models can be helpful in doing so, by offering a tool for analysis of programmatic data. Focusing on Ahmedabad and Delhi, two cities with different coverage levels of PPSA services, our analysis suggests that JEET operations should prioritise working with already-engaged providers to encourage them to participate more actively in Ahmedabad and increasing the use of government supplied FDCs amongst already-active providers in Delhi. Similar modelling can be applied to identify priorities for future operations in other settings.

Previous modelling work estimated the potential epidemiological impact of scaling up PPSA operations in Mumbai and Patna, the cities where the PPSA pilots were first implemented (10). Subsequent analysis used a relatively simple costing model to illustrate how PPSA scaleup would be cost-effective in both settings (7). These findings echoed earlier analysis that similarly showed how public-private mix in India would be cost-effective (32). However, all these analyses were performed prospectively, and thus we had to make assumptions about the effectiveness of different interventions, in cities where PPSA operations did not already exist at scale. Now that JEET has been operating at scale in India, there is detailed programmatic data available for the costs, effectiveness, and real-world coverage of different interventions: our analysis thus adds to previous work by analysing this data in a systematic way, to identify operational priorities for future expansion of PPSA activities, in given cities.

Overall, the interventions with the highest ICERs are those that involve CB-NAAT. Although these interventions are epidemiologically impactful, they are costly, due to the high cost of an CB-NAAT test; the cost of testing symptomatic non-TB infected care-seekers; and the cost of engaging with the active providers to increase their use of CB-NAAT. The cost data used in this analysis suggests that reducing the effort needed to convince private providers to increase CB-NAAT use will be key in decreasing the overall cost. A recent study found that larger providers may be more willing to change their behaviour and adopt the use of CB-NAAT compared to smaller providers; thus,

For peer review only - http://bmjopen.bmj.com/site/about/guidelines.xhtml

targeting these larger providers may provide a more cost-efficient solution and help bring down the cost of increasing the use of CB-NAAT amongst private providers (8). On the other hand, promoting uptake of FDCs – although having modest impact – also consistently showed the lowest estimated ICERs, because of its low cost.

The epidemiological impact is likely to be reduced if there is a high rate of attrition from an active to an inactive status amongst private providers. There are several reasons behind this attrition: first, some providers may not have a sufficient load of TB patients every month and thus may notify infrequently depending on when they see a new TB patient; second, some providers, especially those handling a smaller volume of TB patients, are seen less regularly by the field team and are therefore less likely to notify; third, some providers remain dissatisfied with the services offered by JEET, and therefore intentionally stop notifying patients; fourth, providers may be wary of the scrutiny they receive once they become active and involved with the programme. Understanding the reasons behind this behaviour will be important to ensure engagement efforts do not go to waste.

Limitations of this work can be divided into two categories: transmission model limitations and economic limitations. Owing to a lack of city-specific data, we drew from prevalence and infection surveys from Chennai, another large Indian city. This analysis focusses on urban settings and does not address rural settings, that may have different care-seeking pathways, different rates of TB infection and different provider behaviour (3,19). While JEET activities have so far focused on urban India, addressing needs for private sector engagement in rural settings is an important area for future work. Another area for future work will be to extend this analysis to other cities where PPSA activities have been ongoing. With over 480 such cities to draw from, any future analysis could prioritise cities that capture the geographical and cultural variations across the country. On the cost side, we had to make certain assumptions to fill data gaps. For example, there is no systematic data for the number of visits needed to change the behaviour of a provider; this parameter was estimated by the CHAI field team as a form of expert opinion. Another assumption we made is that the field team visits providers resistant to engagement efforts the same amount of time as non-resistant providers; in reality, after a couple of visits, field officers know which providers are resistant, and thus, may choose instead to focus their efforts on non-resistant providers. Similarly, we do not consider the fact that once a provider has been visited by field staff a certain number of times, any subsequent visits are likely to be ineffective; for example, data from the field team suggests that the increase in a provider's uptake of CB-NAAT becomes negligible after approximately the 25th visit by a field officer. Finally, while our work concentrates on PPSA activities, there are other important interventions not incorporated in the analysis, for example Nikshay Poshan Yojana (33) an initiative to provide TB patients with financial assistance towards nutritional support. While such measures are likely to have had important benefits to TB patients, they are equally available to those reported from both public and private sectors: thus, they may not substantially change our model projections for future PPSA priorities. Nonetheless, another important area for future work is to incorporate data for these and other initiatives, to achieve more refined estimates for the potential impact of future PPSA activities.

For peer review only - http://bmjopen.bmj.com/site/about/guidelines.xhtml

In summary, as India accelerates efforts for drastically reducing TB burden, engaging effectively with the private sector will play a critical role. Modelling analysis can play a useful role in supporting these efforts, not only in informing strategic planning, but also in addressing operational priorities in different settings. Identifying the most cost-efficient ways of achieving PPSA impact will be critical in benefiting TB patients, in the future of India's TB response.

Contributorship statement

NA conceptualized and supervised the study. All authors contributed to the methodology. SR conducted the formal analysis. NA and SR wrote the original draft. AP, RS and AS contributed to data curation and reviewed the draft.

Competing interests

None declared.

Funding

SR was supported by the Wellcome Trust, grant number 203839/A/16/Z.

Data sharing statement

All data relevant to the study are included in the article or uploaded as supplementary information.

Ethics approval

This study does not involve human participants or animal subjects.

For peer review only - http://bmjopen.bmj.com/site/about/guidelines.xhtml

References

1. Pai M, Behr MA, Dowdy D, Dheda K, Divangahi M, Boehme CC, et al. Tuberculosis. *Nature Reviews. Disease Primers*. 2016;2: 16076. https://doi.org/10.1038/nrdp.2016.76.

2. World Health Organisation. *Global tuberculosis report 2022*. 2022 [Accessed 11th May 2023]. https://www.who.int/publications-detail-redirect/9789240061729 [Accessed 11th May 2023].

3. Satyanarayana S, Nair SA, Chadha SS, Shivashankar R, Sharma G, Yadav S, et al. From Where Are Tuberculosis Patients Accessing Treatment in India? Results from a Cross-Sectional Community Based Survey of 30 Districts. *PLOS ONE*. 2011;6(9): e24160. https://doi.org/10.1371/journal.pone.0024160.

4. Sreeramareddy CT, Qin ZZ, Satyanarayana S, Subbaraman R, Pai M. Delays in diagnosis and treatment of pulmonary tuberculosis in India: a systematic review. *The International Journal of Tuberculosis and Lung Disease: The Official Journal of the International Union Against Tuberculosis and Lung Disease*. 2014;18(3): 255–266. https://doi.org/10.5588/ijtld.13.0585.

5. Arinaminpathy N, Batra D, Khaparde S, Vualnam T, Maheshwari N, Sharma L, et al. The number of privately treated tuberculosis cases in India: an estimation from drug sales data. *The Lancet Infectious Diseases*. 2016;16(11): 1255–1260. https://doi.org/10.1016/S1473-3099(16)30259-6.

6. Kundu D, Chopra K, Khanna A, Babbar N, Padmini TJ. Accelerating TB notification from the private health sector in Delhi, India. *The Indian Journal of Tuberculosis*. 2016;63(1): 8–12. https://doi.org/10.1016/j.ijtb.2016.02.002.

7. Deo S, Jindal P, Gupta D, Khaparde S, Rade K, Sachdeva KS, et al. What would it cost to scale-up private sector engagement efforts for tuberculosis care? Evidence from three pilot programs in India. *PLOS ONE*. 2019;14(6): e0214928.

8. Deo S, Jindal P, Papineni S. Integrating Xpert MTB/RIF for TB diagnosis in the private sector: evidence from large-scale pilots in Patna and Mumbai, India. *BMC Infectious Diseases*. 2021;21(123). https://doi.org/10.1186/s12879-021-05817-1.

9. Sachdeva KS, Kumar A, Dewan P, Kumar A, Satyanarayana S. New vision for Revised National Tuberculosis Control Programme (RNTCP): Universal access - 'reaching the un-reached'. *The Indian Journal of Medical Research*. 2012;135(5): 690–694.

10. Arinaminpathy N, Deo S, Singh S, Khaparde S, Rao R, Vadera B, et al. Modelling the Impact of Effective Private Provider Engagement on Tuberculosis Control in Urban India. *Scientific Reports*. 2019;9(3810).

11. Chadha S, Burugina Nagaraja S, Trivedi A, Satapathy S, N M D, Devi Sagili K. Mandatory TB notification in Mysore city, India: Have we heard the private practitioner's plea? *BMC Health Services Research*. 2017;17: 1. https://doi.org/10.1186/s12913-016-1943-z.

12. Central TB Division. *Guidance for TB notification in India*. 2012.

13. Joint Effort for Elimination of Tuberculosis. *Project JEET*. 2019. https://www.projectjeet.in

14. Joint Effort for Elimination of Tuberculosis. *2019 Annual Report*. 2020. https://www.projectjeet.in/wp-content/uploads/2020/10/JEET-Annual-Report-2019_Low-Res.pdf

For peer review only - http://bmjopen.bmj.com/site/about/guidelines.xhtml

15. Central TB Division. *Leading the way. India TB Report 2023.* 2023.

16. Joint Effort for Elimination of Tuberculosis. *2018 Annual Report*. 2019. https://projectjeet.in/wp-content/uploads/2019/08/JEET-Annual-Report-2018_v4.0_10MAY19.pdf

17. World Health Organisation. *A brief history of tuberculosis control in India.* 2010. https://apps.who.int/iris/bitstream/handle/10665/44408/9789241500159_eng.pdf?sequence=1

18. Central TB Division, Directorate General of Health Services, Ministry of Health with Family Welfare. *National Strategic Plan for Tuberculosis Elimination 2017-2025*. 2017.

19. Subbaraman R, Nathavitharana RR, Satyanarayana S, Pai M, Thomas BE, Chadha VK, et al. The Tuberculosis Cascade of Care in India's Public Sector: A Systematic Review and Meta-analysis. *PLOS Medicine*. 2016;13(10): e1002149. https://doi.org/10.1371/journal.pmed.1002149.

20. Houben RMGJ, Lalli M, Kranzer K, Menzies NA, Schumacher SG, Dowdy DW. What if they don't have tuberculosis? The consequences and trade-offs involved in false-positive diagnoses of tuberculosis. *Clinical Infectious Diseases*. 2019;68(1): 150–156. https://doi.org/10.1093/cid/ciy544.

21. Sachdeva KS, Raizada N, Sreenivas A, Van't Hoog A, van den Hof S, Dewan PK, et al. Use of Xpert MTB/RIF in decentralized public health settings and its effect on pulmonary TB and DR-TB case findings in India. *PLOS ONE*. 2015;10(5): e0126065. https://doi.org/10.1371/journal.pone.0126065.

22. Gopi PG, Prasad VV, Vasantha M, Subramani R, Tholkappian AS, Sargunan D, et al. Annual risk of tuberculosis infection in Chennai city. *Indian Journal of Tuberculosis*. 2008;55(3): 157–161.

23. Dhanaraj B, Papanna MK, Adinarayanan S, Vedachalam C, Sundaram V, Shanmugam S, et al. Prevalence and risk factors for adult pulmonary tuberculosis in a metropolitan city of South India. *PloS One*. 2015;10(4): e0124260. https://doi.org/10.1371/journal.pone.0124260.

24. World Health Organisation. *Global Tuberculosis Report 2020*. 2020. https://www.who.int/publications/i/item/9789240013131

25. Haario H, Saksman E, Tamminen J. An adaptive metropolis algorithm. *Bernoulli*. 2001;7(2): 223–242.

26. Ku CC, MacPherson P, Khundi M, Soko RHN, Feasey HRA, Nliwasa M, et al. Durations of asymptomatic, symptomatic, and care-seeking phases of tuberculosis disease with a Bayesian analysis of prevalence survey and notification data. *BMC Medicine*. 2021;19(1): 298. https://doi.org/10.1186/s12916-021-02128-9.

27. Stallworthy G, Dias HM, Pai M. Quality of tuberculosis care in the private health sector. *Journal of Clinical Tuberculosis and Other Mycobacterial Diseases*. 2020;20: 100171. https://doi.org/10.1016/j.jctube.2020.100171.

28. Pai M, Daftary A, Satyanarayana S. TB control: challenges and opportunities for India. *Transactions of the Royal Society of Tropical Medicine and Hygiene*. 2016;110(3): 158–160.

For peer review only - http://bmjopen.bmj.com/site/about/guidelines.xhtml

29. Das J, Kwan A, Daniels B, Satyanarayana S, Subbaraman R, Bergkvist S, et al. Use of standardised patients to assess quality of tuberculosis care: a pilot, cross-sectional study. *Lancet Infectious Diseases*. 2015;15(11): 1305–1313. https://doi.org/10.1016/S1473-3099(15)00077-8.

30. Van't Hoog A, Langendam MW, Mitchell E, Cobelens FG, Sinclair D, Leeflang MMG, et al. *A sytematic review of the sensitivity and specificity of symptom- and chest-radiography screening for active pulmonary tuberculosis in HIV-negative persons and persons with unknown HIV status*. 2013. https://www.who.int/tb/Review2Accuracyofscreeningtests.pdf

31. Steingart KR, Horne DJ, Pai M, Boehme CC, Dendukuri N. Xpert MTB/RIF assay for pulmonary tuberculosis and rifampicin resistance in adults (Review). *Cochrane Database of Systematic Reviews*. 2014;(1).

32. Pantoja A, Lönnroth K, Lal SS, Chauhan LS, Uplekar M, Padma MR, et al. Economic evaluation of public-private mix for tuberculosis care and control, India. Part II. Cost and cost-effectiveness. *The International Journal of Tuberculosis and Lung Disease: The Official Journal of the International Union Against Tuberculosis and Lung Disease*. 2009;13(6): 705–712.

33. Ministry of Health & Family Welfare. *Nutritional Support to TB patients (Nikshay Poshan Yojana)*. 2018. https://tbcindia.gov.in/WriteReadData/l892s/6851513623Nutrition%20support%20DBT%20Scheme%20details.pdf

For peer review only

Figures

Figure 1. Schematic illustration of the model structure. Panel A illustrates the TB transmission model structure**.** Model compartments are as follows: uninfected with TB (U), latent infection (L), active TB disease (I), presented for care and awaiting diagnosis (Dx), on TB treatment (Tx), temporarily dropped out of the TB care cascade due to missed diagnosis or pre-treatment loss-to-follow-up (E), and recovered with a low risk of relapse ($R^{(L)}$) or with a high risk of relapse ($R^{(H)}$). Dx and Tx are stratified by healthcare sector: private (pr) and public (pu) sector. The private sector is further subdivided into several types of providers as described in the main text. Not shown for simplicity is the acquisition and transmission of DR-TB. See supplementary information for further technical details, including model equations and calibration. Panel B illustrates the non-infected TB-symptomatic model structure. Model compartments are as follows: uninfected with TB but TB symptomatic ($U^{(NTB)}$), presented for care and awaiting diagnosis in the public sector ($Dx^{(NTB\text{-}Pu)}$) or private sector ($Dx^{(NTB\text{-}Pr)}$), and TB treatment after a false-positive diagnosis in the public sector ($Tx^{(NTB\text{-}Pu)}$) or private sector ($Tx^{(NTB\text{-}Pr)}$). The private sector is further subdivided into several types of providers as described in the main text. In order to maintain a constant population size amongst the non-infected TB-symptomatic population, we assume that upon treatment completion, individuals return to the uninfected TB-symptomatic state. The size of this population was determined so that amongst all patients presenting for care, there is a 10% prevalence of TB, in either the public or private sector (19–21).

Figure 2. Model projections for the impact of increasing all PPSA service provisions to their feasible or theoretical maximum has on TB incidence in Ahmedabad and Delhi between 2020 and 2035. Panel A and B show the projected trajectory of TB incidence in Ahmedabad and Delhi, respectively, assuming current PPSA scale up between 2017 and 2020, and its maintenance indefinitely (blue-dashed line), assuming that PPSA service provisions are increased to their feasible or theoretical maximum from 2020 to 2021 and maintained indefinitely (green and purple line, respectively). Shaded areas show 95% credible intervals.

Figure 3. Expansion pathway of PPSA service provisions in Ahmedabad (panel A) and Delhi (panel B). The first point on the graph represents the intervention that had the lowest ICER on its own when scaled up to its feasible maximum coverage; the second point represents the additional PPSA, in combination with the first point, had the lowest ICER; and so on, until all interventions are included. Shaded area shows the 95% credible interval. See tables S18-S19 for further details.

For peer review only

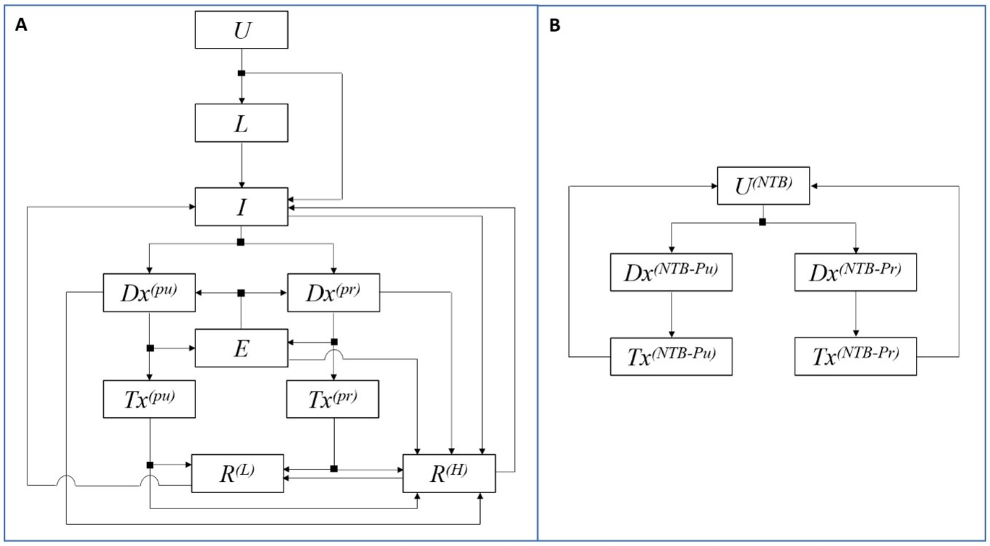

Figure 1. Schematic illustration of the model structure. Panel A illustrates the TB transmission model structure. Model compartments are as follows: uninfected with TB (U), latent infection (L), active TB disease (I), presented for care and awaiting diagnosis (Dx), on TB treatment (Tx), temporarily dropped out of the TB care cascade due to missed diagnosis or pre-treatment loss-to-follow-up (E), and recovered with a low risk of relapse (R(L)) or with a high risk of relapse (R(H)). Dx and Tx are stratified by healthcare sector: private (pr) and public (pu) sector. The private sector is further subdivided into several types of providers as described in the main text. Not shown for simplicity is the acquisition and transmission of DR-TB. See supplementary information for further technical details, including model equations and calibration. Panel B illustrates the non-infected TB-symptomatic model structure. Model compartments are as follows: uninfected with TB but TB symptomatic (U(NTB)), presented for care and awaiting diagnosis in the public sector (Dx(NTB-Pu)) or private sector (Dx(NTB-Pr)), and TB treatment after a false-positive diagnosis in the public sector (Tx(NTB-Pu)) or private sector (Tx(NTB-Pr)). The private sector is further subdivided into several types of providers as described in the main text. In order to maintain a constant population size amongst the non-infected TB-symptomatic population, we assume that upon treatment completion, individuals return to the uninfected TB-symptomatic state. The size of this population was determined so that amongst all patients presenting for care, there is a 10% prevalence of TB, in either the public or private sector (12–14).

159x90mm (220 x 220 DPI)

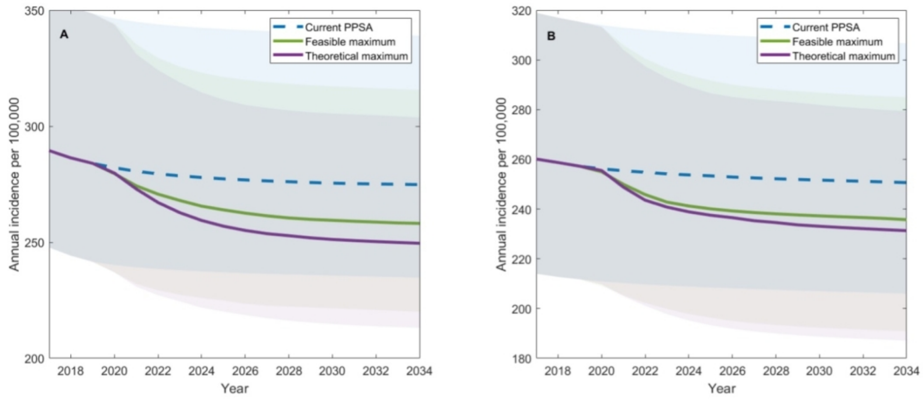

Figure 2. Model projections for the impact of increasing all PPSA service provisions to their feasible or theoretical maximum has on TB incidence in Ahmedabad and Delhi between 2020 and 2035. Panel A and B show the projected trajectory of TB incidence in Ahmedabad and Delhi, respectively, assuming current PPSA scale up between 2017 and 2020, and its maintenance indefinitely (blue-dashed line), assuming that PPSA service provisions are increased to their feasible or theoretical maximum from 2020 to 2021 and maintained indefinitely (green and purple line, respectively). Shaded areas show 95% credible intervals.

189x81mm (220 x 220 DPI)

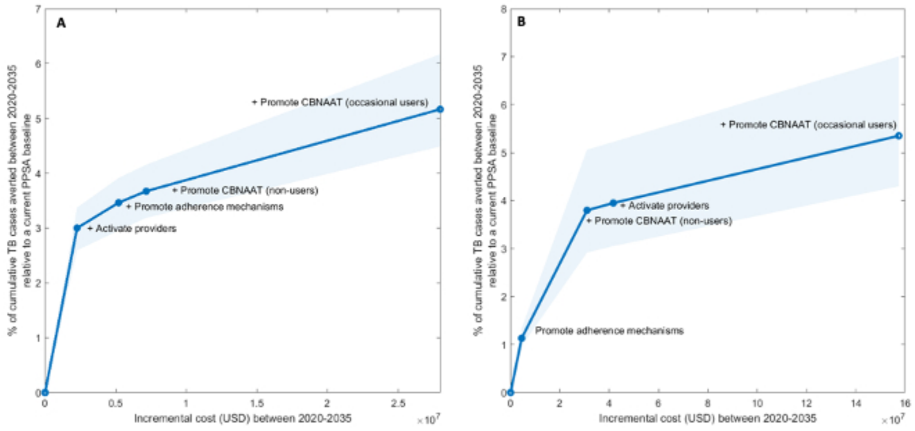

Caption : Figure 3. Expansion pathway of PPSA service provisions in Ahmedabad (panel A) and Delhi (panel B). The first point on the graph represents the intervention that had the lowest ICER on its own when scaled up to its feasible maximum coverage; the second point represents the additional PPSA, in combination with the first point, had the lowest ICER; and so on, until all interventions are included. Shaded area shows the 95% credible interval. See tables S18-19 for further details.

338x190mm (54 x 54 DPI)

Appendix 3

The value and cost efficiency of private sector engagement

1. Materials and methods

Below, we describe the model, including its structure, governing equations, and table of parameters. The model was implemented in MATLAB.

1.1. Model structure

We modelled TB transmission amongst adults (>15 years old) in a typical urban setting in India. We modelled the fragmented healthcare system, by differentiating between the public and private sector (1–7). We further divided the private sector by engagement status, CB-NAAT use and FDC use (see main text for more details on these categories). We calibrated the model to TB prevalence, annual risk of infection, and mortality (table 1). We also modelled the acquisition and transmission of DR-TB, but ignored HIV status as well as distinctions between pulmonary and extrapulmonary TB. The overall model structure is illustrated schematically in fig 1.

For a given set of model parameters, we initially simulated the model to equilibrium after introducing TB into a disease-free population. From 1997 to 2007 and its maintenance until the present day, we incorporated the scale-up of RNTCP (India's national TB programme) and its impact on the TB epidemic (8). From 1997, we also incorporated population growth. From 2017 to 2020, we simulated the scale-up of PPSA services to current 2019 levels. We then simulated the model forward from 2020 to 2035.

1.2. Governing equations for the mathematical transmission model

The equations correspond to the model described in fig 1. State variables (capital letters) are as listed in table S1, while model parameters (lower-case and Greek letters) are as listed in tables S2-S5.

For peer review only

Model stage	Description
U	Uninfected
L	Latent infection
I_q	Active TB
$Dx_q^{(pu)}$	Sought care, awaiting TB diagnosis, public sector
$Dx_q^{(pr)}$	Sought care, awaiting TB diagnosis, private sector. This is split further depending on engagement status and CB-NAAT use (table S6)
$Tx_q^{(FL:pu)}$	Undergoing first line TB treatment, public sector
$Tx_q^{(FL:pr)}$	Undergoing first line TB treatment, private sector. This is split further depending on FDC use and adherence support offered (table S6).
$Tx^{(SL)}$	Undergoing second line TB treatment.
E_q	Between seeking care, after misdiagnosis or loss-to-follow-up
$R^{(L)}$	Recovered after treatment completion, low risk of relapse
$R^{(H)}$	Recovered after self-cure or incomplete treatment, high risk of relapse
**Subscript**	**Description**
q	Drug resistance status (0 = DS-TB, 1 = DR-TB)

Table S1. Model stages and subscript description

Parameter	Symbol	Value		Source
**TB natural history**				
Mean rate of transmission per TB case	β	10.42 yr^{-1} [7.28-17.24]		Model estimate
Relative rate of transmission between DS- and DR-TB	d	0.61 yr^{-1} [0.49-0.79]		Model estimate
Rate of MDR acquisition	m_a	Public sector/active providers that offer either adherence support of government supplied FDCs	0.01 [0.008-0.012]	(9)
	m_b	All private providers that are unengaged, inactive, or active that do not offer government supplied FDCs or adherence support	0.05 [0.04-0.06]	
Proportion of TB infections undergoing rapid progression	θ	0.11 [0.09-0.15]		(10,11)
Per capita hazard rate of progression to active disease from latency	ρ	0.001 yr^{-1} [0.0003-0.0024]		(11,12)
Per-capita hazard rate of relapse	r_1	Following self-cure/treatment default	0.14 yr^{-1} [0.10-0.18]	(9,13)

For peer review only

	r_2	Following treatment cure, >2 years	0.002 yr^{-1} [0.001-0.003]	
Stabilisation of relapse risk following treatment	ς	0.5 yr^{-1} [0.4-0.6]		(13): most relapse occurs in the first two years after treatment
Per-capita hazard rate of spontaneous cure	φ	0.17 yr^{-1} [0.13-0.21]		(14)
Per-capita hazard rate of TB mortality	μ_1	0.18 [0.13-0.25]		Model estimate
Proportion reduction in susceptibility to reinfection due to previous infection	π	0.21 [0.15-0.25]		(15)
**Demographics**				
Per-capita hazard rate of background mortality	μ_2	0.015 yr^{-1}		World Bank estimates: corresponds to an average lifespan of 69 years
Per-capita rate of population growth	σ	0.02 [0.01-0.025]		(16)

Table S2. List of model parameters: TB natural history

Parameter	Symbol	Value		Source
**TB services: diagnosis**				
Proportion of TB cases seeking care in the public sector	η	0.50 [0.40-0.60]		(2,17–19)
Proportion of private providers that are engaged	e	Ahmedabad	0.50	Data provided by CHAI
		Delhi	0.73	
Heterogeneity index of the behaviour of engaged private providers	$\propto$	Ahmedabad	0.39 [0.32-0.46]	Data provided by CHAI
		Delhi	0.32 [0.25-0.41]	
Proportion of engaged private providers that are active	a	Ahmedabad	0.80	Data provided by CHAI
		Delhi	0.44	
Per-capita hazard rate of initial careseeking for TB symptoms	δ_1	1.38 yr^{-1} [0.88-2.38]		Model estimate
Per-capita hazard rate of repeat careseeking for TB symptoms (following missed diagnosis)	δ_2	12 yr^{-1} [6-26]		Assumption: corresponds to range of 2 weeks to 2 months

Proportion of TB cases diagnosed correctly, per careseeking attempt in routine public sector TB services	ε_{sn}	0.84 [0.80-0.85]		(20,21)
Specificity of diagnosis in routine public sector TB services	ε_{sp}	0.98 [0.97-0.99]		(20)
CB-NAAT sensitivity	x_{sn}	0.89 [0.85-0.92]		(22)
CB-NAAT specificity	x_{sp}	0.99 [0.98-1.00]		(22)
Proportion of active private providers that send samples for confirmatory CB-NAAT testing 100% of the time	p_{Xpert1}	Ahmedabad	0.09	Data provided by CHAI
		Delhi	0.04	
Proportion of active private providers that never send samples for confirmatory CB-NAAT testing	p_{Xpert0}	Ahmedabad	0.55	Data provided by CHAI
		Delhi	0.66	
Proportion of samples that are sent off for confirmatory CB-NAAT testing amongst active private providers that occasionally send samples off for confirmatory CB-NAAT testing	x_p	Ahmedabad	0.23	Data provided by CHAI
		Delhi	0.15	
Clinical diagnosis sensitivity (including chest X-ray)	c_{sn}	0.75 [0.65-0.80]		(21)
Clinical diagnosis specificity (including chest x-ray)	c_{sp}	0.85 [0.80-0.95]		(21)
Proportion of DR-TB cases receiving drug susceptibility testing at point of TB diagnosis	$Þ^{(pu)}$	Public sector	0.45 [0.40-0.50]	(23)
	$Þ^{(pr0)}$	Private sector (providers not using CB-NAAT)	0	
	$Þ^{(pr1)}$	Private sector (providers using CB-NAAT)	1.00	

Table S3. List of model parameters: TB diagnosis

For peer review only

Parameter	Symbol	Value		Source
**TB services: treatment**				
Proportion of diagnosed TB cases initiating TB treatment	ω	0.87 [0.85-0.90]		(20). Assumed that patients from active providers are referred to the public sector to initiate second line treatment.
Amongst DR-TB cases failing first-line treatment, the proportion successfully transferred to second-line treatment, public sector	p_{SL}	0.87 [0.85-0.92]		Assumption
TB treatment initiation delay	ϕ	52 yr^{-1}		Assumed: corresponds to a mean treatment delay of 1 week in routine TB care
Proportion of patients that agree to adherence support	p_{adh1}	0.92		Data provided by CHAI
Proportion of active providers that agree to adherence support	p_{adh2}	Ahmedabad	0.93	Data provided by CHAI
		Delhi	0.93	
Proportion of active providers that agree to government supplied FDCs	p_{fdc}	Ahmedabad	0.39	Data provided by CHAI
		Delhi	0.07	
Per-capita hazard rate of treatment completion	τ	First line	2 yr^{-1}	Corresponds to a duration of 6 months
	τ_{mdr}	Second line	0.5 yr^{-1}	Corresponds to a duration of 2 years
Proportion of TB patients that do not complete treatment	χ_{Pu}	First line, public sector	0.15 [0.13-0.17]	(23,24), data provided by CHAI
	χ_{Pr_Z}	First line, private sector: unengaged/inactive/active but no adherence support of government supplied FDCs offered	0.48 [0.45-0.55]	
	χ_{Pr_F}	First line, private sector: active (government	0.17 [0.16 -0.19]	

		supplied FDCs offered, no adherence support offered)		
	$\chi_{\mathrm{Pr\_A}}$	First line, private sector: active (no government supplied FDCs offered, adherence support offered)	0.21 [0.20-0.25]	
	$\chi_{\mathrm{Pr\_AF}}$	First line, private sector: active (government supplied FDCs and adherence support offered)	0.15 [0.13-0.17]	
	χ_{mdr}	Second line	0.50 [0.45-0.60]	

Table S4. List of model parameters: TB treatment

Parameter	Symbol	Value	Source
		**Costs**	
Cost per active TB disease diagnosis (passive case finding)	C_D	USD 18.30	(25)
Cost of first line treatment for active TB disease per person	C_T	USD 23.70 per treatment-month	(25)

Table S5. List of model parameters: TB costs

Label	Description
	**Private sector diagnosis classification**
pr1	Unengaged private providers
pr2	Engaged, inactive private providers
pr3	Engaged, active private providers that send samples for confirmatory CB-NAAT testing 100% of the time
pr4	Engaged, active private providers that send samples for confirmatory CB-NAAT testing occasionally, CB-NAAT
pr5	Engaged, active private providers that send samples for confirmatory CB-NAAT testing occasionally, no CB-NAAT
pr6	Engaged, active private providers that never send samples for confirmatory CB-NAAT testing
	**Private sector first-line treatment classification**
prZ1	Treatment offered by inactive and unengaged private providers
prAF	Engaged, active private providers that offer adherence support and government supplied FDCs
prA	Engaged, active private providers that only offer adherence support
prF	Engaged, active private providers that only offer government supplied FDCs
prZ2	Engaged, active private providers that offer neither adherence support or government supplied FDCs

Table S6. Private sector classification

For peer review only

Label	Classes
S_1	pu, prZ1, prZ2, prAF, prA, prF
S_2	pr1, pr2, pr5, pr6
S_3	pr3, pr4

Table S7. Notation

Uninfected stage

$$\frac{dU}{dt} = T - (\lambda_q + \mu_2)U \quad (1)$$

where term T represents births. To maintain a constant population for simplicity, we assumed that T is equivalent to the number of deaths occurring in the model.

Latent TB infection

$$\frac{dL_q}{dt} = (1-\theta)\lambda_q U + \pi\lambda_q(1-\theta) - (\rho + \mu_2 + \lambda_q)L_q \quad (2)$$

Active disease, pre-careseeking

$$\frac{dI_q}{dt} = \theta\lambda_q U + \pi\lambda_q\theta + \rho L_q + r_1 R^{(H)} + r_2 R^{(L)} - (\varphi + \delta_1 + \mu_1 + \mu_2)I_q \quad (3)$$

where the subscript q denotes drug resistance status:

$$q = \begin{cases} 0 & DS\,TB \\ 1 & DR\,TB \end{cases}$$

We assumed that, when presenting for care, a proportion η of patients visit the public sector, the remainder $(1-\eta)$ visiting the private sector. We divided the private sector further depending on whether private providers are engaged by JEET or not, and whether engaged providers are active or inactive, in notifying TB. Finally, active providers were categorised by their use of CB-NAAT (table S6-S7). The proportion of patients that visit each different type of provider is summarised in table S8.

For peer review only

Provider type	Notation	Formula
Public	$v^{(pu)}$	$v^{(pu)} = \eta$
Unengaged, private	$v^{(pr1)}$	$v^{(pr1)} = (1-\eta)(1-h^{\propto})^{*}$
Engaged, inactive, private	$v^{(pr2)}$	$v^{(pr2)} = (1-\eta)h^{\propto}(1-a)$
Engaged, active, private (always use CB-NAAT)	$v^{(pr3)}$	$v^{(pr3)} = (1-\eta)h^{\propto}ap_{CBNAAT1}$
Engaged, active, private (occasionally use CB-NAAT, patient receives an CB-NAAT test)	$v^{(pr4)}$	$v^{(pr4)} = (1-\eta)h^{\propto}a(1-p_{CBNAAT1}-p_{CBNAAT0})x_p$
Engaged, active, private (occasionally use CB-NAAT, patient does not receive an CB-NAAT test)	$v^{(pr5)}$	$v^{(pr5)} = (1-\eta)h^{\propto}a(1-p_{CBNAAT1}-p_{CBNAAT0})(1-x_p)$
Engaged, active, private (non CB-NAAT user)	$v^{(pr6)}$	$v^{(pr6)} = (1-\eta)h^{\propto}ap_{CBNAT0}$

Table S8. Proportion of patients that visit different providers. Private providers under the classifications pr1, pr2, pr5 and pr6 rely on clinical diagnosis, whereas private providers under the classification pr3 and pr4 employ CB-NAAT testing. *h is the proportion of private providers that have been engaged by the PPSA, and α is a 'heterogeneity index' governing the distribution of patient load across all providers (see fig S1).

The following equations describe the different pathways for patients that seek care with the different providers described above:

Seeking care, awaiting TB diagnosis

$$\frac{dDx_q^{(pu)}}{dt} = v^{(pu)}\delta_1 I_q + v^{(pu)}\delta_2 E_q$$

$$-\left[\varepsilon_1\omega\phi\left(1-s_q^{(pu)}\right)+\left(\varepsilon_1\omega\phi s_q^{(pu)}\right)_q+(1-\omega\varepsilon_1)\phi s_q^{(pu)}+\varphi+\mu_1+\mu_2\right]Dx_q^{(pu)} \quad (4)$$

$$\frac{dDx_q^{(s)}}{dt} = v^{(s)}\delta_1 I_q + v^{(s)}\delta_2 E_q$$

$$-\left[\varepsilon_3\omega\phi(1-s_q^{(pr0)})+(\varepsilon_3\omega\phi s_q^{(pr0)})_q+(1-\omega\varepsilon_3)\phi s_q^{(pr0)}+\varphi+\mu_1+\mu_2\right]Dx_q^{(s)} \quad (5)$$

$$\frac{dDx_q^{(h)}}{dt} = v^{(h)}\delta_1 I_q + v^{(h)}\delta_2 E_q$$

$$-\left[\varepsilon_2\omega\phi(1-\mathrm{s}_q^{(pr1)}) + (\varepsilon_2\omega\phi s_q^{(pr1)})_q + (1-\omega\varepsilon_2)\phi s_q^{(pr1)} + \varphi + \mu_1 + \mu_2\right] Dx_q^{(h)} \qquad (6)$$

Where superscript (s) represents either pr1, pr2, pr5 or pr6 and superscript (h) represents either pr3 or pr4 as defined in table S8.

Patients that are misdiagnosed (as false-negative) enter the compartment E, to re-initiate careseeking after a delay:

Temporarily dropped out of careseeking

$$\frac{dE_q}{dt} = (1-\omega\varepsilon_1)\phi Dx_q^{(pu)} + \sum_{i\in S_3}[(1-\omega\varepsilon_2)\phi]Dx_q^{(i)} - (\varphi + \delta_2 + \mu_1 + \mu_2)E_q$$

when q=0 (7)

$$\frac{dE_q}{dt} = (1-\omega\varepsilon_1)\phi s_{pu} Dx_q^{(pu)} + \sum_{i\in S_2}[(1-\omega\varepsilon_3)\phi s_{pr0}]Dx_q^{(i)} + \sum_{i\in S_3}[(1-\omega\varepsilon_2)\phi s_{pr1}]Dx_q^{(i)}$$

$$+\sum_{i\in S_1}\left[\tau(1-p_{SL}) + \frac{\tau\chi_i}{1-\chi_i}\right]Tx_q^{(i)} - (\varphi + \delta_2 + \mu_1 + \mu_2)E_q$$

when q=1 (8)

where the summand represents patients not being cured by first-line treatment, but not being transferred to second-line treatment. Subscript S_1, S_2, and S_3 are defined in table S7.

Treatment

We categorised active private providers by the treatment services offered: adherence support and government supplied FDCs (prAF); adherence support only (prA); government supplied FDCs only (prF); or neither (prZ2) (table S6). The proportion of patients at active providers that receive the different treatment services offered is defined in table S9.

For peer review only

Provider type	Notation	Formula
Adherence support and government supplied FDCs	$v^{(prAF)}$	$v^{(prAF)} = p_{adh1}p_{adh2}p_{fdc}$
Adherence support only	$v^{(prA)}$	$v^{(prA)} = p_{adh1}p_{adh2}(1-p_{fdc})$
Government supplied FDCs only	$v^{(prF)}$	$v^{(prF)} = (1-p_{adh1}p_{adh2})p_{fdc}$
Neither adherence support or government supplied FDCs	$v^{(prZ)}$	$v^{(prZ)} = (1-p_{adh1}p_{adh2})(1-p_{fdc})$

Table S9. Proportion of patients at active providers that receive treatment services.

For peer review only

Governing equations for the different Tx compartments are:

$$\frac{dTx_q^{(FL:y)}}{dt} = \varepsilon_i \phi \omega v^{(pr\_y)} Dx_q^{(y)} - (m_y + \tau + \frac{\tau \chi_y}{1-\chi_y} + \mu_2) Tx_q^{(FL:y)} \quad \text{when q = 0} \quad (9)$$

$$\frac{dTx_q^{(FL:y)}}{dt} = \varepsilon_i \phi \omega v^{(pr\_y)} (1-s_y) Dx_q^{(y)} + m_y Tx_{q=0}^{(FL:y)} - (\tau p_{SL} + [\tau(1-p_{SL}) + \frac{\tau \chi_y}{1-\chi_y}] + \mu_2) Tx_q^{(FL:y)} \quad \text{when q = 1} \quad (10)$$

$dTx_q^{(FL:y)}$ represents the different Tx compartments (pu, prZ1, prAF, prA, prF and prz2), defined in table S6. The parameter ε_i is the proportion of patients tested that are correctly diagnosed (in other words, the sensitivity of the test) and is dependent on where the patient is diagnosed: ε_1 when Dx^{pu}; ε_3 when Dx^{pr1}, Dx^{pr2}, Dx^{pr5}, Dx^{pr6}; and ε_2 when Dx^{pr3}, Dx^{pr4}. The parameter ϕ is the treatment initiation delay (assumed conservatively to be 1 week across all providers) and ω is the proportion of diagnosed TB cases initiating TB treatment (assumed to be 0.87 across all providers) (20). $v^{(pr\_y)}$, described above, applies only to TB patients that are diagnosed by active private providers. s_y is the proportion of MDR-TB cases receiving drug susceptibility testing at the point of TB diagnosis and is dependent on the amount of CB-NAAT testing. We assume the proportion is 0.45, 1.00 and 0 in the public sector ($s^{(pu)}$), respectively amongst private providers that always use CB-NAAT, and amongst private providers that do not use CB-NAAT, respectively (23). Thus, $1 - s_y$ represents TB patients with DR-TB that are not tested for drug resistance and are therefore mistakenly initiated onto first-line treatment. The parameter m_y is the rate of MDR acquisition while on first-line treatment and is assumed to be higher amongst all private providers (m_b) except active providers that offer government supplied FDCs and adherence support, than public sector providers (m_a) (9). The parameter τ is the per-capita rate of treatment completion for first line treatment and

its value corresponds to a duration of 6 months.

The parameter p_{SL} applies only to TB patients with DR-TB and is defined as the proportion of DR-TB cases that are successfully transferred to second-line treatment following failure of first-line treatment. The parameter χ_y is the proportion of TB cases that do not complete, or are non-adherent to, first line treatment; it is dependent on the quality of treatment services offered by providers and is informed by programmatic data (tables 2-5).

The following equation represents the number of DR-TB patients receiving second-line treatment:

$$\frac{dTx_q^{(SL)}}{dt} = \varepsilon_1\omega\phi s_{pu}Dx_q^{(pu)} + \varepsilon_3\omega\phi s_{pr0}\sum_{i\in S_2} Dx_q^{(i)} + \varepsilon_2\omega\phi s_{pr1}\sum_{i\in S_3} Dx_q^{(i)}$$

$$+\tau p_{SL}\sum_{i\in S_1} Tx_q^{(i)} - (\tau_{mdr} + \frac{\tau_{mdr}\chi_{\mathrm{mdr}}}{1-\chi_{\mathrm{mdr}}} + \mu_2)Tx_q^{(SL)} \quad (11)$$

Where S_1, S_2 and S_3 are sets of indices defined in table S7.

TB patients that successfully complete TB treatment, recover with a low risk of relapse. Governing equations for those with low risk of relapse are:

Recovery, low risk of relapse

$$\frac{dR^{(L)}}{dt} = \tau\sum_{i\in S_1} Tx_q^{(i)} + \varsigma R^{(H)} - (r_2 + \mu_2 + \lambda_q)R^{(L)} \quad (12)$$

Where S_1 is the set of indices defined in table S7.

On the other hand, those recovered from TB with a high risk of relapse (either from previous inadequate treatment or self-cure) have governing equations:

For peer review only

Recovery, high risk of relapse

$$\frac{dR^{(H)}}{dt} = \frac{\tau\chi_{pu}}{1-\chi_{pu}} Tx_q^{(FL:pu)} + \sum_{i\in S_1} (\frac{\tau\chi_{(i)}}{1-\chi_{(i)}}) Tx_q^{(i)} + \varphi \sum_{i\in[S_2,S_3]} [Dx_q^{(pu)} + Dx_q^{(i)} + E_q + I_q]$$

$$-(\varsigma + r_1 + \mu_2 + \lambda_q)R^{(H)} \quad (13)$$

Where S_1, S_2 and S_3 are sets of indices defined in table S7.

The force of infection can be written as:

$$\lambda_q = \frac{\beta\{\sum_{i\in[S_2,S_3]}[I_q + Dx_q^{pu} + Dx_q^{(i)} + E_q]\} + d_q\beta\{\sum_{i\in[S_2,S_3]}[I_q + Dx_q^{pu} + Dx_q^{(i)} + E_q]\}}{N} \quad (14)$$

Where S_2 and S_3 are sets of indices defined in table S7.

Non-TB symptomatic population

To count the number of false positive diagnoses that arise, we modelled a non-TB symptomatic population (fig 1). We defined non-TB symptomatics as patients who do not have TB, but who have symptoms that would make them eligible for testing for TB. We assumed this population to be independent of the TB population described above.

Non-TB symptomatics seeking care

$$\frac{dU^{(NTB)}}{dt} = y_i\phi\omega Dx^{(NTB)} + \tau Tx^{(NTB)} - \delta_1 v^{(x)} U^{(NTB)} \quad (15)$$

where $v^{(x)}$ is drawn from equations 4 to 10, depending on the type of provider a patient seeks care from, and where y_i represents either the specificity of TB diagnosis in the public sector (y_1), specificity of CB-NAAT (y_2) or the specificity of clinical diagnosis (y_3).

Awaiting diagnosis

$$\frac{dDx^{(NTB)}}{dt} = \delta_1 v^{(x)} U^{(NTB)} - \phi\omega Dx^{(NTB)} \quad (16)$$

For peer review only

Undergoing (unnecessary) first-line TB treatment

$$\frac{dTx^{(NTB)}}{dt} = (1 - y_i)\phi\omega Dx^{(NTB)} - \tau Tx^{(NTB)} \quad (17)$$

We assumed the total population size of the non-TB symptomatic population remains constant; we determined the size of this population so that for every true positive TB diagnosis, approximately nine uninfected individuals who present for diagnosis with symptoms of TB are also tested for TB (20,26–30).

1.3. Model calibration

We performed calibrations using Adaptive Bayesian MCMC (table 1 -5)(31), allowing propagation of uncertainty from model inputs to projections in a systematic way.

We define the posterior distribution as:

$$p(\theta|D) \propto L(D|\theta) . \pi(\theta) \quad (18)$$

where D is data; θ is a vector of model inputs subject to uncertainty; L is the likelihood of the data given θ, and π is the prior distribution. We assumed uniform distributions for the prior distributions. We defined the likelihood by fitting log-normal distributions to the calibration targets (see table 1).

Once the MCMC converged, we removed the burn-in period (10,000 iterations) and selected every 50th sample to reduce any autocorrelation. This provided 1200 samples from the posterior distribution.

For peer review only

2. Supporting figures

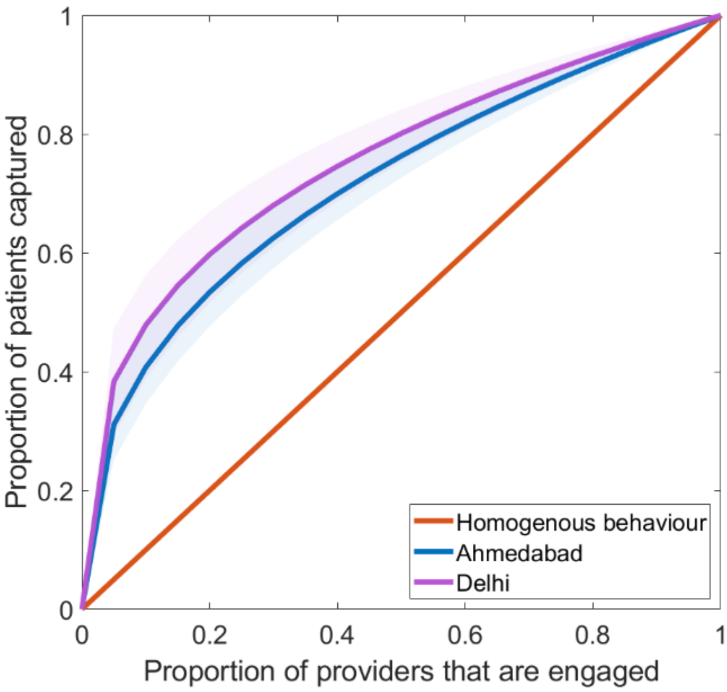

Figure S1. Heterogeneity in the patient share of private providers across Ahmedabad and Delhi. Homogenous patient share (red line) corresponds to a scenario where all private providers have the same numbers of patients. Under this assumption, increasing the proportion of engaged providers will linearly increase the proportion of patients benefiting from a PPSA. In reality, there are a few large private providers that manage most TB patients, and many smaller providers that manage few patients. The larger the area under the curve, the greater the heterogeneity. If the providers that capture the most patients are already engaged by JEET, increasing the proportion of engaged providers further will have little incremental benefit. Therefore, assuming homogenous patient share can overestimate the impact on TB epidemiology. As described in the main text, the 'heterogeneity index' controls the curvature of these plots. Table S11 shows the heterogeneity index calculated for each city.

review only

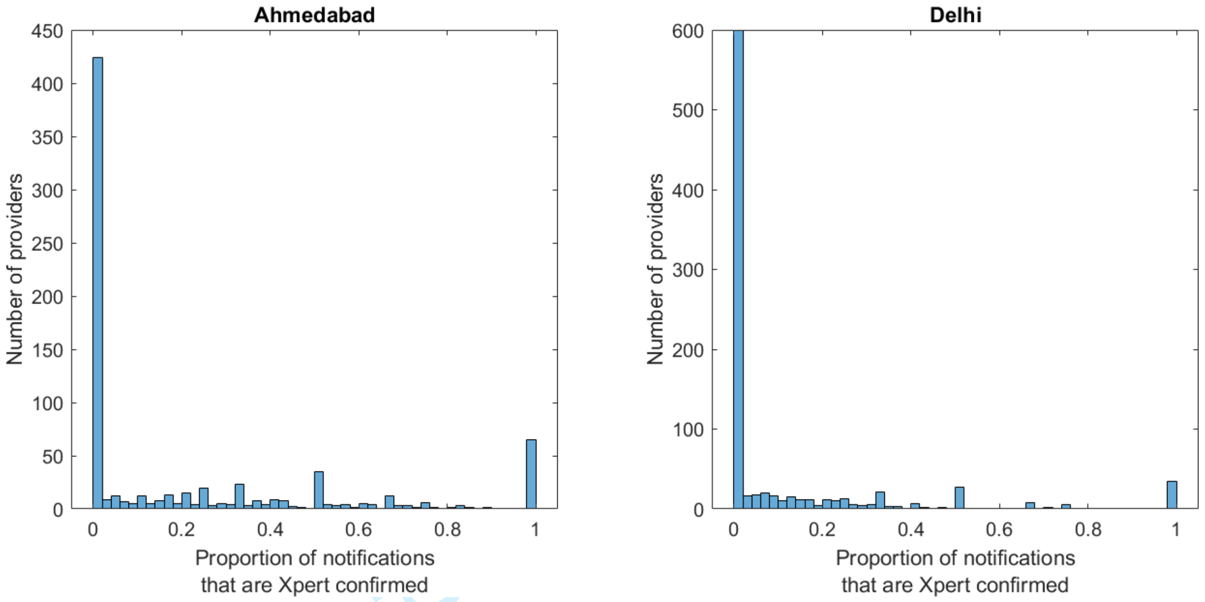

Figure S2. CB-NAAT use amongst active private providers in Ahmedabad and Delhi in 2019. From this data, we classified active providers into three categories: those that always use CB-NAAT, those that use CB-NAAT occasionally, and those that never use CB-NAAT. To be classified as occasionally using CB-NAAT, we assume that providers need to use CB-NAAT for at least 1% of notifications and less than 100% of notifications.

For peer review only - http://bmjopen.bmj.com/site/about/guidelines.xhtml

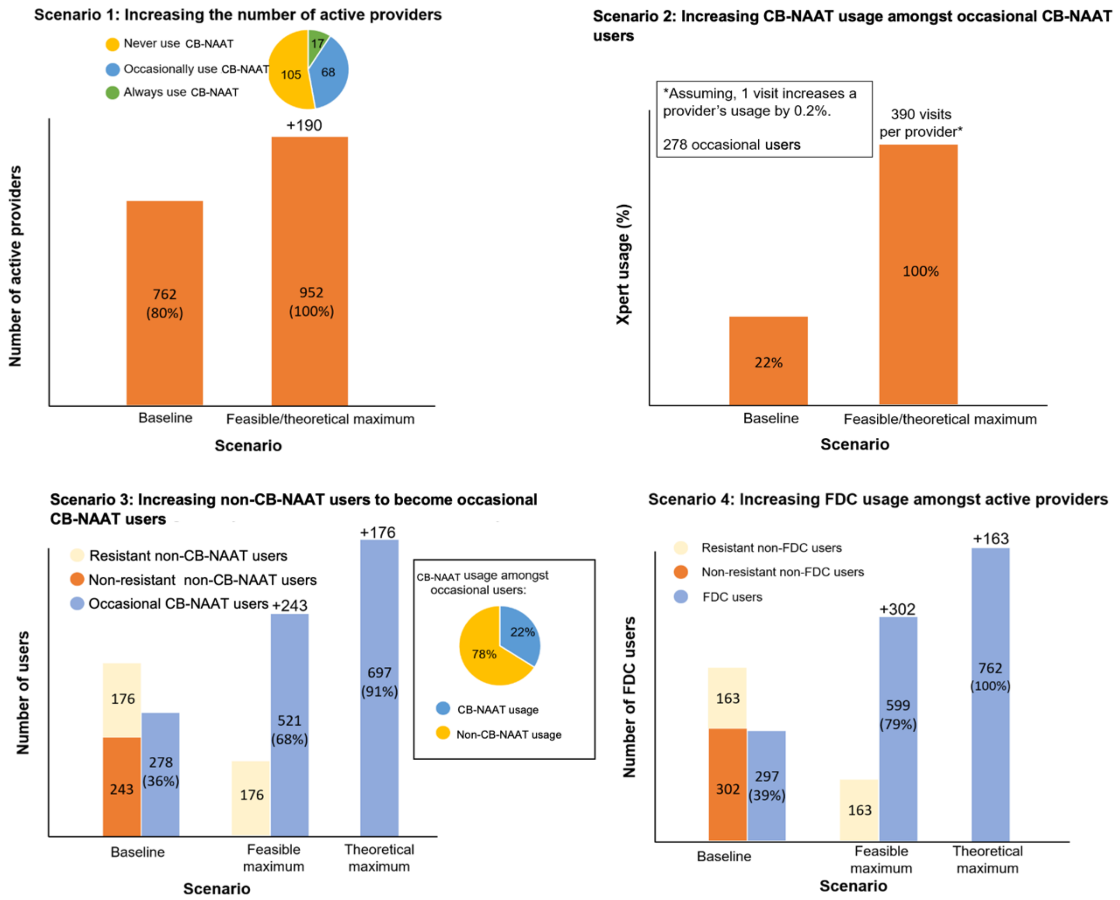

Figure S3. Feasible and theoretical maxima for each type of service provision, Ahmedabad. *Scenario 1:* We assume all inactive providers (e.g., 190) are converted into active providers. We assume no inactive providers are resistant, thus the feasible and theoretical maximum scenarios are the same. Newly active providers are assumed to have the same behaviour as pre-existing active providers; in the case of Ahmedabad, 9% use CB-NAAT 100% of the time, 36% use CB-NAAT occasionally (with an average CB-NAAT usage of 22%), 55% never use CB-NAAT, 98% offer adherence support and 39% consistently use governmental FDCs. For example, of the 190 newly active providers, 17 providers use CB-NAAT 100% of the time, 68 use CB-NAAT occasionally, and 105 never use CB-NAAT. *Scenario 2:* At baseline, occasional CB-NAAT users use CB-NAAT 22% of the time; this is increased to 100% under the feasible and theoretical maximum scenario. We assume no occasional users are resistant to increasing their CB-NAAT usage. It is estimated that one visit by a field officer increases a provider's usage of CB-NAAT by 0.2%. At baseline, Ahmedabad has 278 occasional CB-NAAT users. *Scenario 3:* At baseline, there are 419 non-CB-NAAT users and 278 occasional users. Of the 419 non-CB-NAAT users, it is estimated that 42% are resistant to using CB-NAAT; thus, only 243 non-CB-NAAT users can be converted to become an occasional user. At the theoretical maximum, the percentage of active providers that use CB-NAAT occasionally is 91%; the remaining 9% are providers that always use CB-NAAT. *Scenario 4:* At baseline, there are 465 non-FDC users and 297 consistent FDC users. Of the 465, 35% are resistant to FDCs.

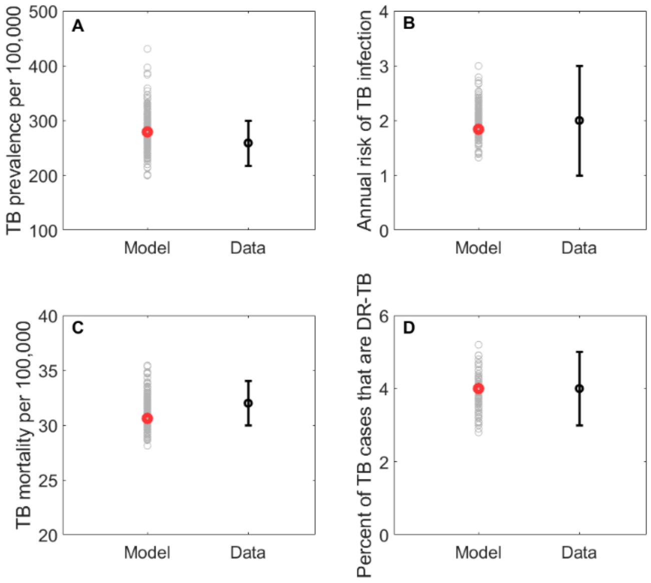

Figure S4. Model fits to data. Data points are described in table 1. Panel A – TB prevalence per 100,000 in a typical urban Indian setting; B – Annual risk of TB infection in a typical urban Indian setting; C – TB deaths per 100,000 in 2019, India; D – Percent of TB cases that are drug-resistant TB in 2019

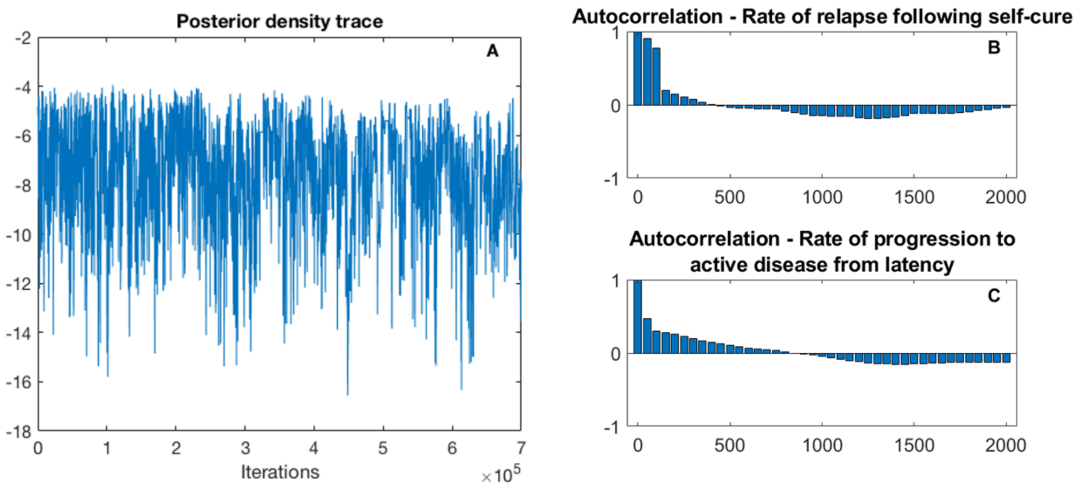

Figure S5. MCMC diagnostics. Panel A shows the posterior density trace, excluding the 'burn-in' period. Panel B and C show autocorrelation plots for two selected parameters (rate of relapse following self-cure and the rate of progression to active disease from latency).

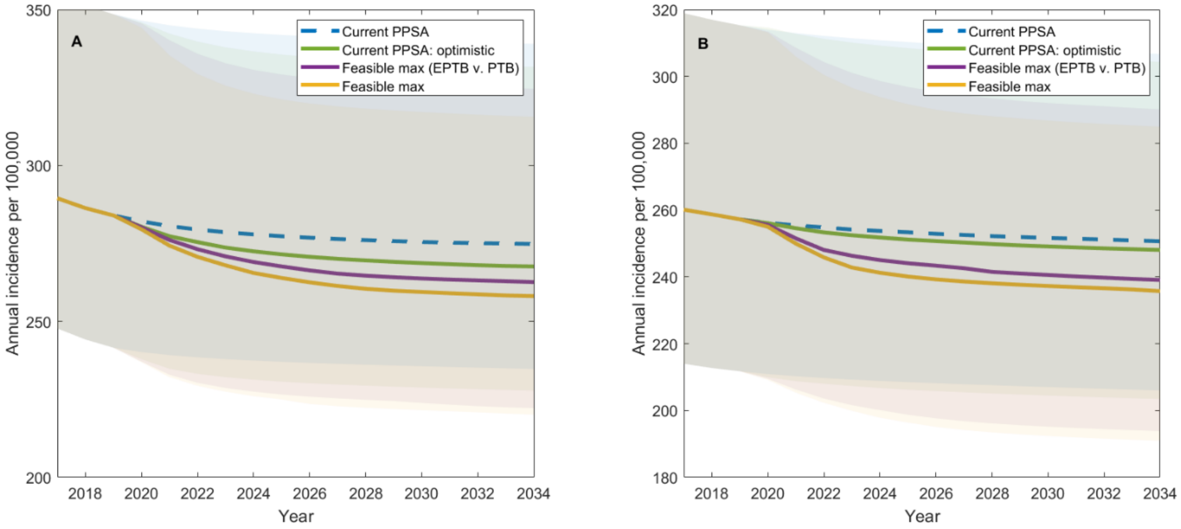

Figure S6. Sensitivity analysis on model projections for the impact of increasing all service provisions to their feasible maximum has on TB incidence in Ahmedabad and Delhi between 2020 and 2035. Panel A shows the projected trajectory of TB incidence for Ahmedabad and Panel B for Delhi, assuming current PPSA scale up between 2017 and 2020 (blue dashed line) and its maintenance indefinitely; assuming current PPSA scale up between 2017 and 2020 whilst optimistically assuming that active providers are continuously active (green line); service provisions are increased to their feasible maximum from 2020 to 2021 and maintained indefinitely, but assuming that 50% of TB cases that present to care are extra-pulmonary TB (purple line); that service provisions are increased to their feasible maximum from 2020 to 2021 and maintained indefinitely, as presented in the main text (yellow line).

peer review only

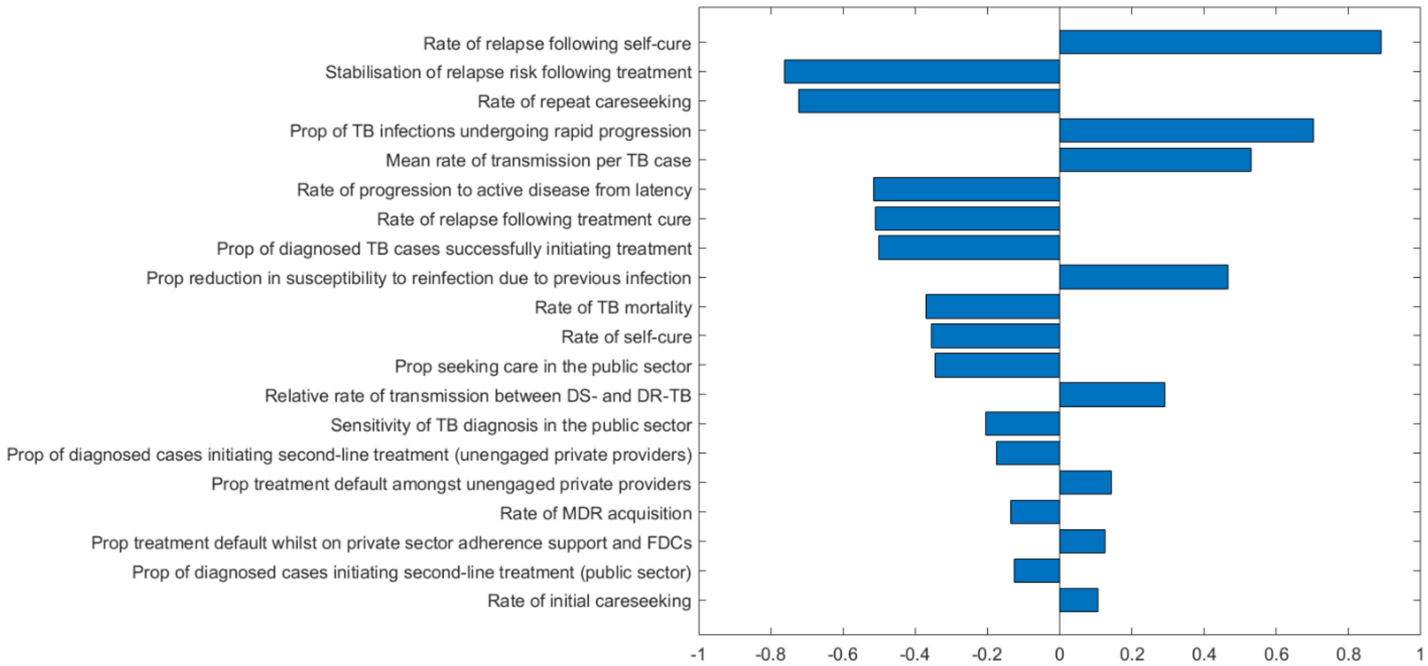

Figure S7. Multivariate sensitivity analysis of impact for Ahmedabad. We used partial rank correlation coefficient (PRCC) to examine which parameter listed in tables 2-5 the output cases averted is most sensitive towards. Larger bars represent more sensitive parameters. Shown are the 20 most influential model parameters, in decreasing order of sensitivity from top to bottom.

For peer review only

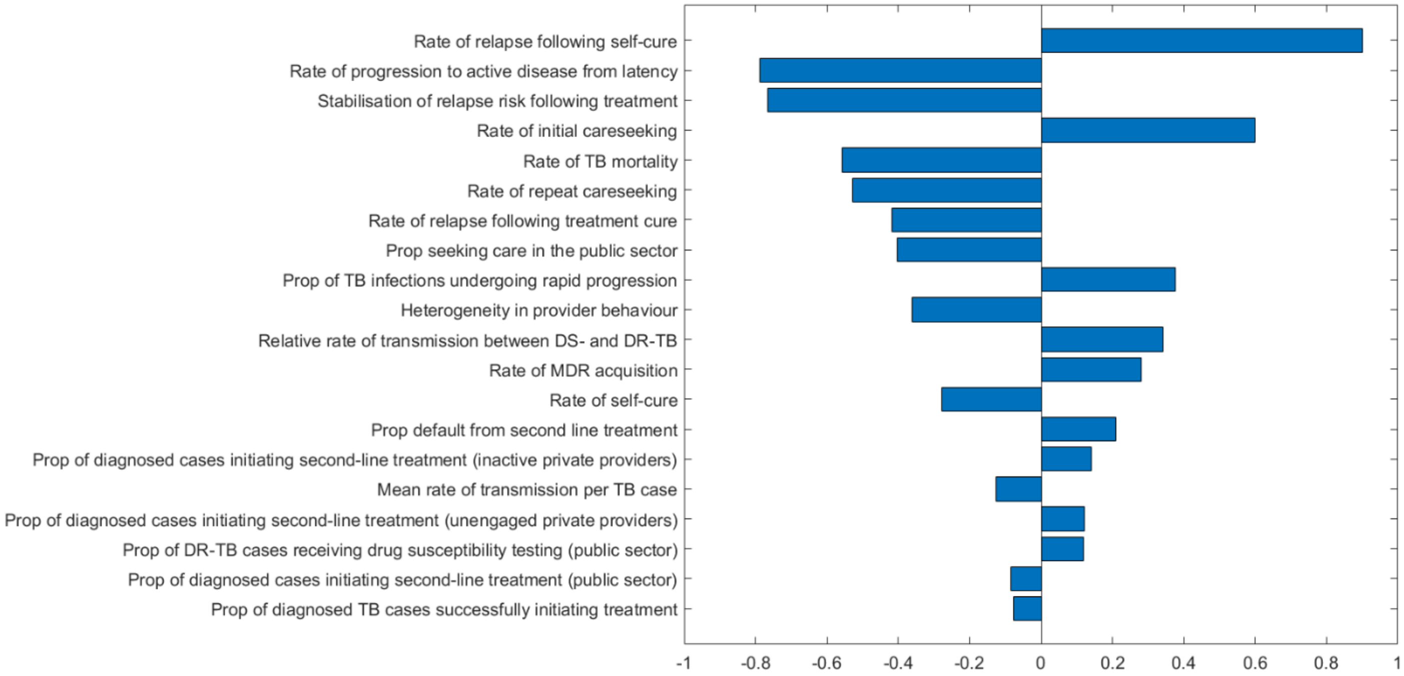

Figure S8. Multivariate sensitivity analysis of impact for Delhi. We used partial rank correlation coefficient (PRCC) to examine which parameter listed in tables 2-5 the output cases averted is most sensitive towards. Larger bars represent more sensitive parameters. Shown are the 20 most influential model parameters, in decreasing order of sensitivity from top to bottom.

For peer review only

3. Supporting tables

City	Number of identified private providers	Number of engaged private providers	Number of active private providers
Ahmedabad	1899	952	762
Delhi	2780	2016	894

Table S10. Number of identified, engaged, and active private providers in 2019 in Ahmedabad and Delhi. We assume that all private providers have been identified.

City	Heterogeneity index (α)
Ahmedabad	0.39 [0.32-0.46]
Delhi	0.32 [0.25-0.41]

Table S11. Heterogeneity index of private providers across Ahmedabad and Delhi. A heterogeneity index of 1 suggests that each provider sees the same number of patients (represented by the red line in fig S1). A smaller heterogeneity index indicates that a smaller number of providers are responsible for a larger number of patients. To calculate the heterogeneity, we divided the logarithm of the proportion of notifications by the logarithm of the proportion of providers responsible for those notifications.

Cost item	Total cost in 2019 (USD)	
	Ahmedabad	Delhi
Field officer salary	31,433	55,936
Hub agent salary	113,808	281,373
SCT agent salary	2055	5287
Treatment coordinator salary	8569	13,445
CMEs	460	6095
Promotional material	10	10
Sputum sample logistics	2020	5090
CB-NAAT tests	83,886	98,088
FDCs**	N/A	

Table S12. Total costs of PPSA activities in Ahmedabad and Delhi in 2019. We assume a conversion rate of USD 1 = 73 INR. *We assume a fix cost per FDC of 45 USD.

For peer review only

Category	Description
Provider activation	Costs associated with the engagement of inactive providers to encourage them to become active and start notifying their TB cases
Provider retention	Costs associated with the engagement of active providers to encourage them to remain active and keep notifying their TB cases
Diagnostic engagement	Costs associated with encouraging active providers to start using CB-NAAT, to increase their use of CB-NAAT and maintain their use of CB-NAAT
Diagnostic logistics	All other costs associated with CB-NAAT testing that are not to do with engagement. Includes cost of transporting sputum samples, cost of staff and cost of the CB-NAAT tests.
Treatment engagement	Costs associated with encouraging active providers to start using, increase or maintain their use of government supplied FDCs and adherence support.
Treatment logistics	Cost of government supplied FDCs and adherence support

Table S13. Costing categories. We categorise each cost item into one of the categories listed in this table to help identify the cost items that will increase as the coverage of the different interventions increase.

PPSA activity	Effort needed
Provider activation	5 (4-7) visits needed to onboard an inactive provider
Increasing CB-NAAT usage amongst occasional users	One visit increases a provider's CB-NAAT use by 0.2%. Assumed the same across all cities.
Converting non-CB-NAAT users to occasionally use CB-NAAT	7 (5-8) visits needed to convert a non-CB-NAAT user. Assumed the same across all cities
Converting non-FDC users to use FDC	6 (4-7) visits needed to convert a non-FDC user. Assumed the same across all cities.

Table S14. Engagement effort needed for each PPSA service provision in Ahmedabad and Delhi in 2019. As described in the text, values shown in these tables are essentially expert opinion, elicited through in-dept discussion with implementing teams from CHAI.

For peer review only

Ahmedabad						
**Cost item**	**Cost category**					
	Provider activation	Provider retention	Diagnostic engagement	Diagnostic logistics	Treatment engagement	Treatment logistics
Field officer	10%	42%	24%	-	24%	-
Hub agent	-	51%	-	24%	-	25%
SCT agent	-	-	-	100%	-	-
Treatment coordinator	-	-	-	-	-	100%
CMEs	-	40%	30%	-	30%	-
Promotional material	-	52%	29%	-	19%	-
Sputum sample logistics	-	-	-	100%	-	-
CB-NAAT	-	-	-	100%	-	-
FDCs	-	-	-	-	-	100%
**Delhi**						
**Cost item**	**Cost category**					
	Provider activation	Provider retention	Diagnostic engagement	Diagnostic logistics	Treatment engagement	Treatment logistics
Field officer	13%	28%	32%	-	27%	-
Hub agent	-	74%	-	18%	-	8%
SCT agent	-	-	-	100%	-	-
Treatment coordinator	-	-	-	-	-	100%
CMEs	-	40%	30%	-	30%	-
Promotional material	-	52%	29%	-	19%	-
Sputum sample logistics	-	-	-	100%	-	-
CB-NAAT	-	-	-	100%	-	-
FDCs	-	-	-	-	-	100%

Table S15. Costing split across PPSA activities for Ahmedabad and Delhi in 2019. The field officer split was determined by the percentage of calls that were made by a field officer for provider activation, provider retention, diagnostic engagement and treatment engagement; similarly, the hub agent split was determined by the proportion of time hub agents estimated they had spent on provider retention, diagnostic logistics and treatment logistics; the CMEs and promotional materials splits were determined by the amount of engagement that was aimed at provider retention, diagnostic engagement and treatment engagement, which was assumed constant across all cities. Each row adds up to 100%.

Cost item	Total cost	Category split	Unit description	No. of units	Cost per unit (USD)
Field officer	31,433	10%	No. of visits in 2019 made to onboard an inactive provider	1548	2.08
**PPSA activity: Provider retention**					
Field officer	31,433	42%	No. of active providers in 2019	762	17.77
Hub agent	113,808	51%	No. of active providers in 2019	762	78.12
CMEs	460	40%	No. of providers that attended CMEs in 2019	51	3.70
Promotional material	10	52%	No. of active providers in 2019	762	0.007
**PPSA activity: Diagnostic engagement**					
Field officer	31,433	24%	No. of visits in 2019 made to active providers to increase CB-NAAT usage	3572	2.17
			No. of active providers in 2019	762	10.15
CMEs	460	30%	No. of providers that attended CMEs in 2019	51	2.78
Promotional material	10	29%	No. of active providers in 2019	762	0.004
**PPSA activity: Diagnostic logistics**					
Hub agent	113,808	24%	No. of CB-NAAT tests performed in 2019	3615	7.75
SCT agent	2055	100%	No. of CB-NAAT tests performed in 2019	3615	0.58
Sputum sample logistics	2020	100%	No. of CB-NAAT tests performed in 2019	3615	0.57
CB-NAAT tests	83,886	100%	No. of CB-NAAT tests performed in 2019	3615	23.80
**PPSA activity: Treatment engagement**					
Field officer	31,433	24%	No. of visits in 2019 made to active providers to use FDCs	3619	2.14
			No. of active providers in 2019	762	10.15
CMEs	460	30%	No. of providers that attended CMEs in 2019	51	2.78
Promotional material	10	19%	No. of active providers in 2019	762	0.003
**PPSA activity: Treatment logistics**					
Treatment coordinator	8569	100%	No. of patient treatment months in 2019	57,868	0.15
Hub agent	113,808	25%	No. of FDC initiations in 2019	3445	8.47
FDCs	-	-	-	-	45

Table S16. Unit costs for Ahmedabad

For peer review only - http://bmjopen.bmj.com/site/about/guidelines.xhtml

Cost item	Total cost	Category split	Unit description	No. of units	Cost per unit (USD)
**PPSA activity: Provider activation**					
Field officer	55,936	13%	No. of visits in 2019 made to onboard an inactive provider	2984	2.50
**PPSA activity: Provider retention**					
Field officer	55,936	28%	No. of active providers in 2019	894	17.97
Hub agent	281,373	74%	No. of active providers in 2019	894	238.88
CMEs	6095	40%	No. of providers that attended CMEs in 2019	1469	1.70
Promotional material	10	52%	No. of active providers in 2019	894	0.006
**PPSA activity: Diagnostic engagement**					
Field officer	55,936	32%	No. of visits in 2019 made to active providers to increase CB-NAAT usage	7219	2.54
			No. of active providers in 2019	894	20.54
CMEs	6095	30%	No. of providers that attended CMEs in 2019	1469	1.28
Promotional material	10	29%	No. of active providers in 2019	894	0.003
**PPSA activity: Diagnostic logistics**					
Hub agent	281,373	18%	No. of CB-NAAT tests performed in 2019	4227	12.29
SCT agent	5287	100%	No. of CB-NAAT tests performed in 2019	4227	1.28
Sputum sample logistics	5090	100%	No. of CB-NAAT tests performed in 2019	4227	1.23
CB-NAAT tests	98,088	100%	No. of CB-NAAT tests performed in 2019	4227	23.80
**PPSA activity: Treatment engagement**					
Field officer	55,936	27%	No. of visits in 2019 made to active providers to use FDCs	5863	2.64
			No. of active providers in 2019	894	17.33
CMEs	6095	30%	No. of providers that attended CMEs in 2019	1469	1.28
Promotional material	10	19%	No. of active providers in 2019	894	0.002
**PPSA activity: Treatment logistics**					
Treatment coordinator	13,445	100%	No. of patient treatment months in 2019	108,915	0.13
Hub agent	281,373	8%	No. of FDC initiations in 2019	1480	13.65
FDCs	-	-	-	-	45

Table S17. Unit costs for Delhi.

Intervention	Incremental cost (2020-2035, USD)	Percentage of cumulative TB cases averted (2020-2035)	Cost-efficiency
**Individual interventions**			
Increasing the number of active providers (assuming newly active providers adopt average behaviour)	2,269,300 (1,477,000 – 3,723,000)	3.00% (2.59-3.37)	0.0032 (0.0019- 0.0050)
Increasing the number of active providers (assuming newly active providers' behaviour is 25% worse than average)	2,224,400 (1,435,200 – 3,672,300)	2.98% (2.58-3.35)	0.0032 (0.0019- 0.0051)
Increasing the number of active providers (assuming newly active providers' behaviour is 50% worse than average)	2,120,000 (1,339,000 – 3,555,300)	2.89% (2.48-3.24)	0.0033 (0.0019- 0.0053)
Increasing CB-NAAT use amongst occasional users	9,005,000 (6,801,000 – 13,190,000)	2.83% (2.42-3.33)	0.0008 (0.0006- 0.0010)
Increasing the number of occasional CB-NAAT users	2,211,700 (1,683,500 – 3,275,100)	2.32% (1.97-2.67)	0.0025 (0.0018- 0.0033)
Increasing the number of FDC users	1,787,700 (1,438,900 – 2,503,800)	2.53% (2.13-2.91)	0.0031 (0.0024- 0.0041)
**Combination of the two most cost-efficient interventions: Increasing the number of active providers* + …**			
+Increasing the number of occasional CB-NAAT users	5,020,300 (3,590,000 – 7,512,900)	3.21% (2.84-3.61)	0.0015 (0.0010- 0.0022)
+ Increasing CB-NAAT use amongst occasional users	13,427,000 (10,019,000 – 19,839,000)	3.81% (3.40-4.45)	0.0007 (0.0005- 0.0009)
+ Increasing the number of FDC users	5,217,100 (3,791,600 – 8,182,300)	3.46% (2.96-3.91)	0.0016 (0.0010- 0.0022)
**Combination of the three most cost-efficient interventions: Increasing the number of active providers* + the number of FDC-users + …**			
+Increasing the number of occasional CB-NAAT users	7,163,000 (5,671,000 – 10,478,000)	3.67% (3.18-4.16)	0.0012 (0.0008- 0.0017)
+ Increasing CB-NAAT use amongst occasional users	15,467,000 (11,921,000 – 22,411,000)	4.27% (3.81-4.87)	0.0007 (0.0005- 0.0009)
**Combination of all interventions***			
+ Increasing CB-NAAT use amongst occasional users	27,976,000 (21,331,000 – 40,913,000)	5.17% (4.49-6.17)	N/A

Table S18. Cost efficiency of service provisions in Ahmedabad. We also conducted a sensitivity analysis on the behaviour of newly active providers, and whether increasing the number of active providers remains the most cost-efficient individual intervention if the behaviour of newly active providers is assumed to be 25% and 50% worse than pre-existing active providers. Indeed, even if the behaviour of newly active providers is 50% worse, this intervention is still the most cost-efficient individual intervention when scaled up to its feasible maximum.
*We assume that these newly active providers adopt average behaviour.

For peer review only

Intervention	Incremental cost (2020-2035, USD)	Percentage of cumulative TB cases averted (2020-2035)	Cost-efficiency
**Individual interventions**			
Increasing the number of active providers (assuming newly active providers adopt average behaviour)	20,760,000 (12,478,000 – 36,094,000)	3.06% (2.43-3.98)	0.0003 (0.0002- 0.0006)
Increasing CB-NAAT use amongst occasional users	22,249,000 (13,546,000 – 30,724,000)	1.11% (0.97-1.27)	0.0001 (0.0001- 0.0002)
Increasing the number of occasional CB-NAAT users	4,920,900 (2,985,500 – 6,819,600)	0.86% (0.73-1.03)	0.0004 (0.0003- 0.0006)
Increasing the number of FDC users	4,496,400 (3,283,400 – 5,866,200)	1.13% (0.97-1.34)	0.0006 (0.0004- 0.0007)
**Combination of the two most cost-efficient interventions: Increasing the number of FDC-users + …**			
+Increasing the number of active providers	30,981,000 (21,744,000-46,622,000)	3.80% (2.92-5.06)	0.00026 (0.00017- 0.0046)
+Increasing the number of occasional CB-NAAT users	9,250,000 (6,796,000 – 12,272,000)	1.21% (1.05-1.41)	0.00028 (0.00021-0.00038)
+ Increasing CB-NAAT use amongst occasional users	26,794,000 (17,716,000 – 36,310,000)	1.47% (1.29-1.72)	0.00012 (0.00008-0.00017)
**Combination of the three most cost-efficient interventions: Increasing the number of FDC users + increasing the number of occasional CB-NAAT users + …**			
+ Increasing the number of active providers	41,648,000 (30,441,000 - 60,488,000)	3.95% (3.05-5.22)	0.00020 (0.00013 – 0.00033)
+ Increasing CB-NAAT use amongst occasional users	60,238,000 (37,969,000 – 82,081,000)	1.96% (1.64-2.35)	0.00007 (0.00005 – 0.00010)
**Combination of all interventions**			
+ Increasing CB-NAAT use amongst occasional users	157,450,000 (100,600,000 – 213,200,000)	5.35% (4.30-7.00)	N/A

Table S19. Cost efficiency of service provisions in Delhi. *We assume that these newly active providers adopt average behaviour.

For peer review only

Cost category	Baseline	Increasing no. of active providers	Increasing CB-NAAT usage amongst occasional CB-NAAT users	Increasing the no. of occasional CB-NAAT users	Increasing the no. of providers offering FDCs
**Provider retention**	1.2 million	1.5 million	1.2 million	1.2 million	1.2 million
**Onboarding engagement**	N/A	2,000	N/A	N/A	N/A
**CB-NAAT engagement**	12,000	15,000	0.3 million	14,000	12,000
**FDC engagement**	11,000	14,000	11,000	11,000	17,000
**CB-NAAT logistics**	6.0 million (4.6 – 8.9 million)	7.6 million (5.7 – 11.0 million)	15.0 million (12.2 – 23.8 million)	8.5 million (6.4 – 12.7 million)	6.0 million (4.7 – 8.9 million)
**Adherence support**	0.1 million (0.1 – 0.2 million)	0.2 million (0.1 – 0.3 million)	0.1 million (0.1 – 0.2 million)	0.1 million (0.1 – 0.2 million)	0.1 million (0.1 – 0.2 million)
**FDCs**	5.5 million (3.2 – 9.0 million)	6.7 million (4.1 – 11.0 million)	4.3 million (2.7 – 7.0 million)	5.2 million (3.1 – 8.4 million)	7.4 million (4.9 – 11.6 million)
**Public sector diagnoses**	35.0 million (26.6 – 50.4 million)	35.0 million (25.2 – 50.4 million)	35.0 million (25.2 – 50.4 million)	35.0 million (26.6 – 50.4 million)	35.0 million (26.6 – 50.4 million)
**Public sector treatment**	68.6 million (53.2– 93.8 million)	68.6 million (53.2– 92.4 million)	68.6 million (53.2– 93.8 million)	68.6 million (53.2– 93.8 million)	68.6 million (53.2– 93.8 million)

Table S20. Breakdown of total costs across each intervention between 2020 and 2035 for Ahmedabad.

For peer review only

Cost category	Baseline	Increasing no. of active providers	Increasing CB-NAAT usage amongst occasional CB-NAAT users	Increasing the no. of occasional CB-NAAT users	Increasing the no. of providers offering FDCs
**Provider retention**	3.5 million	8.0 million	3.5 million	3.5 million	3.5 million
**Onboarding engagement**	N/A	14,000	N/A	N/A	N/A
**CB-NAAT engagement**	22,000	50,000	0.3 million	32,000	22,000
**FDC engagement**	18,000	41,000	18,000	18,000	32,000
**CB-NAAT logistics**	8.1 million (4.9 – 11.0 million)	18.0 million (11.2 – 25.2 million)	32.2 million (19.6 – 44.8 million)	13.5 million (8.3 – 18.2 million)	8.1 million (4.9 – 11.0 million)
**Adherence support**	0.3 million (0.2 – 0.4 million)	0.6 million (0.3 – 0.9 million)	0.2 million (0.1 – 0.4 million)	0.3 million (0.2 – 0.4 million)	0.3 million (0.2 – 0.4 million)
**FDCs**	10.2 million (5.3 – 19.6 million)	23.8 million (11.9 – 44.8 million)	7.7 million (4.2 – 15.4 million)	9.7 million (5.0 – 19.6 million)	15.4 million (9.8 – 25.2 million)
**Public sector diagnoses**	117.6 million (74.2 – 154 million)	116.2 million (72.8 – 154 million)	116.2 million (72.8 – 154 million)	117.6 million (74.2 – 154 million)	117.6 million (74.2 – 154 million)
**Public sector treatment**	224 million (168 million – 294 million)	224 million (168 million – 294 million)	224 million (168 million – 294 million)	224 million (168 million – 294 million)	224 million (168 million – 294 million)

Table S21. Breakdown of total costs across each intervention between 2020 and 2035 for Delhi.

For peer review only

Intervention	Maintenance costs sensitivity analysis	Incremental cost (2020-2035, USD)	Percentage of cumulative TB cases averted (2020-2035)	Cost-efficiency
Increasing the number of active providers	*-20%:*	1,963,400 (1,170,200-3,416,300)	3.00% (2.59-3.37)	0.0037 (0.0021-0.0063)
	+20%	2,575,300 (1,782,200-4,028,200)		0.0028 (0.0017-0.0041)
Increasing the number of occasional CB-NAAT users	*-20%:*	1,965,900 (1,440,400-3,030,200)	2.32% (1.97-2.67)	0.0028 (0.0020-0.0038)
	+20%	2,455,700 (1,930,200-3,520,000)		0.0023 (0.0017-0.0029)
Increasing CB-NAAT use amongst occasional users	*-20%:*	8,743,000 (6,671,000-13,031,000)	2.83% (2.42-3.33)	0.0008 (0.0006-0.0010)
	+20%	9,232,000 (7,160,000-13,521,000)		0.0007 (0.0006-0.0009)
Increasing the number of FDC users	*-20%:*	1,541,800 (1,194,000-2,260,900)	2.53% (2.13-2.91)	0.0039 (0.0026-0.0050)
	+20%	2,031,600 (1,683,800-2,750,700)		0.0027 (0.0022-0.0036)

Table S22. Sensitivity analysis on the cost of maintenance in Ahmedabad. We explored whether increasing or decreasing maintenance cost by 20% effected the cost-efficiency order of individual service provisions. Maintenance costs include the cost of provider retention, the cost of CB-NAAT engagement to maintain CB-NAAT use, and the cost of FDC engagement to maintain FDC use.

For peer review only

Intervention	Maintenance costs sensitivity analysis	Incremental cost (2020-2035, USD)	Percentage of cumulative TB cases averted (2020-2035)	Cost-efficiency
Increasing the number of active providers	*-20%:*	19,145,000 (10,865,000 – 34,481,000)	3.06% (2.43-3.98)	0.0003 (0.0002-0.0007)
	+20%	22,375,000 (14,095,000 – 37,712,000)		0.0003 (0.0002 – 0.0006)
Increasing the number of occasional CB-NAAT users	*-20%:*	4,205,900 (2,272,200 – 6,104,300)	0.86% (0.73-1.03)	0.0004 (0.0003-0.0008)
	+20%	5,638,400 (3,704,700 – 7,536,800)		0.0003 (0.0002-0.0005)
Increasing CB-NAAT use amongst occasional users	*-20%:*	21,455,000 (12,823,000 – 29,889,000)	1.11% (0.97-1.27)	0.0001 (0.0001-0.0002)
	+20%	22,887,000 (14,255,000– 31,321,000)		0.0001 (0.0001-0.0002)
Increasing the number of FDC users	*-20%:*	3,782,600 (2,569,100 – 5,154,300)	1.13% (0.97-1.34)	0.0007 (0.0005-0.0009)
	+20%	5,215,100 (4,001,600 – 6,586,800)		0.0005 (0.0004-0.0006)

Table S23. Sensitivity analysis on the cost of maintenance in Delhi. We explored whether increasing or decreasing maintenance cost by 20% effected the cost-efficiency order of individual service provisions. Maintenance costs include the cost of provider retention, the cost of CB-NAAT engagement to maintain CB-NAAT use, and the cost of FDC engagement to maintain FDC use.

For peer review only

4. Bibliography

1. Mistry N, Lobo E, Shah S, Rangan S, Dholakia Y. Pulmonary Tuberculosis in Patna, India: Durations, Delays, and Health Care Seeking Behaviour Among Patients Identified Through Household Surveys. *Journal of Epidemiology and Global Health*. 2017;7(4): 241–248.

2. Veesa KS, John KR, Moonan PK, Kaliappan SP, Manjunath K, Sagili K, et al. Diagnostic Pathways and Direct Medical Costs Incurred by New Adult Pulmonary Tuberculosis Patients Prior to Anti-Tuberculosis Treatment - Tamil Nadu, India. *PLOS ONE*. 2018;13(2): e0191591.

3. Pandey A, Ploubidis GB, Clarke L, Dandona L. Hospitalisation Trends in India From Serial Cross-Sectional Nationwide Surveys: 1995 to 2014. *BMJ Open*. 2017;7(12): e014188.

4. Sachdeva KS, Satyanarayana S, Dewan P, Nair SA, Reddy R, Kundu D, et al. Source of Previous Treatment for Re-Treatment TB Cases Registered under the National TB Control Programme, India, 2010. *PLOS ONE*. 2011;6(7): e22061.

5. Satyanarayana S, Nair SA, Chadha SS, Shivashankar R, Sharma G, Yadav S, et al. From Where Are Tuberculosis Patients Accessing Treatment in India? Results From a Cross-Sectional Community Based Survey of 30 Districts. *PLOS ONE*. 2011;6(9): e24160.

6. Sharma G, Sinha A, Bastian S, Santha T. Knowledge and behaviour of chest symptomatics in urban slum populations of two states in India towards care-seeking. *The Indian Journal of Tuberculosis*. 2013;60(2): 95–106.

7. Satyanarayana S, Subbaraman R, Shete P, Gore G, Das J, Cattamanchi A, et al. Quality of Tuberculosis Care in India: A Systematic Review. *International Journal of Tuberculosis and Lung Disease*. 2015;19(7): 751–763.

8. World Health Organisation. *A brief history of tuberculosis control in India.* 2010. https://apps.who.int/iris/bitstream/handle/10665/44408/9789241500159_eng.pdf?sequence=1

9. Menzies D, Benedetti A, Paydar A, Martin I, Royce S, Pai M, et al. Effect of Duration and Intermittency of Rifampin on Tuberculosis Treatment Outcomes: A Systematic Review and Meta-Analysis. *PLOS Med*. 2009;6(9): e1000146. https://doi.org/10.1371/journal.pmed.1000146.

10. Vynnycky E, Fine PE. The natural history of tuberculosis: the implications of age-dependent risks of disease and the role of reinfection. *Epidemiology and Infection*. 1997;119(2): 183–201.

11. Dye C, Garnett GP, Sleeman K, Williams BG. Prospects for worldwide tuberculosis control under the WHO DOTS strategy. Directly observed short-course therapy. *Lancet (London, England)*. 1998;352(9144): 1886–1891.

12. Horsburgh CR, O'Donnell M, Chamblee S, Moreland JL, Johnson J, Marsh BJ, et al. Revisiting rates of reactivation tuberculosis: a population-based approach. *American Journal of Respiratory and Critical Care Medicine*. 2010;182(3): 420–425. https://doi.org/10.1164/rccm.200909-1355OC.

13. Thomas A, Gopi PG, Santha T, Chandrasekaran V, Subramani R, Selvakumar N, et al. Predictors of relapse among pulmonary tuberculosis patients treated in a DOTS programme in South India. *International Journal of Tuberculosis and Lung Disease*. 2005;9(5): 556–561.

14. Tiemersma EW, van der Werf MJ, Borgdorff MW, Williams BG, Nagelkerke NJD. Natural history of tuberculosis: duration and fatality of untreated pulmonary tuberculosis in HIV negative patients: a systematic review. *PloS One*. 2011;6(4): e17601. https://doi.org/10.1371/journal.pone.0017601.

15. Andrews JR, Noubary F, Walensky RP, Cerda R, Losina E, Horsburgh CR. Risk of progression to active tuberculosis following reinfection with Mycobacterium tuberculosis. *Clinical Infectious Diseases*. 2012;54(6): 784–791.

16. World Bank. *Data: India*. 2015 [Accessed 12th May 2016]. http://data.worldbank.org/country/india [Accessed 12th May 2016].

For peer review only

17. Pandey S, Chadha VK, Laxminarayan R, Arinaminpathy N. Estimating tuberculosis incidence from primary survey data: a mathematical modeling approach. *The International Journal of Tuberculosis and Lung Disease*. 2017;21(4): 366–374. https://doi.org/10.5588/ijtld.16.0182.

18. Sachdeva KS, Satyanarayana S, Dewan P, Nair SA, Reddy R, Kundu D, et al. Source of Previous Treatment for Re-Treatment TB Cases Registered under the National TB Control Programme, India, 2010. *PLOS ONE*. 2011;6(7): e22061.

19. Satyanarayana S, Nair SA, Chadha SS, Shivashankar R, Sharma G, Yadav S, et al. From Where Are Tuberculosis Patients Accessing Treatment in India? Results from a Cross-Sectional Community Based Survey of 30 Districts. *PLOS ONE*. 2011;6(9): e24160. https://doi.org/10.1371/journal.pone.0024160.

20. Subbaraman R, Nathavitharana RR, Satyanarayana S, Pai M, Thomas BE, Chadha VK, et al. The Tuberculosis Cascade of Care in India's Public Sector: A Systematic Review and Meta-analysis. *PLOS Medicine*. 2016;13(10): e1002149. https://doi.org/10.1371/journal.pmed.1002149.

21. Van't Hoog A, Langendam MW, Mitchell E, Cobelens FG, Sinclair D, Leeflang MMG, et al. *A sytematic review of the sensitivity and specificity of symptom- and chest-radiography screening for active pulmonary tuberculosis in HIV-negative persons and persons with unknown HIV status*. 2013. https://www.who.int/tb/Review2Accuracyofscreeningtests.pdf

22. Steingart KR, Horne DJ, Pai M, Boehme CC, Dendukuri N. Xpert MTB/RIF assay for pulmonary tuberculosis and rifampicin resistance in adults (Review). *Cochrane Database of Systematic Reviews*. 2014;(1).

23. World Health Organisation. *Global Tuberculosis Report 2020*. 2020. https://www.who.int/publications/i/item/9789240013131

24. Subbaraman R, Nathavitharana RR, Mayer KH, Satyanarayana S, Chadha VK, Arinaminpathy N, et al. Constructing care cascades for active tuberculosis: A strategy for program monitoring and identifying gaps in quality of care. *PLOS Medicine*. 2019;16(2): e1002754.

25. Menzies NA, Gomez GB, Bozzani F, Chatterjee S, Foster N, Baena IG, et al. Cost-effectiveness and resource implications of aggressive action on tuberculosis in China, India, and South Africa: a combined analysis of nine models. *The Lancet Global Health*. 2016;4(11): e816–e826. https://doi.org/10.1016/S2214-109X(16)30265-0.

26. Houben RMGJ, Lalli M, Kranzer K, Menzies NA, Schumacher SG, Dowdy DW. What if they don't have tuberculosis? The consequences and trade-offs involved in false-positive diagnoses of tuberculosis. *Clinical Infectious Diseases*. 2019;68(1): 150–156. https://doi.org/10.1093/cid/ciy544.

27. Fochsen G, Deshpande K, Diwan V, Mishra A, Diwan VK, Thorson A. Health care seeking among individuals with cough and tuberculosis: a population-based study from rural India. *International Journal of Tuberculosis and Lung Disease*. 2006;10(9): 995–1000.

28. Gopi PG, Subramani R, Narayanan PR. Evaluation of different types of chest symptoms for diagnosing pulmonary tuberculosis cases in community surveys. *Indian Journal of Tuberculosis*. 2008;55(3): 116–121.

29. Rao VG, Bhat J, Yadav R, Gopalan GP, Nagamiah S, Bhondeley MK, et al. Prevalence of pulmonary tuberculosis - a baseline survey in central India. *PLOS One*. 2012;7(8): e43225. https://doi.org/10.1371/journal.pone.0043225.

30. Sachdeva KS, Raizada N, Sreenivas A, Van't Hoog A, van den Hof S, Dewan PK, et al. Use of Xpert MTB/RIF in decentralized public health settings and its effect on pulmonary TB and DR-TB case findings in India. *PLOS ONE*. 2015;10(5): e0126065. https://doi.org/10.1371/journal.pone.0126065.

31. Haario H, Saksman E, Tamminen J. An adaptive metropolis algorithm. *Bernoulli*. 2001;7(2): 223–242.

For peer review only

For peer review only

CHEERS 2022 Checklist

	Item	Guidance for Reporting	Reported in section
**TITLE**			
Title	1	Identify the study as an economic evaluation and specify the interventions being compared.	N/A
**ABSTRACT**			
Abstract	2	Provide a structured summary that highlights context, key methods, results and alternative analyses.	Page 1
**INTRODUCTION**			
Background and objectives	3	Give the context for the study, the study question and its practical relevance for decision making in policy or practice.	Page, 1-2
**METHODS**			
Health economic analysis plan	4	Indicate whether a health economic analysis plan was developed and where available.	Page 5-6
Study population	5	Describe characteristics of the study population (such as age range, demographics, socioeconomic, or clinical characteristics).	Table 1-2
Setting and location	6	Provide relevant contextual information that may influence findings.	Table 1and page 3-4
Comparators	7	Describe the interventions or strategies being compared and why chosen.	Page 6
Perspective	8	State the perspective(s) adopted by the study and why chosen.	Page 5
Time horizon	9	State the time horizon for the study and why appropriate.	Page 5
Discount rate	10	Report the discount rate(s) and reason chosen.	Page 6
Selection of outcomes	11	Describe what outcomes were used as the measure(s) of benefit(s) and harm(s).	Page 6
Measurement of outcomes	12	Describe how outcomes used to capture benefit(s) and harm(s) were measured.	Page 6
Valuation of outcomes	13	Describe the population and methods used to measure and value outcomes.	N/A
Measurement and valuation of resources and costs	14	Describe how costs were valued.	Table S8-13
Currency, price date, and conversion	15	Report the dates of the estimated resource quantities and unit costs, plus the currency and year of conversion.	Table S8
Rationale and description of model	16	If modelling is used, describe in detail and why used. Report if the model is publicly available and where it can be accessed.	Model is fully specified in the manuscript materials
Analytics and assumptions	17	Describe any methods for analysing or statistically transforming data, any extrapolation methods, and approaches for validating any model used.	Figure S1, table S10, page 6
Characterizing heterogeneity	18	Describe any methods used for estimating how the results of the study vary for sub-groups.	Page 6-7
Characterizing distributional effects	19	Describe how impacts are distributed across different individuals or adjustments made to reflect priority populations.	N/A
Characterizing uncertainty	20	Describe methods to characterize any sources of uncertainty in the analysis.	Page 6-7
Approach to engagement with patients and others affected by the study	21	Describe any approaches to engage patients or service recipients, the general public, communities, or stakeholders (e.g., clinicians or payers) in the design of the study.	N/A
**RESULTS**			
Study parameters	22	Report all analytic inputs (e.g., values, ranges, references) including uncertainty or distributional assumptions.	Table 1-2
Summary of main results	23	Report the mean values for the main categories of costs and outcomes of interest and summarise them in the most appropriate overall measure.	Table 3, S14-15
Effect of uncertainty	24	Describe how uncertainty about analytic judgments, inputs, or projections affect findings. Report the effect of choice of discount rate and time horizon, if applicable.	Page 9-10, fig S6-8
Effect of engagement with patients and others affected by the study	25	Report on any difference patient/service recipient, general public, community, or stakeholder involvement made to the approach or findings of the study	N/A
**DISCUSSION**			
Study findings, limitations, generalizability, and current knowledge	26	Report key findings, limitations, ethical or equity considerations not captured, and how these could impact patients, policy, or practice.	Page 8
**OTHER RELEVANT INFORMATION**			
Source of funding	27	Describe how the study was funded and any role of the funder in the identification, design, conduct, and reporting of the analysis	
Conflicts of interest	28	Report authors conflicts of interest according to journal or International Committee of Medical Journal Editors requirements.	

Husereau D, Drummond M, Augustovski F, de Bekker-Grob E, Briggs AH, Carswell C, Caulley L, Chaiyakunapruk N, Greenberg D, Loder E, Mauskopf J, Mullins CD, Petrou S, Pwu RF, Staniszewska S; CHEERS 2022 ISPOR Good Research Practices Task Force. Consolidated Health Economic Evaluation Reporting Standards 2022 (CHEERS 2022) Statement: Updated Reporting Guidance for Health Economic Evaluations. BMJ. 2022;376:e067975.
The checklist is Open Access distributed in accordance with the terms of the Creative Commons Attribution (CC BY 4.0) license, which permits others to distribute, remix, adapt and build upon this work, for commercial use, provided the original work is properly cited. See: http://creativecommons.org/licenses/by/4.0/.

For peer review only
